# Scorpionate Complexes [(κ^*n*^‑Tp^Ph,Me^)NiS_2_CNR_2_] (*n* = 2, 3) as a Structural and Spectroscopic Model for Reduced Nickel-Dependent Superoxide Dismutase

**DOI:** 10.1021/acs.inorgchem.5c05781

**Published:** 2026-02-11

**Authors:** Huaibo Ma, Haoshuang Wang, Javad Shokraiyan, Jeffrey L. Petersen, Gregory T. Rohde, Victor G. Young, Michael P. Jensen

**Affiliations:** † Department of Chemistry & Biochemistry, 1354 Ohio University, Athens, Ohio 45701, United States; ‡ C. Eugene Bennett Department of Chemistry, 5631 West Virginia University, Morgantown, West Virginia 26506, United States; § X-Ray Crystallographic Facility, Department of Chemistry, 5635 University of Minnesota Twin Cities, Minneapolis, Minnesota 55455, United States

## Abstract

Scorpionato-dithiocarbamate Ni­(II) complexes [(Tp^Ph,Me^)­NiS_2_CNR_2_], where Tp^Ph,Me^ = hydrotris­(3-phenyl-5-methylpyrazol-1-yl)­borate and NR_2_ = NMe_2_, NEt_2_, NPh_2_, carbazolyl, are advanced as structural and spectroscopic models for the active site of nickel-dependent superoxide dismutase in its reduced state. The Tp^Ph,Me^ ligand can adopt variable κ^2^- and κ^3^-coordination modes in the synthetic complexes that give rise to several ligand field conformations: square-planar, elongated square-pyramidal, square-pyramidal, and trigonal-bipyramidal. The first two are red and diamagnetic (*S* = 0), and the latter two are green and paramagnetic (*S* = 1). The different spin states are facilitated by a range of resonance structures within the dithiocarbamate coligand. These different geometric and spin isomers have been captured in the solid state and structurally characterized by X-ray crystallography. Electronic structures of a simplified [(Tp)­NiS_2_CNMe_2_] model in relevant conformations, spin and oxidation states were calculated using Density Functional Theory and compared to the enzyme. Reversible one-electron redox couples were observed at biologically relevant potentials. We propose that the diamagnetic, square-planar and elongated square-pyramidal conformations at Ni­(II) are relevant to the base-off and base-on conformations, respectively, reported for NiSOD.

## Graphical abstract

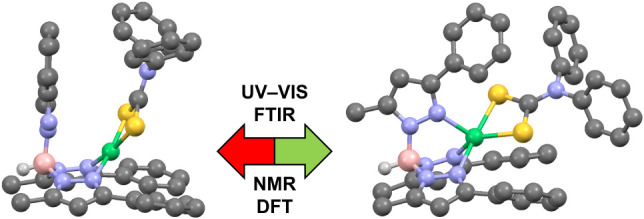

## 1 Introduction

Organisms that inhabit aerobic environments are exposed to oxidative stress arising from reactive oxygen species (“ROS”) that are generated by incomplete reduction of O_2_.^1^ The superoxide radical anion (O_2_^–,·^) is considered to be the primary ROS because it promotes the Fenton reaction to form hydroxyl radical (HO^·^), and it couples with nitric oxide to form peroxynitrite (ONOO^–^), which is another potent oxidant and reactive nitrogen species.^2^ Superoxide dismutases (SODs) are metal-dependent enzymes that catalyze dismutation of superoxide radicals to oxygen and hydrogen peroxide (eq 1) at diffusion-controlled rates. This activity inhibits oxidative stress by reducing the concentration and lifetime of superoxide.^3–6^ Rapid turnover depends on a one-electron couple at a catalytic metal ion, which is poised at a potential between those of the redox half-reactions of the superoxide substrate.^3,6^


1 $$2{O_{2}}^{-}+2H^{+}\to O_{2}+H_{2}O_{2}$$

Eukaryotes, including humans, express two distinct SODs.^4^ A manganese-dependent enzyme (MnSOD) is found in mitochondria. The Mn­(III) ion of the oxidized state resides in a trigonal-bipyramidal ligand field consisting of three imidazole nitrogens from histidine side chains, a carboxylate anion from an aspartate side chain, and a hydroxide ligand.^2–4,7^ A structurally distinct Cu/ZnSOD is found primarily in the cytoplasm and on exterior cell surfaces.^3,4^ The oxidized active site contains a Cu­(II) ion that is coordinated by four histidine residues, of which one bridges to an inert Zn­(II) ion.^3,8,9^ The superoxide dismutase activity of this enzyme was the first to be recognized in 1969.^10^ An iron-dependent enzyme that is homologous to MnSOD is found in bacteria and in plant chloroplasts.^4,6^ FeSOD is thought to be ancient, and the closely related Mn-dependent enzyme presumably evolved as the biosphere continued to oxidize and soluble Fe­(II) was depleted.^6^

Most recently, a third class of nickel-dependent SOD was discovered,^3–5,11–14^ which is found in some bacteria and cyanobacteria.^15,16^ X-ray crystal structures of two isoforms of NiSOD, reported in 2004, revealed an unprecedented active site (Figure 1).^17–22^ The first nine amino acids are a conserved H_2_N–HCXXPCXXY-sequence that forms a so-called “nickel hook” to chelate nickel. The Cys-2 and Cys-6 side chain thiolates act as equatorial ligands, along with the *N*-terminal amine and the adjacent amide anion to form an N_2_S_2_ square plane. Two conformations were observed: the His-1 side chain was either protonated at N_δ_ and hydrogen-bonded to an adjacent backbone carbonyl, or it was deprotonated and associated with the axial position of nickel, albeit with curiously long Ni–N bond distances of 2.35(5) and 2.63 Å observed in the different crystal structures.^17,19^

**Figure 1:**
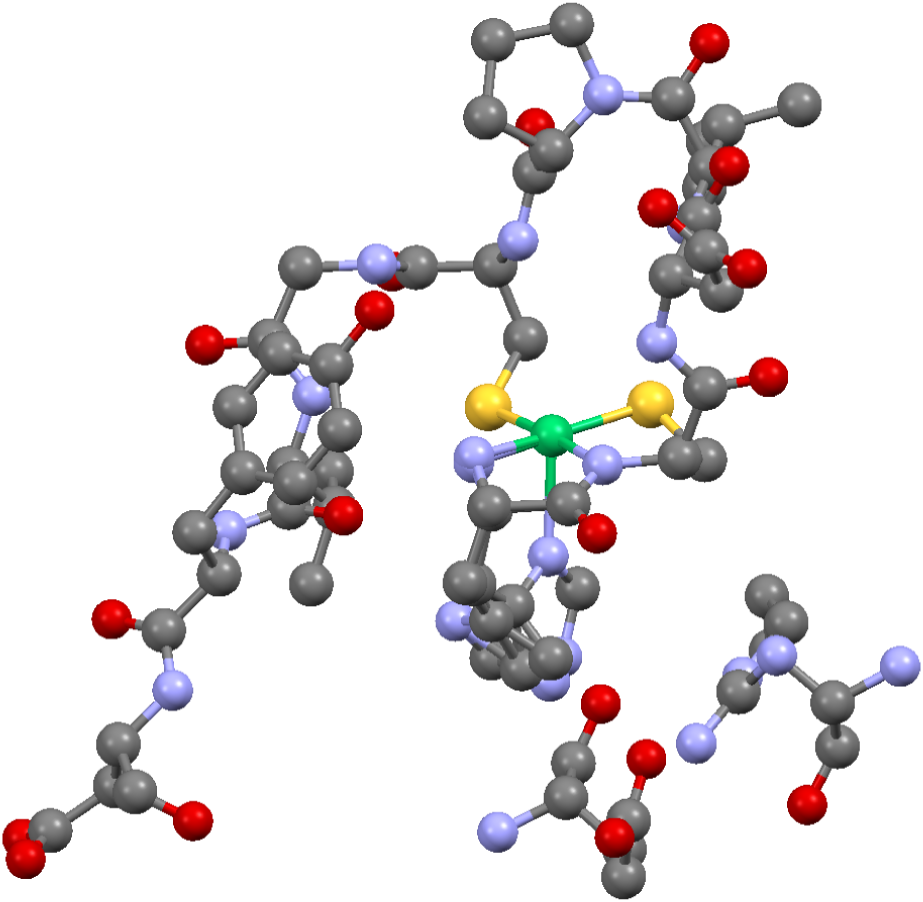
Active site nickel hook structure of NiSOD (1T6U.pdb).^17,18^ Hydrogen atoms are omitted for clarity. In addition to the first ten *N*-terminal residues and the coordinated nickel (rendered in green, center), the Glu-17 and Arg-47 residues from a flanking subunit are included (lower right), and these interact with the protonated N_ε_ of the His-1 imidazole ring. Both conformations of the latter are shown (bottom center). Reproduced from reference 17, Copyright 2004, American Chemical Society.

The NiSOD enzyme is isolated as a ca. 50:50 mixture of Ni­(II) and Ni­(III), as determined by EPR integration.^23^ The oxidized fraction exhibits a definitive low-spin (*S* = 1/2) rhombic EPR signal with distinctive hyperfine coupling to an axial nitrogen atom,^11,12,23^ as well as a characteristic UV LMCT absorption band at 378 nm and a visible shoulder near 530 nm.^11,12,24^ Barondeau et al. observed comparable fractions of base-off, square-planar and elongated square-pyramidal conformations by X-ray crystallography, and suggested they correspond to Ni­(II) and Ni­(III) oxidation states, respectively.^17^ This assignment was made notwithstanding an axial Ni–N bond length of 2.35(5) Å in the latter, “a much longer bond length to Ni^3+^ than typical”. Wuerges et al. further reported that the base-off form accumulated with X-ray exposure.^19^ They were able to obtain the base-on form alone at low exposures, and noted that the observed axial Ni–N distance of 2.63 Å “exceeds by ≈0.5 Å the commonly found bond length between Ni­(III) and N_δ_ of imidazolates and may indicate that reduction occurs already at low X-ray doses.” Nevertheless, the base-on and base-off conformations were respectively referred to as “oxidized” and “reduced” in a subsequent review by the same research group.^20–22^

SOD activity generally consists of diffusion-controlled oxido-reductase turnover, in which a one-electron redox couple of the active-site metal ion sustains a ping-pong mechanism (eqs 2, 3).^3–5,17,19^ Both redox half-reactions are coupled to proton transfer (e.g., PCET),^25^ such that the active site transiently stores not only one electron, but also one proton that is ultimately derived from bulk water.^3–5,26^ This essential proton is accommodated on the hydroxo ligand of reduced MnSOD and FeSOD.^3,5^ Reduction of Cu/ZnSOD releases the bridging histidine ligand from Cu­(I), which then acts as a proton acceptor.^3,4^ A similar role for the axial His-1 imidazole ligand of NiSOD might be proposed. However, the base is remote from the substrate access channel, on the opposite side of the equatorial ligand plane. Amide linkages within the nickel hook or the Tyr-9 phenolate were suggested as alternative bases.^17^ Protonation of the cysteine thiolate ligands has also been proposed.^23,24,27–31^ The presence of two well-ordered water molecules in the active site pocket may also be relevant.^17,19^

$$M^{n+}+2H^{+}+{O_{2}}^{-}\to M^{(n+1)+}+H_{2}O_{2}$$ 2

3 $$M^{(n+1)+}+{O_{2}}^{-}\to M^{n+}+O_{2}$$

Site-directed mutagenesis studies on NiSOD have confirmed that conserved residues contributing to the active-site ligand field are essential to catalytic activity: the H1A, C2S and C6S mutants were all found to be inactive.^32,33^ The cysteine thiolate ligands are essential to lowering the potential of the Ni­(II/III) couple to the midpoint for superoxide dismutation (ca. 290 mV vs NHE);^34^ indeed, sulfur donor atoms are found generally in redox-active nickel-dependent enzymes.^19,23,34^ Unfortunately, thiolate ligands are susceptible to inactivating oxidation and sulfoxidation reactions.^35,36^ Fine tuning of the ligand field by the axial imidazole base,^24,33^ as well as the mixed amine/amide equatorial ligation,^24,35^ are thought to be necessary to optimize catalysis while protecting the cysteine thiolate coligands. The restricted size of the substrate access channel may function to provide steric protection to these ligands.^17^ Protonation of the cysteine thiolates should also be protective against ROS chemistry.^29^

DFT calculations on simplified models, by Fiedler et al. and by Mullins et al., have been utilized to interpret the electronic structures and spectroscopy of the resting oxidation states.^24,35^ The calculations suggest that the nitrogen ligands protect the thiolate coligands and promote metal-centered redox chemistry through three effects. First, the strong covalency of the equatorial backbone 2-amide donor in the reduced state was proposed to curtail the Ni–S covalency in the redox-active orbital, thus favoring nickel-centered reoxidation.^24,35^ Moreover, hydrogen bonding of the coordinated thiolates is also protective against oxidation.^35^ Fiedler et al. modeled the effects of protonation on the cysteine thiolate ligands, which resulted in a slight decrease in Ni–S­(H) bond length at reduced, square-planar Ni­(II).^24^ Finally, coordination of the axial His-1 base in the oxidized state raises the energy of the Ni 3 $d_{z^{2}}$ SOMO above filled Ni 3d π* orbitals that exhibit significant Ni–S π-covalency, which prevents oxidation of the cysteine thiolate ligands.

DFT calculations were also utilized to model active-site turnover. Pelmenschikov and Siegbahn proposed a complete catalytic cycle with an inner-sphere mechanism for both redox half-reactions.^30^ They invoked a high-spin, square-pyramidal Ni­(II) intermediate, in which the Cys-2 thiolate ligand is transiently protonated. Such an intermediate would facilitate evolution of triplet O_2_ from a six-coordinate adduct, which is necessarily paramagnetic, as well as reoxidation upon substrate coordination. Prabhakar et al. proposed a reduced intermediate with diamagnetic, square-planar Ni­(II) and transient protonation of Cys-6.^31^ Neupane et al. proposed an outer-sphere mechanism in which the axial ligand remains weakly associated with low-spin Ni­(II).^37^ Such an interaction would involve four electrons between the lone pair on the nitrogen donor atom and the filled $d_{z^{2}}$ orbital of Ni­(II); a covalent bond would be obviated, but a weakly attractive electrostatic interaction, akin to an anagostic interaction,^38^ is not excluded. Indeed, the resulting repulsive antibonding overlap destabilizes the 3 $d_{z^{2}}$ orbital above the Ni–S 3d π* orbitals (Scheme 1), so the former functions as the redox-active orbital in both half-reactions, and thiolate oxidation is circumvented.

**Scheme 1:**
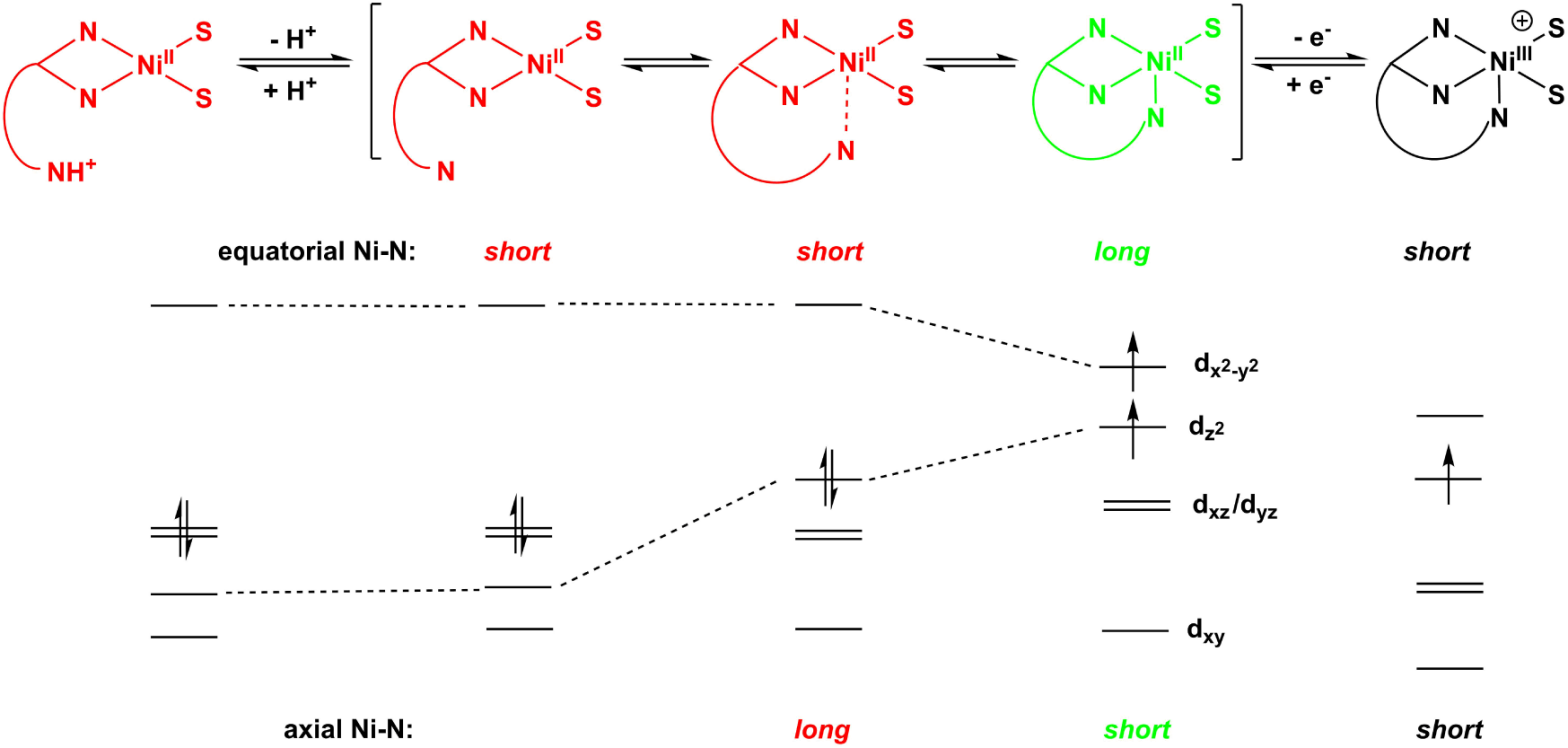
Axial Base Equilibrium of the Scorpionate Ligand and Ligand Field Splittings at Nickel

Diffusion-controlled turnover of NiSOD requires a low activation barrier that limits the degree of structural rearrangement during each redox half-reaction.^30,37^ The calculations of Neupane et al. further indicated that oxidation of a square-pyramidal, low-spin Ni­(II) intermediate obliges only an axial Ni­(III)–N compression, which minimizes the reorganization energy during turnover.^37^ In contrast, a high-spin intermediate that results from strong axial Ni­(II)–N coordination would result in oxidation from the 3 $d_{x^{2}‐y^{2}}$ σ* orbital with a high degree of Ni–S covalency, and the resulting equatorial compression would induce a larger reorganization energy. The proposal of Neupane et al. attributes particular mechanistic importance of the axial His-1 imidazole ligand toward both oxidation states,^37^ and this has motivated our present work.

Model studies have been utilized to mimic the structure and reactivity at the unique active site of NiSOD. Two different approaches have been reviewed: those that use short-chain peptide maquettes to replicate the nickel hook;^39,40^ or synthetic small-molecule analogues that incorporate some features of the nickel hook within a polydentate ligand.^40^ The maquettes reproduce the first six to 13 amino acids of the NiSOD sequence, such that all five ligands of the nickel hook are retained. The peptides generally stabilize the Ni­(II) oxidation state, but oxidation to Ni­(III) and catalytic dismutation activity were demonstrated in some cases, albeit at lower rates and with distinctive mechanistic features.^39^ In one impressive instance, a truncated acetyl-coenzyme A sequence was modified to exhibit SOD activity by an F598H mutation that installed an axial histidine donor proximal to the distal Ni site, which was already ligated equatorially by two cysteine ligands and two backbone amidates.^41^

A plethora of synthetic complexes has been advanced as models of the NiSOD active site.^40^ Most of these replicate aspects of the equatorial ligand field around Ni­(II), but generally are not catalytically active. Some models do support reversible redox couples, and EPR spectra consistent with a Ni­(III) species have been obtained.^42^ While the various synthetic models reproduce the equatorial donors with a varying degree of accuracy,^40^ one feature that is generally lacking is a functional intramolecular axial base equilibrium that replicates the critical function of the His-1 imidazole side chain (Scheme 1). Additions of exogenous aza ligands to Ni­(III) have been documented spectroscopically, but not characterized structurally.^42–45^ Moreover, several recent examples have been reported for an intramolecular axial base equilibrium at Ni­(II) centers with N_2_S_2_ equatorial ligation, but only within bis­(thiolato)-bridged bimetallic complexes.^46–49^ To the best of our knowledge, intramolecular ligation of an axial nitrogen base, in a manner akin to the His-1 side chain, has not been replicated in any monometallic Ni­(II) model complex.

With respect to this last point, two examples warrant additional comment. Nakane et al. synthesized a pentadentate ligand with an N_3_S_2_ donor set composed of terminal thioethers and intervening amide and amine donors, with a 2-pyridylethyl arm on the latter.^50^ A crystal structure of the Ni­(II) complex revealed an elongated square-pyramidal ligand field geometry, in which the axial position was filled by one of the thioether donors. The long Ni···S axial distance of 2.787(3) Å contrasted with the equatorial Ni–S bond length of 2.192(4) Å. A ^1^H NMR spectrum showed broadened resonances over a range of 11–23 ppm that demonstrate some degree of paramagnetism in solution; however, no other evidence was presented for spin crossover coupled to an axial base equilibrium. Truong et al. also reported a pentadentate ligand with a N_3_S_2_ donor set.^51^ A crystal structure of the Zn­(II) complex revealed a square-pyramidal geometry with an axial pyridylmethyl donor and an equatorial N_2_S_2_ donor set that comprised a tertiary amine, amide and *cis*-alkylthiolates. However, an X-ray crystal structure of the Ni­(II) complex anion showed that the pyridylmethyl arm was rotated away from the metal ion and not coordinated. The complex was approximately diamagnetic in solution (μ_eff_ ≤ 0.8 μ_B_).

We previously communicated complexes of the type [(Tp^R^′^,Me^)­NiS_2_CNR_2_], where Tp^R^′^,Me^ = hydrotris­(3-R′,5-methylpyrazol-1-yl)­borate (R′ = Me, Ph and R = Me, Et, Ph) as NiSOD models.^52–54^ The combination of Tp and dithiocarbamate coligands reproduces,^55,56^ to a tractable first approximation, the donor set and ligand field geometry of the NiSOD active site. Of particular interest, the so-called “scorpionate” ligand anions can bind to Ni­(II) in alternative κ^2^- and κ^3^-chelation modes that mimic the variable coordination of the His-1 side chain by a structural isomer and close electronic mimic of imidazole.^57,58^

The pentacoordinate [(κ^3^-Tp^Me,Me^)­NiS_2_CNR_2_] (NR_2_ = NEt_2_ and NPh_2_) complexes were isolated as green crystals.^53^ Analogues with a bulkier, bidentate scorpionate ligand [(κ^2^-Tp^Ph,Me^)­NiS_2_CNR_2_] (NR_2_ = NEt_2_ and NPh_2_) were obtained as red crystals.^52^ Finally, green crystals of square-pyramidal [(κ^3^-Tp^Ph,Me^)­NiS_2_CNMe_2_] exhibit a solid-state spin equilibrium between axially-compressed paramagnetic (*S* = 1) and axially-elongated diamagnetic (*S* = 0) states.^54^ All of these complexes appear green in solution, and exhibit redox couples at potentials relevant to SOD activity. These features enable access to the full range of coordination chemistry within the space occupied by NiSOD.

Prior to our work, Calabrese et al. reported isolation of [(κ^3^-Tp^*i*Pr,Br,H^)­MS_2_CNEt_2_] as green crystals, but did not provide additional details.^59^ Harding et al. subsequently reported several complexes [(κ^3^-Tp^Ph,Ph^)­MS_2_CNR_2_] (M = Co, Ni; NR_2_ = NEt_2_, N^*n*^Bu_2_, NBz_2_, and/or N-*cyclo*-C_4_H_8_).^60,61^ The Ni­(II) complexes were described as green solids, and a crystal structure of the pyrrolidine derivative revealed a pentacoordinate geometry.

We report herein a full investigation of the [(Tp^Ph,Me^)­NiS_2_CNR_2_] complexes, where NR_2_ = NMe_2_ (**1**); NEt_2_ (**2**); NPh_2_ (**3**); NC_12_H_8_, or carbazolate, (**4**), as structural and spectroscopic models for reduced NiSOD. In addition to the X-ray crystal structures reported previously,^52–54^ we obtained structures for pseudopolymorphic crystals of **2**–**4** that establish disparate square-planar, elongated square-pyramidal, and compressed square-pyramidal conformations resulting from κ^2^- versus κ^3^-scorpionate ligation and spin crossover. The complexes exhibit a dynamic axial base equilibrium in solution (Scheme 1). The various conformations and spin states are also supported by shifts in the dithiocarbamate resonance structure (Scheme 2).^56^ There are clear effects of the conformational, axial base and spin equilibria on magnetism and on electronic and vibrational spectra. Reactions induced by separately added protons and KO_2_ were documented, and reversible redox couples were observed at potentials relevant to SOD activity. DFT calculations were carried out on simplified nickel hook and [(Tp)­NiS_2_CNMe_2_] conformational models that further illuminate similarities in geometric and electronic structures, and that facilitate rigorous spectroscopic assignments and comparisons.

**Scheme 2:**
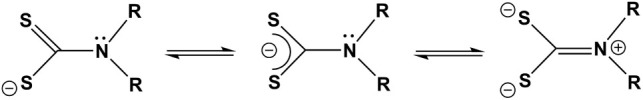
Resonance Structures of the Zwitterionic Dithiocarbamate Ligands

## 2 Experimental Section

### 2.1 Physical Methods

^1^H NMR data were obtained on a Varian Unity spectrometer (500 MHz), processed using the *MestReNova* software suite and referenced internally to solvent.^62^ Solution magnetic moments were determined in CDCl_3_ at 295 K, using the NMR method of Evans.^63^ FT-IR spectra were recorded from KBr pellets on a Thermo-Electron Nicolet 380 spectrophotometer. UV–vis–NIR spectra were obtained on an Agilent HP-8453 diode-array spectrophotometer. Cyclic voltammetry was acquired using a CH Instruments CH1730A workstation; data were acquired from CH_2_Cl_2_ solutions with 0.1 M ^*n*^Bu_4_NPF_6_ supporting electrolyte at a scan rate of 50–100 mV/s at 295 K, using platinum working and counter electrodes with a Ag/AgCl reference electrode. All spectroscopic data were rendered using *SigmaPlot*.^64^ Elemental analyses were carried out by Atlantic Microlabs, Inc. (Norcross, GA).

### 2.2 General Synthetic Procedures

All syntheses were carried out under inert atmosphere, either in an argon-filled glovebox (MBRAUN Unilab), or under purified nitrogen using Schlenk techniques. All glassware was dried in a hot oven and allowed to cool under inert atmosphere. All reagents were purchased from commercial vendors and used without further purification, except for organic solvents that were dried by reflux and distillation over a suitable desiccant under nitrogen.^65^ The precursor complex [(Tp^Ph,Me^)­Ni–Cl] was synthesized as described previously.^66^

#### 2.2.1 Synthesis of Sodium Dithiocarbamate Salts

A solid sample of NaOH (1.0 g, 25 mmol) was dissolved in a minimal amount of water and added to an aqueous solution (40 wt %) of HNMe_2_ (2.82 g, 25 mmol). The solution was cooled in an ice water bath and CS_2_ (15 mL, 25 mmol) was added dropwise. After stirring 0.5 h at 0 °C and 1.0 h at room temperature, the solvents were removed under vacuum to leave a light-orange solid residue. Yield: 2.12 g (15 mmol, 59%). Alternatively, a solid sample of carbazole (1.25 g, 7.5 mmol) was dissolved in dry THF (15 mL) under argon. Equivalent NaH (0.18 g, 7.5 mmol) was added gradually to the solution with stirring. After gas evolution ceased, the solution was cooled to 0 °C and liquid CS_2_ (4.5 mL, 7.5 mmol) was added dropwise with stirring. Hexane was added to precipitate a yellow solid, which was recovered by filtration and dried. Yield: 0.8 g (3.0 mmol, 40%).

#### 2.2.2 Synthesis of [(Tp^Ph,Me^)­NiS_2_CNMe_2_] **(1)**

Solid samples of crystalline pink [(Tp^Ph,Me^)­Ni–Cl] (450 mg, 0.78 mmol) and Na­(S_2_CNMe_2_) (168 mg, 1.17 mmol) were dissolved in CH_2_Cl_2_ (20 mL) with stirring. The resulting green solution was evaporated to dryness. The solids were extracted with toluene, and the extracts were filtered and dried under vacuum. The residue was extracted into CH_3_CN, and slow evaporation of the resulting solution at room temperature yielded green crystals. Yield: 294 mg (0.44 mmol, 57%). Anal. Calcd (found) for C_33_H_34_BN_7_NiS_2_: C, 59.84 (59.91); H, 5.17 (5.24); N, 14.80 (14.78); S, 9.68 (10.09). ^1^H NMR (CDCl_3_, 298 K; δ, ppm): 53.5 (3H, 4-H); 43.3 (6H, N–C*H*_3_); 7.3 (4H, 3-*para*); 6.8 (6H, 3-*meta*); 4.6 (6H, 3-*ortho*); 1.8 (9H, 5-CH_3_); −8.4 (1H, B–H). μ_eff_ = 2.77 μ_B_. UV–vis (CH_2_Cl_2_, λ_max_, nm; ε, mM^–1^ cm^–1^): 235 (48); 298 (sh, 8.6); 356 (sh, 1.5); 425 (0.8); 650 (0.1). FTIR (KBr, cm^–1^): 2529, ν­(B–H), major; 2474, ν­(B–H), minor; 1392, ν­(C=N); 1257, ν_as_(NMe_2_); 985, ν_as_(CS_2_).

#### 2.2.3 Synthesis of [(Tp^Ph,Me^)­NiS_2_CNEt_2_] **(2)**

The synthesis was carried out by the same method as for **1** above, using solid samples of [(Tp^Ph,Me^)­Ni–Cl] (100 mg, 0.17 mmol) and Na­(S_2_CNEt_2_) (45 mg, 0.26 mmol). Red crystals were obtained by slow evaporation of a CH_3_CN solution. Yield: 64 mg, 55%. Alternatively, red crystals of a CH_2_Cl_2_ monosolvate were obtained from slow diffusion of *n*-hexane into a CH_2_Cl_2_ solution. Anal. Calcd (found) for C_35_H_38_BN_7_NiS_2_: C, 60.89 (60.84); H, 5.55 (5.55); N, 14.20 (14.26). ^1^H NMR (CDCl_3_, 295 K; δ, ppm): 52.7 (3H, 4-H); 34.0 (4H, *N–*C*H*_2_); 7.1 (3H, 3-Ph, *para*); 6.8 (6H, 3-Ph, *meta*); 4.7 (6H, 3-Ph, *ortho*); 1.6 (9H, 5-Me); 1.1 (6H, *N–*CH_2_C*H*_3_); −8.5 (1H, B–H). μ_eff_ = 2.52 μ_B._ UV–vis (CH_2_Cl_2_, λ_max_, nm; ε, mM^–1^ cm^–1^): 238 (60.3); 318 (16.9); 388 (sh, 2.8); 422 (1.4); 650 (0.1). FTIR (KBr, cm^–1^): 2523, ν­(B–H), minor; 2478, ν­(B–H), major; 1456, ν­(C=N); 1277, ν_as_(NEt_2_); 1003, ν_as_(CS_2_).

#### 2.2.4 Synthesis of [(Tp^Ph,Me^)­NiS_2_CNPh_2_] **(3)**

The synthesis was carried out by the same method as for **1** above, using solid samples of [(Tp^Ph,Me^)­Ni–Cl] (100 mg, 0.17 mmol) and Na­(S_2_CNPh_2_) (70 mg, 0.26 mmol). Green crystals were obtained by diffusion of *n*-hexane into a CH_2_Cl_2_ solution at −33 °C. Red crystals of a monosolvate were obtained alternatively by slow evaporation of a CH_3_CN solution. Yield: 104 mg, 78%. Anal. Calcd (found) for C_43_H_38_BN_7_NiS_2_: C, 65.67 (65.73); H, 4.87 (4.81); N, 12.47 (12.49). ^1^H NMR (CDCl_3_, 295 K; δ, ppm): 54.2 (3H, 4-H); 8.4 (4H, *N*–Ph, *ortho*); 7.7 (4H, *N*–Ph, *meta*); 7.1 (3H, 3-Ph, *para*); 6.8 (6H, 3-Ph, *meta*); 4.4 (6H, 3-Ph, *ortho*); 4.1 (2H, *N*–Ph, *para*); 1.6 (9H, 5-Me); −8.5 (1H, B–H). μ_eff_ = 2.49 μ_B._ UV–vis (CH_2_Cl_2_, λ_max_, nm; ε, mM^–1^ cm^–1^): 238 (55.8); 293 (17.3); 429 (1.6); 670 (0.1). FTIR (KBr, cm^–1^): 2548, ν­(B–H), green; 2477, ν­(B–H), red; 1352, ν­(C=N), green; 1407, ν­(C=N), red.

#### 2.2.5 Synthesis of [(Tp^Ph,Me^)­NiS_2_CNC_12_H_8_] **(4)**

The synthesis was carried out by the same method as for **1** above, using solid samples of [(Tp^Ph,Me^)­Ni–Cl] (50 mg, 0.09 mmol) and Na­(S_2_CNC_12_H_8_) (23 mg, 0.09 mmol), both dissolved in methanol (30 mL). Green crystals were obtained by slow diffusion of *n*-hexane into a dichloromethane solution at room temperature. Yield: 54 mg (0.07 mmol, 80%). Red crystals of a solvated pseudopolymorph were obtained from dichloromethane/hexane at −40 °C; these turned green irreversibly upon warming toward room temperature. Anal. Calcd (found) for C_43_H_36_BN_7_NiS_2_: C, 65.84 (66.34); H, 4.63 (4.51); N, 12.50 (12.49). ^1^H NMR (CDCl_3_, 295 K; δ, ppm): 60.4 (3H, 4-H); 9.5 (2H, carbazole); 9.2 (2H, carbazole); 8.8 (2H, carbazole); 8.6 (2H, carbazole); 6.8 (6H, 3-Ph, *para*); 6.6 (6H, 3-Ph, *meta*); 4.3 (3H, 3-Ph, *ortho*); 2.8 (9H, 5-Me); −8.0 (1H, B–H). μ_eff_ = 2.55 μ_B._ UV–vis (CH_2_Cl_2_, λ_max_, nm; ε, mM^–1^ cm^–1^): 239 (48.4); 285 (18.1, sh); 330 (15.8); 355 (17.2); 432 (2.7); 658 (0.1). FTIR (KBr, cm^–1^; green crystals): 2546, ν­(B–H); 1295, ν­(C=N); 1164, ν_as_(NR_2_); 1045, ν_as_(CS_2_).

#### 2.2.6 Syntheses of [(HB­{pz^Ph,Me^}_2_{OC­(O)­CF_3_})­NiS_2_CNPh_2_] **(5)** and [(Tp^Ph,Me^)­Ni­(Hpz^Ph,Me^)­(OC­{O}­CF_3_)] **(6)**

A solid sample of complex **3** (50 mg, 0.06 mmol) was dissolved in dichloromethane to give a green solution. To the stirred solution at room temperature was added CF_3_CO_2_H (5.0 μL, 1.0 equiv). The color of the solution changed immediately from green to red. The solution was layered with *n*-hexane and allowed to stand ca. 2 weeks at 0 °C. A mixture of red crystals of **5** and green crystals of **6** was obtained, in a combined yield of 36 mg, and these were separated manually and characterized separately. Anal. Calcd (found) for **5,** C_35_H_29_BF_3_N_5_NiO_2_S_2_: C, 56.63 (56.18); H, 3.94 (4.17); N, 9.43 (10.42). ^1^H NMR (CDCl_3_, 295 K; δ, ppm): ca. 8 (ca. 10H, v br.); 7.4 and 7.3 (10H); 6.9 (2H, 4-H); 2.3 (6H, 5-Me). UV–vis (CH_2_Cl_2_, λ_max_, nm; ε, mM^–1^ cm^–1^): 240 (25.3); 296 (9.2, sh); 370 (1.7, sh); 424 (0.5, sh). FTIR (KBr, cm^–1^): 2511, ν­(B–H); 1676, ν­(C=O); 1410, ν­(C=N); 1197, ν­(CF_3_). Anal. Calcd (found) for **6**, C_42_H_38_BF_3_N_8_NiO_2_: C, 62.02 (61.62); H, 4.71 (4.61); N, 13.78 (13.77). ^1^H NMR (CDCl_3_, 295 K; δ, ppm): 66.5 (1H, 4-H); 63.1 (2H, 4-H); 47.3 (1H, 4-pz-H); 11.0–5.4; 2.6 (9H, 5-Me?); 1.0 (8H, ?); −9.7 (1H, B–H); −10.2 (3H, 3-Me-pz-H). UV–vis (CH_2_Cl_2_, λ_max_, nm; ε, mM^–1^ cm^–1^): 240 (54.1); 323 (1.4); 382 (0.2); 430 (0.2); 642 (0.05); 925 (0.3). FTIR (KBr, cm^–1^): 2546, ν­(B–H); 1666, ν­(C=O); 1197, ν­(CF_3_).

#### 2.2.7 Isolation of [(μ-pz^Ph,Me^)­NiS_2_CNMe_2_]_2_ **(7)**

Solid samples of **1** (50 mg, 0.08 mmol) and KO_2_ (6.0 mg, 0.08 mmol) were dissolved in MeCN (2 mL), and 20 mg 18-crown-6 (0.08 mmol) was added to the stirred solution. The color of the solution changed slowly from green to red to orange. After stirring for 3 h, a pink-red precipitate separated. The solids were recovered by filtration and dissolved in a minimal quantity of dichloromethane. The solution was layered with diethyl ether. A small quantity of red crystals was recovered after standing for several days at room temperature. ^1^H NMR (CD_2_Cl_2_, 295 K; δ, ppm): 8.89 (4H, 3-Ph, *ortho*); 7.58 (4H, 3-Ph, *meta*); 7.37 (2H, 3-Ph, *para*); 5.95 (2H, 4-pz); 3.18 (6H, NMe); 3.05 (6H, NMe); 2.74 (6H, 5-Me).

### 2.3 DFT Calculations

The Amsterdam Density Functional software package was used for all calculations.^67,68^ Calculations were carried out on simplified nickel hook structures (Schemes S1 and S2, Tables S1–S16 and Figures S1–S12), the Me_2_NCS_2_^–^ free ligand (Tables S17–S20 and Figures S13–S15), and various conformations of [(L)­NiS_2_CNMe_2_] (L = Tp, Bp) and [(L)­NiS_2_CNMe_2_]^+^ (Tables S21–S54 and Figures S16–S35). Full details are given in Supporting Information.

### 2.4 X-ray Crystallography

Complexes **1**–**7** were structurally characterized by X-ray diffraction. Full details are given in Supporting Information. The data collections and refinements are summarized in Table 1. Thermal ellipsoid plots, rendered using *Mercury*,^69^ are shown in Figures 2–8, and Figures S36 and S37. Relevant bond lengths and angles are given in the figure captions and compared in Tables 2 and 3.

**Figure 2:**
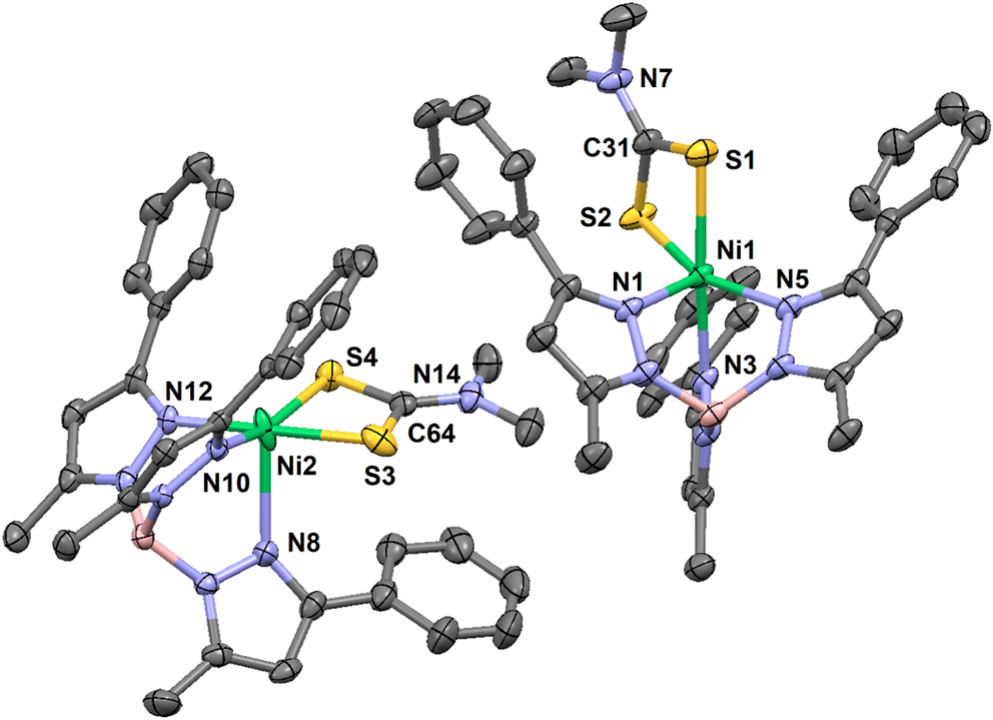
Thermal ellipsoid plot for the two independent molecules of [(Tp^Ph,Me^)­NiS_2_CNMe_2_] (**1**) at 293 K (30% probability). Hydrogen atoms are omitted for clarity. Select bond lengths (Å) for **1**: Ni1–N1, 2.048(1); Ni1–N3, 2.111(1); Ni1–N5, 2.058(1); Ni1–S1, 2.3929(5); Ni1–S2, 2.3435(5); C31–S1, 1.708(2); C31–S2, 1.711(2); C31–N7, 1.329(2); Ni2–N8, 2.149(1); Ni2–N10, 2.046(1); Ni2–N12, 2.083(1); Ni2–S3, 2.3614(5); Ni2–S4, 2.3234(6); C64–S3, 1.702(2); C64–S4, 1.722(2); C64–N14, 1.327(2). Select bond angles (°) for **1**: N1–Ni1–N3, 90.70(5); N1–Ni1–N5, 95.50(6); N3–Ni1–N5, 84.31(5); N1–Ni1–S1, 96.84(4); N1–Ni1–S2, 108.47(4); N3–Ni1–S1, 172.10(4); N3–Ni1–S2, 100.22(4); N5–Ni1–S1, 97.26(4); N5–Ni1–S2, 155.45(5); S1–Ni1–S2, 75.18(2); S1–C31–S2, 115.40(9); N7–C31–S1, 122.6(1); N7–C31–S2, 122.0(2); N8–Ni2–N10, 94.56(6); N8–Ni2–N12, 91.78(5); N10–Ni2–N12, 85.66(6); N8–Ni2–S3, 93.85(4); N8–Ni2–S4, 111.25(5); N10–Ni2–S3, 98.18(4); N10–Ni2–S4, 153.73(4); N12–Ni2–S3, 172.91(4); N12–Ni2–S4, 97.91(4); S3–Ni2–S4, 76.02(2); S3–C64–S4, 114.9(1); N14–C64–S3, 122.8­(2); N14–C64–S4, 122.4(2). Select torsions (°) for **1**: H–B1–N2–N1, 179.6; H–B2–N9–N8, 167.2.

**Figure 3:**
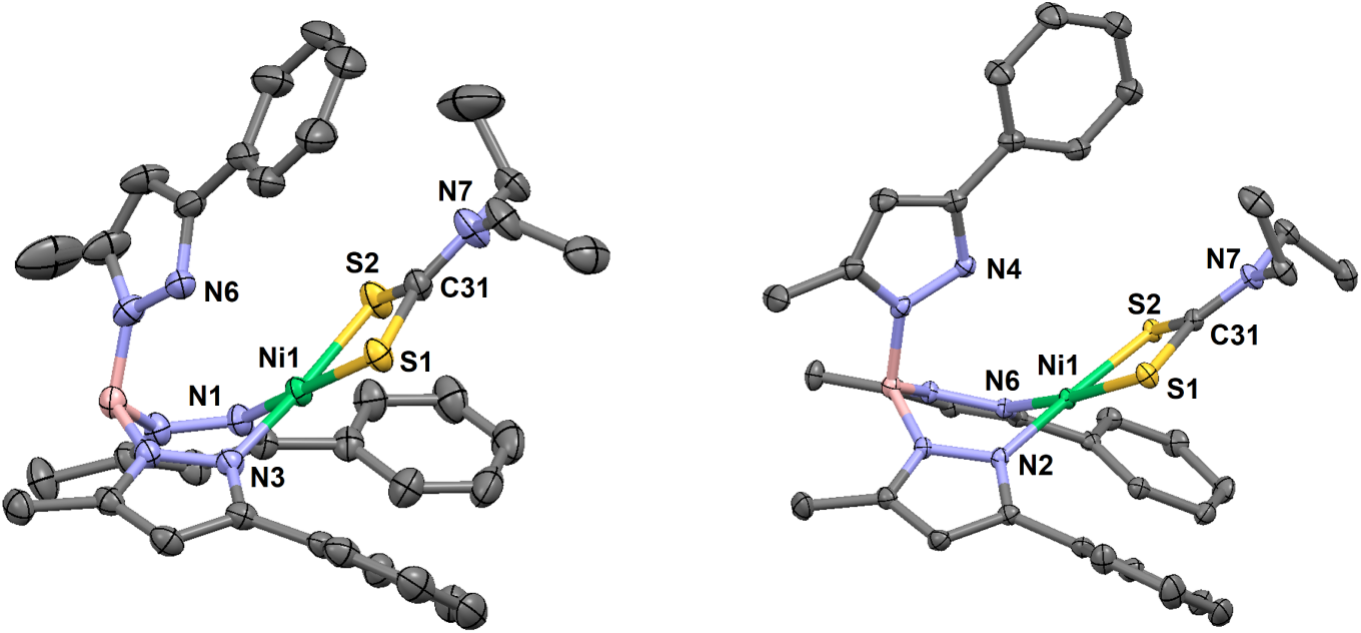
Thermal ellipsoid plots (30% probability) for [(Tp^Ph,Me^)­NiS_2_CNEt_2_] (**2**) at 293 K (left, only one orientation is shown of the disordered *N*-ethyl substituent), and a pseudopolymorphic CH_2_Cl_2_ solvate at 173 K (right, disordered solvent molecule not shown). Hydrogen atoms are omitted for clarity. Select bond lengths (Å) for **2**: Ni1–N1, 1.929(1); Ni1–N3, 1.934(1); Ni1···N6, 2.805(1); Ni1–S1, 2.1990(5); Ni1–S2, 2.1929(5); C31–S1, 1.706(2); C31–S2, 1.710(2); C31–N7, 1.318(2). Select bond lengths (Å) for **2**·CH_2_Cl_2_: Ni1–N2, 1.920(1); Ni1–N6, 1.915(1); Ni1···N4, 3.001(2); Ni1–S1, 2.2038(5); Ni1–S2, 2.1969(5); C31–S1, 1.714(2); C31–S2, 1.714(2); C31–N7, 1.313(2). Select bond angles (°) for **2**: N1–Ni1–N3, 90.32(6); N1–Ni1–S1, 172.86(4); N1–Ni1–S2, 95.73(4); N3–Ni1–S1, 96.16(4); N3–Ni1–S2, 172.46(5); S1–Ni1–S2, 78.06(2); S1–C31–S2, 108.1(1); N7–C31–S1, 126.3(2); N7–C31–S2, 125.6(2). Select bond angles (°) for **2**·CH_2_Cl_2_: N2–Ni1–N6, 90.81(6); N2–Ni1–S1, 96.27(4); N2–Ni1–S2, 174.67(4); N6–Ni1–S1, 172.61(4); N6–Ni1–S2, 94.49(4); N4···Ni1–N2, 88.50(5); N4···Ni1–N6, 79.46(5); N4···Ni1–S1, 98.56(3); N4···Ni1–S2, 92.98(3); S1–Ni1–S2, 78.46(2); S1–C31–S2, 108.56(9); N7–C31–S1, 126.5(1); N7–C31–S2, 125.0(1). Select torsion (°) for **2**: H–B1–N5–N6, 133.2. Select torsion (°) for **2**·CH_2_Cl_2_: H–B1–N3–N4, 153.6.

**Figure 4:**
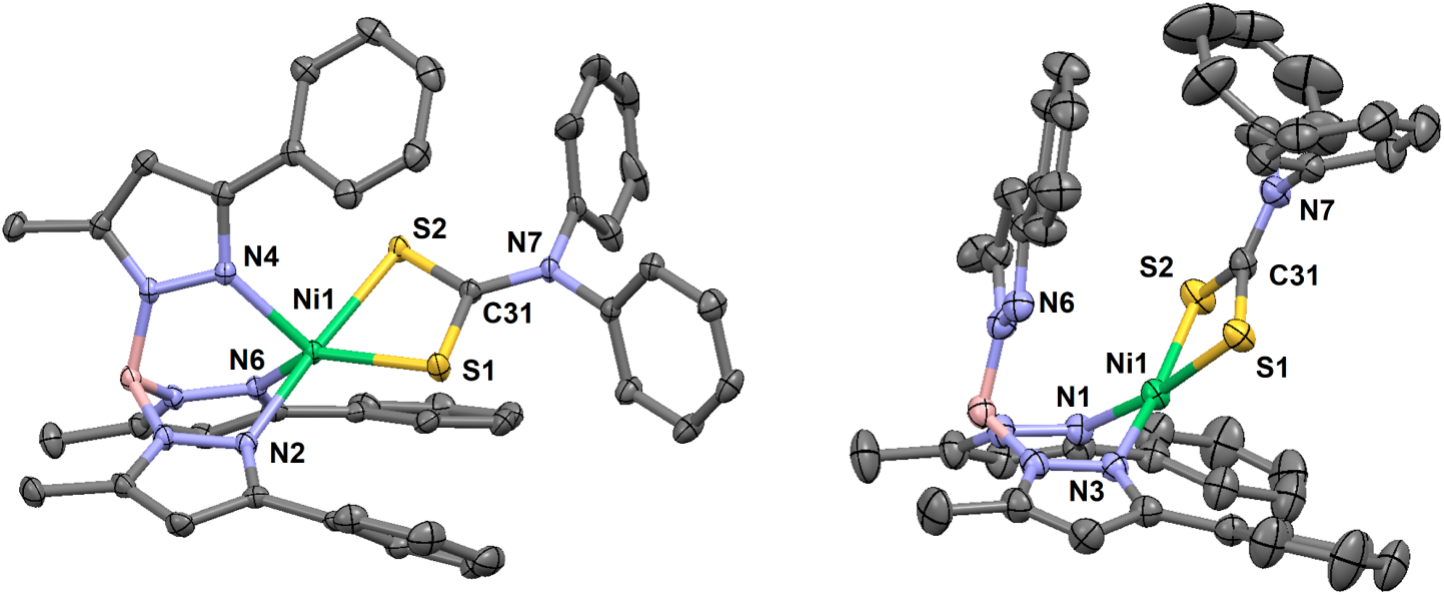
Thermal ellipsoid plots (30% probability) for [(Tp^Ph,Me^)­NiS_2_CNPh_2_] (**3**) at 173 K (left), and a pseudopolymorphic MeCN hemisolvate at 293 K (right, disordered solvent molecule not shown). Hydrogen atoms are omitted for clarity. Select bond lengths (Å) for **3**: Ni1–N2, 2.107(3); Ni1–N4, 2.040(2); Ni1–N6, 2.079(2); Ni1–S1, 2.3415(8); Ni1–S2, 2.4210(8); C31–S1, 1.720(3); C31–S2, 1.691(3); C31–N7, 1.351(4). Select bond lengths (Å) for **3**·0.5MeCN: Ni1–N1, 1.910(2); Ni1–N3, 1.911(1); Ni1···N6, 3.541(2); Ni1–S1, 2.2013(5); Ni1–S2, 2.1832(5); C31–S1, 1.701(2); C31–S2, 1.704(2); C31–N7, 1.333(3). Select bond angles (Å) for **3**: N2–Ni1–N4, 87.7(1); N2–Ni1–N6, 87.0(1); N4–Ni1–N6, 96.3(1); N2–Ni1–S1, 100.21(7); N2–Ni1–S2, 175.32(7); N4–Ni1–S1, 124.11(7); N4–Ni1–S2, 94.69(7); N6–Ni1–S1, 138.94(7); N6–Ni1–S2, 96.77(7); S1–Ni1–S2, 75.12(3); S1–C31–S2, 116.7(2); S1–C31–N7, 120.6(2); S2–C31–N7, 122.7(2). Select bond angles (°) for **3**·0.5MeCN: N1–Ni1–N3, 90.34(6); N1–Ni1–S1, 171.34(5); N1–Ni1–S2, 94.38(5); N3–Ni1–S1, 95.64(5); N3–Ni1–S2, 170.65(5); S1–Ni1–S2, 78.84(2); S1–C31–S2, 109.7(1); S1–C31–N7, 126.0(2); S2–C31–N7, 124.3(1). Select torsion (°) for **3**: H–B1–N3–N4, 166.2. Select torsion (°) for **3**·0.5MeCN: H–B1–N5–N6, 83.5.

**Figure 5:**
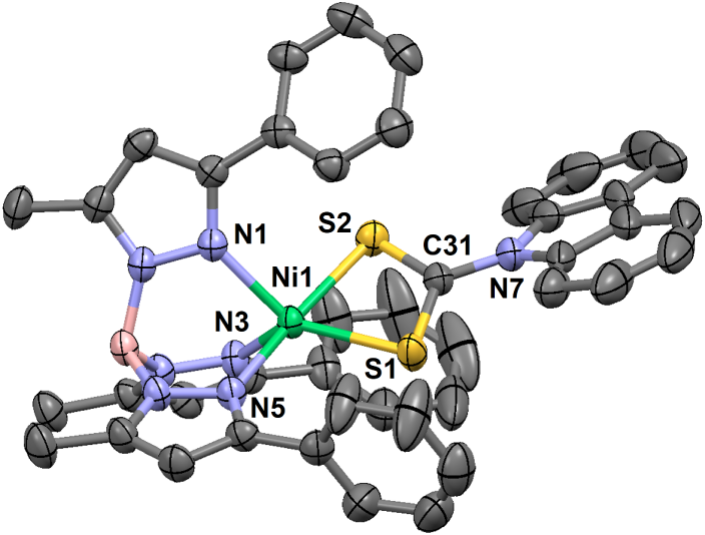
Thermal ellipsoid plots (30% probability) for green crystals of [(Tp^Ph,Me^)­NiS_2_CNC_12_H_8_] (**4**) at 293 K. Hydrogen atoms are omitted for clarity. Select bond lengths (Å) for **4**: Ni1–N1, 2.042(2); Ni1–N3, 2.035(2); Ni1–N5, 2.078(2); Ni1–S1, 2.350(1); Ni1–S2, 2.425(1); C31–N7, 1.384(4); C31–S1, 1.697(3); C31–S2, 1.669(3). Select bond angles (°) for **4**: N1–Ni1–N3, 92.42(9); N1–Ni1–N5, 86.72(9); N3–Ni1–N5, 90.65(9); N1–Ni1–S1, 138.78(7); N1–Ni1–S2, 95.25(6); N3–Ni1–S1, 127.72(7); N3–Ni1–S2, 96.54(7); N5–Ni1–S1, 100.14(6); N5–Ni1–S2, 172.45(6); S1–Ni1–S2, 73.60(3); S1–C31–S2, 116.4(2); S1–C31–N7, 121.3(2); S2–C31–N7, 122.3(2). Select torsion (°) for **4**: H–B1–N2–N1, 169.4.

**Figure 6:**
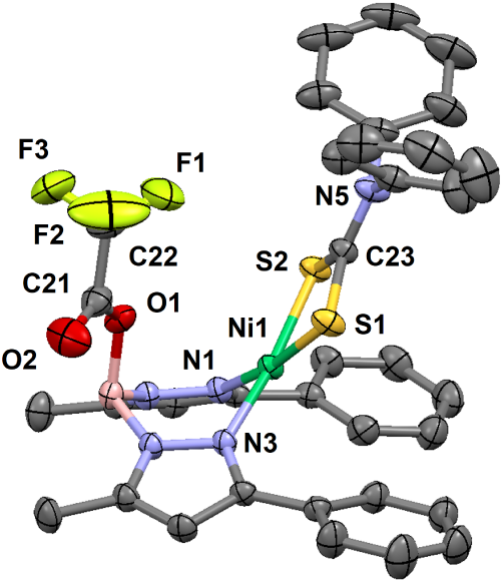
Thermal ellipsoid plot (30% probability) for [(HB­{pz^Ph,Me^}_2_{OC­(O)­CF_3_})­NiS_2_CNPh_2_] (**5**) at 293 K. Hydrogen atoms are omitted for clarity. Only one orientation of the rotationally disordered −CF_3_ moiety is shown. Select bond lengths (Å) for **5**: Ni1–N1, 1.908(2); Ni1–N3, 1.916(2); Ni1–S1, 2.2040(6); Ni1–S2, 2.1916(6); C23–S1, 1.705(2); C23–S2, 1.706(2); C23–N5, 1.331(3); B1–O1, 1.510(3); C21–O1, 1.301(3); C21–O2, 1.196(4); C21–C22, 1.513(5); C22–F1, 1.288(7); C22–F2, 1.304(5); C22–F3, 1.301(6). Select bond angles (°) for **5**: N1–Ni1–N3, 90.53(7); N1–Ni1–S1, 171.03(6); N1–Ni1–S2, 93.41(5); N3–Ni1–S1, 96.66(5); N3–Ni1–S2, 170.98(5); S1–Ni1–S2, 78.73(2); S1–C23–S2, 109.7(1); N5–C23–S1, 125.5(2); N5–C23–S2, 124.9(2); O1–C21–O2, 128.4(3); O1–C21–C22, 112.2(3); O2–C21–C22, 119.5(3). Select torsion (°) for **5**: H–B1–O1–C21, 61.5.

**Figure 7:**
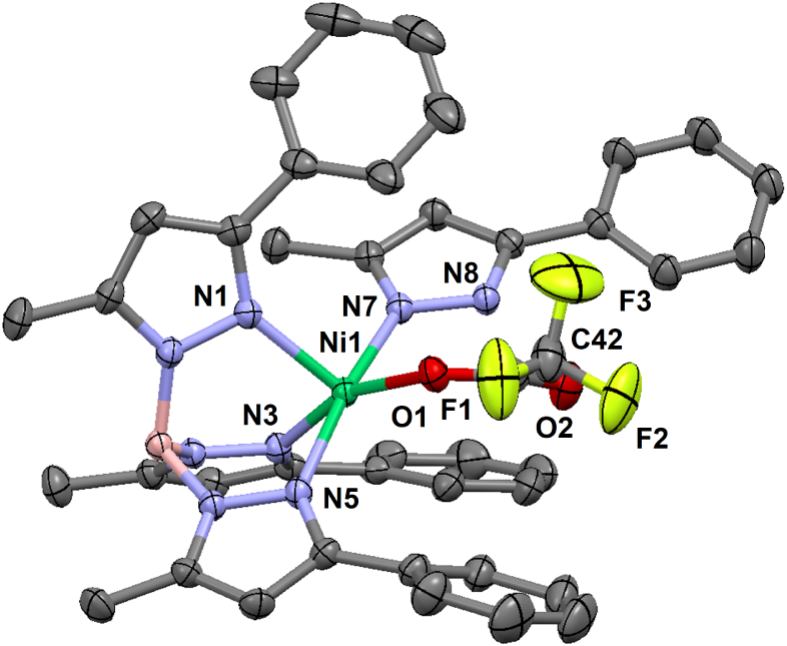
Thermal ellipsoid plot (30% probability) for [(Tp^Ph,Me^)­Ni­(Hpz^Ph,Me^)­(OC­{O}­CF_3_)] (**6**) at 293 K. Hydrogen atoms are omitted for clarity. Only one orientation of the rotationally disordered −CF_3_ moiety is shown. Select bond lengths (Å) for **6**: Ni1–N1, 2.037(2); Ni1–N3, 2.071(2); Ni1–N5, 2.093(2); Ni1–N7, 2.055(2); Ni1–O1, 2.047(2); Ni1···O2, 3.543(2); C41–O1, 1.254(3); C41–O2, 1.222(3); C41–C42, 1.547(4); C42–F1, 1.292(4); C42–F2, 1.306(4); C42–F3, 1.302(5); N8···O2, 2.756(3). Select bond angles (°) for **6**: N1–Ni1–N3, 94.31(7); N1–Ni1–N5, 92.98(7); N1–Ni1–N7, 95.41(7); N1–Ni1–O1, 103.69(7); N3–Ni1–N5, 85.25(6); N3–Ni1–N7, 90.39(7); N3–Ni1–O1, 160.67(7); N5–Ni1–N7, 170.81(6); N5–Ni1–O1, 86.78(6); N7–Ni1–O1, 94.84(6); O1–C41–O2, 130.0(2); O1–C41–C42, 114.1(2); O2–C41–C42, 115.9(2). Select torsion (°) for **6**: H–B1–N2–N1, 174.2.

**Figure 8:**
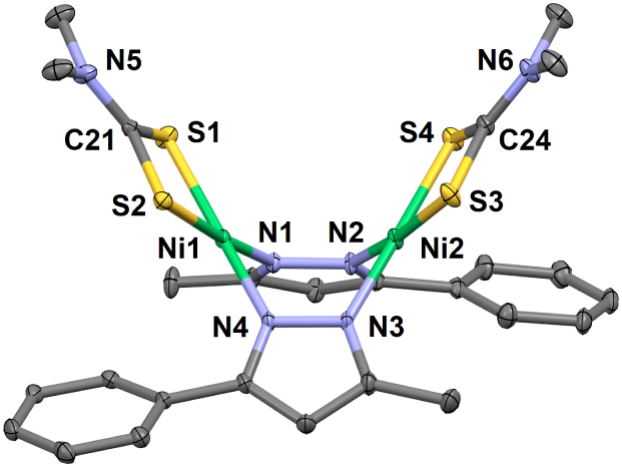
Thermal ellipsoid plot (30% probability) for [(μ-pz^Ph,Me^)­NiS_2_CNMe_2_]_2_ (**7**) at 123 K. Hydrogen atoms are omitted for clarity. Select bond lengths (Å) for **7**: Ni1–N1, 1.904(3); Ni1–N4, 1.898(3); Ni1–S1, 2.200(1); Ni1–S2, 2.199(1); C21–S1, 1.715(3); C21–S2, 1.713(3); C21–N5, 1.318(4); Ni2–N2, 1.887(3); Ni2–N3, 1.898(3); Ni2–S3, 2.204(1); Ni2–S4, 2.200(1); C24–S3, 1.723(3); C24–S4, 1.714(3); C24–N6, 1.311(4); Ni1···Ni2, 3.0786(9). Select bond angles (°) for **7**: N1–Ni1–N4, 90.5(1); N1–Ni1–S1, 96.22(8); N1–Ni1–S2, 174.88(9); N4–Ni1–S1, 172.89(9); N4–Ni1–S2, 94.23(9); S1–Ni1–S2, 79.00(4); S1–C21–S2, 109.4(2); S1–C21–N5, 124.7(2); S2–C21–N5, 125.8(3); N2–Ni2–N3, 90.1(1); N2–Ni2–S3, 172.54(9); N2–Ni2–S4, 93.96(9); N3–Ni2–S3, 96.93(9); N3–Ni2–S4, 175.53(9); S3–Ni2–S4, 78.92(4); S3–C24–S4, 109.0(2); S3–C24–N6, 125.2(3); S4–C24–N6, 125.8(3).

**Table 1:** Summary of X-ray Crystallography

Complex	**1**	**1**	**2**	**2**	**3**
Empirical formula	C_33_H_34_BN_7_NiS_2_	C_33_H_34_BN_7_NiS_2_	C_35_H_38_BN_7_NiS_2_	C_35_H_38_BN_7_NiS_2_·CH_2_Cl_2_	C_43_H_38_BN_7_NiS_2_·0.5MeCN
Formula weight (g mol^–1^)	662.31	662.31	690.36	775.29	806.97
Crystal color, morphology	green	green block	red-orange, irregular	red block	orange block
Temperature (K)	293(2)	123(2)	293(2)	173(2)	293(2)
Crystal system	triclinic	triclinic	monoclinic	monoclinic	monoclinic
Space group	*P*1̅ (No. 2)	*P*1̅ (No. 2)	*P*2_1_/*c* (No. 14)	*P*2_1_/*c* (No. 14)	*C*2/*c* (No. 15)
*a* (Å)	12.1442(8)	12.004(1)	11.9864(6)	13.592(1)	37.626(2)
*b* (Å)	12.6611(9)	12.536(1)	22.936(1)	17.900(2)	9.7449(6)
*c* (Å)	25.058(2)	24.678(2)	13.8614(7)	15.778(2)	22.628(2)
α (deg)	90.556(1)	91.034(1)	90	90	90
β (deg)	102.357(1)	101.353(1)	111.214(1)	96.854(2)	92.636(1)
γ (deg)	116.994(1)	116.788(1)	90	90	90
*V* (Å^3^)	3328.3(4)	3225.9(5)	3552.6(3)	3811.3(7)	8288.0(9)
*Z*	4	4	4	4	8
*D*_calcd._ (g cm^–3^)	1.322	1.364	1.291	1.351	1.293
Absorption coefficient (mm^–1^)	0.743	0.766	0.699	0.651	0.610
Crystal size (mm)	0.58 × 0.40 × 0.20	0.40 × 0.38 × 0.30	0.50 × 0.38 × 0.28	0.45 × 0.25 × 0.15	0.54 × 0.34 × 0.20
Reflections collected	23405	38246	23868	44717	27803
Independent reflections (*R*_int_)	14697 (0.0325)	14519 (0.0282)	8088 (0.0355)	8730 (0.0417)	9251 (0.0363)
Observed reflections	11612	11726	6788	6787	7111
Data/restraints/parameters	14697/1/849	14519/0/803	8088/4/440	8730/0/420	9251/14/561
GoF	1.029	1.040	1.038	1.048	1.047
*R*1, *wR*2 (*I* > 2σ{*I*})	0.0500/0.1373	0.0373/0.0849	0.0379/0.1036	0.0314/0.0694	0.0372/0.0940
*R*1, *wR*2 (all data)	0.0632/0.1496	0.0525/0.0934	0.0466/0.1111	0.0504/0.0784	0.0553/0.1096
Difference peak, hole (e Å^–3^)	1.344/–1.030	0.559/–1.498	0.342/–0.197	0.315/–0.239	0.404/–0.197

Complex	**3**	**4**	**5**	**6**	**7**
Empirical formula	C_43_H_38_BN_7_NiS_2_	C_43_H_36_BN_7_NiS_2_	C_35_H_29_BF_3_N_5_NiO_2_S_2_	C_42_H_38_BF_3_N_8_NiO_2_	C_26_H_30_N_6_Ni_2_S_4_
Formula weight (g mol^–1^)	786.44	784.43	742.27	813.32	672.22
Crystal color, morphology	green block	green, irregular	red fragment	green block	red plate
Temperature (K)	173(2)	293(2)	293(2) K	293(2) K	123(2)
Crystal system	monoclinic	triclinic	triclinic	triclinic	monoclinic
Space group	*P*2_1_ (No. 4)	*P*1̅ (No. 2)	*P*1̅ (No. 2)	*P*1̅ (No. 2)	*P*2_1_/*c* (No. 14)
*a* (Å)	10.866(1)	10.7628(7)	11.1443(8)	12.091(1)	10.161(3)
*b* (Å)	13.916(2)	13.1002(9)	12.4491(9)	12.525(1)	17.993(6)
*c* (Å)	13.928(2)	16.001(1)	13.6203(9)	15.520(1)	19.078(6)
α (deg)	90	69.748(1)	87.510(1)	104.659(2)	90
β (deg)	110.920(2)	78.548(1)	84.068(1)	94.539(2)	95.967(5)
γ (deg)	90	66.311(1)	70.055(1)	118.055(2)	90
*V* (Å^3^)	1967.2(4)	1933.6(2)	1766.7(2)	1951.8(3)	3469(2)
*Z*	2	2	2	2	4
*D*_calcd._ (g cm^–3^)	1.328	1.347	1.395	1.384	1.287
Absorption coefficient (mm^–1^)	0.640	0.651	0.722	0.559	1.349
Crystal size (mm)	0.35 × 0.18 × 0.15	0.48 × 0.36 × 0.22	0.40 × 0.24 × 0.12	0.48 × 0.42 × 0.30	0.25 × 0.12 × 0.03
Reflections collected	23446	13320	12493	13950	39874
Independent reflections (*R*_int_)	8921 (0.0352)	8483 (0.0398)	7832 (0.0364)	8720 (0.0268)	7937 (0.0671)
Observed reflections	7903	6611	6326	7345	5333
Data/restraints/parameters	8921/1/490	8483/0/490	7832/6/472	8720/0/531	7937/0/349
GoF	1.015	1.046	1.026	1.057	1.071
*R*1, *wR*2 (*I* > 2σ{*I*})	0.0304/0.0653	0.0619/0.1667	0.0438/0.1119	0.0468/0.1246	0.0408/0.0780
*R*1, *wR*2 (all data)	0.0392/0.0697	0.0765/0.1829	0.0568/0.1216	0.0566/0.1349	0.0834/0.0921
Difference peak, hole (e Å^–3^)	0.223/–0.239	0.451/–0.453	0.365/–0.215	0.837/–0.427	0.584/–0.469

**Table 2:** Comparative Coordinate and Dithiocarbamate Bond Lengths (Å)^a^

Complex	τ^5^ ^b^	Ni–N_ax/eq_	Ni–N_eq/ax_	Ni–N_eq/eq_	Ni–S_eq/ax_	Ni–S_eq/eq_	C–S_eq/ax_	C–S_eq/eq_	C=NR_2_
**1–Ni1** ^c^	0.29	2.038(2)	2.111(2)	2.048(2)	2.4006(6)	2.3420(6)	1.712(2)	1.723(2)	1.327(3)
**1–Ni2** ^c^	0.22	2.401(2)	2.003(2)	1.972(2)	2.2721(7)	2.2567(6)	1.705(2)	1.720(2)	1.320(3)
**1–Ni1** ^d^	0.28	2.048(1)	2.111(1)	2.058(1)	2.3929(5)	2.3435(5)	1.708(2)	1.711(2)	1.329(2)
**1–Ni2** ^d^	0.32	2.149(1)	2.083(1)	2.046(1)	2.3614(5)	2.3234(6)	1.702(2)	1.722(2)	1.327(2)
**2** ^e^	0.01	2.805(1)	1.929(1)	1.934(1)	2.1990(5)	2.1929(5)	1.706(2)	1.710(2)	1.318(2)
**2** ^f^	0.03	3.001(2)	1.920(1)	1.915(1)	2.1969(5)	2.2038(5)	1.714(2)	1.714(2)	1.313(2)
**3** ^g^	0.61	2.040(2)	2.107(3)	2.079(2)	2.4210(8)	2.3415(8)	1.691(3)	1.720(3)	1.351(4)
**3** ^h^	0.01	3.541(2)	1.910(2)	1.911(1)	2.2013(5)	2.1832(5)	1.701(2)	1.704(2)	1.333(3)
**4** ^i^	0.56	2.042(2)	2.078(2)	2.035(2)	2.425(1)	2.350(1)	1.669(3)	1.697(3)	1.384(4)
**5** ^j^	0.00		1.908(2)	1.916(2)	2.2040(6)	2.1916(6)	1.705(2)	1.706(2)	1.331(3)
**7–Ni1** ^k^	0.03		1.904(3)	1.898(3)	2.199(1)	2.200(1)	1.713(3)	1.715(3)	1.318(4)
**7–Ni2** ^k^	0.05		1.898(3)	1.887(3)	2.200(1)	2.204(1)	1.714(3)	1.723(3)	1.311(4)

a Subscripts denote atom position on square-pyramidal/trigonal-bipyramidal limits; thus, N_ax/eq_ is the axial donor on the square pyramid and an equatorial donor on a trigonal bipyramid.

b Ref 74.

c 123 K (Figure S36).

d 293 K (Figure 2).

e 293 K (Figure 3).

f CH_2_Cl_2_ solvate at 173 K (Figure 3).

g 173 K (Figure 4).

h MeCN hemisolvate at 293 K (Figure 4).

i 293 K (Figure 5).

j 293 K (Figure 6).

k 123 K (Figure 8).

**Table 3:** Comparative Coordinate and Dithiocarbamate Bond Angles (°)^a^

Complex	τ^5^ ^b^	N_ax/eq_–Ni–N_eq/ax_	N_ax/eq_–Ni–N_eq/eq_	N_ax/eq_–Ni–S_eq/ax_	N_ax/eq_–Ni–S_eq/eq_	N_eq/ax_–Ni–N_eq/eq_	N_eq/ax_–Ni–S_eq/ax_	N_eq/ax_–Ni–S_eq/eq_	N_eq/eq_–Ni–S_eq/ax_	N_eq/eq_–Ni–S_eq/eq_	S_eq/ax_–Ni–S_eq/eq_	S_eq/ax_–C–S_eq/eq_
**1–Ni1** ^c^	0.29	90.77(7)	95.18(7)	96.05(5)	108.94(5)	84.13(7)	172.88(5)	100.43(5)	97.25(5)	155.29(5)	75.43(2)	115.3(1)
**1–Ni2** ^c^	0.22	88.77(6)	93.18(7)	89.71(5)	103.82(5)	88.45(6)	174.86(5)	98.05(5)	96.54(5)	161.87(5)	77.54(2)	111.8(1)
**1–Ni1** ^d^	0.28	90.70(5)	95.50(6)	96.84(4)	108.47(4)	84.31(5)	172.10(4)	100.22(4)	97.26(4)	155.45(5)	75.18(2)	115.40(9)
**1–Ni2** ^d^	0.32	91.78(5)	94.56(6)	93.85(4)	111.25(5)	85.66(6)	172.91(4)	97.91(4)	98.18(4)	153.73(4)	76.02(2)	114.9(1)
**2** ^e^	0.01					90.32(6)	172.86(4)	95.73(4)	96.16(4)	172.46(5)	78.06(2)	108.1(1)
**2** ^f^	0.03	88.50(5)	79.46(5)	98.56(3)	92.98(3)	90.81(6)	174.67(4)	96.27(4)	94.49(4)	172.61(4)	78.46(2)	108.56(9)
**3** ^g^	0.61	87.7(1)	96.3(1)	94.69(7)	124.11(7)	87.0(1)	175.32(7)	100.21(7)	96.77(7)	138.94(7)	75.12(3)	116.7(2)
**3** ^h^	0.01					90.34(6)	171.34(5)	94.38(5)	95.64(5)	170.65(5)	78.84(2)	109.7(1)
**4** ^i^	0.56	86.72(9)	92.42(9)	95.25(6)	138.78(7)	90.65(9)	172.45(6)	100.14(6)	96.54(7)	127.72(7)	73.60(3)	116.4(2)
**5** ^j^	0.00					90.53(7)	171.03(6)	93.41(5)	96.66(5)	170.98(5)	78.73(2)	109.7(1)
**7–Ni1** ^k^	0.03					90.5(1)	174.88(9)	96.22(8)	94.23(9)	172.89(9)	79.00(4)	109.4(2)
**7–Ni2** ^k^	0.05					90.1(1)	175.53(9)	96.93(9)	93.96(9)	172.54(9)	78.92(4)	109.0(2)

a Subscripts denote atom position on square-pyramidal/trigonal-bipyramidal limits; thus, N_ax/eq_ is the axial donor on the square pyramid and an equatorial donor on a trigonal bipyramid.

b Ref 74.

c 123 K (Figure S36).

d 293 K (Figure 2).

e 293 K (Figure 3).

f CH_2_Cl_2_ solvate at 173 K (Figure 3).

g 173 K (Figure 4).

h MeCN hemisolvate at 293 K (Figure 4).

i 293 K (Figure 5).

j 293 K (Figure 6).

k 123 K (Figure 8).

## 3 Results and Discussion

### 3.1 Synthesis and Characterization of Complexes 1–4

The dithiocarbamate complexes [(Tp^Ph,Me^)­NiS_2_CNR_2_] (NR_2_ = NMe_2_, **1**; NEt_2_, **2**; NPh_2_, **3**; NC_12_H_8_, **4**) were prepared. Several different organic *N*–substituents were chosen to induce a variety of steric and electronic effects on coordination of the dithiocarbamate coligand.^56,70–73^ The product complexes were obtained by reaction of [(Tp^Ph,Me^)­Ni–Cl] with the corresponding dithiocarbamate salts, NaS_2_CNR_2_. A change in color of the reaction solutions indicated conversion of the pink chloride precursor complex to the lime green product complexes. The products were crystallized using various techniques, particularly slow evaporation of CH_3_CN solutions or slow diffusion of *n*-hexane into CH_2_Cl_2_ solutions, and at different temperatures. We obtained green crystals of **1**, red crystals of **2**, and both red and green pseudopolymorphic crystals of **3** and **4**.

#### 3.1.1 X-ray Crystallography

The structures of complexes **1**–**4** were determined, along with those of three decomposition products **5–7** discussed below. Thermal ellipsoid plots are shown in Figures 2–8 and Figures S36 and S37. A solvated, thermally sensitive red crystal of **4** diffracted weakly; we obtained a model of relatively poor quality that is provided only as Supporting Information (Figure S37). Relevant coordinate bond lengths and angles, as well as those within the dithiocarbamate coligands, are given in the figure captions and compared in Tables 2 and 3, respectively.

**Table 4:** ^1^H NMR Chemical Shifts (ppm) Observed for Complexes **1**–**7** ^a^

	3-Ph						
Complex	*o*	*m*	*p*	4-H	5-Me	B–H	coligand
*paramagnetic*							
[(Tp^Ph,Me^)_2_Ni]	5.9	7.3	5.9	54.6	–1.6	–7.9	
[(Tp^Ph,Me^)Ni–Cl]	9.7	8.7	8.0	78.6	4.5	–13.7	
**1**	4.6	6.8	7.3	53.5	1.8	–8.4	43.3 (6H, NMe)
**2**	4.7	6.8	7.1	52.7	1.6	–8.5	34.0 (4H, NCH_2_); 1.1 (6H, Me)
**3**	4.4	6.8	7.1	54.2	1.9	–8.4	8.4 (4H, Ph); 7.7 (4H, Ph); 4.1 (2H, *p*-Ph)
**4**	4.3	6.6	6.8	60.4	2.8	–8.0	9.5 (2H); 9.2 (2H); 8.8 (2H); 8.6 (2H)
**6**	11.0–5.4 (15H)			66.5 (1H), 63.1 (2H)	2.6 (9H)	–9.7	47.3 (1H); 11.0–5.4 (5H); −10.2 (3H)
*diamagnetic*							
KTp^Ph,Me^ ^b^	7.72	7.20	7.11	6.35	2.25	5.0	
**2·H** ^**+**^	8.66 (4H)	7.60 (4H)	7.36 (2H)	6.29 (2H)	2.60 (6H)	5.0	2.86 (4H, NCH_2_); 0.63 (6H, Me)
	7.72 (2H)	7.48 (2H)	7.81 (1H)	6.64 (1H)	1.89 (3H)		
**3·H** ^**+**^	8.65 (4H)	7.57 (4H)	7.46 (2H)	6.25 (2H)	2.59 (6H)	5.0	7.13 (1H, *p*); 7.05 (2H); 6.59 (2H)
	7.77 (1H)	7.46 (2H)	7.77 (2H)	6.80 (1H)	1.86 (3H)		
**5**	10–6 (br, 10H)			6.87 (2H)	2.27 (6H)	^d^	7.38–7.29 (10H)
**7** ^c^	8.89	7.58	7.37	5.95	2.74	–	3.18 (6H, NMe); 3.05 (6H, NMe)

a In CDCl_3_ solution at 295 K, except as noted.

b In *d*_6_-acetone.

c In CD_2_Cl_2_.

d Not observed.

Red crystals of unsolvated **2**, **2**·CH_2_Cl_2_, **3**·0.5MeCN and **4**·CH_2_Cl_2_·C_6_H_14_ all hosted diamagnetic Ni­(II) complexes with square-planar or elongated square-pyramidal geometries. The axial pyrazolyl arm in unsolvated **2** (Figure 3, left) and in red **3** (Figure 4, right) were detached and rotated away from the metal, with H–B–N–N torsions of 133.2° and 83.5°, respectively. In contrast, the axial arm of **2**·CH_2_Cl_2_ exhibited an H–B–N–N torsion of 153.6°, and was thus disposed more axially over the Ni­(II) center at a nonbonding Ni···N distance of 3.001(2) Å (Figure 3, right). A similar disposition of the axial arm was observed for red **4** (Figure S37). In either case, the Ni­(II) ion was located within the equatorial N_2_S_2_ donor plane, such that the equatorial bond angles summed to ca. 360° (Table 3). Observed equatorial Ni–N and Ni–S coordinate bond lengths were on the order of 1.92 and 2.20 Å, respectively (Table 2 and Figure 9). By way of comparison, the averaged values for the two structures of base-off NiSOD were 1.95 ± 0.08 Å and 2.19 ± 0.03 Å, respectively.^17–21^

**Figure 9:**
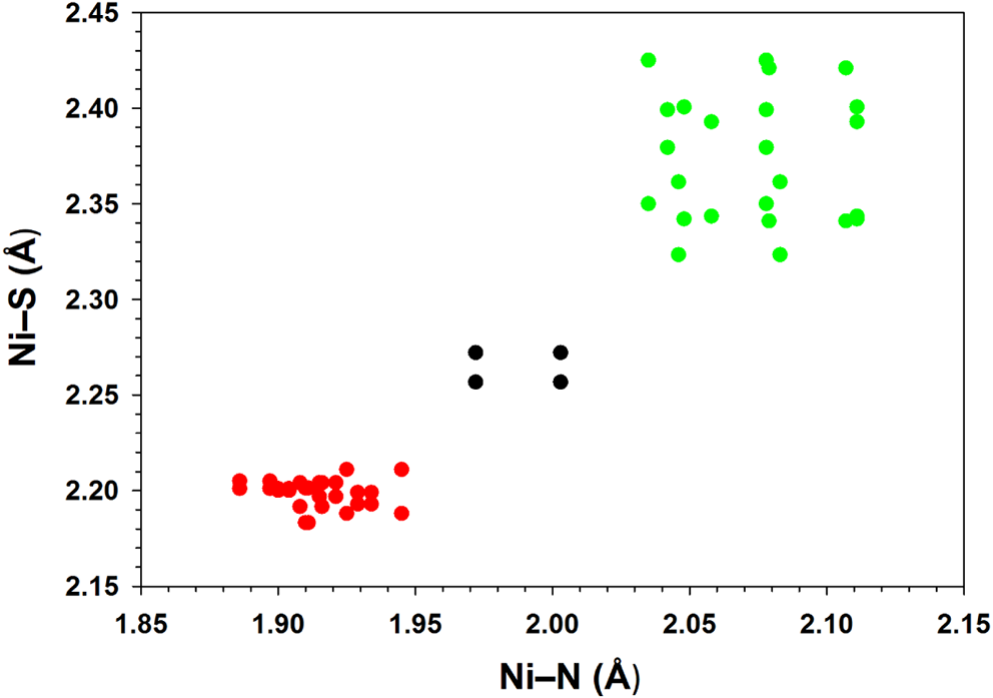
Plot of equatorial Ni–N vs Ni–S bond lengths for complexes **1**–**5** and **7** (i.e., Ni–X_eq/eq_ and Ni–X_eq/ax_, Table 2). Data for diamagnetic complexes are shown in red, and paramagnetic complexes in green; those rendered in black are for the Ni2 site of complex **1** at 123 K, which exhibits partial spin crossover.

Green crystals of **1**, **3** and **4** hosted paramagnetic pentacoordinate Ni­(II) complexes. The Ni–N and Ni–S bond lengths averaged 2.07 ± 0.04 and 2.37 ± 0.04 Å, respectively (Table 2 and Figure 9); these are much longer than the equatorial bonds in the red structures. The green conformations exhibit trigonal distortion along one N–Ni–S axis (i.e., τ^5^ = 0.2–0.6, Table 3).^74^ Given restricted chelate bites of the coligands (i.e., N–Ni–N ≈ 90°, S–Ni–S ≈ 75°), the τ^5^ geometry index is constrained to an upper limit well below unity. Indeed, least-squares N_2_S equatorial and NS_2_ axial planes meet at a dihedral angle of 89.6° in green **3,** and at 89.4° in green **4**. The independent molecules of **1** thus adopt a distorted square-pyramidal geometry (Figure 2 and Figure S36), while green **3** and **4** are distorted to the trigonal-bipyramidal limit (Figure 4, left and Figure 5).

The trigonal distortion reflects a pivot of dithiocarbamate relative to the face of the Tp^Ph,Me^ ligand, such that one N–Ni–S *trans* angle remains fixed near linearity along the emerging trigonal axis, while the other diverges (Figure S38). This pivot is accompanied by a shift in the resonance structure of the dithiocarbamate coligand (Scheme 2):^56,70–73^ the CS_2_ angle increases with increasing τ^5^ values (Table 3 and Figure S39); the C–S and Ni–S bond lengths diverge, with respective contraction and elongation evident along the trigonal axis (Table 2, Figures S39 and S40). The C=N bond also lengthens with increasing τ^5^ value (Table 2 and Figure S41), but the range is of marginal significance, and is more strongly affected by the various *N*–substituents between the dithiocarbamate ligands. It is tempting to speculate that trigonal distortion minimizes steric contact between the aromatic substituents on the 3-pyrazolyl positions and on the dithiocarbamates in **3** and **4**; however, conformational polymorphs of [(Tp^Ph,Ph^)­CoS_2_CN^*n*^Bu_2_] that exhibit τ^5^ values of 0.07 and 0.49 have been structurally characterized.^61^

The structure at one of two independent molecules in green **1** was found to be uniquely temperature-dependent. A structure determination at 293 K (Figure 2) revealed both molecules in square-pyramidal geometries with short apical Ni–N bond lengths (Table 2). Upon lowering the temperature to 123 K (Figure S36), the apical Ni2–N8 bond length at Ni2 increased from 2.149(1) Å to 2.401(2) Å (Table 2), with offsetting contractions of the equatorial coordinate bonds (Figure 9); in contrast, the structure at Ni1 remained constant within experimental uncertainty. As discussed previously,^54^ these observations reflect an incomplete solid-state spin crossover that occurs selectively at Ni2. Extrapolation to the diamagnetic limit gives an axial Ni2–N8 bond length of 2.61 Å, which further establishes the elongated square-pyramidal conformation found in solvated red **2** and **4**. Prior examples with strong-field equatorial coligands include [(Tp^Me,Me^)­Ni­(CN)_2_]^+^ and [(Tp)­Ni­(C_6_H_5_)­PMe_3_], with axial Ni···N distances of 2.389(2) and 2.57(1) Å, respectively.^75,76^ An elongated square-pyramidal geometry represents the spin isomer of the paramagnetic, compressed square-pyramidal geometry, and the other crystal structures suggest that further isomerization is facile, to square-planar and trigonal-bipyramidal conformations, respectively.

#### 3.1.2 ^1^H NMR Spectroscopy

The isolated ^3^A_2g_ electronic ground state, under ideal *D*_3*d*_ symmetry, in the homoleptic sandwich complex [(Tp^Me,Me^)_2_Ni] gives rise to chemical shifts dominated by the paramagnetic contact shift (i.e., through-bond spin delocalization).^77^ The 4-pyrazolyl ring proton resonance is shifted downfield significantly to 50.8 ppm (in C_6_D_6_ at 303 K), whereas the signals of the σ-bonded borohydride (−9.0 ppm) and the 3- and 5-methyl groups (−9.0 and −2.0 ppm, respectively) are shifted weakly upfield. The spectrum of the sandwich complex [(Tp^Ph,Me^)_2_Ni] in CDCl_3_ at 295 K is analogous (Figure S42 and Table 4).^78^ The trigonal precursor complex [(Tp^Ph,Me^)­Ni–Cl], of ideal *C*_3*v*_ symmetry, is distinguished only by modestly larger chemical shifts (Figure S43 and Table 4).^79^ The spectrum of diamagnetic K­(Tp^Ph,Me^) in *d*_6_-acetone is also consistent with effective *C*_3*v*_ symmetry (Figure S44 and Table 4).^80^

Complexes **1**–**4** dissolved to give uniformly green solutions, regardless of the color of the crystals. ^1^H NMR spectra of the individual complexes are compared in Figure 10 and Table 4 (and shown individually in Figures S45–S48). In each case, both halves of the dithiocarbamate ligands were equivalent, as well as all three pyrazole rings. The latter observation indicates that some dynamic process yields effective counter-rotation of the two coligands.

**Figure 10:**
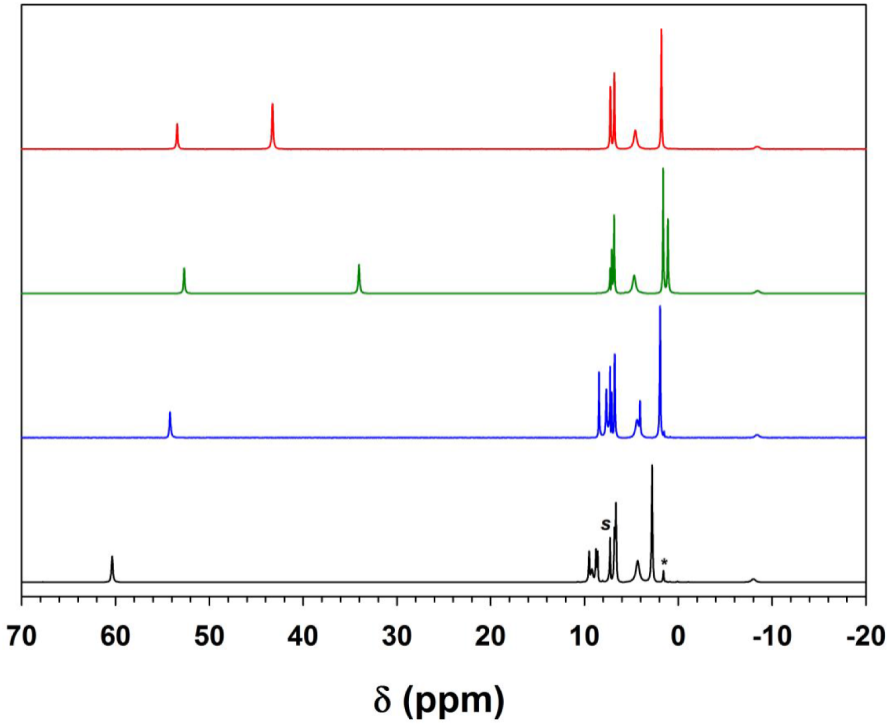
^1^H NMR spectra (CDCl_3_, 295 K) of complexes **1** (top, red), **2** (green), **3** (blue), and **4** (black, bottom). The labels *s* and * denote residual solvent and H_2_O, respectively.

Observed paramagnetic chemical shifts of the scorpionate ligand were generally intermediate between those of the sandwich complex [(Tp^Ph,Me^)_2_Ni] and the chloride complex [(Tp^Ph,Me^)­Ni–Cl] (Table 4). Large downfield chemical shifts were observed for the 4-H pyrazolyl resonances, generally increasing in the order **1** ≈ **2** ≤ **3** < **4**; as the paramagnetic shifts are proportional to local spin density, a modest corresponding trend in dithiocarbamate chelate strength is suggested. The *N*-methyl signal of **1** was observed at 43.3 ppm, compared to the *N*-methylene of **2** (34.0 ppm). The borohydride resonance was observed with a characteristic upfield shift. Other chemical shifts were generally closer to limiting diamagnetic values; one exception was the unique upfield shift of the 3-*ortho*-phenyl resonances (i.e., 4.3–4.7 ppm). The latter were also broadened by proximity to the paramagnetic Ni­(II) ion.

Magnetic susceptibilities of complexes **1**–**4** were measured in solution by the Evans NMR method in CDCl_3_ at 295 K.^63^ The solution susceptibility determined for **1** was 2.77 μ_B_, which approximates the spin-only value of 2.83 μ_B_ for an *S* = 1 ion, but is much less than the value of 3.45 μ_B_ previously extrapolated for the high-spin limit in the solid state.^53^ Therefore, we assess that an equilibrium of **1** between paramagnetic and diamagnetic isomers is established in solution, which yields a green:red mole fraction of ca. 64:36. The other complexes **2**–**4** appear to behave similarly, as observed solution susceptibilities typically fell near 2.5 μ_B_.

#### 3.1.3 FTIR Spectroscopy

Complexes **1**–**4** were characterized in the solid state by FTIR spectroscopy (as KBr pellets). Of particular interest were the ν­(B–H) modes of the Tp^Ph,Me^ ligand, the core ν­(C=N), ν_as_(NR_2_) and ν_as_(CS_2_) modes within the dithiocarbamate coligand, and any differences in these modes that arise from the Ni­(II) spin state and scorpionate ligand denticity. Bands arising from the dithiocarbamate coligand were distinguished by comparison with the spectrum of [(Tp^Ph,Me^)­Ni–Cl] (Figure S49).^79^

The IR spectrum of the potassium *N*-dimethyldithiocarbamate salt exhibits five prominent bands at 1498, 1361, 1257, 1125, and 966 cm^–1^.^81^ Normal mode analysis assigned δ­(Me) character to the first two bands, with the former strongly coupled to a weak adjacent mode of ν­(C=N) character. The third and fifth bands were assigned as admixtures of ν_as_(N–Me_2_) and ν_as_(CS_2_) character, while the fourth was taken to be a ρ­(Me) mode.^81^

The FTIR spectrum of green **1** displayed two ν­(B–H) modes, a major peak at 2529 cm^–1^ and a minor peak at 2474 cm^–1^, that are consistent with κ^3^- and κ^2^-scorpionate ligation,^57^ respectively (Figure 11). As for the free dithiocarbamate salt, the fingerprint region exhibited five relatively strong bands at 1508, 1392, 1257, 1153, and 985 cm^–1^ that had no counterparts in the spectrum of [(Tp^Ph,Me^)­Ni–Cl] (Figure S50). Unfortunately, the corresponding bands of the minor κ^2^-Tp component were not resolved.

**Figure 11:**
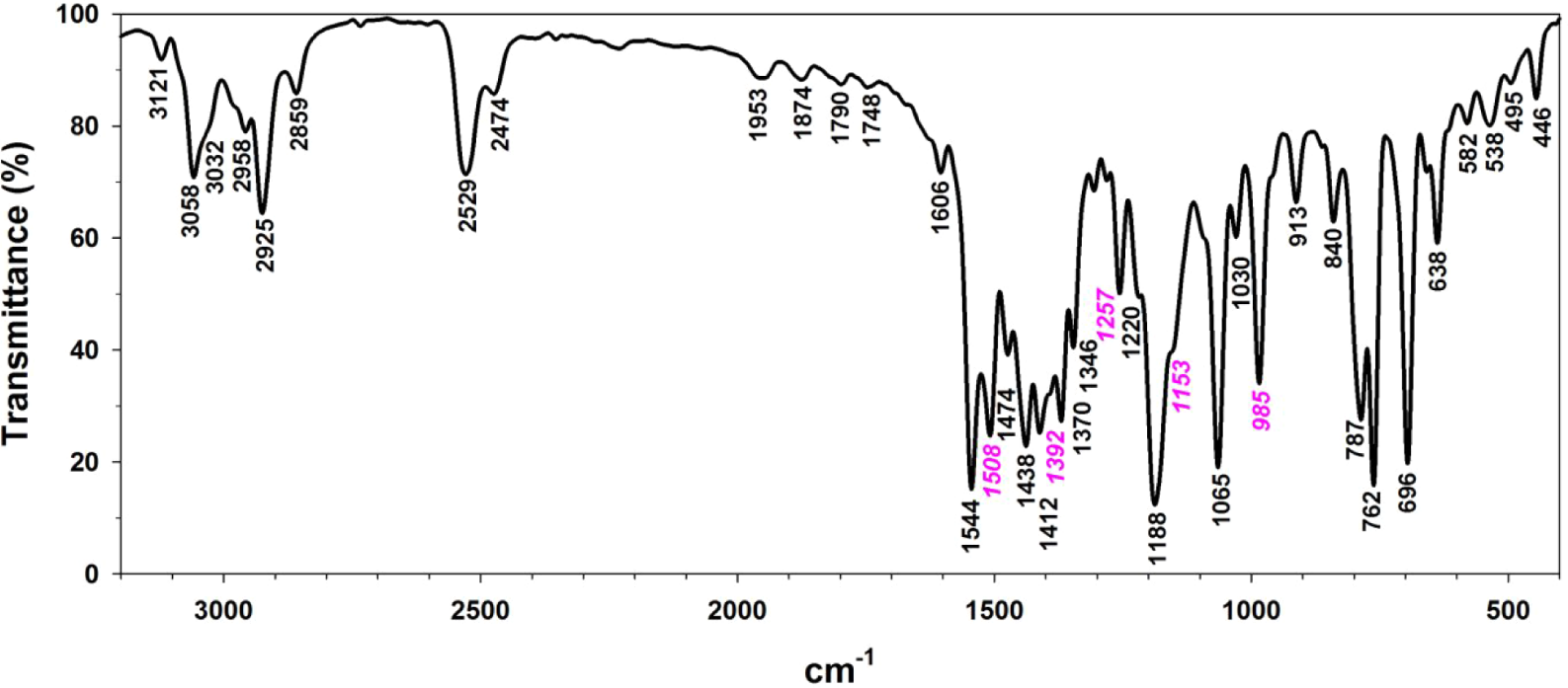
FTIR spectrum (KBr pellet) of complex **1**. Peaks labeled in italic pink do not coincide with any band in the spectrum of [(Tp^Ph,Me^)­Ni–Cl] (Figure S49) and are assigned accordingly to modes within the dimethyldithiocarbamate ligand.

The FTIR spectrum of complex **2**, obtained from red crystals, exhibited two ν­(B–H) modes, specifically a major peak at 2478 cm^–1^ and a minor peak at 2523 cm^–1^ (Figure S51). Again, five strong fingerprint bands not evident in the spectrum of [(Tp^Ph,Me^)­Ni–Cl] were observed at 1511, 1456, 1277, 1151, and 1003 cm^–1^ (Figure S52). The band at 1456 cm^–1^ appears to be the counterpart of the band of green **1** at 1392 cm^–1^; although both bands are likely convoluted with proximal *N*-alkyl deformations,^81^ they plausibly exhibit substantial ν­(C=N) character, such that the shift between them reflects a change in the dithiocarbamate resonance that affects the C=N π bond order (Scheme 2).

Complex **3** afforded a unique opportunity to compare distinct spectra of red and green crystals of the same complex (Figure 12). The spectrum obtained from red crystals is shown in Figure S53; the fingerprint region is compared with that of [(Tp^Ph,Me^)­Ni–Cl] in Figure S54. The spectrum obtained from green crystals is shown in Figure S55; the fingerprint region is compared with that of [(Tp^Ph,Me^)­Ni–Cl] in Figure S56. The green crystals exhibited a major ν­(B–H) mode at 2548 cm^–1^, and the red crystals at 2477 cm^–1^. A prominent fingerprint band observed at 1352 cm^–1^ for the green sample apparently shifted to 1407 cm^–1^ for the red sample, perhaps consistent with ν­(C=N) character. Unfortunately, additional core modes of the dithiocarbamate ligand were not resolved in either spectrum.

**Figure 12:**
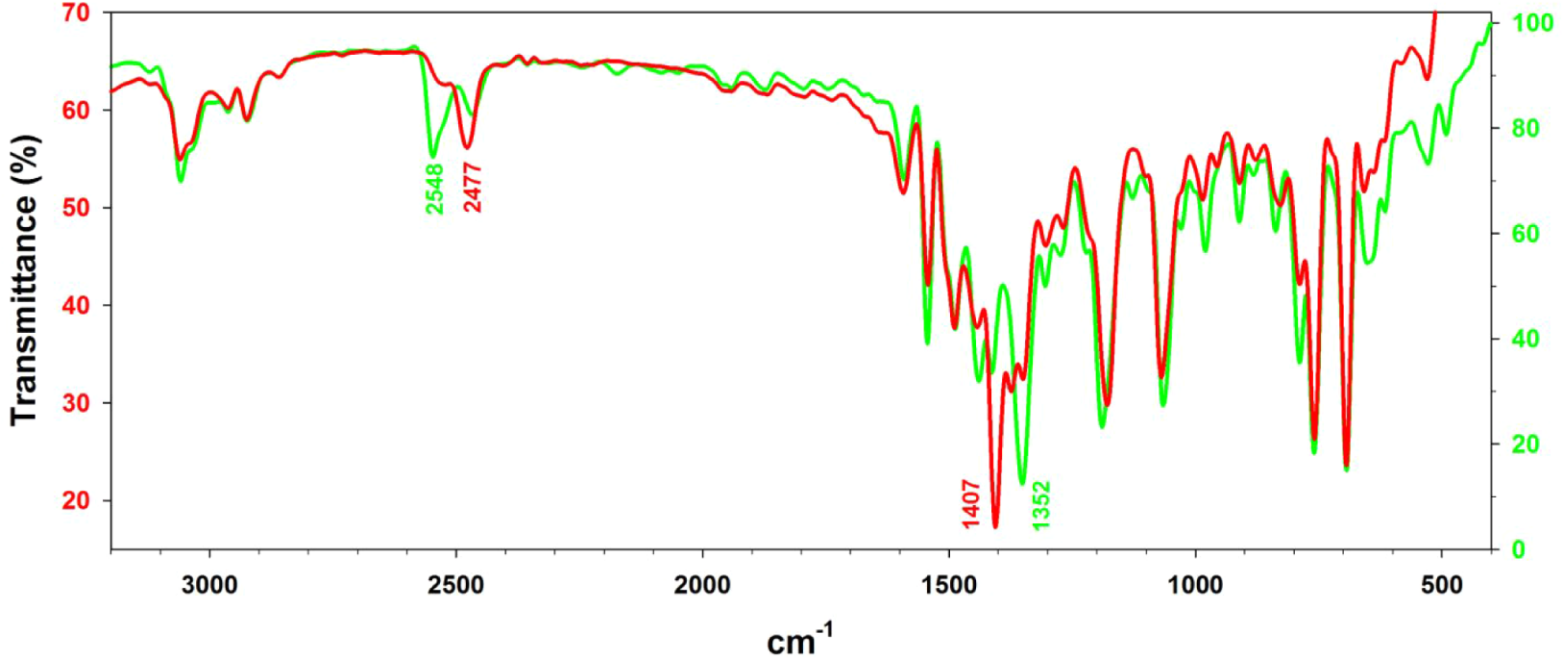
FTIR spectra (KBr pellets) of red and green crystals of complex **3**.

The FTIR spectrum obtained from green crystals of **4** displayed a single ν­(B–H) mode at 2546 cm^–1^ (Figure S57). Comparison with the spectrum of [(Tp^Ph,Me^)­Ni–Cl] revealed nine unique fingerprint bands (Figure S58). Six of these likely arise from lattice CH_2_Cl_2_ solvent and from the carbazole ring system.^82^ The other three bands, at 1295, 1164, and 1045 cm^–1^, might be attributed to the core ν­(C=N), ν_as_(NR_2_) and ν_as_(CS_2_) modes, respectively.

The FTIR spectra of complexes **1**–**4** confirm a substantial blue shift in the ν­(B–H) mode for κ^3^-scorpionate chelation in green crystals of paramagnetic complexes relative to κ^2^-scorpionate chelation in red crystals of diamagnetic complexes.^57^ A shift in the resonance structure within the dithiocarbamate ligand is also suggested. The ν­(C=N) mode is likely to be complicated by coupling to alkyl deformation modes in **1** and **2**,^81^ but the blue shift of a single band between the respective spectra, and between the green and red isomers of **3**, seems consistent with increased C=N bond order. The putative ν­(C=N) modes also appear to red-shift for the green isomers in the order **1** > **3** > **4**, which mirrors the trends in C=N bond length (Figure S41) and ^1^H NMR contact shifts (Figure 10). Taken together, the spectra suggest *N*-substituent and Ni­(II) spin-state effects on the resonance structure of the dithiocarbamate ligand (Scheme 2).

#### 3.1.4 UV–Vis–NIR Spectroscopy

The electronic spectra of dithiocarbamate complexes **1**–**4**, in CH_2_Cl_2_ at 295 K, are highly comparable (Figure 13). Green solutions of the four complexes exhibited a relatively weak ligand field band (ε ≈ 10^2^ M^–1^ cm^–1^) near 650 nm that is typical of a pentacoordinate, weak-field Ni­(II) complex.^83,84^ A second common feature was an intense band (ε = 5–6 × 10^4^ M^–1^ cm^–1^) near 240 nm, that can be assigned to π → π* transitions within the 3-phenyl substituents.^79^ Additional bands of intermediate intensities and wavelengths were evident in all four spectra that can be assigned as charge transfers involving the dithiocarbamate ligands. Finally, the spectrum of **4** also contains unique bands within the near-UV region that can be assigned to excitations within the carbazole moiety.^82^

**Figure 13:**
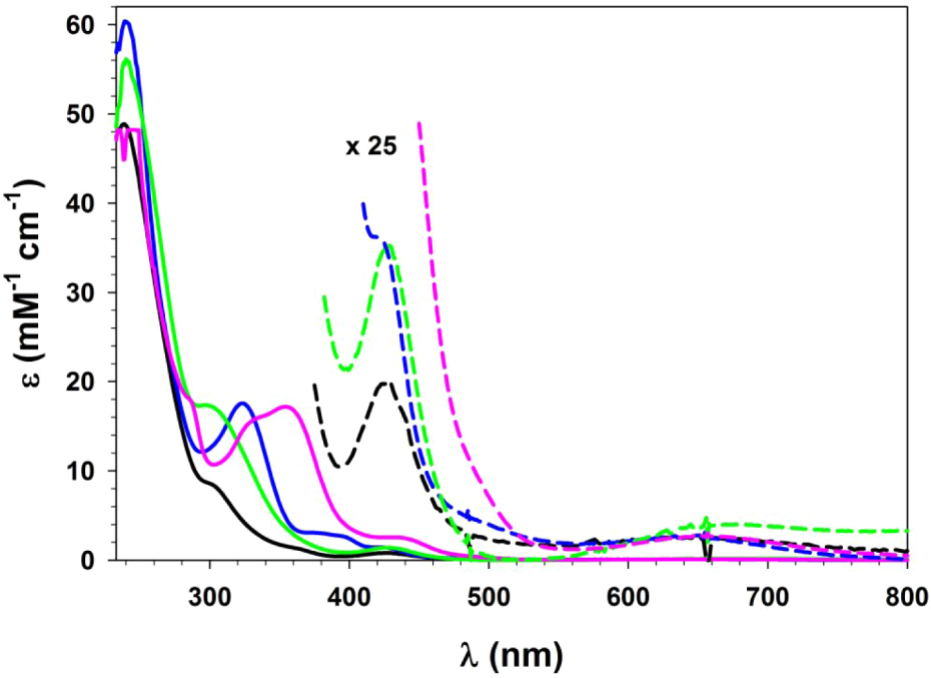
UV–vis–NIR spectra (CH_2_Cl_2_, 295 K) of complexes **1** (black), **2** (blue), **3** (green), and **4** (pink). Discontinuities evident in the dashed vertical expansions are Balmer lines that arise within the deuterium lamp.

### 3.2 Axial Base Equilibrium

Several lines of evidence suggest the model complexes **1**–**4** exhibit a dynamic axial base equilibrium in solution that is coupled to spin crossover (Scheme 1). Observed magnetic susceptibilities in solution all fall below spin-only values. Red crystals of diamagnetic Ni­(II) complexes were obtained from green solutions of **2**–**4**. Regardless of the crystal color, two ν­(B–H) modes were observed in the FTIR spectra of **1**–**3**. Finally, a spin equilibrium for Ni­(II) between elongated and compressed square-pyramidal ligand fields was observed for **1** even in the solid state.^54^

We first attempted to monitor the axial base equilibrium by ^1^H NMR spectroscopy. Spectra of a CDCl_3_ solution of **3** were obtained at 10° intervals between 238–298 K (Figure S59). The observed chemical shifts of both the 4-pyrazole and borohydride resonances gave empirically linear trends against inverse temperature (Figure S60), and the extrapolated intercepts fell close to expected diamagnetic chemical shifts (Table 4). However, it was not possible to deconvolute the temperature effect on spin population from that on axial base equilibrium. Alternative variation in solvent polarity had little effect on the observed spectra of **4** (Figures S61 and S62).

Addition of a slight excess of CF_3_CO_2_H to solutions of complexes **2** and **3** in CDCl_3_ changed the solution color from green to red by driving the axial equilibrium to a protonated base-off limit (Scheme 1). ^1^H NMR spectra of cationic proton adducts displayed the expected diamagnetic chemical shifts (Table 4) and a 2:1 mirror symmetry of coordinated and detached pyrazole arms (Figure 14). All expected resonances were fully resolved for **2·H** ^**+**^ (Figure S63); however, there was some accidental degeneracy in the 3-phenyl resonances of **3·H** ^**+**^ (Figure S64). Regardless, the data clearly establish that addition of CF_3_CO_2_H drives the axial base equilibrium to the base-off limit through protonation of the complex (Scheme 1).

**Figure 14:**
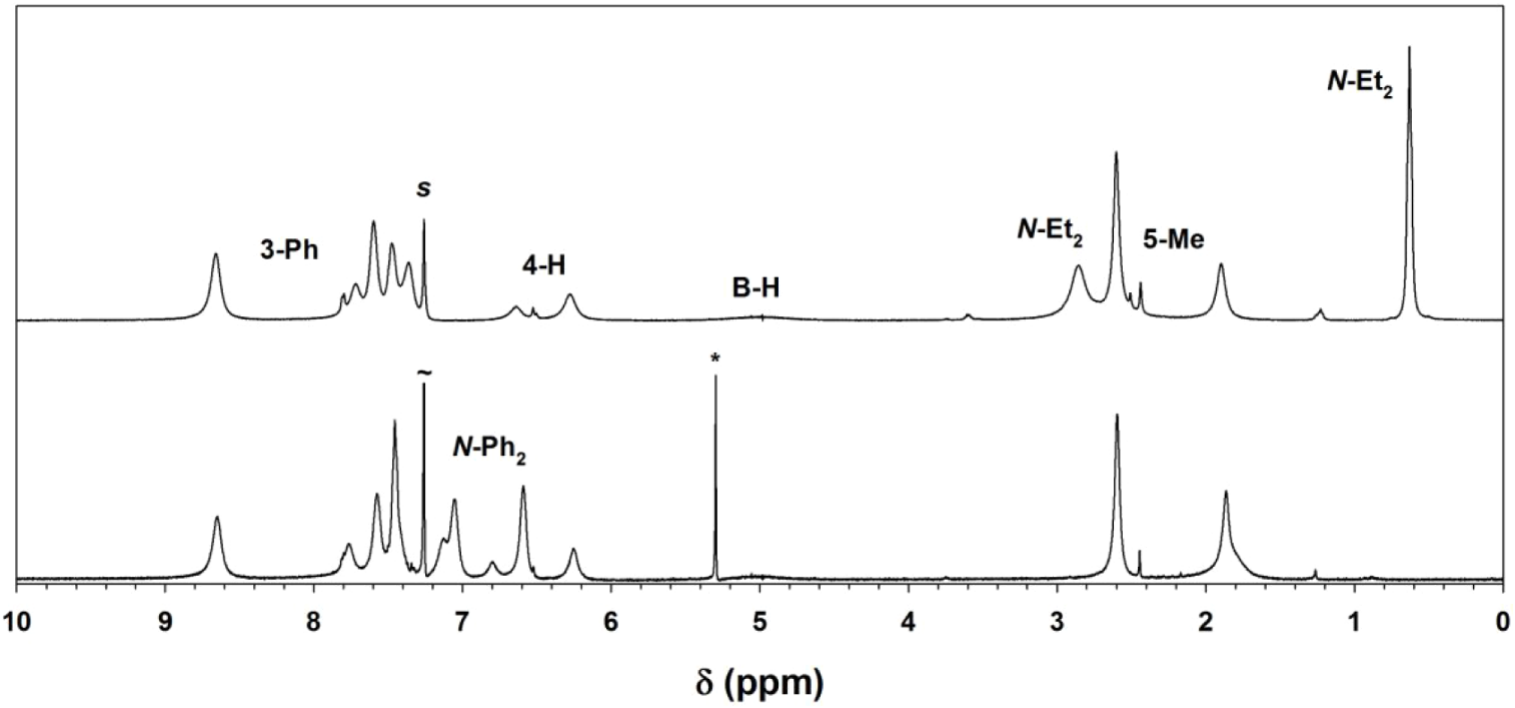
^1^H NMR spectra (CDCl_3_, 295 K) of complexes **2·H** ^**+**^ (top) and **3·H** ^**+**^ (bottom) in solution with added CF_3_CO_2_H. The residual solvent signal is marked (*s*) and truncated for clarity (∼) in the lower spectrum, and a CH_2_Cl_2_ impurity is denoted (*).

Titration of complex **3** with a stoichiometric amount of CF_3_CO_2_H was monitored alternatively by UV–vis spectroscopy (Figure 15). Three isosbestic points were observed (at 411, 477, and 598 nm). The ligand field band of the green form at 670 nm was replaced with a ligand field band at 520 nm in the red form, and the CT band at 429 nm was replaced with a stronger band rising from the isosbestic point at 411 nm. The color change from green to red was cleanly reversed by addition of excess NEt_3_.

**Figure 15:**
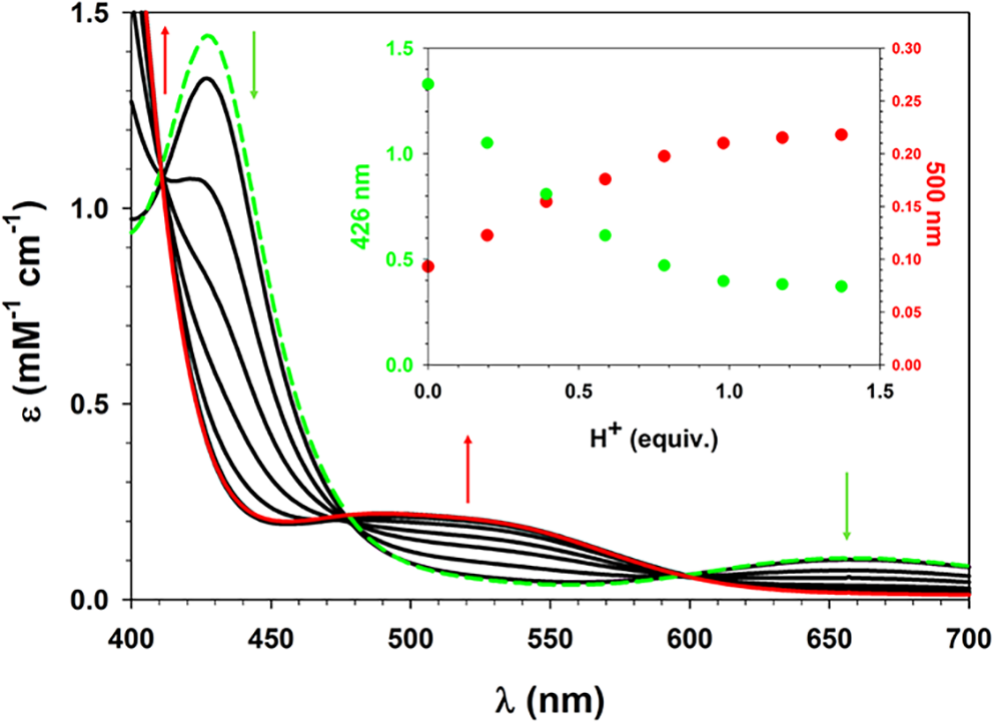
UV–vis spectra recorded during titration of complex **3** with CF_3_CO_2_H in MeCN at 293 K. Inset shows extinctions at 426 and 500 nm as a function of added acid. The dashed green line was recorded after a final addition of excess NEt_3_.

In order to directly observe the axial base equilibrium, a solution of **3** in MeCN was heated from 293–333 K, and electronic spectra were recorded at intervals of 10 °C. The obtained raw data (Figure S65) were corrected for thermal expansion of solvent (Figure 16).^85^ In order to fit the temperature-dependent absorbance to two-component equilibrium (eq 4), it was necessary to estimate the limiting extinctions of the red and green conformations. Extinction at 660 nm results only from the green isomer, and a 52% mole fraction at 293 K was estimated from the solution magnetic susceptibility. Extinction at this wavelength decreased with increasing temperature, and the observed shift in mole fractions between 293 and 333 K was used to extrapolate limiting spectra of the red and green isomers. This extrapolation demonstrates the presence of three isosbestic points at 414, 443, and 573 nm, and that the red isomer shows an absorption peak at 442 nm, with a broad shoulder near 540 nm. The change in absorption between the extrapolated limits was used to calculate van’t Hoff plots at four wavelengths, 400, 427, 470, and 660 nm (Figure 16 inset). The averaged slope of the colinear plots was −6.8(2) × 10^2^ and the averaged intercept was 2.23(7), which corresponds to ΔH° = 1.35(4) kcal/mol and ΔS° = 4.4(1) cal/mol·K.

4 $$\ln K=\ln\left({[red]}_{T}/{[green]}_{T}\right)=-\Delta G^{{}^{\circ}}/RT=\ln\;\{\left(A_{green}-A_{T}\right)/\left(A_{T}-A_{red}\right)\}$$

**Figure 16:**
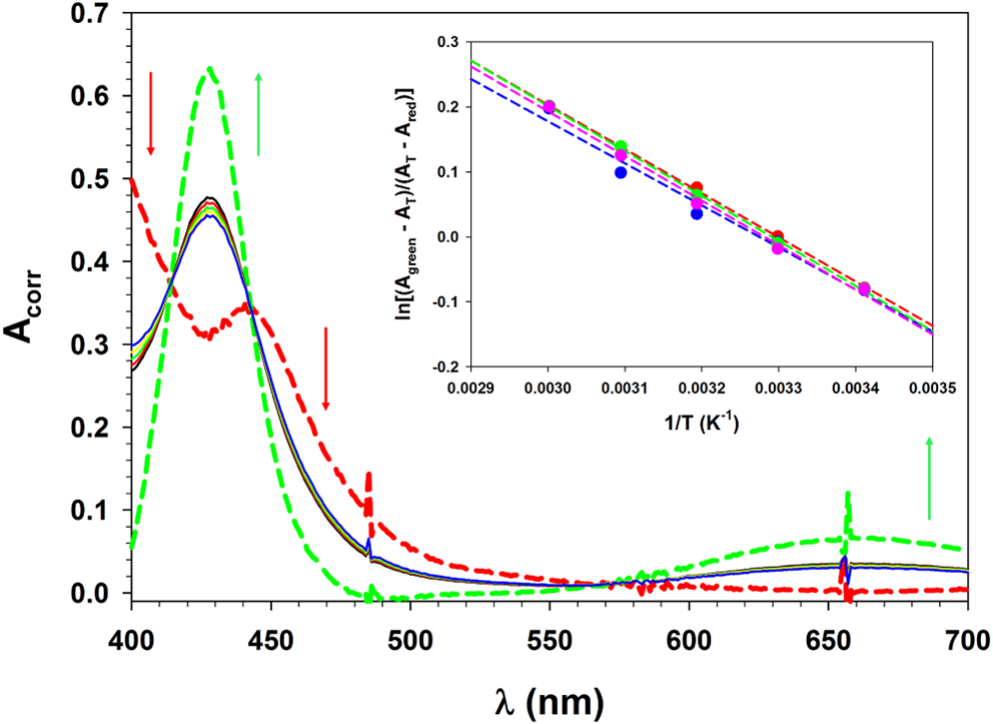
Temperature-dependent UV–vis spectra of complex **3** in MeCN, corrected for change in solvent density. The dashed lines are extrapolated absorption limits of the red and green isomers. The inset shows van’t Hoff plots of absorbance change at 400 nm (pink: *y* = 2.3(1) − 688(30)*x*, *r* ^2^ = 0.994), 427 nm (red: *y* = 2.24(3) − 680(89)*x*, *r* ^2^ = 0.999), 470 nm (green: *y* = 2.28(4) − 694(13)*x*, *r* ^2^ = 0.999) and 660 nm (blue: *y* = 2.1(2) − 648(61)*x*, *r* ^2^ = 0.974).

The extrapolated spectrum of red **3** differed slightly from the observed spectrum of the protonated red adduct **3**·**H** ^+^. In particular, the 520 nm feature of **3**·**H** ^+^ was more prominent, as the 442 nm band of the thermal species was absent and presumably blue-shifted. There are two plausible explanations: first, protonation of the axial donor nitrogen causes inductive effects through boron and into the coordinated equatorial pyrazolyl rings that shifts the 442 nm band; second, spectra of diamagnetic square-planar and elongated square-pyramidal conformations might differ somewhat. We believe the first explanation is correct; the second would require that the ratio of mole fractions for the two red forms remain fixed in order to maintain the isosbestic points observed in the thermal equilibrium (Figure 16).

### 3.3 Reaction with Superoxide and Protons

Attempts to crystallize the protonated adduct **3·H** ^**+**^ failed, as this species slowly decomposes upon standing. We obtained both red and green crystals from the red mother liquor, but the colors were somewhat different than for **3**. The red crystals were magenta in tone, and the green crystals were emerald rather than lime green. X-ray crystallography showed that the magenta complex **5** was [(HB­{pz^Ph,Me^}_2_{OC­(O)­CF_3_})­NiS_2_CNPh_2_], which contains a altered scorpionate ligand (Figure 6). The emerald crystals proved to be [(κ^3^-Tp^Ph,Me^)­Ni­(Hpz^Ph,Me^)­(OC­{O}­CF_3_)] (**6**, Figure 7).

Complex **5** results from nucleophilic substitution at boron, in which the protonated pyrazole arm of **3·H** ^**+**^ is displaced by the trifluoroacetate conjugate base (Scheme 3). The displaced Hpz^Ph,Me^ equivalent can attack a second molecule of **3·H** ^**+**^; this results in loss of the neutral dithiocarbamic acid and coordination of both Hpz^Ph,Me^ and trifluoroacetate at Ni­(II) in **6**. This reaction entails proton transfer from the axial base to the departing dithiocarbamic acid, perhaps analogous to a proton transfer between the axial His-1 base and Cys-2 or Cys-6 thiolate of NiSOD. It is possible that the liberated dithiocarbamic acid might react further with **3·H** ^**+**^ to extract Ni­(II) and form equivalent [Ni­(S_2_CNPh_2_)_2_] and [H_2_Tp^Ph,Me^] [O_2_CCF_3_]. Some additional colorless to pale yellow material was also obtained, but no efforts were made to identify additional coproducts.

**Scheme 3:**
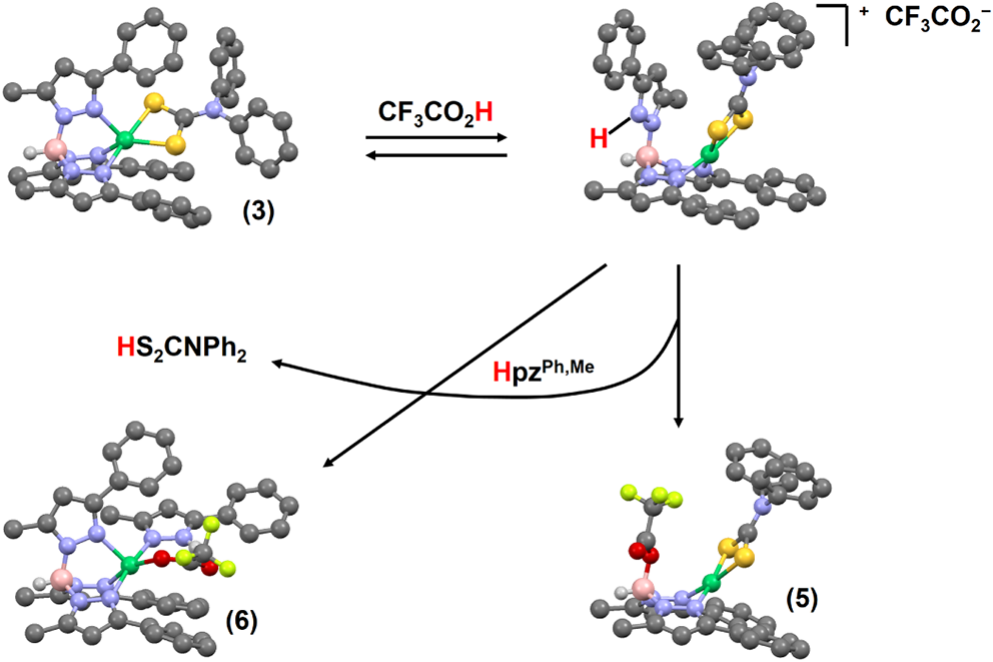
Summary of Proton-Induced Decomposition of **3**

Complex **1** was reacted with KO_2_ in acetonitrile solution, in the presence of 18-crown-6. No dismutation catalysis was anticipated, insofar as no source of labile protons was available under such conditions. Instead, the superoxide acted as another nucleophile toward boron. The complex was apparently cleaved into HB­(pz^Ph,Me^)_2_ and [(pz^Ph,Me^)­NiS_2_CNMe_2_] fragments, and the latter dimerized to form [(μ-1,2-pz^Ph,Me^)­NiS_2_CNMe_2_]_2_ (**7**), as confirmed by X-ray crystallography (Figure 8).

The magenta complex [(HB­{pz^Ph,Me^}_2_{OC­(O)­CF_3_})­NiS_2_CNPh_2_] (**5**) features an altered, bidentate scorpionate ligand (Figure 6). The remaining equatorial pyrazoles support a square-planar geometry with bond lengths that are virtually identical to those of **2** and the red form of **3** (Table 2). The introduced carbonyl (C21/O2) was rotated away from the axial vector of Ni­(II), to a H–B1–O1–C21 torsion of 61.5° and a nonbonded Ni···O2 distance of 4.516(2) Å. The heteroscorpionate ligand of **5** is not without precedent. Ghosh and Parkin obtained a formate analogue by insertion of CO_2_ into the axial B–H bond of [(Bp^*t*Bu,*i*Pr^)­Zn–Cl] (where Bp = dihydrobis­{pyrazolyl}­borate); one oxygen was bonded to boron and the other was ligated to Zn­(II) to give a distorted trigonal-pyramidal product complex.^86^ Fujita et al. prepared a ligand with one acetate and two *N*-methylimidazolyl donors; the acetate carbonyl was not coordinated in X-ray crystal structures of several square-planar and octahedral complexes of Ni­(II).^87,88^

The ^1^H NMR spectrum of **5** (Figure S66) displays diamagnetic chemical shifts (Table 4) that are consistent with the square-planar geometry. A single 5-methyl resonance was observed, which is consistent with *C*_*s*_ symmetry. The resonances were unusually broad, which suggests slow rotation of the pair of chemically equivalent phenyl rings on either of the two coligands; however, such behavior was not evident in the spectrum of **3·H** ^**+**^. The FTIR spectrum of **5** (Figure S67) displays a single ν­(B–H) mode at 2511 cm^–1^; this unusual frequency presumably reflects the presence of trifluoroacetate in place of an axial pyrazolyl arm. A ν­(C=O) mode of the trifluoroacetyl ester was evident as a strong band at 1676 cm^–1^, and bands observed between 1128 and 1197 cm^–1^ can be plausibly assigned ν­(CF_3_) character. A strong band at 1410 cm^–1^ was assigned to the ν­(C=N) mode, which compares to 1407 cm^–1^ for red **3**. The electronic spectrum of **5** (Figure S68) exhibited four shoulders, at ca. 300, 370, 420, and 520 nm, on a monotonic rise into the UV region. We note that the 420 nm feature compares to a similar feature at 440 nm in the extrapolated spectrum of red **3** (Figure 16), which may be further blue-shifted in the spectrum of of **3·H** ^**+**^ (Figure 15). This suggests an inductive effect of the uncoordinated axial groups through boron that affects the equatorial coordination and the energy of this transition.

The structure of [(κ^3^-Tp^Ph,Me^)­Ni­(Hpz^Ph,Me^)­(OC­{O}­CF_3_)] (**6**) was pentacoordinate (Figure 7), with a distorted square-pyramidal N_4_O ligand field (τ^5^ = 0.17). The trifluoroacetate anion was monodentate, with a Ni1–O1 bond length of 2.047(2) Å and a nonbonding Ni1···O2 distance of 3.543(2) Å. The C–O1 bond length of the coordinated oxygen was 1.254(3) Å; the C–O2 bond length of the uncoordinated oxygen was 1.222(3) Å. The Hpz^Ph,Me^ ligand was coordinated to Ni­(II) through 2-nitrogen (i.e., proximal to the 3-methyl substituent), and a proton on the 1-nitrogen (i.e., proximal to the 5-phenyl substituent) was hydrogen-bonded to the distal oxygen on the trifluoroacetate ligand. The Ni1–N7 coordinate bond length to the Hpz^Ph,Me^ ligand was 2.055(2) Å, compared to an average Ni–N bond length of 2.07 ± 0.03 Å to the three aza donors on the intact scorpionate ligand. Overall, the structure of **6** closely conforms to that of the benzoate analogue, [(Tp^Ph,Me^)­Ni­(Hpz^Ph,Me^)­(O_2_CPh)],^89^ except that the latter exhibits a slightly larger trigonal distortion (τ^5^ = 0.23), and the Ni–OC­(O)­R bond length to the relatively basic benzoate anion is significantly shorter, 1.993(2) Å.

The ^1^H NMR spectrum of **6** (Figure S69) shows a 1:2:1 pattern of paramagnetically shifted 4-pyrazole proton resonances (Table 4) that is indicative of dynamic *C*_*s*_ symmetry. A dynamic process must interconvert the enantiomers of the chiral static structure without achieving equilibration of all three pyrazole donor arms, as in **1**–**4**. One possibility is the exchange of carboxylate oxygen atom coordination across opposing faces of the pyrazole. Seven of the nine expected aromatic signals were resolved. An unusual upfield shift was observed for the methyl substituent on the pyrazole ligand, which is uniquely displaced inward toward the Ni­(II) ion. Unfortunately, the other methyl resonances are not interpretable; two peaks in a 9.8:8.0 ratio, as opposed to the expected 6:3, were observed at 2.6 and 1.0 ppm. We suggest that at least one of these reflects the presence of an unidentified organic impurity, and that the authentic signals may be accidentally degenerate.

The ν­(B–H) mode was observed at 2546 cm^–1^ in the FTIR spectrum of complex **6** (Figure S70). Unique bands were identified in the fingerprint region, at 1666, 1197, 1138, and 722 cm^–1^, by comparison with [(Tp^Ph,Me^)­Ni–Cl] (Figure S71). The first of these can be confidently assigned to the carboxylate ν_as_(CO_2_) mode, the next two likely represent ν­(CF_3_) character, and the last may be a δ­(CF_3_) mode. Unfortunately, the ν­(N–H) mode was not resolved from background arising from residual H_2_O. Electronic spectra of **6** (in CH_2_Cl_2_ at 295 K) were recorded at two different concentrations (Figure S72). Observation of a ligand field band at 640 nm is consistent with a pentacoordinate, high-spin structure.^83,84^ A band at 940 nm, observed at lower concentration, likely results from reversible dissociation of the Hpz^Ph,Me^ ligand.^79^

The dimeric structure of **7** affords equivalent square-planar ligand fields at both Ni­(II) centers. Coordinate bond lengths and angles were equivalent to those in **2** within experimental error (Tables 2 and 3). Both pyrazoles were deprotonated and bridged between the two Ni­(II) centers through their adjacent nitrogen atoms; the dithiocarbamate ligands were bound symmetrically (Figure 8). The Ni1···Ni2 separation was 3.0786(9) Å, and the dihedral angle between equatorial least-squares planes was 76.0°. The bis­(pyrazolate)-bridged structure has some precedent.^90^

The ^1^H NMR spectrum of **7** (in CD_2_Cl_2_ at 295 K; Figure S73) exhibited diamagnetic chemical shifts (Table 4) that are consistent with square-planar coordination of Ni­(II). The spectrum resembled that of **2·H** ^**+**^, except for the resonances of the missing axial arms. Only one set of pyrazole resonances were observed, and the *N*-methyl signals showed pairwise degeneracy. It is not clear whether this results from *C*_2_ or *C*_*s*_ symmetry, from a head-to-tail structure as observed in the X-ray crystal structure (Figure 8), or from a head-to-head isomer. Indeed, some small unassigned NMR resonances were evident, so the presence of both isomers cannot be excluded.

### 3.4 Cyclic Voltammetry

The redox properties of complexes **2**, **3**, and **5** were determined by cyclic voltammetry in CH_2_Cl_2_ solutions with 0.1 M ^*n*^Bu_4_N^+^PF_6_^–^ electrolyte vs external ferrocene (Fc/Fc^+^, Figures S74–S77).^91^ Reversible one-electron couples were observed in all cases, with *E*°′ = −0.08 V for **2**, 0.00 V for **3**, and 0.03 V for **5**. The midpoint for superoxide dismutation (+0.29 V vs NHE)^34^ extrapolates to −0.17 V versus Fc/Fc^+^ herein.^91^ The model complexes exhibit redox properties relevant to SOD activity. A comparison of the voltammograms for **3** and **5** shows little difference (Figure 17) despite the absence of an axial base in the latter. It may be that the base-off isomer of **3** is oxidized preferentially, or that an axial interaction serves only to direct the oxidized complex into a stable Ni­(III) valence state. Regardless, the reversibility of the redox couples indicates that the oxidized species are stable on a time scale of seconds.

**Figure 17:**
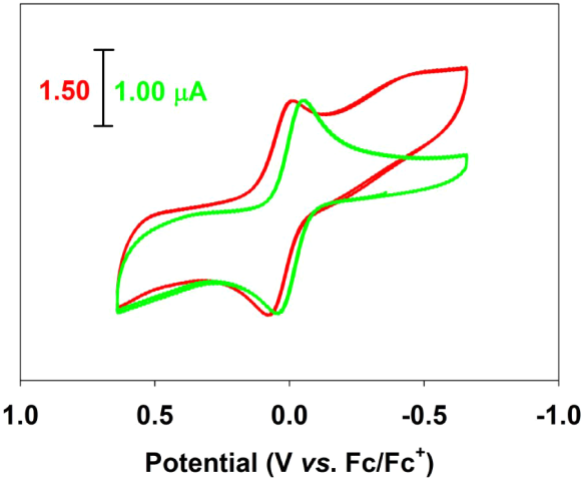
Cyclic voltammograms of **3** (green) and **5** (red). Recorded in CH_2_Cl_2_ solution with 0.1 M ^*n*^Bu_4_NPF_6_ at a scan rate of 100 mV/s at 295 K.

### 3.5 DFT Calculations

Geometry optimizations on simplified [(Tp)­NiS_2_CNMe_2_] and nickel hook models were undertaken in order to compare observed geometric structures, calculated electronic configurations and spin states. TD-DFT calculations were done in order to assign and compare the electronic spectra. Frequency calculations were performed on [(Tp)­NiS_2_CNMe_2_] models and the dithiocarbamate free anion in order to confirm assignments of FT-IR spectra.

#### 3.5.1 Geometry Optimizations

Simplified [(Tp)­NiS_2_CNMe_2_] models, with hydrogen atoms in place of the 3- and 5-pyrazolyl substituents, were optimized for low-spin Ni­(II) in square-planar (*1*), elongated square-pyramidal (*2*) and trigonal-bipyramidal geometries (*4*); the latter two geometries were reoptimized for high-spin Ni­(II) (*3* and *5*, respectively). Oxidized cations were optimized in square-pyramidal and trigonal-bipyramidal geometries (*6* and *7*, respectively). [(Bp)­NiS_2_CNMe_2_]^*n*+^ models that lack an axial base were also calculated (*n* = 0, model *8* and *n* = 1, model *9*). All nine models converged successfully, but the obtained electronic structures of *4* and *9* were problematic. The calculated HOMO–LUMO gap of *4* was too small (<0.6 eV, Table S42) to stabilize the low-spin configuration. The Mulliken spin density at the nickel ion in *9* was relatively low for Ni­(III) (Table S29), the spin density and charge on sulfur were unusual, and the frequency calculation exhibited a strong imaginary pyrazole ring deformation mode; these anomalies are suggestive of radical character on the ligands.

For comparative purposes, computational models of the nickel hook were derived from the published crystallographic coordinates (Scheme S1).^17,18^ Two nonessential side chains were reduced, specifically D3A and L4G (the former is known to retain fractional SOD activity),^34^ and the peptide chain was truncated after the sixth residue as a *C*-terminal amide. To maintain a neutral charge in the reduced state, a proton was added on the terminal amide nitrogen. The geometry was optimized with high- and low-spin Ni­(II) (*S* = 1 and 0, models *A* and *B*, respectively). In the first case, the terminal proton migrated into position for hydrogen-bonding with the cysteine-6 thiolate sulfur (Figure S1); in the second, the proton transferred completely to give a thiol ligand on Ni­(II) (Figure S2). The unrestrained His-1 imidazole side chain remained in an axial position in model *B*. A diamagnetic Ni­(II) model *C* was optimized after the axial His-1 base was detached from Ni­(II) and the proton transferred to this base (Figure S3). Consistent with observed structures,^17–21^ model *C* was favored by 0.4–0.5 eV over model *B*, and by 0.9–1.4 eV over *A*, regardless of the functional (BP86, OPBE or B3LYP*, Table S1). Finally, a neutral Ni­(III) model *D* (*S* = 1/2) was optimized without the added proton (Figure S4).

Among the synthetic models, low-spin, elongated square-pyramidal *2* was energetically preferred for Ni­(II) using the BP86 and OPBE functionals (Table S21), but the base-off model *1* was essentially degenerate (0.00–0.02 eV). The hybrid B3LYP* functional favored the high-spin analogue *3*. However, differences in calculated energies of Ni­(II) spin isomers were always sufficiently small to suggest that thermal equilibration is possible. Trigonal-bipyramidal geometries were slightly higher in energy, by <0.1 eV for high-spin Ni­(II) and Ni­(III), and by ca. 0.3 eV for low-spin Ni­(II) (Tables S21 and S22). Tetrahedral geometries and high-spin Ni­(III) were strongly disfavored (Tables S22–S24).

Comparison of models *1*, *2* and *8* showed coincident equatorial Ni–N and Ni–S bond lengths of 1.94 and 2.24 Å, respectively (Table S25). Uniform dithiocarbamate C=N bond lengths of 1.33 Å were also calculated, and the axial Ni···N distance in *2* was 3.06 Å. Compared with the experimental values observed in two structures of **2**, in red **3,** in **5** and in **7** (Table 2), the various calculated distances were longer by ca. 0.01–0.05 Å. The calculated CS_2_ bond angles of 108.0–108.1° in *1*, *2* and *8* (Table S27) compare to a range of 108.1(1)–109.7(1)° in the five crystal structures just enumerated (Table 3).

The optimized geometry of our base-off nickel hook model *C* can be compared to corresponding synthetic models *1* and *8*, to the crystal structures of our red synthetic complexes **2**, **3**, **5**, and **7**, to previous DFT models,^24,30,31,37^ and to the base-off conformation of NiSOD itself.^17–19,21^ Comparisons of coordinate bond lengths (Table 5) and angles (Table S55) are remarkably consistent across this range of examples. Moreover, an overlay plot of the optimized nickel hook model *C* with the first six residues of NiSOD in the experimental base-off conformation (1Q0M.pdb)^19,21^ gives good agreement, based on least-squares alignment of the equatorial ligand donor atoms (Figure 18).^69^ Previous assignments of the base-off enzyme conformation as low-spin Ni­(II) are clearly supported.^17–22^

**Figure 18:**
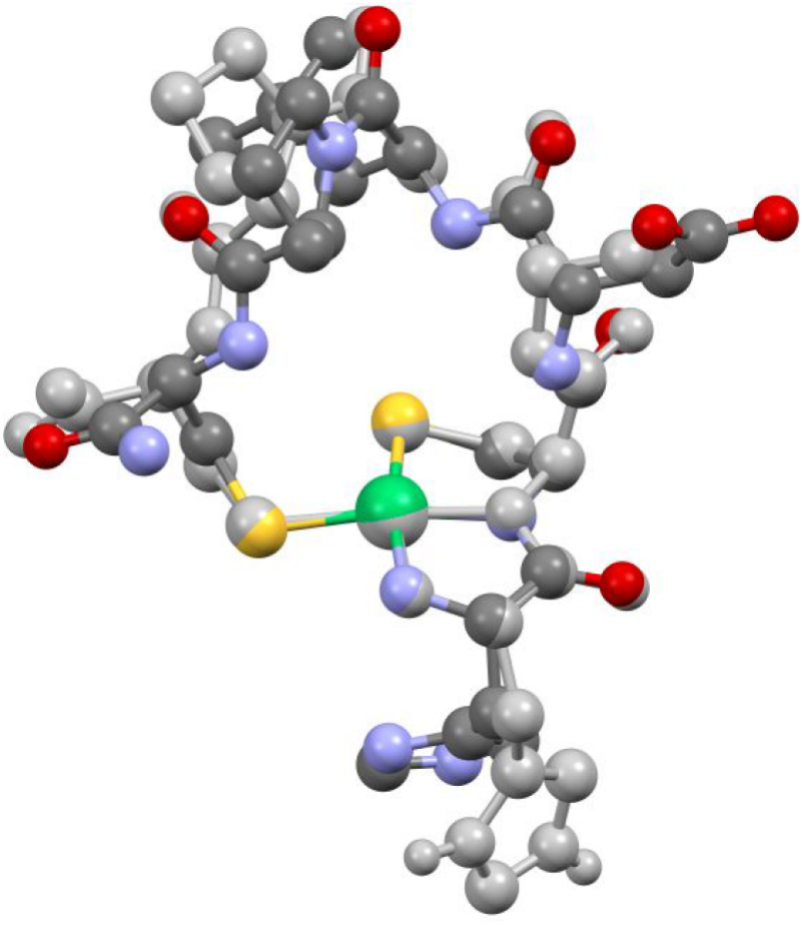
Least-squares overlay of the equatorial donor atoms in the experimental NiSOD base-off conformation (1Q0M.pdb, in color)^19,21^ with the base-off, low-spin Ni­(II) model *C* (rendered in gray). Hydrogen atoms are omitted for clarity, except for the doubly protonated base of the model.

**Table 5:** Coordinate Bond Lengths (Å) of Base-Off NiSOD vs Analogous Models

data set/model	Ni–N1_eq_	Ni–N2	Ni–S2	Ni–S6	Ni···N_2_S_2_	Ref
base-off NiSOD (X-ray)						
1T6U.pdb	1.87(6)	1.91(3)	2.16(2)	2.19(2)	–0.01	^17,18^
1Q0M.pdb	2.07	1.94	2.24	2.18	–0.07	^19,21^
base-off Ni(II) hook (DFT)						
Ni^II^-off-ls	2.02	1.91	2.19	2.24	–0.13	^37^
red	1.98	1.89	2.18	2.22	–0.04	^24^
red-S6H	1.99	1.86	2.18	2.19	–0.13	^24^
I_0_	1.97	1.85	2.22	2.28		^31^
4′	1.98	1.91	2.25	2.31	–0.14	^30^
*C*	2.05	1.93	2.21	2.25	–0.03	^a^
base-off Ni(II) synthetic complexes (X-ray)						
**2** (293 K)	1.93/1.93		2.19/2.20		–0.01	^a^
**3**·0.5MeCN	1.91/1.91		2.18/2.20		–0.12	^a^
**5**	1.91/1.92		2.19/2.20		–0.11	^a^
**7** (Ni1)	1.90/1.90		2.20/2.20		–0.04	^a^
**7** (Ni2)	1.89/1.90		2.20/2.20		–0.04	^a^
base-off Ni(II) models (DFT)						
*1*	1.94/1.94		2.24/2.24		–0.02	^a^
*8*	1.94/1.94		2.24/2.24		0.02	^a^

a This work.

The cationic oxidized model *6* adopted a significantly shorter axial interaction of 2.05 Å, compared with 3.06 Å in *2*; the equatorial coordinate bonds were slightly longer (Table S26). Significant displacement of nickel from the equatorial plane, toward the axial donor, was also evident in *6*. Unfortunately, no structural data are available for comparison against this DFT model. The neutral oxidized nickel hook model *D* adopted a structure consistent with *6* and with the previous DFT models (Table 6).^24,30,31,37^ The equatorial coordinate bond lengths were similar to those reported for the base-on conformation of NiSOD; however, the calculated axial Ni–N bond distance of 2.10 Å was significantly shorter and nickel was displaced from the equatorial plane, as in *6*. As a result, the calculated N–Ni–S *trans* angles were smaller in *6* and *D* (Table S56) than in *2* and *B*.

**Table 6:** Coordinate Bond Lengths (Å) of Base-On NiSOD vs Analogous Models

data set/model	Ni–N1_ax_	Ni–N1_eq_	Ni–N2	Ni–S2	Ni–S6	Ni···N_2_S_2_	Ref
base-on NiSOD (X-ray)							
1T6U.pdb	2.35(5)	2.02(10)	1.91(3)	2.16(2)	2.19(2)	+0.04	^17,18^
1Q0D.pdb	2.63	2.11	1.93	2.24	2.26	–0.01	^19,20^
Ni(III) hook (DFT)							
ox^1^	2.07	2.03	1.93	2.18	2.24	+0.27	^24^
Ni^III^-on	2.00	2.00	1.90	2.14	2.18	+0.22	^37^
1	2.07	2.04	1.95	2.28	2.32	+0.23	^30^
I_a3_	2.10	2.01	1.92	2.23	2.29		^31^
*D*	2.10	2.04	1.97	2.22	2.27	+0.31	^a^
Ni(III) model (DFT)							
*6*	2.05	1.99/1.99		2.26/2.26		+0.23	^a^
base-on, low-spin Ni(II) hook (DFT)							
Ni^II^-on-ls	2.61	1.96	1.87	2.14	2.18		^37^
*B*	2.97	2.02	1.90	2.22	2.26	–0.10	^a^
base-on, low-spin Ni(II) synthetic complexes (X-ray)							
**2**·CH_2_Cl_2_	3.00	1.92/1.92		2.20/2.20		+0.01	^a^
**4** (red)	2.94	1.91/1.92		2.18/2.19		–0.04	^b^
base-on, low-spin Ni(II) model (DFT)							
*2*	3.06	1.94/1.94		2.24/2.24		+0.05	^a^
base-on, high-spin Ni(II) hook (DFT)							
Ni^II^-on-hs	1.96	2.16	2.01	2.29	2.25	+0.24	^37^
4	2.06	2.12	2.03	2.60	2.37	+0.31	^30^
*A*	2.06	2.16	2.07	2.35	2.50	+0.21	^a^
base-on, high-spin Ni(II) synthetic complex (X-ray)							
**1** (Ni1, 293 K)	2.05	2.06/2.11		2.34/2.39		+0.30	^a^
base-on, high-spin Ni(II) model (DFT)							
*3*	2.03	2.09/2.09		2.42/2.42		+0.30	^a^

a This work.

b Figure S37 in Supporting Information.

An overlay plot of the oxidized nickel hook model *D* with the base-on conformation of NiSOD (1Q0D.pdb)^19,20^ was not so good (Figure 19, left). As just noted, the axial Ni–N bond lengths in the calculated models are much shorter than those observed in the crystal structures, and there is a clear disparity in displacement of the nickel ion from the equatorial ligand plane, which is reflected in the differing *trans* N–Ni–S bond angles. In fact, the elongated square-pyramidal Ni­(II) model *B* affords a better overlay with base-on NiSOD (Figure 19, right). The equatorial bond lengths are similar, and the nickel ion is disposed close to the equatorial plane. However, the axial Ni···N distances calculated in models *B* and *2*, and those observed in synthetic complexes **2** and red **4**, are consistently longer (Table 6) than those observed experimentally for base-on NiSOD.^17–21^ On the other hand, an axial Ni···N distance of only 2.61 Å was extrapolated for the low-spin limit of **1**,^54^ and a corresponding distance of 2.389(2) Å was observed experimentally in the diamagnetic Ni­(II) complex [(Tp^Me,Me^)­Ni­(CN)_2_]^+^.^75^

**Figure 19:**
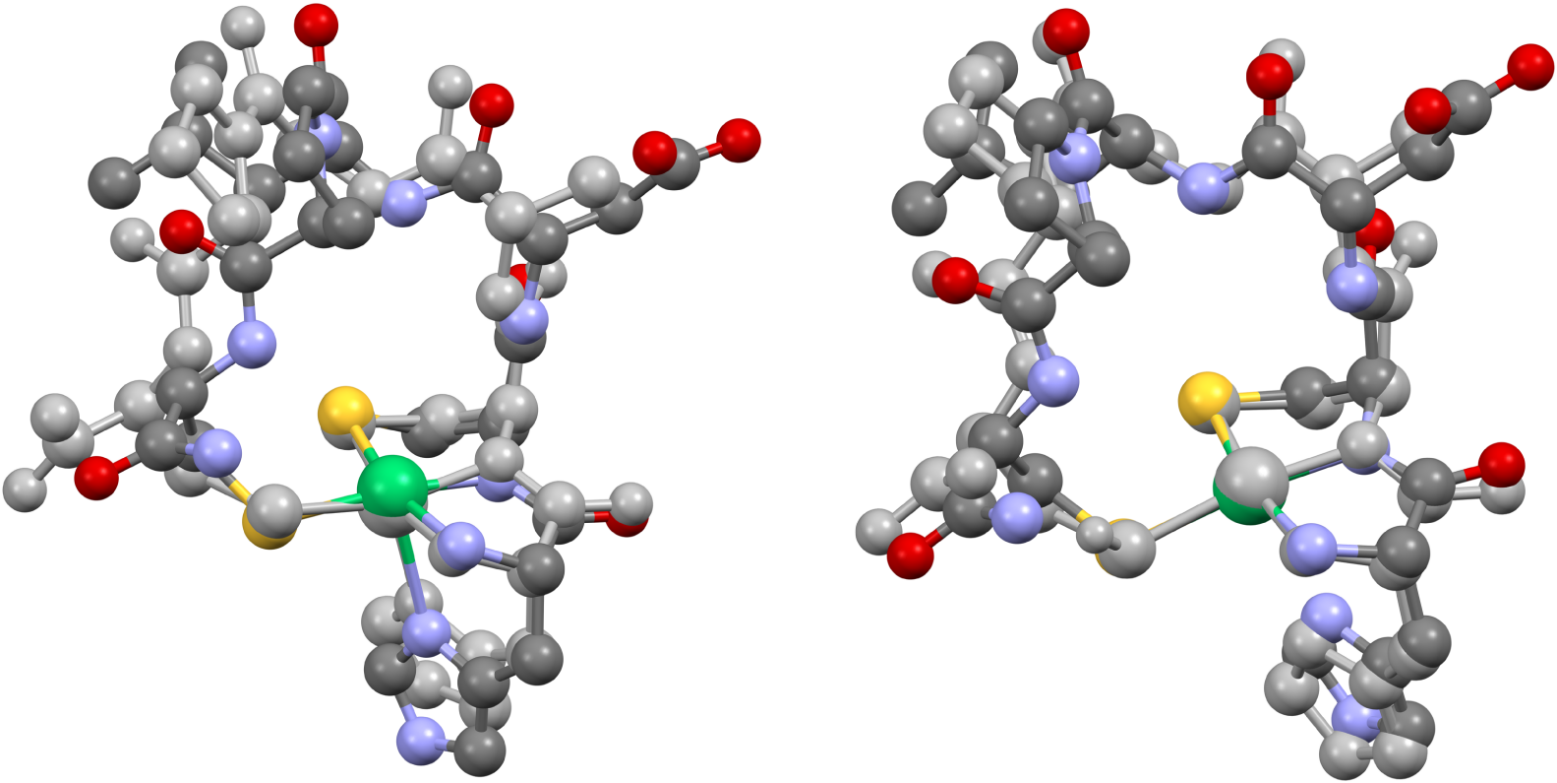
Least-squares overlay of the equatorial donor atoms of the experimental NiSOD base-on conformation (1Q0D.pdb, in color)^19,20^ with the Ni­(III) model *D* (rendered in gray, left), and with the base-on, low-spin Ni­(II) model *B* (rendered in gray, right). Hydrogen atoms are omitted for clarity, except for the protonated Cys-6 thiol of *B*.

Spin crossover of Ni­(II) is coupled to substantial axial contraction, from 3.06 to 2.03 Å as calculated for DFT models *2* and *3* (Table S25), and elongation of equatorial Ni–N and Ni–S bonds, from 1.94 to 2.09 Å and from 2.24 to 2.42 Å, respectively (Scheme 1). The C=N bond of the dithiocarbamate was lengthened slightly, from 1.33 to 1.35 Å, and the CS_2_ angle increased from 108° to 115° (Table S27). Finally, the Ni­(II) ion was displaced from the equatorial plane toward the axial donor by an additional 0.25 Å (Table S25). The metrics calculated for high-spin Ni­(II) were consistent with experimental values for the corresponding Ni1 site of green **1** (Tables 2 and 3), allowing for some trigonal distortion.

A role for high-spin Ni­(II) in the base-on NiSOD conformation can be excluded. An overlap plot of nickel hook model *A* shows a shorter axial Ni–N bond that again leads to displacement of Ni­(II) from the equatorial plane, and the equatorial bonds are elongated by the presence of an unpaired electron in the Ni­(II) 3 $d_{x^{2}‐y^{2}}$ orbital (Figure S78). Such features are observed in the distorted square-pyramidal synthetic complex **1**, and reproduced in *3* and related DFT models (Tables 6 and S56).^30,37^

**Table 7:** Comparison of Calculated Electronic Transitions in the Reduced, Base-Off Models *C* and *1* to Absorption Data for Reduced NiSOD

No.	cm^**–**1^	ε (M^**–**1^ cm^**–**1^)	assignment
NiSOD_red_ (observed)^a^			
A	17110	70	LF: d_*xz*/yz_ → $d_{x^{2}‐y^{2}}$
B	18430	150	LF: d_*xz*/yz_ → $d_{x^{2}‐y^{2}}$
C	20500	180	LF: $d_{z^{2}}$ → $d_{x^{2}‐y^{2}}$
D	22240	480	LF: d_*xy*_ → $d_{x^{2}‐y^{2}}$
E	24970	500	LMCT: S_σ/π_ → $d_{x^{2}‐y^{2}}$
F	27650	880	LMCT: S_σ/π_ → $d_{x^{2}‐y^{2}}$
G	29220	750	LMCT: S_σ/π_ → $d_{x^{2}‐y^{2}}$
model *C* (calculated)^b^			
1A	13950	50	LF: d_*xz*/yz_ → $d_{x^{2}‐y^{2}}$
2A	15060	20	LF: d_*xz*/yz_ → $d_{x^{2}‐y^{2}}$
3A	17850	40	LF: $d_{z^{2}}$ → $d_{x^{2}‐y^{2}}$
7A	22070	880	LF: d_*xy*_ → $d_{x^{2}‐y^{2}}$
9A	23340	80	LMCT: S_δ_ → $d_{x^{2}‐y^{2}}$
18A	25560	1740	LMCT: S_π_ → $d_{x^{2}‐y^{2}}$
34A	28500	2290	MLCT: d_*yz*_ → amide π*
40A	30630	5350	MLCT: $d_{z^{2}}$ → amide π*
41A	31440	1010	LMCT: 2S_σ_ → $d_{x^{2}‐y^{2}}$
44A	32030	4400	LMCT: 6S_σ_ → $d_{x^{2}‐y^{2}}$
model *1* (calculated)^c^			
1A	15990	30	LF: d_*xz*/yz_ → $d_{x^{2}‐y^{2}}$
2A	16990	10	LF: d_*xz*/yz_ → $d_{x^{2}‐y^{2}}$
3A	17760	40	LF: $d_{z^{2}}$ → $d_{x^{2}‐y^{2}}$
8A	23900	50	LMCT: S_δ_ 2a_2_ → $d_{x^{2}‐y^{2}}$
9A	24100	100	LF: d_*xy*_ → $d_{x^{2}‐y^{2}}$
10A	24850	5310	MLCT: d_*yz*_ → 4b_1_ π*
13A	27860	30	LMCT: S_π_ 3b_1_ → $d_{x^{2}‐y^{2}}$
16A	28540	870	MLCT: d_*yz*_ → pz π*
23A	31840	4260	MLCT: d_*yz*_ → pz π*
34A	36580	16380	LMCT: S_σ_ 6b_2_ → $d_{x^{2}‐y^{2}}$
59A	42790	5140	LMCT: S_σ_ 7a_1_ → $d_{x^{2}‐y^{2}}$

a Table 7 in ref 24.

b Table S15.

c Table S48.

#### 3.5.2 Electronic Configurations

Comparisons of calculated frontier molecular orbitals between the various synthetic and nickel hook models were also of interest. The classical (d_*xy*_)^2^ $\left(d_{z^{2}}\right)$ ^2^(d_*xz*_,d_*yz*_)^4^( $d_{x^{2}‐y^{2}}$ )^0^ ligand field splitting of a Ni­(II), d^8^ metal ion in a square-planar geometry was obtained for the base-off nickel hook model *C* (Figure 20). The topology and ordering of frontier orbitals for the corresponding synthetic model *1* were similar. The Ni 3d orbitals were stabilized in *1* relative to *C,* which may reflect a less electron-rich environment. The LUMO in both models was the Ni $3d_{x^{2}‐y^{2}}$ orbital with substantial Ni–S σ* covalency: 44% Ni 3d and 32% S 3p in *C* (113a); and 47 and 36%, respectively, in *1* (63a). Calculated HOMO–LUMO gaps were 1.3 eV in *C* and 1.6 eV in *1*. The nominally degenerate HOMOs, the Ni 3d_*xz*_ and 3d_*yz*_ orbitals, were split by ca. 0.15 eV. The orbitals in the model *C* (112a and 111a) exhibited four-electron π* overlap with Cys-6 and Cys-2 thiolates, respectively, and the former was further destabilized by overlap with the *trans* backbone amide. In contrast, the orbitals in *1* (62a and 60a) were split by distinctive δ* and π* overlap with the dithiocarbamate sulfur atoms. The coligand in *1* also affords a low-energy π* acceptor orbital (64a) that has no analogue in the nickel hook (relevant frontier orbitals of the free dithiocarbamate anion are listed in Table S18 and shown in Figure S14). These orbitals exhibited an average of 67% Ni 3d and 22% S 3p character in *C*, and 72% Ni 3d and 13% S 3p character in *1*. The Ni 3 $d_{z^{2}}$ orbital (110a in *C* and 61a in *1*) was disposed proximally in the absence of an axial interaction. The nonbonding Ni 3d_*xy*_ orbital (109a in *C* and 57a in *1*) was disposed 1.1 eV below the HOMO and 2.5 eV below the LUMO in *C*, compared to 1.3 and 2.8 eV, respectively, in *1*. Below that were sulfur π and σ donors, stabilized another ca. 0.4 and 1.2 eV, respectively, in *C*, compared to 0.0–0.5 eV and 0.6–1.7 eV, respectively, in *1*. The calculated gap between Ni 3d and S 3p orbitals in *1* is quantitatively consistent with photoelectron spectra of homoleptic, square-planar Ni­(II) dithiocarbamate complexes.^92,93^

**Figure 20:**
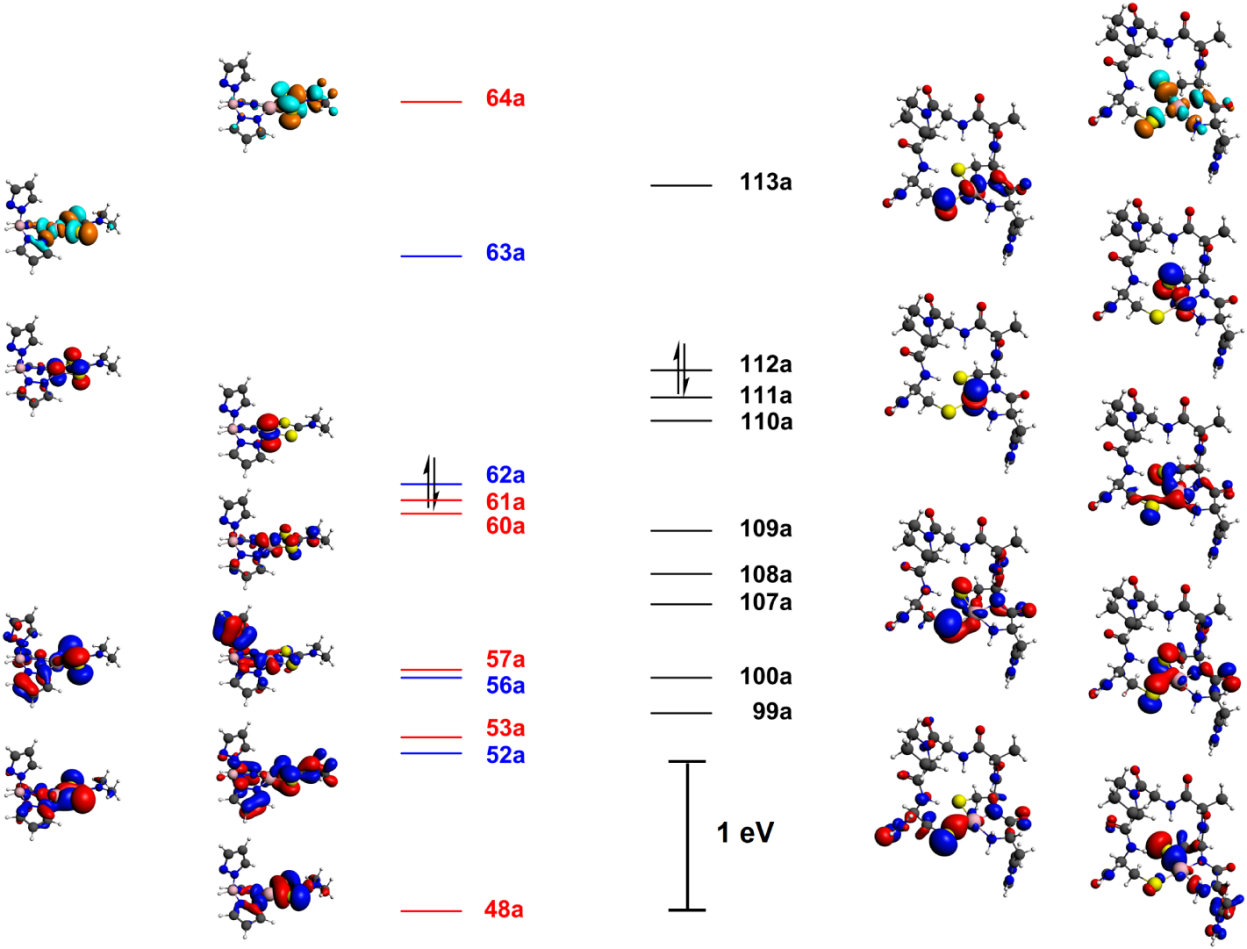
Comparison of select Ni- and S-centered frontier orbitals for the reduced, base-off nickel hook model *C* (right) and the synthetic analogue model *1* (left). Relative energies are indicated at center (those of *1* are color-coded by mirror symmetry), and isocontour plots are shown on the periphery (those of *1* are separated by mirror symmetry).

Weak axial association in the elongated square-pyramidal models *B* and *2* (Figures S6 and S21, respectively) destabilized the Ni 3 $d_{z^{2}}$ orbital relative to the base-off models *C* and *1*. The Ni 3 $d_{z^{2}}$ orbital was the HOMO in the resulting (d_*xy*_)^2^(d_*xz*_,d_*yz*_)^4^ $\left(d_{z^{2}}\right)$ ^2^( $d_{x^{2}‐y^{2}}$ )^0^ electron configuration, and should be preferred as the redox-active orbital. The HOMO in *B* exhibited 79% Ni 3d and 9% S 3p character, versus 86% and 0%, respectively, in *2*. The Cys-6 thiolate is protonated in *B*, and this resulted in a large stabilization of the corresponding S 3p_π_ donor orbital. As a result, the corresponding Ni 3d π* orbital (110a) shows a negligible contribution from sulfur (75% Ni 3d and 4% S 3p, Table S10).

The high-spin Ni­(II) models *A* and *3* were broadly similar (Figures S5 and S22, respectively). Axial compression was coupled to transfer of one electron from the low-spin HOMO into the LUMO (i.e., Ni 3 $d_{z^{2}}$ → 3 $d_{x^{2}‐y^{2}}$ , Scheme 1), with corresponding elongation of equatorial bonds. The high-spin state is not observed for NiSOD; as the resulting SOMOs exhibit substantial Ni–S covalency, this may not be advantageous with respect to metal-centered redox reactivity. The hydrogen bond to the Cys-6 thiolate in *A* appears to have a negligible electronic effect.

Frontier orbitals for the oxidized nickel hook model *D* and the analogous model *6* are similar (Figure S79). The Ni 3 $d_{x^{2}‐y^{2}}$ orbital was unoccupied in both models. As for high-spin Ni­(II), the Ni 3 $d_{z^{2}}$ orbital was destabilized by an axial Ni–N bond and remained as the SOMO in both models (112a and 37a′, respectively). This orbital occupancy is consistent with formulation of legitimate low-spin (*S* = 1/2) d^7^ Ni­(III) centers. The 3d orbitals were stabilized by the higher oxidation state; this increases Ni–S covalency generally, and the filled Ni 3d orbitals fell below the S 3p π/δ donor orbitals. The total ligand field splitting for Ni­(III) in *D*, 3.5 and 3.0 eV for the respective α- and β-spin orbitals, is larger than for Ni­(II), 2.5 eV in *B*; analogous values of 3.5 and 3.6 eV in *6* compare with 2.7 eV in *2*.

Additional calculations were performed on trigonal-bipyramidal complex models *4*, *5*, and *7* with low-spin Ni­(II), high-spin Ni­(II), and Ni­(III), respectively (Figures S23, S24 and S26). As noted previously, the HOMO–LUMO gap in *4* was unrealistically small. The frontier orbitals of *5* (Table S43) were dispersed similarly to square-pyramidal *3*; analogously, the orbital dispersion in oxidized model *7* (Table S45) was similar to that of *6*. However, the SOMOs in this geometry feature strong donation from sulfur, such that ligand-centered oxidation may be problematic. A trigonal-bipyramidal geometry is circumvented for NiSOD by rigid chelation through the peptide backbone and constraint associated with the cysteine-2 side chain.

The Ni­(II) square-planar model *C*_s_-[(Bp)­NiS_2_CNMe_2_] (*8*; Table S46 and Figure S27) was essentially equivalent to the base-off model *1*. On the other hand, the oxidized, square-planar model *9* (Table S47 and Figure S28) was distinct from square-pyramidal *6*. In the absence of an axial base, the Ni 3 $d_{z^{2}}$ orbital in *9* dropped below the SOMO (β-22a″). The latter exhibited substantial Ni–S δ* covalency (44% Ni 3d and 37% S 3p), as well as a significant contribution from pyrazolyl ring π orbitals. Mulliken spin density on nickel was diminished in *9* compared with *6* (0.48 vs 0.76, respectively; Table S29), while that on the sulfur atoms increased (+0.19 vs −0.03, respectively). The calculations call into question the redox innocence of supporting ligation in *9*, and serve again to underscore a plausible role for the axial base in maintaining metal-centered oxidation.^24,37^

#### 3.5.3 TD-DFT Calculations

Given the analogous electronic configurations (Figure 20), calculated electronic spectra of the base-off, reduced nickel hook model *C* (Figure S11 and Table S15) and its analogue *1* (Figure S29 and Table S48) exhibited some similarities (Figure 21 and Table 7). These can be compared directly to the reported spectrum of reduced NiSOD (cf., Figure 2 and Table 7 of Fiedler et al.).^24^ The latter included four bands with ligand field character, observed between 17,110 and 22,240 cm^–1^, and three stronger bands with S 3p → Ni 3d LMCT character observed between 24,970 and 29,220 cm^–1^. The calculated spectrum of *C* was consistent with the experimental spectrum, except that predicted ligand field bands extended to lower energies and the CT extinctions were too high. Four ligand field bands were calculated for *C* between 14,000 and 22,000 cm^–1^, and four additional S 3p → Ni 3d LMCT bands were calculated for *C* between 23,300 and 32,000 cm^–1^. Intense MLCT transitions from Ni­(II) into the backbone amide π* orbital were calculated at 28,500 and 30,600 cm^–1^. The elongated square-pyramidal model *B* (Figure S10 and Table S14) was somewhat different. The proton shift from the axial His-1 base in *C* to Cys-6 in *B* suppressed the contributions of the latter; associated LMCT bands were blue-shifted and reduced in intensity compared with *C*.

**Figure 21:**
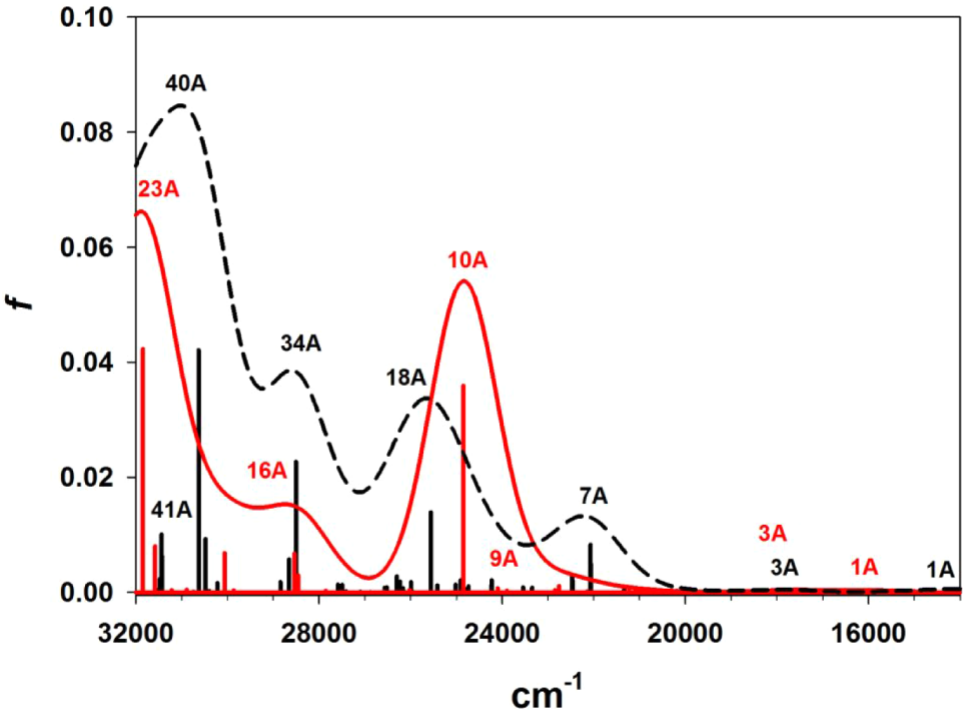
Calculated electronic spectra of the reduced, base-off nickel hook model *C* (dashed black line) and the synthetic analogue model *1* (solid red line), rendered with arbitrary line widths of 1700 cm^–1^. Select transitions are compared in Table 7; all calculated transitions are listed in Tables S15 and S48, respectively.

Calculations on the base-off Ni­(II) model *1* gave four ligand field bands between 16,000 and 24,000 cm^–1^. The S 3p π/δ donor orbitals supported weak LMCT bands at 24,000 and 28,000 cm^–1^, and the S 3p σ orbitals gave rise to much stronger transitions at 36,600 and 42,800 cm^–1^. Calculated LF and LMCT transitions were slightly blue-shifted compared with *C*, which is consistent with relative stabilization of the sulfur donor orbitals within the dithiocarbamate ligand, and the larger calculated ligand field splitting. MLCT transitions into the pyrazole and dithiocarbamate π* orbitals were calculated at intermediate energies for *1*, but these have no counterparts within the nickel hook. Calculated spectra of the elongated square-pyramidal *2* (Table S49 and Figure S30), which lacks a proton, and square-planar model *8* (Table S54 and Figure S35), which lacks an axial base, were nearly identical to that of *1*. The experimental spectrum of the base-free complex **5** can be compared to the calculated spectrum of the analogous model *8* (Figure S80 and Table S57).

Calculated spectra for the neutral oxidized nickel hook model *D* (Figure S12 and Table S16) can be compared (Table 8) to experimental UV–vis spectrum of as-isolated NiSOD,^11,12,24^ which is dominated by the oxidized component (cf., Figure 1 and Table 7 in Fiedler et al.).^24^ There are three caveats. First, energies for this open-shell model exhibit systematic red-shifting; for example, a prominent band observed at 26,850 cm^–1^ appears to have a counterpart in band 38A, which is calculated at 21,960 cm^–1^ (i.e., a red shift of 0.6 eV). Second, the high oxidation state stabilizes filled Ni 3d π orbitals and enhances Ni–S covalency (vide supra); accordingly, a plethora of bands with admixed ligand field and charge transfer character was calculated for *D*, and interpretation of these is not straightforward. Third, the low-energy hole in the 3 $d_{z^{2}}$ orbital of Ni­(III) provides a second acceptor orbital for two NIR CT transitions, but spectra for NiSOD in aqueous solution only extend to ca. 12,500 cm^–1^.^11,12,24^ The calculated spectrum of the cationic model *6* (Figure S33 and Table S52) is similar to that of *D* (Figure 22), albeit with generally blue-shifted transitions and somewhat lower CT extinctions. The lack of axial His-1 and backbone amide π donors in *6* also obviates some corresponding LMCT bands (Table 8).

**Figure 22:**
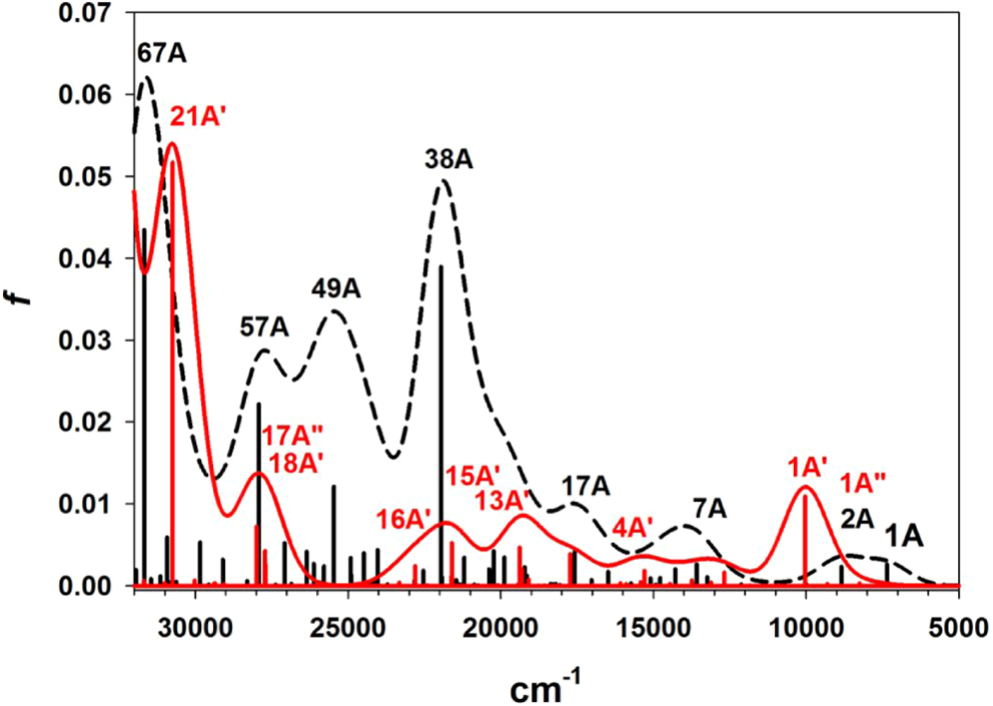
Calculated electronic spectra of the oxidized nickel hook model *D* (dashed black line) and the synthetic analogue model *6* (solid red line), rendered with arbitrary line widths of 1700 cm^–1^. Select transitions are compared in Table 8; all calculated transitions are listed in Tables S16 and S52, respectively.

**Table 8:** Comparison of Calculated Electronic Transitions in the Ni­(III) Models *D* and *6* to Absorption Data for As-Isolated NiSOD

No.	cm^**–**1^	ε (M^**–**1^ cm^**–**1^)	assignment
NiSOD_ox_ (observed)^a^			
1	14380	300	LF: $d_{z^{2}}$ → $d_{x^{2}‐y^{2}}$
2	15650	510	LMCT: S/N/O π → d_σ_
3	18000	1340	LMCT: S/N/O π → d_σ_
4	19920	1510	LMCT: S/N/O π → d_σ_
5	23290	2020	LMCT: S/N/O π → d_σ_
6	24700	3080	LMCT: N/O_σ_ → $d_{x^{2}‐y^{2}}$
7	26850	6800	LMCT: 6S_σ_ → $d_{x^{2}‐y^{2}}$
8	28720	4020	LMCT: 2S_σ_ → $d_{x^{2}‐y^{2}}$
9	30400	4900	
10	31950	4740	
model *D* (calculated)^b^			
1A	7350	270	LMCT: Sp_δ_* → $d_{z^{2}}$
2A	8850	320	LMCT: Sp_π_* → $d_{z^{2}}$
5A	12130	30	LF: $d_{z^{2}}$ → $d_{x^{2}‐y^{2}}$
7A	13580	370	LMCT: 1-His π → $d_{z^{2}}$
11A	15100	100	LMCT: Sp_π_* → $d_{x^{2}‐y^{2}}$
17A	17580	710	LF: d_*yz*_ → $d_{z^{2}}$
20A	18200	30	LF: d_*xy*_ → $d_{z^{2}}$
24A	19150	240	LF: d_*xz*_ → $d_{z^{2}}$
38A	21960	4000	LF: d_*xy*_ → $d_{x^{2}‐y^{2}}$
			LMCT: im π → $d_{z^{2}}$
			LMCT: 6S_σ_ → $d_{x^{2}‐y^{2}}$
39A	22540	300	LF: d_*xz*_ → $d_{x^{2}‐y^{2}}$
49A	25460	1830	LF: d_*xy*_ → $d_{x^{2}‐y^{2}}$
			LMCT: 2S_σ_ → $d_{x^{2}‐y^{2}}$
57A	27920	2220	LF: d_*xz*_ → $d_{x^{2}‐y^{2}}$
67A	31670	4750	LMCT: 1-His π → $d_{x^{2}‐y^{2}}$
model *6* (calculated)^c^			
1A″	8250	60	LMCT: Sδ 2a_2_ → $d_{z^{2}}$
1A′	10030	1180	LMCT: Sπ 3b_1_ → $d_{z^{2}}$
2A′	11850	10	LMCT: Sδ 2a_2_ → $d_{x^{2}‐y^{2}}$
5A″	13110	70	LF: $d_{z^{2}}$ → $d_{x^{2}‐y^{2}}$
8A″	16060	20	LMCT: Sπ 3b_1_ → $d_{x^{2}‐y^{2}}$
11A″	19320	230	LMCT: Sσ 6b_2_ → $d_{z^{2}}$
13A′	19390	490	LF: d_*xz*_ → $d_{z^{2}}$
15A′	21610	650	LMCT: Sσ 6b_2_ → $d_{x^{2}‐y^{2}}$
14A″	22400	10	LF: d_*yz*_ → $d_{z^{2}}$
16A′	22800	290	LF: d_*xy*_ → $d_{z^{2}}$
18A′	26990	430	LMCT: Sσ 7a_1_ → $d_{z^{2}}$
17A″	28000	940	LF: d_*xz*_ → $d_{x^{2}‐y^{2}}$
18A″	29350	40	LF: d_*xy*_ → $d_{x^{2}‐y^{2}}$
20A′	30420	100	MCLT: $d_{z^{2}}$ → 4b_1_ π*
21A′	30740	5170	LF: d_*yz*_ → $d_{x^{2}‐y^{2}}$
			LMCT: Sσ 6b_2_ → $d_{x^{2}‐y^{2}}$
21A″	31000	70	LMCT: Sσ 7a_1_ → $d_{x^{2}‐y^{2}}$

a Table 7 in ref^24^.

b Table S16.

c Table S52.

TD-DFT calculations were also performed for high-spin nickel hook model *A* (Figure S9 and Table S13), square-pyramidal analogue *3* (Figure S31 and Table S50) and trigonal-bipyramidal analogue *5* (Figure S32 and Table S51). Compared with low-spin Ni­(II) models, the ligand field is weakened by occupation of the Ni 3 $d_{x^{2}‐y^{2}}$ orbital, and ligand field bands are shifted to lower energies, even allowing for systematic red-shifting of open-shell models. As for the Ni­(III) complexes, the vacancy in the β-spin Ni 3 $d_{z^{2}}$ orbital increases the number of charge transfer and ligand field bands. There are no experimental data against which the calculated spectrum of *A* can be evaluated. That of model *3* appears to match the experimental spectrum of complex **1** reasonably well (Figure S81 and Table S58), allowing for the red-shifting and some exaggerated extinctions. Calculated spectra of models *3* and *5* are quite similar (Figure S82).

#### 3.5.4 Frequency Calculations

Frequency calculations were conducted on the *N*-dimethylcarbamate anion (i.e., model *0*, Table S20), as well as the complex models *1*–*9* (Table 9). The nickel hook models were omitted, given computational demands resulting from the relatively large number of atoms and lack of symmetry. Of particular interest was the ν­(B–H) mode of the Tp ligand, core modes of the dithiocarbamate ligand and proximal *N*-Me deformations, as well as any imaginary frequencies.

**Table 9:** Calculated Frequencies (cm^–1^) and Intensities of Select Vibrational Modes for DFT Models *0–9*

Model	ν(B–H)	ν(C=N)	δ(Me)	ν_as_(N–Me_2_)	ρ(Me)	ν_as_(CS_2_)	imaginary
*0*	—	1317 (179)	1473 (110)	1245 (69)	1112 (28)	949 (184)	–12 (0)
*1*	2513 (81)	1520 (273)	1394 (139)	1224 (32)	1135 (251)	973 (20)	–21 (−2)
*2*	2502 (97)	1512 (245)	1391 (162)	1229 (33)	1132 (208)	978 (12)	
*3*	2519 (93)	1498 (133)	1377 (144)	1221 (39)	1116 (296)	958 (57)	
*4*	2512 (100)	1511 (248)	1389 (105)	1228 (33)	1129 (282)	977 (11)	–85 (−7)
*5*	2517 (94)	1498 (136)	1375 (134)	1219 (39)	1114 (292)	960 (54)	–32 (0)
*6*	2573 (55)	1569 (356)	1401 (55)	1208 (16)	1148 (165)	944 (3)	
*7*	2571 (56)	1573 (354)	1397 (48)	1206 (16)	1145 (165)	945 (6)	–57 (0)
*8*	2577 (146)^a^	1555 (392)	1394 (76)	1205 (31)	1141 (203)	946 (16)	–22 (1)
	2378 (121)^b^						
*9*	2512 (74)^a^	1592 (514)	1403 (69)	1202 (24)	1155 (152)	903 (115)	–207 (−591)
	2409 (74)^b^						

a ν_as_(BH_2_).

b ν_s_(BH_2_).

Reasonable agreement was obtained between calculations herein on the free anion and the previous normal coordinate analysis on the potassium salt.^81^ The frequency calculation on model *0* (Table S20) returned five relatively intense fingerprint modes (Table 9) that compare to the prominent bands in the IR spectrum of the salt.^81^ The greatest ν­(C=N) amplitude was exhibited by the calculated 5A_1_ mode at 1317 cm^–1^, but significant couplings to the 3A_1_ mode at 1473 cm^–1^ and the 6A_1_ mode at 1112 cm^–1^, of respective δ­(Me) and ρ­(Me) character, were predicted. The 5A_1_ mode also exhibited some ν_s_(N–Me_2_) character, which otherwise dominates the comparatively weak 7A_1_ mode at 858 cm^–1^. The largest ν_as_(N–Me_2_) amplitude was evident in the 5B_2_ mode calculated at 1245 cm^–1^, although strong admixture with the flanking 6B_2_ mode of ρ­(Me) character at 1029 cm^–1^ was predicted. The 7B_2_ mode at 949 cm^–1^ showed overwhelming ν_as_(CS_2_) character, although minor coupling into 5B_1_ was evident. In view of the couplings predicted here and previously,^81^ some need for caution in the interpretation of observed fingerprint bands is evident.

A frequency calculation for the high-spin model *3* returned five analogous fingerprint modes (Table 9); however, predominant δ­(Me) and ν­(C=N) character was transposed between the highest two modes compared with model *0*. This calculation can be compared to the experimental spectrum of **1**, which exhibits fingerprint bands at 1508, 1392, 1257, 1153, and 985 cm^–1^ (Figure 11). The calculated ν­(C=N)/δ­(Me) modes red-shifted modestly from 1512/1391 cm^–1^ for low-spin *2* to 1498/1377 cm^–1^ for high-spin *3*. In contrast, the ν­(B–H) mode of the Tp ligand weakly blue-shifted from low- to high-spin Ni­(II), which is consistent with the change between κ^2^-/κ^3^-chelation.^57^ Small red shifts were indicated for the ν_as_(N–Me_2_) and ν_as_(CS_2_) modes. Calculated frequencies for the fingerprint modes of high-spin, square-pyramidal *3* and trigonal-bipyramidal *5* were nearly identical.

Compared with the Ni­(II) models, the oxidized models *6* and *7* were calculated with strongly blue-shifted ν­(B–H) and ν­(C=N) modes, and weakly red-shifted ν_as_(N–Me_2_) and ν_as_(CS_2_) modes. No significant shifts were predicted between the square-pyramidal and trigonal-bipyramidal geometries. On the other hand, removal of the axial base in model *9* induced a further blue shift in the ν­(C=N) mode and a red shift in the ν_as_(CS_2_) mode.

The free dithiocarbamate salt model *0* showed a single imaginary mode involving concerted rotations of the *N*–Me substituents. Model *1* exhibited a single weak imaginary mode that entailed rotation of the detached pyrazole arm toward the axial position on nickel. Trigonal-bipyramidal models *4*, *5*, and *7* each showed a weak imaginary mode involving counter-rotation of the coligands toward square-pyramidal. No imaginary frequencies were found for the models *2*, *3*, or *6*. Taken together, these imaginary modes support a square-pyramidal geometry as the global minimum for both spin states of Ni­(II) and for both oxidation states. The [(Bp)­NiS_2_CNMe_2_] model *8* gave a weak imaginary mode similar to that of model *0* that has no effect on the planar ligand field geometry. However, the corresponding oxidized model *9* showed an unusually strong imaginary mode that entailed asymmetric distortion of the pyrazolyl rings, which further underscores the possibility of redox noninnocence in the supporting coligands in the absence of an axial base.

## 4 Summary and Conclusions

This study reported syntheses and characterizations of [(Tp^Ph,Me^)­NiS_2_CNR_2_] complexes as a structural and spectroscopic model for the reduced state of NiSOD. Syntheses and characterizations of these model complexes were tractable. Experimental and theoretical approaches were combined to characterize the geometric and electronic structures of the synthetic complexes and to compare these aspects to those of the reduced enzyme. Key observations and conclusions are summarized.

The combination of hydrotris­(pyrazolyl)­borate and dithiocarbamate coligands replicates, to a first approximation, the equatorial N_2_S_2_ donor set of NiSOD. The scorpionate ligand also affords a fifth potential donor, a detachable pyrazolyl base that mimics the electronic properties and variable coordination of the His-1 imidazole side chain within the nickel hook. Four ligand field geometries were identified by X-ray crystallography, specifically square-planar, elongated square-pyramidal, compressed square-pyramidal and trigonal-bipyramidal. The first two support a red, diamagnetic complex of Ni­(II), and the latter two support a green, paramagnetic complex of Ni­(II). These were further differentiated by distinct resonance structures within the zwitterionic dithiocarbamate coligand.

The model complexes lack a counterpart to the backbone amide donor, and the pair of cysteine thiolates found in the nickel hook are stronger donors than the zwitterionic dithiocarbamate coligand in our model complexes. Hence, our models manifest a weak ligand field relative to NiSOD, and we obtained a mixture of high- and low-spin Ni­(II) isomers, rather than the low-spin Ni­(II) and Ni­(III) obtained in the isolated enzyme. Aqueous solubility is precluded, and the nonbiological pyrazolate–boron linkage within the scorpionate ligand is subject to nucleophilic displacement, although an unexpected benefit in the latter case was the isolation of a rigorously κ^2^-scorpionate ligand derivative without an axial donor.

Notwithstanding the chemical approximations, the base-off isomers of our model complexes exhibit equatorial coordinate bond lengths coincident with those observed for the nickel hook. DFT and TD-DFT calculations on the base-off nickel hook and the analogous [(Tp)­NiS_2_CNMe_2_] model suggest comparable electronic structures. Further calculations on the experimentally established low-spin, base-on conformation support previous hypotheses that a weak axial base interaction is necessary and sufficient to promote the Ni 3 $d_{z^{2}}$ as the redox-active molecular orbital and to circumvent sulfur-centered oxidation.

The observed and calculated geometric structures lead us to suggest that the base-on conformation revealed in the X-ray crystal structures of NiSOD is better fit by Ni­(II) than by Ni­(III). The oxidized state should exhibit a short axial Ni–N bond and significant displacement of the nickel ion from the N_2_S_2_ equatorial plane, yet neither feature is evident in the enzymatic crystal structures. On the other hand, we observed an elongated square-pyramidal conformation in X-ray structures of three diamagnetic Ni­(II) complexes, including **1**, **2**, and **4**, and relevant structural features were reproduced in simplified DFT models of the nickel hook and our synthetic complexes. This hypothesis is constrained by the lack of unambiguous structural data for an axially ligated Ni­(III) model complex (and perhaps for NiSOD itself). Synthesis of such a complex is the focus of our continuing research effort.

## Supplementary Material







## Notes

Details of the DFT calculations, details of the X-ray crystallography, miscellaneous structural analyses, and all spectroscopic and characterization data (PDF)
Cartesian coordinates of optimized DFT nickel hook structures (XYZ)
Cartesian coordinates of optimized DFT model structures (XYZ)

## #

Institute of Advanced Materials, School of Flexible Electronics (Future Technologies), Nanjing Tech University, Nanjing 211816, People’s Republic of China

## ⊥

Marshall School, 1215 Rice Lake Road, Duluth, MN 55811, United States of America.
