# Supplementary material for: Scorpionate Complexes [(κ n ‑TpPh,Me)NiS2CNR2] (n = 2, 3) as a Structural and Spectroscopic Model for Reduced Nickel-Dependent Superoxide Dismutase
Source: Inorg Chem. 2026 Feb 11;65(7):4149–73. doi: 10.1021/acs.inorgchem.5c05781 (PMC12933891; doi:10.1021/acs.inorgchem.5c05781)
Supplement: Supplementary file 1 [file ic5c05781_si_001.pdf]

Supporting Information

Scorpionate complexes [(κ^n-Tp^{Ph,Me})NiS$_2$CNR$_2$] (n = 2, 3) as a structural and spectroscopic model for reduced nickel-dependent superoxide dismutase

Huaibo Ma,[a] Haoshuang Wang,[a] Javad Shokraiyan,[a] Jeffrey L. Petersen,[b]
Gregory T. Rohde,[c] Victor G. Young, Jr.[c] and Michael P. Jensen[a,*]

(a) Department of Chemistry & Biochemistry, Ohio University, Athens, OH 45701 UNITED STATES

(b) C. Eugene Bennett Department of Chemistry, West Virginia University, Morgantown, WV 26506 UNITED STATES

(c) X-ray Crystallographic Facility, Department of Chemistry, University of Minnesota, Minneapolis, MN 55455 UNITED STATES

* email: jensenm@ohio.edu

2.3. DFT calculations. Geometry optimizations were performed using the Amsterdam Density Functional 2008.01 software suite,[67,68] with the Vosko–Wilk–Nusair LDA functional,[94] the BP86 GGA correction,[95,96] the Slater-type TZP orbital basis set (with frozen atomic cores),[97] and default convergence parameters. A solvent continuum model (COSMO) consistent with acetonitrile was also applied.[98] Single-point calculations on the optimized geometries were repeated using the OPBE and the hybrid B3LYP* (i.e., 15% Hartree–Fock) functionals.[99,100] TD-DFT and frequency calculations were performed on selected models (vide infra).[101–103]

Simplified computational models of the reduced nickel hook structure were constructed from the first nickel site listed in the published crystal structure of NiSOD (1T6U.pdb):[17,18] the backbone sequence was cleaved after the sixth residue as an amidium cation, –C(O)NH_3^+; the base-off conformation was deleted; the D3 carboxylate was replaced by hydrogen (i.e., D3A, known to be a conservative mutation); the ^{i}Bu group of L4 was replaced by hydrogen (i.e., L4G); and remaining hydrogen atoms were added into ideal positions (Scheme S1 in Supporting Information). This gave a model of neutral charge with Ni(II) and 224 valence electrons, H_2N–HCAGPC–C(O)NH_3•Ni ($C_{22}H_{33}N_9NiO_6S_2$, 642.38 g/mol). The initial model was optimized in a high-spin state (model *A*), and then re-optimized in a low-spin state (*B*). The 6-amidium proton moved into a hydrogen-bonded position with the cysteine-6 thiolate sulfur atom in *A*, and was fully transferred to give a thiol and free amide in *B*. The axial His-1 donor ring in model *B* was rotated away from nickel, and the labile proton was moved onto N_ε, and the resulting base-off conformation was re-optimized (*C*). The proton and one electron were deleted from *B* and the oxidized state was re-optimized (*D*). Owing to residual atom motion within the unrestrained span of the nickel hook backbone, full convergence was not achieved; the gradients invariably converged, but not the Cartesian atom displacements (which typically varied by 0.02 Å on average and by 0.06 Å maximum). Therefore, the geometry optimizations were run through 60 cycles until the change in energy diminished to ca. 0.02 eV; single-point calculations were then performed, which converged normally.

The orientation of Cartesian axes is given in Scheme S2. The model energies, calculated bond lengths and angles, and Mulliken charges and spin densities of nickel and the donor atoms are given in Tables S1–S4.[67,68] Optimized structures, rendered with *Mercury*,[69] are shown in Figures S1–S4, and atomic coordinates are given in Tables S5–S8. Relative energies and proposed assignments of select frontier orbitals are given in Tables S9–S12 and illustrated in Figures S5–S8. Electronic spectra calculated by TD-DFT methods are shown in Figures S9–S12, and the calculated transitions are assigned and listed in Tables S13–S16.

A model of *exo(H,H)*-Me_2NCS_2Na with ideal C_{2v} symmetry (model *0*) was constructed with the assistance of the "symmetrize" command available in the ADF graphical interface.[67,68] The structure, rendered with Mercury,[69] is shown in Figure S13, and the atomic coordinates are given in Table S17. The relative energies and compositions of select frontier molecular orbitals for the free anion are tabulated in Table S18 and shown in Figure S14. The calculated electronic spectrum of the free anion, which was obtained by TD-DFT methods, is shown in Figure S15, and relevant transitions are tabulated in Table S19. A complete list of normal modes with calculated energies and intensities is given in Table S20.

DFT models of [(Tp)NiS_2CNMe_2], of neutral charge and 124 valence electrons with Ni(II), $C_{12}H_{16}BN_7NiS_2$ (391.94 g/mol) were constructed from an X-ray crystal structure of [($Tp^{Me,Me}$)NiS_2CNMe_2] by replacement of the 3- and 5-pyrazole methyl substituents with hydrogen atoms. Ideal symmetry was obtained using the "symmetrize" command available in the graphical interface of the ADF software suite.[67,68] Seven independent models with the following sets of oxidation state, applied symmetry, geometry and spin states were calculated: reduced, C_1, square-planar with detached axial base, S = 0 (model *1*); reduced, C_s, axially elongated square-pyramidal, S = 0 (model *2*); reduced, C_s, axially compressed square-pyramidal, S = 1 (*3*); reduced, C_s, axially compressed trigonal-bipyramidal, S = 0 (*4*); reduced, C_s, axially elongated trigonal-bipyramidal, S = 1 (*5*); oxidized, C_s, square-pyramidal, S = ½ (*6*); and oxidized, C_s, trigonal-bipyramidal, S = ½ (*7*). The axial pyrazole rings of *2* and *6* were each replaced by a hydrogen

atom, and the resulting $[(Bp)NiS_2CNMe_2]^{n+}$ models, Bp = dihydrobis(pyrazol-1-yl)borate, were re-optimized to give models *8* and *9* (*n* = 0 and 1, respectively). Relative model energies are presented in Tables S21–S24, calculated bond lengths and angles are shown in Tables S25–S28, and Mulliken charges and spin densities are given in Table S29.[67,68] Model structures, rendered using Mercury,[69] are presented in Figures S16–S19. Optimized atomic coordinates are given in Tables S30–S38. Relative energies and compositions of select frontier orbitals are listed in Tables S39–S47 and plotted in Figures S20–S28. Electronic spectra calculated by TD-DFT methods for models *1*–*3* and *5*–*8* are shown in Figures S29–S35; calculated transitions are assigned and listed in Tables S48–S54 in Supporting Information.

2.4. X-ray crystallography. The X-ray crystal structures of $[(Tp^{Ph,Me})NiS_2CNR_2]$ (R = Me, **1**; Et, **2**; and Ph, **3**), $[(Tp^{Ph,Me})NiS_2CNC_{12}H_8]$ (**4**), $[(HB\{pz^{Ph,Me}\}_2\{OC(O)CF_3\})NiS_2CNPh_2]$ (**5**), and $[(Tp^{Ph,Me})Ni(Hpz^{Ph,Me})(OC\{O\}CF_3)]$ (**6**) were performed by Prof. Jeffrey L. Petersen at West Virginia University. A suitable crystal was selected from each sample, washed with perfluoropolyether PFO-XR75 (Lancaster), and sealed in a glass capillary under a nitrogen atmosphere. Each sample was aligned on a Siemens P4 diffractometer equipped with a Mo Kα radiation source (λ = 0.71073 Å), a graphite monochromator, a monocap collimator, and a SMART CCD detector. Data were collected at room temperature, 293(2) K. The program SMART was used for diffractometer control, frame scans, indexing, orientation matrix calculations, least-squares refinement of unit cell parameters, and the data collection.[104] Raw data frames were read by SAINT and integrated using 3d profiling algorithms.[105] A semi-empirical absorption correction was applied using the SADABS routine available in SAINT.[106,107] All data were corrected for Lorentz and polarization effects. Data preparation was carried out using XPREP.[106,107] The structures were solved by a combination of direct methods and difference Fourier techniques with the use of SHELXTL.[106,107] Hydrogen atoms were included as fixed contributions, using a riding model with isotropic temperature factors set at 1.2 (on an aromatic carbon or boron) or 1.5 (aliphatic carbon) times the adjacent heavy atom. The positions of methyl hydrogen atoms were

optimized by a rigid rotating group refinement with idealized tetrahedral angles. No correction for secondary extinction was applied. The linear absorption coefficient, atomic scattering factors, and anomalous dispersion corrections were calculated from values found in the International Tables of X-ray Crystallography.[108]

In the structure of **1**, the C5–C10 phenyl ring exhibited a two-site rotational disorder; this was refined by constraining the C8•••C8′ distance within 0.02 Å. In the structure of **2**, one of the ethyl groups suffered from a two-site conformational disorder with equivalent populations; the C–C and C–N bond lengths were constrained to within 0.02 Å of 1.54 and 1.45 Å, respectively. In the structure of **3**, the C38–C43 phenyl ring on the dithiocarbamate ligand suffered from a two-site conformational disorder; the C–C and C–N distances were constrained to within 0.02 Å of 1.39 and 1.45 Å, respectively. In the structure of **5**, the trifluoromethyl substituent suffered from a two-site conformational disorder; the C–F bond lengths were constrained within 0.01 Å of 1.33 Å during refinement. The trifluoromethyl group in the structure of **6** exhibited a two-site, Star-of-David disorder in a 9:1 ratio; the fluorine atoms of the minor conformation were refined with isotropic temperature parameters.

Additional crystal structures of **1**, **2**, and **3**, and of $[(\mu\text{-pz}^{Ph,Me})NiS_2CNMe_2]_2$ (**7**) were obtained by Dr. Victor G. Young, Jr. at the University of Minnesota. Suitable crystals were selected and placed onto the tips of 0.1 mm diameter glass capillaries and mounted on a CCD area detector diffractometer for data collection at 123(2) or 173(2) K using MoKα radiation with a graphite monochromator. The intensity data were corrected for absorption and decay using SADABS.[109] Final cell constants were calculated from strong reflections from the actual data collection after integration using SAINT.[105] The structures were solved by direct methods and refined using Bruker SHELXTL.[106,107] Non-hydrogen atoms were refined with anisotropic displacement parameters. All hydrogen atoms were placed in ideal positions and refined as riding atoms with relative isotropic displacement parameters. A dichloromethane solvent molecule

found in the lattice of **2** was disordered over at least two sites; this was removed with the use of PLATON/SQUEEZE.[110] The packing of **7** forms two infinite channels parallel to the *a* axis that are filled randomly with solvent molecules, including a site partially occupied with CH_2Cl_2. The channels comprise 733 $Å^3$, or 21% of the unit cell volume. This volume could accommodate ca. 40 non-hydrogen atoms, or ca. 10 for each complex molecule. This rough estimate seems consistent with the mixture of CH_2Cl_2 and diethyl ether from which crystals of **7** were grown. The disordered inclusions were removed using PLATON/SQUEEZE.[110]

The structure of a solvated pseudopolymorph of **4** was determined by Dr. Gregory T. Rohde at the University of Minnesota. A suitable crystal was placed onto the tip of 0.1 mm diameter glass capillary and mounted on a CCD area detector diffractometer using MoKα radiation with a graphite monochromator. The intensity data, collected at 173(2) K, were corrected for absorption and decay using SADABS.[109] Final cell constants were calculated from strong reflections from the actual data collection after integration using SAINT.[105] The structure was solved by direct methods using SIR2004,[111] and refined using SHELXL-97.[106] All hydrogen atoms were placed in ideal positions and refined as riding atoms with relative isotropic displacement parameters, except for the borohydride atom that was refined isotropically. The lattice was found to host one equivalent of an ordered dichloromethane molecule. Badly disordered and fractionally occupied hexane was also present, which obliged removal using PLATON/SQUEEZE.[110] Removal of the inclusion left artificial voids totaling 816 $Å^3$, or 17% of the total cell volume; this would correspond to ca. two hexane molecules per complex molecule at full occupancy. The final *R* value was unusually high. The results are provided as Supporting Information only (Figure S37); particular caution is urged with regard to interpretation of this structure.

Additional references

(94) Vosko, S. H.; Wilk, L.; Nusair, M. Accurate spin-dependent electron liquid correlation energies for local spin density calculations: a critical analysis. *Can. J. Phys*. **1980**, *58* (8), 1200–1211. DOI: 10.1139/p80-159

(95) Becke, A. D. Density-functional exchange-energy approximation with correct asymptotic behavior. *Phys. Rev. A: At., Mol., Opt. Phys*. **1988**, *38* (6), 3098–3100. DOI: 10.1103/PhysRevA.38.3098

(96) Perdew, J. P. Density-functional approximation for the correlation energy of the inhomogeneous electron gas. *Phys. Rev. B: Condens. Matter Mater. Phys*. **1986**, *33* (12), 8822–8824. DOI: 10.1103/PhysRevB.33.8822

(97) van Lenthe, E.; Baerends, E. J. Optimized Slater-type basis sets for the elements 1–118. *J. Comput. Chem.* **2003**, *24* (9), 1142–1156. DOI: 10.1002/jcc.10255

(98) Pye, C. C.; Ziegler, T.; van Lenthe, E.; Louwen, J. N. An implementation of the conductor-like screening model of solvation within the Amsterdam density functional package – Part II. COSMO for real solvents. *Can. J. Chem*. **2009**, *87* (7), 790–797. DOI: 10.1139/V09-008

(99) Swart, M.; Ehlers, A. W.; Lammertsma, K. Performance of the OPBE exchange-correlation functional. *Mol. Phys*. **2004**, *102* (23-24), 2467–2474. DOI: 10.1080/0026897042000275017

(100) Stephens, P. J.; Devlin, F. J.; Chabalowski, C. F.; Frisch, M. J. *Ab Initio* Calculation of Vibrational Absorption and Circular Dichroism Spectra Using Density Functional Force Fields. *J. Phys. Chem*. **1994**, *98* (45), 11623–11627. DOI: 10.1021/j100096a001

(101) van Gisbergen, S. J. A.; Snijders J. G.; Baerends E. J., Implementation of time-dependent density functional response equations. *Comput. Phys. Commun*. **1999**, *118* (2-3), 119–138. DOI: 10.1016/S0010-4655(99)00187-3

(102) Wang, F.; Ziegler, T. Excitation energies of some d^1 systems calculated using time-dependent density functional theory: an implementation of open-shell TDDFT theory for doublet–doublet excitations. *Mol. Phys*. **2004**, *102* (23-24), 2585–2595. DOI: 10.1080/0026897042000275080

(103) Wolff, S. K. Analytical Second Derivatives in the Amsterdam Density Functional Package. *Int. J. Quantum Chem*. **2005**, *104* (5), 645–659. DOI: 10.1002/qua.20653

(104) *SMART*, version 5.6; Bruker Analytical X-ray Systems: Madison, WI, 2001.

(105) *SAINT*; Bruker Analytical X-ray Systems: Madison, WI, 2004.

(106) *SHELXTL*, version 6.1; Bruker Analytical X-ray Systems: Madison, WI, 2000.

(107) Sheldrick, G. M. A short history of SHELX. *Acta Crystallogr.* **2008**, *A64* (1), 112-122. DOI: 10.1107/S0108767307043930

(108) *International Tables for X-ray Crystallography*, Vol. 4; Kynoch Press, 1974; p 55.

(109) Blessing, R. H. An empirical correction for absorption anisotropy. *Acta Crystallogr*. **1995**, *A51* (1), 33-38. DOI: 10.1107/S0108767394005726

(110) Spek, A. L. PLATON, An Integrated Tool for the Analysis of the Results of a Single Crystal Structure Determination. *Acta Crystallogr.* **1990**, *A46* (s1), c34. DOI: 10.1107/S0108767390099780

(111) Burla, M. C.; Camalli, M.; Carrozzini, B.; Cascarano, G. L.; Giacovazzo, C.; Polidori, G.; Spagna, R. *SIR2002*: the program. *J. Appl. Crystallogr*. **2003**, *36*, 1103. DOI: 10.1107/S0021889803012585

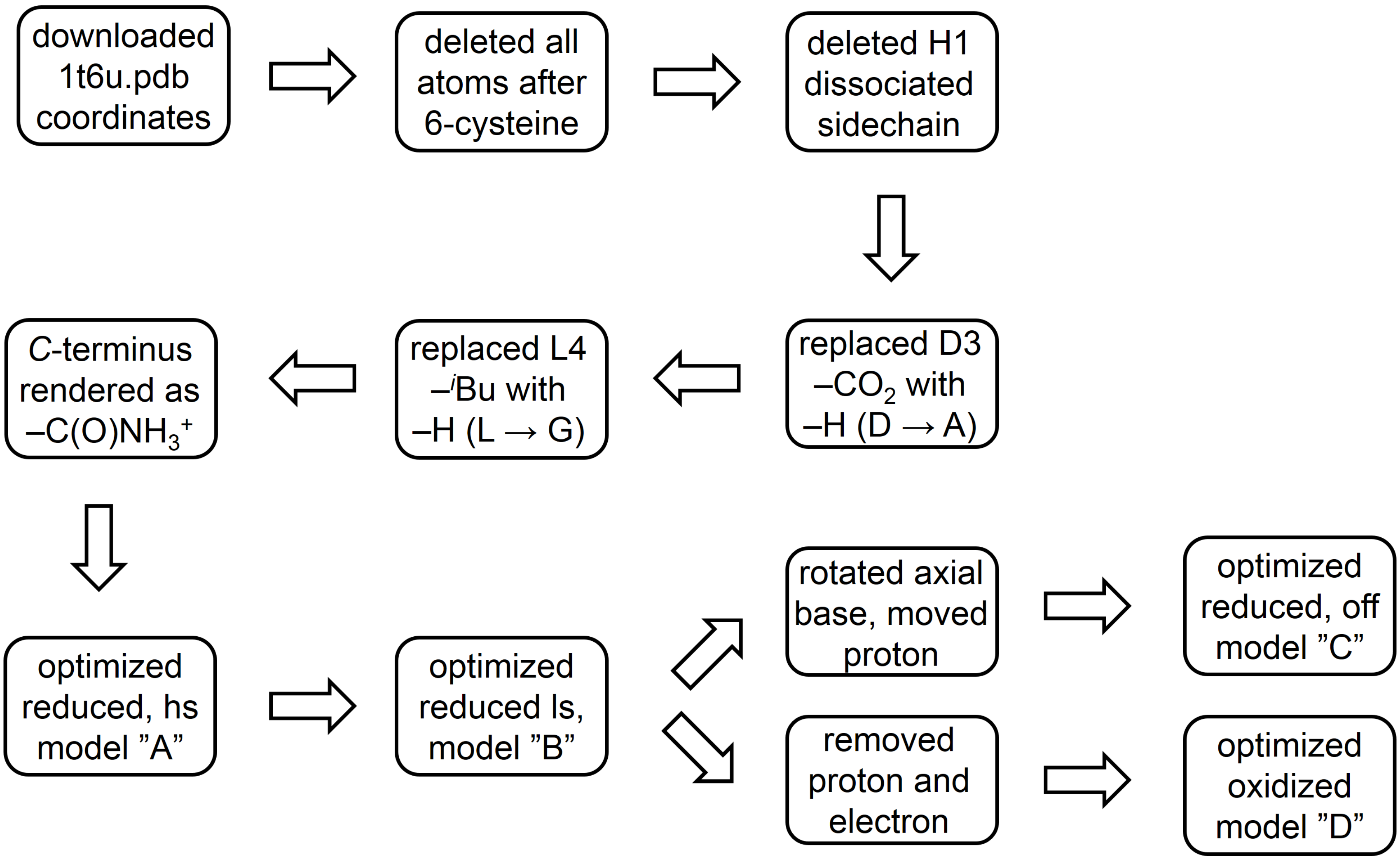

Scheme S1. DFT model building of the modified nickel hook structures.

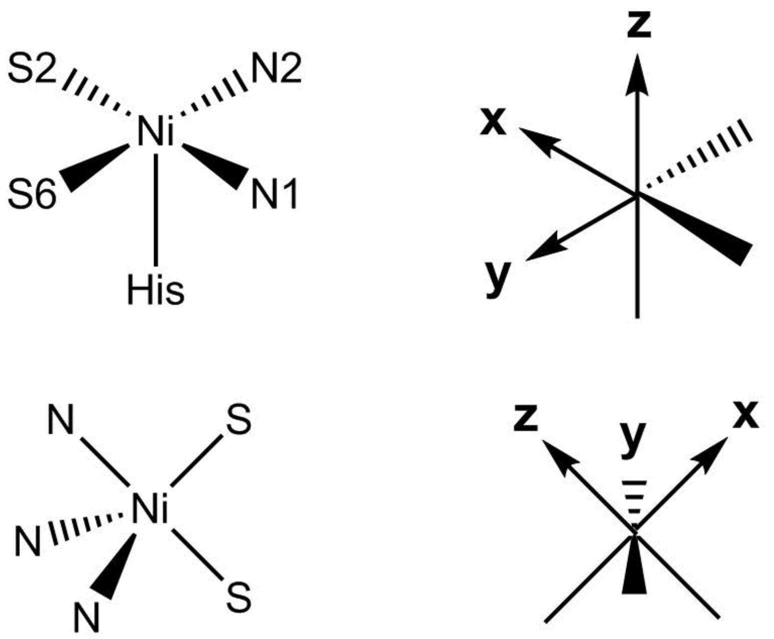

Scheme S2. Orientation of the Cartesian axes for square-pyramidal (top) and trigonal-bipyramidal (bottom) geometries, which are consistent with limiting C_{4v} and D_{3h} symmetries, respectively. Atom labels in the former correspond to residue numbering of NiSOD.

Table S1. Summary of modified nickel hook models.

Model	Ox. state	Spin state	Relative energy (eV)[(a)]	Axial His-1 base	disposition of added proton equivalent
A	Ni^{II}	*S* = 1	1.3/1.4/0.9	on, short	on C-terminal amide N, H-bonded to Cys-6
B	Ni^{II}	*S* = 0	0.4/0.5/0.4	on, long	on Cys-6 thiol
C	Ni^{II}	*S* = 0	0.0/0.0/0.0	off, protonated	on detached His-1 sidechain
D	Ni^{III}	*S* = ½		on, short	none

(a) BP86/OPBE/B3LYP* functionals.

Table S2. Calculated bond distances (Å) in nickel hook models.

Model	$Ni–N1_{ax}$	$Ni–N1_{eq}$	Ni–N2	Ni–S2	Ni–S6	$Ni\cdots N_2S_2$ [(a)]
A	2.06	2.16	2.07	2.35	2.50	+0.21
B	2.97	2.02	1.90	2.22	2.26	−0.10
C		2.05	1.93	2.21	2.25	−0.03
D	2.10	2.04	1.97	2.22	2.27	+0.31

(a) displacement of nickel from a least-squares N_2S_2 equatorial plane, with positive values defined toward the axial donor.

Table S3. Calculated bond angles (°) in nickel hook models.

Model	$N1_{ax}–Ni–N1_{eq}$	$N1_{ax}–Ni–N2$	$N1_{ax}–Ni–S2$	$N1_{ax}–Ni–S6$	$N1_{eq}–Ni–N2$	$N1_{eq}–Ni–S2$	$N1_{eq}–Ni–S6$	N2–Ni–S2	N2–Ni–S6	S2–Ni–S6
A	89.1	89.5	105.7	96.1	78.1	157.7	100.2	85.3	174.1	94.8
B	73.9	85.1	104.9	85.2	83.7	171.8	93.6	88.1	170.3	94.4
C					81.2	168.9	96.4	87.8	175.7	94.7
D	88.8	89.0	99.9	112.8	81.6	165.3	92.3	86.7	157.4	95.2

Table S4. Calculated Mulliken charges and spin densities in nickel hook models.

Model	Ni		$N1_{ax}$		$N1_{eq}$		N2		S2		S6	
	Z	*S*	Z	*S*	Z	*S*	Z	*S*	Z	*S*	Z	*S*
A	0.40	1.39	−0.35	0.10	−0.21	0.07	−0.49	0.11	−0.34	0.21	−0.16	0.07
B	0.14	0.00	−0.38	0.00	−0.24	0.00	−0.49	0.00	−0.18	0.00	0.41	0.00
C	0.03	0.00	0.04	0.00	−0.26	0.00	−0.50	0.00	−0.18	0.00	−0.33	0.00
D	0.26	0.72	−0.34	0.13	−0.19	0.01	−0.47	0.04	−0.07	0.01	−0.17	0.06

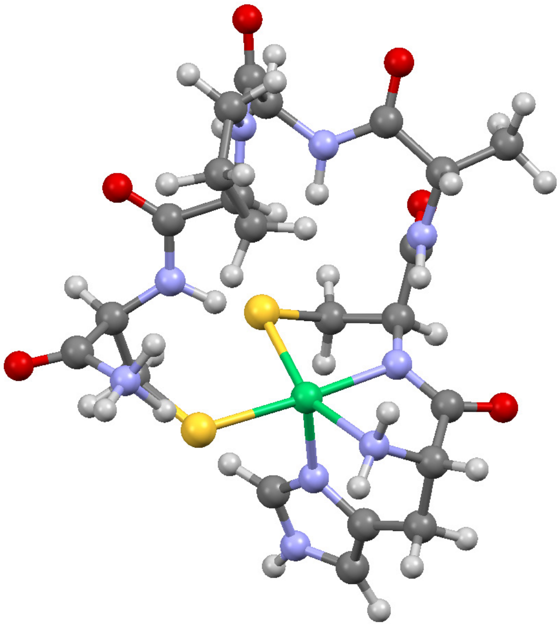

Figure S1. Optimized structure of the nickel hook model *A* with high-spin Ni^{II} (S = 1). Note the hydrogen bond between an elongated N–H bond of the protonated amide and the Cys-6 thiolate sulfur.

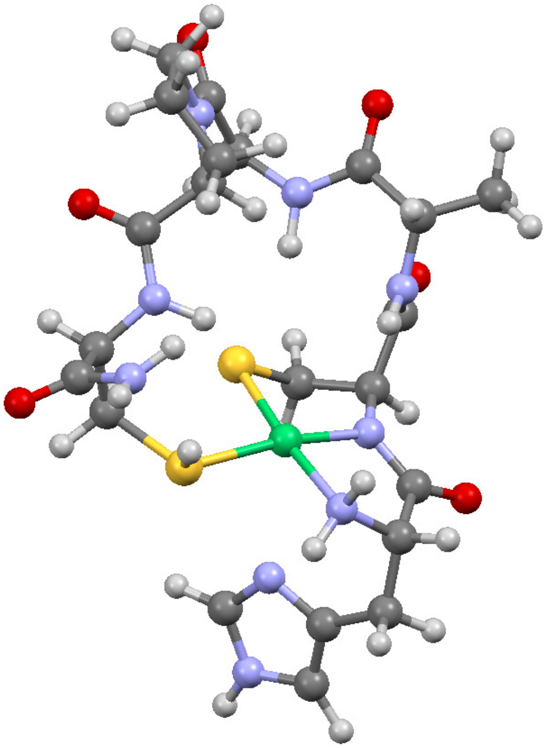

Figure S2. Optimized structure of the nickel hook model *B* with low-spin Ni^{II} (S = 0). Note the protonated Cys-6 thiol.

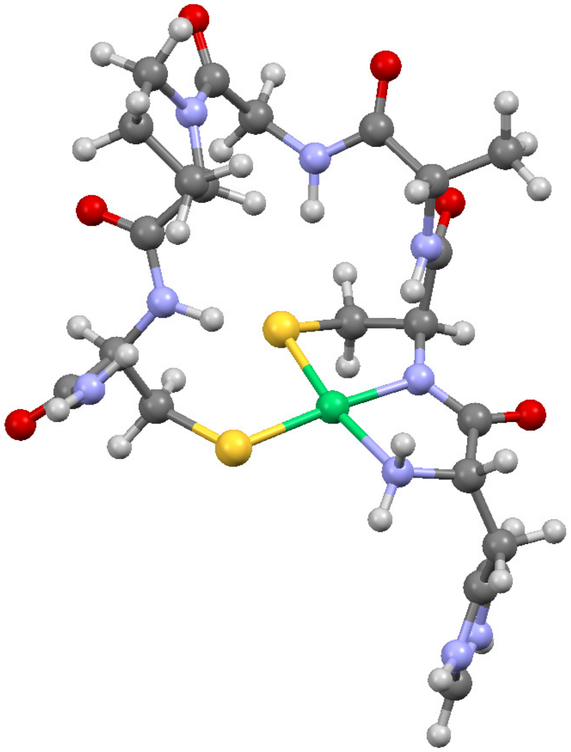

Figure S3. Optimized structure of the nickel hook model *C* with low-spin Ni(II) (S = 0). Note the protonated Nε of the His-1 imidazole.

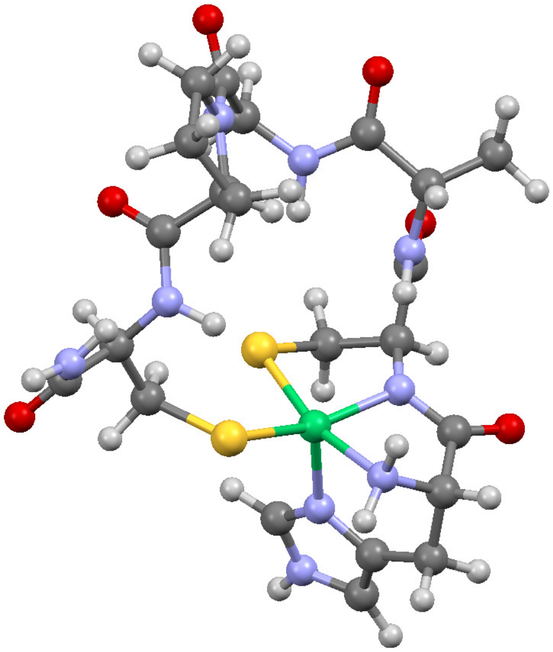

Figure S4. Optimized structure of the nickel hook model *D* with low-spin Ni(III).

Table S5. Optimized atomic coordinates for the nickel hook model *A.*

	X	Y	Z
N	0.15595381	-0.54156282	2.08681046
C	0.19453674	0.76292340	2.79817064
C	-0.76365849	1.78522976	2.15289767
O	-1.03101538	2.82583079	2.81817948
C	1.62450770	1.33800922	2.77956769
C	2.17459975	1.68296975	1.41345998
N	1.66650952	1.18883805	0.21651474
C	3.24407248	2.50073514	1.11198064
C	2.39949428	1.69628206	-0.77080145
N	3.36476273	2.49258089	-0.26151341
N	-1.13922245	1.46508817	0.90767460
C	-1.78692658	2.46803311	0.06446262
C	-3.30473379	2.30836510	-0.06264206
O	-3.98001010	3.13789072	-0.69784660
C	-1.18751219	2.43340181	-1.35234396
S	-1.02521125	0.70399431	-1.98821979
N	-3.80930260	1.19246846	0.51651444
C	-5.22720535	0.85517842	0.60602581
C	-5.78151898	0.28914640	-0.71685251
O	-7.00485304	0.22891728	-0.90059905
C	-6.12067242	1.96773522	1.16918309
H	-7.10516077	1.55119760	1.40841947
N	-4.87410597	-0.20930133	-1.59698455
C	-5.28966334	-0.91745249	-2.80476419
C	-5.85829237	-2.34299808	-2.60554228
O	-6.99811401	-2.59295350	-3.04603859
H	-4.44029110	-0.94596780	-3.49871292
N	-5.10640742	-3.27629986	-1.96516576
C	-3.71415105	-3.11542084	-1.54084687
C	-2.71421758	-3.30352458	-2.71138711
O	-2.96775183	-3.93250602	-3.74270183
C	-3.55386776	-4.22069088	-0.46829494
C	-4.45565340	-5.35667851	-0.97863398
C	-5.63129204	-4.63392280	-1.65845228
N	-1.48571107	-2.78910930	-2.43918963
C	-0.29972474	-2.97896021	-3.26440821
C	0.36578859	-4.32894141	-3.05110843
O	1.02971687	-4.91598412	-3.87671408
C	0.79104662	-1.94016455	-2.85436916
S	1.18640551	-1.93521466	-1.04853694
N	0.37404371	-4.67649433	-1.63429907
Ni	0.00000000	0.00000000	0.00000000
H	2.25572179	1.51299097	-1.82773613
H	3.91232440	3.08060581	1.73620793
H	-0.10139721	0.64844755	3.85066941
H	2.29119254	0.62614236	3.29157350
H	1.61554147	2.24678030	3.39555509

Table S5. continued.

	X	Y	Z
H	-0.68201073	-1.05766282	2.36556531
H	0.95252178	-1.11255850	2.38100063
H	1.69857165	-2.15727790	-3.42901938
H	0.43234480	-0.93600810	-3.12173257
H	-0.52794332	-2.87145262	-4.32941616
H	-3.55526657	-2.12679939	-1.09325007
H	-3.91762068	-3.82981999	0.49210903
H	-2.51076224	-4.52906023	-0.31841922
H	-4.78795853	-6.01325564	-0.16618450
H	-3.91338298	-5.97311125	-1.70760962
H	-5.96056342	-5.13144206	-2.57810327
H	-6.49883966	-4.53877722	-0.99089668
H	-6.10666086	-0.37056731	-3.28370141
H	-1.63848512	3.48008163	0.47672641
H	-0.18748882	2.88479128	-1.34116071
H	-1.81713555	3.01356637	-2.03657688
H	-5.28096747	-0.00027861	1.29612619
H	-6.24423748	2.77978150	0.44546158
H	-5.67851038	2.37100754	2.08874258
H	-0.57232413	-4.80374826	-1.26530964
H	0.79037415	-3.62414061	-1.08355475
H	-1.37139826	-2.14209549	-1.65039233
H	-3.88143641	-0.01195667	-1.43900399
H	-3.12496473	0.66541552	1.06343638
H	4.04759546	3.01014337	-0.80504568
H	0.93443801	-5.50094250	-1.41381336

Table S6. Optimized atomic coordinates for the nickel hook model *B.*

	X	Y	Z
N	0.15595381	-0.54156282	2.08681046
C	0.19453674	0.76292340	2.79817064
C	-0.76365849	1.78522976	2.15289767
O	-1.03101538	2.82583079	2.81817948
C	1.62450770	1.33800922	2.77956769
C	2.17459975	1.68296975	1.41345998
N	1.66650952	1.18883805	0.21651474
C	3.24407248	2.50073514	1.11198064
C	2.39949428	1.69628206	-0.77080145
N	3.36476273	2.49258089	-0.26151341
N	-1.13922245	1.46508817	0.90767460
C	-1.78692658	2.46803311	0.06446262
C	-3.30473379	2.30836510	-0.06264206
O	-3.98001010	3.13789072	-0.69784660
C	-1.18751219	2.43340181	-1.35234396
S	-1.02521125	0.70399431	-1.98821979
N	-3.80930260	1.19246846	0.51651444
C	-5.22720535	0.85517842	0.60602581
C	-5.78151898	0.28914640	-0.71685251
O	-7.00485304	0.22891728	-0.90059905
C	-6.12067242	1.96773522	1.16918309
H	-7.10516077	1.55119760	1.40841947
N	-4.87410597	-0.20930133	-1.59698455
C	-5.28966334	-0.91745249	-2.80476419
C	-5.85829237	-2.34299808	-2.60554228
O	-6.99811401	-2.59295350	-3.04603859
H	-4.44029110	-0.94596780	-3.49871292
N	-5.10640742	-3.27629986	-1.96516576
C	-3.71415105	-3.11542084	-1.54084687
C	-2.71421758	-3.30352458	-2.71138711
O	-2.96775183	-3.93250602	-3.74270183
C	-3.55386776	-4.22069088	-0.46829494
C	-4.45565340	-5.35667851	-0.97863398
C	-5.63129204	-4.63392280	-1.65845228
N	-1.48571107	-2.78910930	-2.43918963
C	-0.29972474	-2.97896021	-3.26440821
C	0.36578859	-4.32894141	-3.05110843
O	1.02971687	-4.91598412	-3.87671408
C	0.79104662	-1.94016455	-2.85436916
S	1.18640551	-1.93521466	-1.04853694
N	0.37404371	-4.67649433	-1.63429907
Ni	0.00000000	0.00000000	0.00000000
H	2.25572179	1.51299097	-1.82773613
H	3.91232440	3.08060581	1.73620793
H	-0.10139721	0.64844755	3.85066941
H	2.29119254	0.62614236	3.29157350
H	1.61554147	2.24678030	3.39555509

Table S6. continued.

	X	Y	Z
H	-0.68201073	-1.05766282	2.36556531
H	0.95252178	-1.11255850	2.38100063
H	1.69857165	-2.15727790	-3.42901938
H	0.43234480	-0.93600810	-3.12173257
H	-0.52794332	-2.87145262	-4.32941616
H	-3.55526657	-2.12679939	-1.09325007
H	-3.91762068	-3.82981999	0.49210903
H	-2.51076224	-4.52906023	-0.31841922
H	-4.78795853	-6.01325564	-0.16618450
H	-3.91338298	-5.97311125	-1.70760962
H	-5.96056342	-5.13144206	-2.57810327
H	-6.49883966	-4.53877722	-0.99089668
H	-6.10666086	-0.37056731	-3.28370141
H	-1.63848512	3.48008163	0.47672641
H	-0.18748882	2.88479128	-1.34116071
H	-1.81713555	3.01356637	-2.03657688
H	-5.28096747	-0.00027861	1.29612619
H	-6.24423748	2.77978150	0.44546158
H	-5.67851038	2.37100754	2.08874258
H	-0.57232413	-4.80374826	-1.26530964
H	0.79037415	-3.62414061	-1.08355475
H	-1.37139826	-2.14209549	-1.65039233
H	-3.88143641	-0.01195667	-1.43900399
H	-3.12496473	0.66541552	1.06343638
H	4.04759546	3.01014337	-0.80504568
H	0.93443801	-5.50094250	-1.41381336

Table S7. Optimized atomic coordinates for the nickel hook model *C.*

	X	Y	Z
N	0.91403195	0.01367913	1.83567715
C	0.83464654	1.35833451	2.51041322
C	-0.11720740	2.26705484	1.71813751
O	-0.31306644	3.44938565	2.11311180
C	2.12653114	2.10885618	2.95448421
C	3.10871982	2.83750286	2.07136130
N	4.46702808	2.52241721	2.00812547
C	2.98591276	4.01709911	1.37464497
C	5.13883252	3.45229112	1.31569696
N	4.24714700	4.36912833	0.93582291
N	-0.70889532	1.65970756	0.67049640
C	-1.65356452	2.44633549	-0.12864987
C	-3.12415793	2.13367118	0.18267427
O	-4.03473181	2.86764255	-0.24236602
C	-1.39321009	2.16209830	-1.60600901
S	-1.18339141	0.33553553	-1.83284686
N	-3.35089656	0.99177450	0.87105160
C	-4.69627100	0.48888865	1.14568740
C	-5.45308294	0.19226933	-0.18004044
O	-6.68681136	0.27650340	-0.26977621
C	-5.47530171	1.35377603	2.13742234
H	-6.47286289	0.93157955	2.30820298
N	-4.67625293	-0.28300532	-1.19110031
C	-5.23771751	-0.70231666	-2.46816581
C	-5.92057537	-2.08345076	-2.58000874
O	-6.99184019	-2.14871304	-3.22023237
H	-4.43070741	-0.67215644	-3.21276931
N	-5.36008489	-3.20066108	-2.05876082
C	-4.05860519	-3.36169978	-1.38897302
C	-2.90644488	-3.51022898	-2.41413357
O	-3.12081681	-3.85755675	-3.58805855
C	-4.25910721	-4.67496098	-0.59215287
C	-5.15883471	-5.52029017	-1.50826524
C	-6.08108185	-4.49628244	-2.18660103
N	-1.64803407	-3.32670351	-1.91270743
C	-0.46117258	-3.43441703	-2.74705553
C	0.28322878	-4.77452570	-2.64242326
O	1.11734423	-5.05166737	-3.52950307
C	0.51175630	-2.25520408	-2.51641636
S	0.98471877	-1.88077853	-0.75699280
N	0.03325199	-5.55537944	-1.57815715
Ni	0.00000000	0.00000000	0.00000000
H	6.20273035	3.47459483	1.12017069
H	2.11878660	4.63644739	1.18620416
H	0.36122707	1.19659616	3.49045580
H	2.69796258	1.45891601	3.63272078
H	1.70285716	2.90057279	3.59088176

Table S7. continued.

	X	Y	Z
H	0.29045087	-0.60787336	2.35811239
H	1.84516808	-0.40097958	1.93041106
H	1.42478053	-2.47017205	-3.08409045
H	0.05587560	-1.34509651	-2.92664064
H	-0.77997060	-3.40957821	-3.79664266
H	-3.84948631	-2.51753608	-0.72102274
H	-4.76936476	-4.43892417	0.35097764
H	-3.30717195	-5.16445945	-0.34977246
H	-5.72272362	-6.27661963	-0.94863240
H	-4.55297264	-6.03984595	-2.26194468
H	-6.26044448	-4.72342212	-3.24484049
H	-7.05788876	-4.40802197	-1.68973456
H	-6.01212939	0.00604216	-2.78095390
H	-1.53156665	3.51845691	0.07856012
H	-0.47517883	2.66857453	-1.92766193
H	-2.22454408	2.51109869	-2.23247947
H	-4.55868831	-0.49930431	1.61024646
H	-5.57900154	2.38166212	1.77088026
H	-4.93580429	1.37328442	3.09283986
H	-0.75089748	-5.34916037	-0.96551879
H	-1.49288513	-2.87344554	-1.00555049
H	-3.65232101	-0.17942720	-1.13121192
H	-2.51981212	0.45824024	1.13361801
H	4.47247823	5.20848045	0.40912448
H	0.45008732	-6.48187713	-1.53297135
H	4.89541792	1.67670177	2.37396291

Table S8. Atomic coordinates for the oxidized model *D* of the NiSOD hook.

	X	Y	Z
N	0.37483912	-0.45263071	1.95680086
C	0.39425768	0.85044871	2.67561896
C	-0.64983065	1.79971414	2.06214648
O	-0.96372816	2.83932409	2.68722586
C	1.79824316	1.48175421	2.57325958
C	2.20561460	1.86779915	1.17057475
N	1.64997289	1.29510744	0.03016741
C	3.13594398	2.80102665	0.76636079
C	2.21572990	1.87695955	-1.02638993
N	3.12241643	2.78753961	-0.61359763
N	-1.08481921	1.41799808	0.83704651
C	-1.84125519	2.37053613	0.00569744
C	-3.35992927	2.11651894	-0.08864324
O	-4.08006012	2.99861168	-0.58554229
C	-1.26042697	2.35441498	-1.41109118
S	-0.92792655	0.60775759	-1.92625969
N	-3.78696829	0.90863503	0.36177178
C	-5.18317247	0.47395731	0.50866617
C	-5.78055722	-0.10563001	-0.80305860
O	-7.00711241	-0.21135149	-0.95806924
C	-6.09162945	1.50240533	1.19564930
H	-7.09160314	1.07625890	1.33354471
N	-4.89433142	-0.59634798	-1.71314526
C	-5.34036024	-1.15429756	-2.98972032
C	-6.02377216	-2.54271472	-2.98365508
O	-7.08253123	-2.67165117	-3.63655113
H	-4.46895029	-1.20109336	-3.65498296
N	-5.47419768	-3.59675743	-2.32765989
C	-4.17597688	-3.65818357	-1.64393449
C	-3.00485166	-3.69376069	-2.66213958
O	-3.20206441	-3.73167285	-3.88171693
C	-4.29398729	-4.94495754	-0.79468414
C	-5.16699146	-5.86869833	-1.66206239
C	-6.15470744	-4.91778544	-2.35493507
N	-1.75007290	-3.67591711	-2.11780110
C	-0.55934710	-3.51679665	-2.94759750
C	0.25544648	-4.81251877	-3.17617058
O	1.28350727	-4.76371053	-3.87550612
C	0.35151251	-2.34165372	-2.50727352
S	0.54023994	-2.09479383	-0.68343531
N	-0.22199663	-5.94669116	-2.63496893
Ni	0.00000000	0.00000000	0.00000000
H	1.98366595	1.67285756	-2.06409325
H	3.78566410	3.45874255	1.32986110
H	0.15541686	0.71048738	3.73667009
H	2.52164752	0.77354143	3.00878981
H	1.81661657	2.37303350	3.21332530

Table S8. continued.

	X	Y	Z
H	-0.36869061	-1.05354648	2.32217515
H	1.25235533	-0.95780873	2.11088219
H	1.34561387	-2.51691892	-2.93104876
H	-0.03907303	-1.41153960	-2.93756812
H	-0.91990542	-3.26046637	-3.95401708
H	-4.04157585	-2.77861947	-0.99978137
H	-4.80371015	-4.70088077	0.14702704
H	-3.31560663	-5.37485956	-0.54754111
H	-5.68142332	-6.63136901	-1.06557429
H	-4.55224367	-6.38814664	-2.41030920
H	-6.37975569	-5.20405574	-3.38916271
H	-7.10764292	-4.84144996	-1.81096222
H	-6.08356011	-0.49386252	-3.44945632
H	-1.74698257	3.38103393	0.43183795
H	-0.32124918	2.91987023	-1.44278165
H	-1.95462544	2.79766157	-2.13662704
H	-5.13766074	-0.40342264	1.17206981
H	-6.17023222	2.42409935	0.60943717
H	-5.67777615	1.74834917	2.18181069
H	-1.09081042	-5.95269345	-2.11013237
H	-1.62682461	-3.44696948	-1.12633195
H	-3.90509600	-0.36674792	-1.59916720
H	-3.05257771	0.35222261	0.80167235
H	3.70450690	3.35379907	-1.22403857
H	0.21785671	-6.83450774	-2.86134652

Table S9. Frontier molecular orbitals for the nickel hook model *A* (Ni^{II}, *S* = 1).

**No.**	**E (eV)**	**Ni 3d (%)**	**S 3p (%)**	**assignment**	**Ni/S_2 overlap**
**α spin**					
122a	− 0.43	0	(22)	σ*(S–H)	
121a	− 0.55	0	3	1-amido π*	
120a	− 0.76	0	1	σ*(N–H)	
119a	− 0.89	0	1	1-His imidazole π*	
118a	− 1.01	0	0	2-amide π*	
117a	− 1.05	0	0	3-am π*	
116a	− 1.38	0	0	4-am π*	
115a	− 1.60	0	0	5-am π*	
114a	− 2.67	0	3	6-amide π* (α-spin LUMO)	
113a	− 4.22	30	40	Ni $3d_{x2-y2}$ (α-spin HOMO)	σ*
112a	− 4.98	14	69	Ni $3d_{xz}$	π* (S2)
111a	− 5.24	21	5	Ni $3d_{z^2}$	
110a	− 5.54	39	22	Ni $3d_{yz}$	π* (S6)
109a	− 5.78	19	12	amide CO lp	
108a	− 5.89	3	0	1-His imidazole π	
107a	− 5.96	24	19	1-amido π	
106a	− 6.08	2	3	am π	
105a	− 6.16	57	8	S2 $p_π$	π ($3d_{xz}$)
104a	− 6.19	45	12	Ni $3d_{xy}$	
103a	− 6.20	3	0	amide CO lp	
102a	− 6.26	0	0	4-am π	

Table S9. continued.

**No.**	**E (eV)**	**Ni 3d (%)**	**S 3p (%)**	**assignment**	**Ni/S_2 overlap**
101a	− 6.45	28	29	S6 p_π	π (d_{yz})
100a	− 6.65	14	12	amide CO lp	
99a	− 6.72	30	34	S2 p_σ	σ
98a	− 6.79	12	11	amide CO lp	
97a	− 6.87	13	12	S6 p_σ	σ
96a	− 6.91	3	2	amide CO lp	
95a	− 6.99	0	0	3-amide π	
94a	− 7.10	1	2	2-amide π	
**β spin**					
122a	− 0.40	0	(20)	σ*(S–H)	
121a	− 0.51	0	6	1-amido π*	
120a	− 0.70	0	1	σ*(N–H)	
119a	− 0.87	0	1	1-His imidazole π*	
118a	− 1.00	0	0	2-amide π*	
117a	− 1.05	0	0	3-amide π*	
116a	− 1.38	0	0	4-amide π*	
115a	− 1.59	0	0	5-amide π*	
114a	− 2.65	4	4	6-amide π*	
113a	− 2.82	54	19	Ni $3d_{x2-y2}$	σ*
112a	− 3.57	67	7	Ni $3d_{z}^{2}$ (β-spin LUMO)	
111a	− 4.76	37	45	Ni $3d_{xz}$ (β-spin HOMO)	π* (S2)
110a	− 5.01	65	8	Ni $3d_{yz}$	π* (S6)

Table S9. continued.

**No.**	**E (eV)**	**Ni 3d (%)**	**S 3p (%)**	**assignment**	**Ni/S_2 overlap**
109a	− 5.35	76	5	Ni $3d_{xy}$	
108a	− 5.53	38	36	S2 p_{π}	π (d_{xz})
107a	− 5.68	19	14	1-amido π	
106a	− 5.81	6	9	amide CO lp	
105a	− 5.90	0	2	1-His imidazole π	
104a	− 6.09	0	0	amide CO lp	
103a	− 6.19	9	16	amide CO lp	
102a	− 6.21	17	26	S6 p_{π}	π (d_{yz})
101a	− 6.25	2	4	4-amide π	
100a	− 6.27	19	43	S2 p_{σ}	σ
99a	− 6.59	4	48	S6 p_{σ}	σ
98a	− 6.61	3	0	amide CO lp	
97a	− 6.83	0	2	amide CO lp	
96a	− 6.89	0	1	amide CO lp	
95a	− 6.98	0	0	3-amide π	
94a	− 7.06	0	1	2-amide π	

Table S10. Frontier molecular orbitals for the nickel hook model *B* (Ni^{II}, *S* = 0).

**No.**	**E (eV)**	**Ni 3d (%)**	**S 3p (%)**	**assignment**	**Ni/S_2 overlap**
122a	− 0.48	0	0	1-His imidazole π^*	
121a	− 0.73	0	2	σ^*(N–H)	
120a	− 0.81	0	1	1-amido π^*	
119a	− 0.98	0	0	2-amide π^*	
118a	− 1.09	0	25	σ^*(S–H)	
117a	− 1.15	0	12	4-amide π^*	
116a	− 1.33	0	0	3-amide π^*	
115a	− 1.51	0	4	6-amide π^*	
114a	− 1.56	0	0	5-amide π^*	
113a	− 3.29	45	27	Ni $3d_{x2-y2}$ (LUMO)	σ^*
112a	− 4.44	79	9	Ni $3d_{z}^{2}$ (HOMO)	
111a	− 4.67	65	16	Ni $3d_{xz}$	π^* (S2)
110a	− 4.86	75	4	Ni $3d_{yz}$	π^* (S6)
109a	− 5.59	0	9	1-His imidazole π	
108a	− 5.76	56	26	Ni $3d_{xy}$	
107a	− 5.96	18	2	amide CO lp	
106a	− 6.03	9	3	amide CO lp	
105a	− 6.12	0	0	amide CO lp	
104a	− 6.19	0	0	amide CO lp	
103a	− 6.21	0	0	4-am π	
102a	− 6.33	34	19	S2 p_{π}	π (d_{xz})
101a	− 6.46	0	0	amide CO lp	
100a	− 6.67	3	6	1-His imidazole lp	

Table S10. continued.

No.	E (eV)	Ni 3d (%)	S 3p (%)	assignment	Ni/S_2 overlap
99a	− 6.75	10 {	19	S2 p_σ − amide CO lp	σ }
98a	− 6.76	9	19	S2 p_σ + amide CO lp	σ
97a	− 6.89	0	1	3-amide π	
96a	− 6.91	5	0	1-amido π	
95a	− 7.00	0	0	1-His imidazole π	
94a	− 7.08	1	4	2-amide π	
93a	− 7.23	0	4	6-amide π	
92a	− 7.34	2	42	S6 p_σ	σ
91a	− 7.55	0	11	5-amide π	

Table S11. Frontier molecular orbitals for the nickel hook model *C* (Ni^{II}, *S* = 0).

**No.**	**E (eV)**	**Ni 3d (%)**	**S 3p (%)**	**assignment**	**Ni/S_2 overlap**
121a	− 0.76	1	0	1-imido π*	
120a	− 0.90	0	0	2-amide π*	
119a	− 1.05	0	0	4-amide π*	
118a	− 1.08	0	0	6-amide π*	
117a	− 1.15	0	0	1-His imidazole π*	
116a	− 1.23	0	0	3-amide π*	
115a	− 1.37	0	0	5-amide π*	
114a	− 1.79	0	0	1-His imidazole π*	
113a	− 2.80	44	32	Ni $3d_{x2-y2}$ (LUMO)	σ*
112a	− 4.14	68	18	Ni $3d_{yz}$ (HOMO)	π* (S6)
111a	− 4.30	66	25	Ni $3d_{xz}$	π* (S2)
110a	− 4.44	87	0	Ni $3d_{z}^{2}$	
109a	− 5.27	61	20	Ni $3d_{xy}$	
108a	− 5.56	22	39	$2Sp_{\pi}$ – $6Sp_{\pi}$	δ
107a	− 5.77	9	43	$6Sp_{\pi}$ + $2Sp_{\pi}$	π
106a	− 5.95	18	17	amide CO lp	
105a	− 6.06	3	2	amide CO lp	
104a	− 6.08	12	10	amide CO lp	
103a	− 6.12	1	5	amide CO lp	
102a	− 6.15	2	3	amide CO lp	
101a	− 6.19	8	10	amide CO lp	
100a	− 6.29	12	31	6S p_{σ}	σ
99a	− 6.56	11	43	2S p_{σ}	σ

Table S11. continued.

No.	E (eV)	Ni 3d (%)	S 3p (%)	assignment	Ni/S_2 overlap
98a	− 6.59	0	1	4-amide π	
97a	− 6.73	1	2	1-His imidazole π	
96a	− 6.79	2	1	1-amido π	

Table S12. Frontier molecular orbitals for the nickel hook model *D* (Ni^{III}, *S* = ½).

No.	E (eV)	Ni 3d (%)	S 3p (%)	assignment	Ni/S$_2$ overlap
**α spin**					
121a	− 0.55	0	0	σ*(N–H)	
120a	− 0.90	0	0	1-amido π*	
119a	− 1.02	0	0	1-His imidazole π*	
118a	− 1.13	0	2	6-amide π*	
117a	− 1.14	0	0	4-amide π*	
116a	− 1.21	0	0	2-amide π*	
115a	− 1.34	0	0	3-amide π*	
114a	− 1.50	0	0	5-amide π*	
113a	− 3.98	31	44	Ni $3d_{x2-y2}$ (α-spin LUMO)	σ*
112a	− 5.30	13	71	S2 p_π − S6 p_π (α-spin HOMO)	δ*
111a	− 5.36	25	47	S6 p_π + S6 p_π	π*
110a	− 5.56	22	10	Ni $3d_{z2}$	
109a	− 6.02	1	6	1-His imidazole π	
108a	− 6.12	3	16	amide CO lp	
107a	− 6.17	0	0	amide CO lp	
106a	− 6.22	0	0	4-amide π	
105a	− 6.25	0	0	amide CO lp	
104a	− 6.26	16	8	amide CO lp	
103a	− 6.29	1	1	amide CO lp	
102a	− 6.64	19	12	Ni $3d_{yz}$	π (S6)
101a	− 6.66	13	12	amide CO lp	

Table S12. continued.

No.	E (eV)	Ni 3d (%)	S 3p (%)	assignment	Ni/S_2 overlap
100a	− 6.78	26	13	S6 p_σ	σ
99a	− 6.90	20	27	Ni $3d_{xz}$	π (S2)
98a	− 6.97	0	0	6-amide π	
97a	− 6.98	0	0	3-amide π	
96a	− 7.06	31	7	1-amido π	
95a	− 7.27	9	5	2-amide π	
94a	− 7.30	32	13	S2 p_σ	σ
93a	− 7.38	3	11	5-amide π	
92a	− 7.48	57	10	Ni $3d_{xy}$	
91a	− 7.93	20	1	1-His imidazole π	
**β spin**					
121a	− 0.53	0	3	σ*(N–H)	
120a	− 0.88	0	0	1-amido π*	
119a	− 1.01	0	0	1-His imidazole π*	
118a	− 1.13	0	2	6-amide π*	
117a	− 1.14	0	0	4-amide π*	
116a	− 1.20	0	0	2-amide π*	
115a	− 1.33	0	0	3-amide π*	
114a	− 1.50	0	0	5-amide π*	
113a	− 3.65	39	35	Ni $3d_{x2-y2}$	σ^*_{eq}
112a	− 4.33	57	15	Ni $3d_{z^2}$ (β-spin LUMO)	
111a	− 5.16	21	69	S2 p_π − S6 p_π (β-spin HOMO)	δ*

Table S12. continued.

No.	E (eV)	Ni 3d (%)	S 3p (%)	assignment	Ni/S_2 overlap
110a	− 5.40	30	27	S2 p_π + S6 p_π	π^*
109a	− 5.99	4	14	1-His imidazole π	
108a	− 6.06	7	10	amide CO lp	
107a	− 6.10	11	12	amide CO lp	
106a	− 6.17	0	0	amide CO lp	
105a	− 6.22	0	0	4-amide π	
104a	− 6.24	0	0	amide CO lp	
103a	− 6.27	2	0	amide CO lp	
102a	− 6.41	33	24	Ni $3d_{yz}$	π
101a	− 6.52	30	16	Ni $3d_{xy}$	
100a	− 6.69	41	16	Ni $3d_{xz}$	π
99a	− 6.71	11	10	amide CO lp	
98a	− 6.91	18	8	1-amido π	
97a	− 6.97	0	0	6-amide π	
96a	− 6.98	0	0	3-amide π	
95a	− 7.18	27	29	S6 p_σ	σ
94a	− 7.19	29	17	S2 p_σ	σ
93a	− 7.26	16	11	2-amide π	
92a	− 7.38	2	3	5-amide π	
91a	− 7.83	14	1	1-His imidazole π	

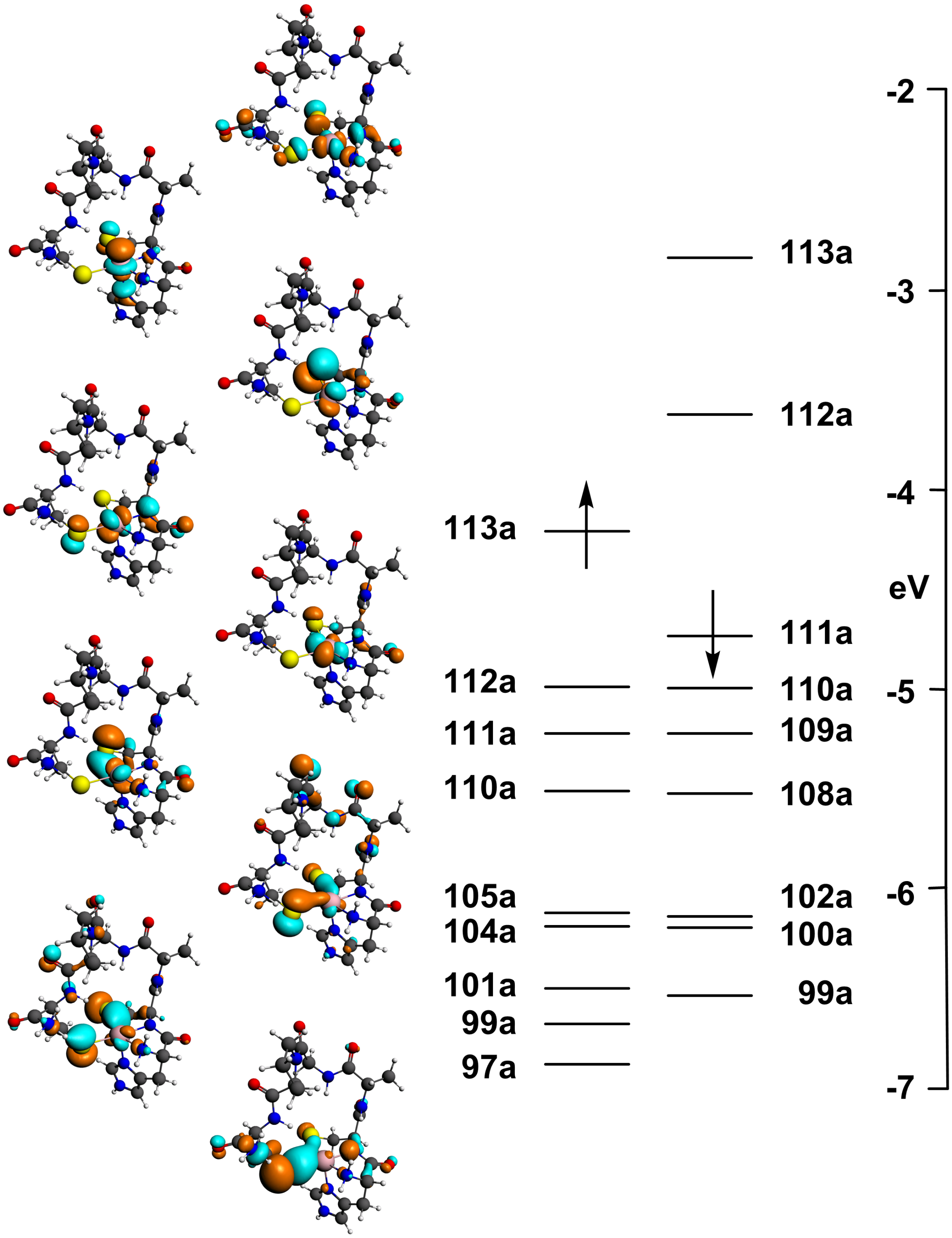

Figure S5. Relative energies of select Ni 3d- and S 3p-based frontier orbitals (from Table S9, right) for the nickel hook model *A* (Ni^{II}, *S* = 1), with isocontour plots of the β-spin orbitals (left).

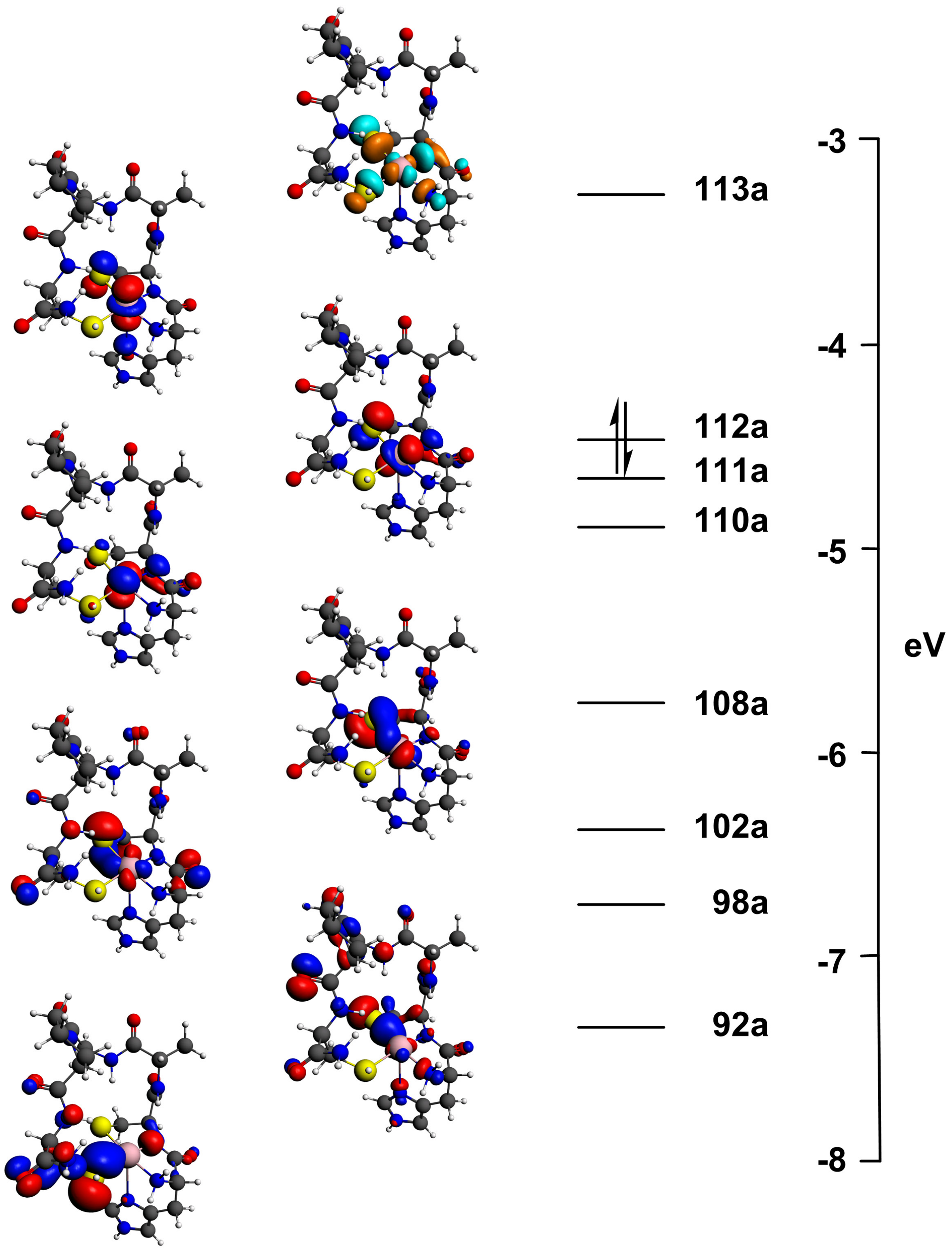

Figure S6. Relative energies of select Ni 3d- and S 3p-based frontier orbitals (from Table S10, right) for the nickel hook model *B* (Ni^{II}, S = 0), with isocontour plots of the β-spin orbitals (left).

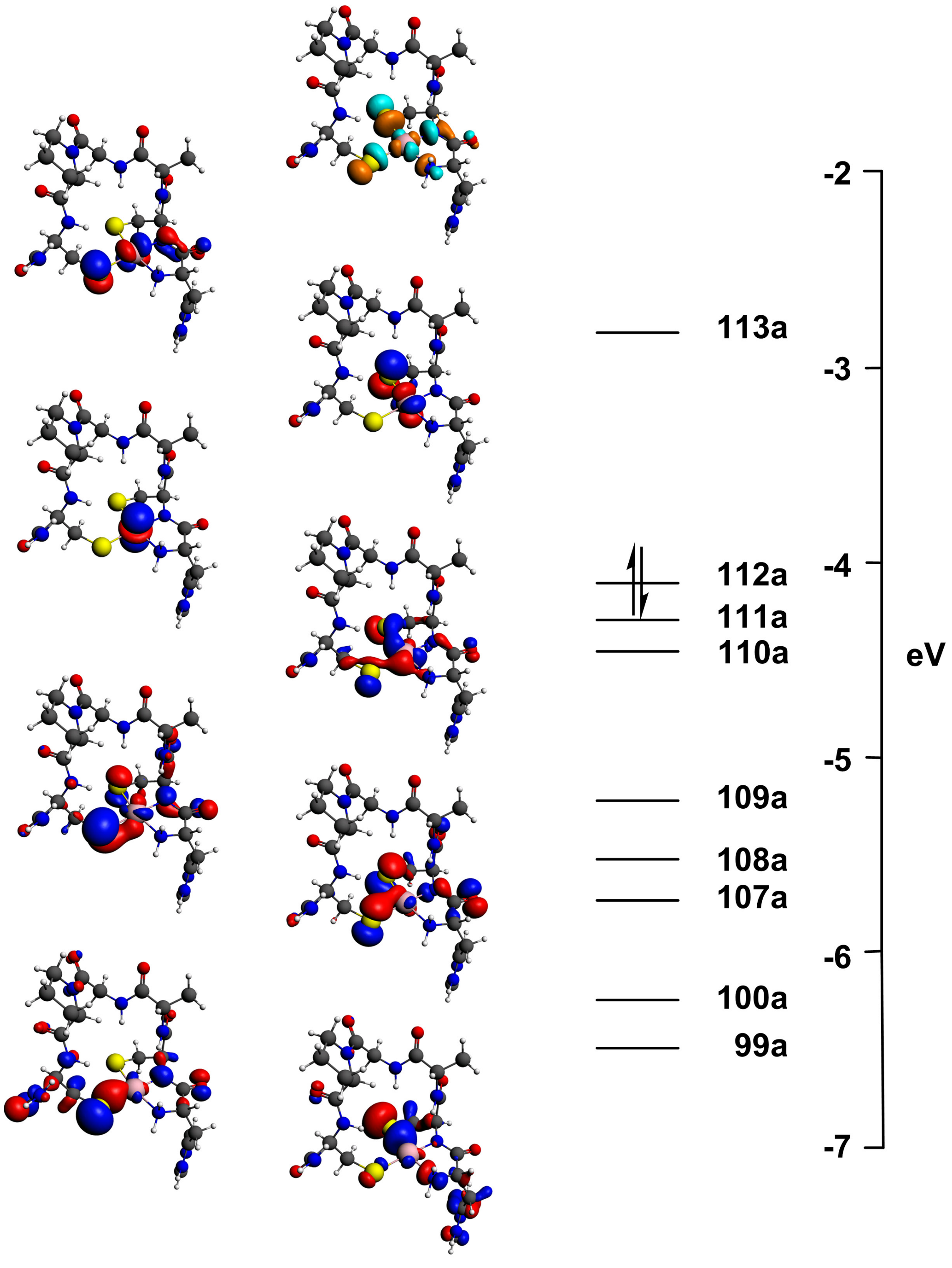

Figure S7. Relative energies of select Ni 3d- and S 3p-based frontier orbitals (from Table S11, right) for the nickel hook model *C* (Ni^{II}, *S* = 0), with isocontour plots of the β-spin orbitals (left).

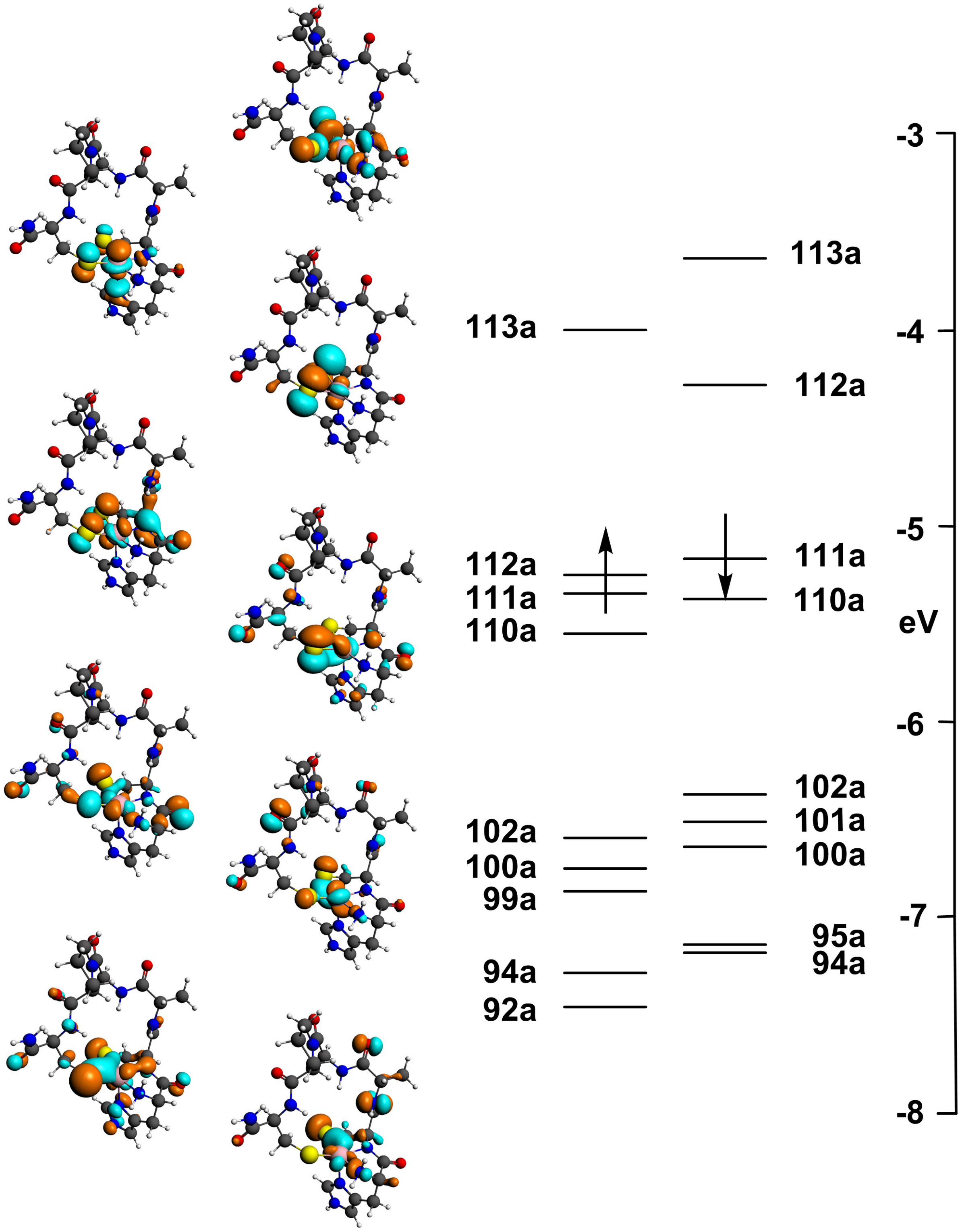

Figure S8. Relative energies of select Ni 3d- and S 3p-based frontier orbitals (from Table S12, right) for the nickel hook model *D* (Ni^{III}, S = ½), with isocontour plots of the β-spin orbitals (left).

Table S13. Calculated electronic excitations for the nickel hook model *A* (Ni^{II}, *S* = 1).

No.	E/eV	f	orbitals	fraction	assignment
1A	1.238	0.0020	111a → 112a β	0.972	LF
2A	1.426	0.0021	110a → 112a β	0.972	LF
3A	1.552	0.0016	113a → 114a α	0.998	MLCT
4A	1.930	0.0011	109a → 112a β	0.481	LF
			111a → 113a β	0.353	LF
			108a → 112a β	0.102	2S p_π → Ni d_{z2}
5A	1.971	0.0032	109a → 112a β	0.478	LF
			111a → 113a β	0.308	LF
			108a → 112a β	0.145	2S p_π → Ni d_{z2}
6A	2.026	0.0012	108a → 112a β	0.554	2 Sp_π → Ni d_{z2}
			111a → 113a β	0.302	LF
7A	2.122	0.0006	111a → 114a β	0.951	MLCT
8A	2.165	0.0064	107a → 112a β	0.755	1am π → Ni d_{z2}
			108a → 112a β	0.118	2S p_π → Ni d_{z2}
9A	2.257	0.0015	106a → 112a β	0.627	LMCT
			110a → 113a β	0.312	LF
10A	2.297	0.0003	110a → 113a β	0.491	LF
			106a → 112a β	0.238	LMCT
11A	2.316	0.0040	112a → 114a α	0.854	MLCT
12A	2.338	0.0014	105a → 112a β	0.845	LMCT
13A	2.383	0.0034	110a → 114a β	0.874	MLCT
14A	2.474	0.0021	109a → 113a β	0.939	LF
15A	2.521	0.0007	104a → 112a β	0.995	LMCT
16A	2.572	0.0001	111a → 114a α	0.999	MLCT
17A	2.618	0.0008	113a → 115a α	0.946	MLCT
18A	2.627	0.0004	103a → 112a β	0.812	LMCT
			102a → 112a β	0.154	6S p_π → Ni d_{z2}

Table S13. continued.

No.	E/eV	f	orbitals	fraction	assignment
19A	2.655	0.0011	102a → 112a β	0.781	6S p_π → Ni d_{z2}
			103a → 112a β	0.115	LMCT
20A	2.686	0.0001	101a → 112a β	0.945	LMCT
21A	2.704	0.0000	109a → 114a β	0.812	MLCT
			100a → 112a β	0.145	2S p_σ → Ni d_{z2}
22A	2.713	0.0010	100a → 112a β	0.683	2S p_σ → Ni d_{z2}
			109a → 114a β	0.176	MLCT
23A	2.824	0.0075	108a → 113a β	0.690	2S p_π → Ni d_σ*
			108a → 114a β	0.128	intraligand
24A	2.840	0.0012	113a → 116a α	0.995	MLCT
25A	2.879	0.0104	110a → 114a α	0.869	MLCT
26A	2.901	0.0039	108a → 114a β	0.762	intraligand
			107a → 113a β	0.107	1am π → Ni d_σ*
27A	2.920	0.0043	107a → 113a β	0.753	1am π → Ni d_σ*
28A	3.026	0.0022	107a → 114a β	0.482	intraligand
			106a → 113a β	0.248	LMCT
			99a → 112a β	0.181	6S p_σ → Ni d_{z2}
29A	3.039	0.0071	107a → 114a β	0.422	intraligand
			98a → 112a β	0.301	LMCT
			106a → 113a β	0.210	LMCT
30A	3.059	0.0169	99a → 112a β	0.479	6S p_σ → Ni d_{z2}
			98a → 112a β	0.363	LMCT
31A	3.076	0.0018	105a → 113a β	0.692	MLCT
			99a → 112a β	0.140	6S p_σ → Ni d_{z2}
32A	3.095	0.0028	106a → 113a β	0.229	MLCT
			109a → 114a α	0.215	intraligand
			105a → 113a β	0.215	LMCT
			98a → 112a β	0.182	LMCT
33A	3.119	0.0023	109a → 114a α	0.714	intraligand

Table S13. continued.

No.	E/eV	f	orbitals	fraction	assignment
34A	3.169	0.0014	106a → 114a β	0.578	intraligand
			111a → 115a β	0.348	MLCT
35A	3.171	0.0009	113a → 117a α	0.974	MLCT
36A	3.172	0.0016	111a → 115a β	0.629	MLCT
			106a → 114a β	0.285	intraligand
37A	3.212	0.0018	113a → 118a α	0.992	MLCT
38A	3.222	0.0001	108a → 114a α	0.991	intraligand
39A	3.258	0.0021	105a → 114a β	0.959	intraligand
40A	3.264	0.0003	97a → 112a β	0.986	LMCT
41A	3.265	0.0002	104a → 113a β	0.994	LMCT
42A	3.289	0.0004	107a → 114a α	0.989	intraligand
43A	3.324	0.0001	96a → 112a β	0.991	LMCT
44A	3.331	0.0002	113a → 119a α	0.989	MLCT
45A	3.374	0.0001	103a → 113a β	0.704	LMCT
			102a → 113a β	0.291	6S p_{π} → Ni d_{σ}*
46A	3.377	0.0006	112a → 115a α	0.801	MLCT
			111a → 116a β	0.183	MLCT
47A	3.387	0.0032	111a → 116a β	0.810	MLCT
			112a → 115a α	0.178	MLCT
48A	3.410	0.0000	95a → 112a β	0.586	LMCT
			106a → 114a α	0.387	intraligand
49A	3.411	0.0000	106a → 114a α	0.597	intraligand
			95a → 112a β	0.395	LMCT
50A	3.420	0.0013	110a → 115a β	0.942	MLCT
51A	3.431	0.0001	101a → 113a β	0.931	LMCT
52A	3.440	0.0000	104a → 114a β	0.997	intraligand

Table S13. continued.

No.	E/eV	f	orbitals	fraction	assignment
53A	3.445	0.0012	100a → 113a β	0.300	2S p_π → Ni d_σ*
			102a → 113a β	0.291	6S p_π → Ni d_σ*
			113a → 120a α	0.158	MLCT
			103a → 113a β	0.108	LMCT
54A	3.474	0.0034	113a → 120a α	0.768	MLCT
			105a → 114a α	0.104	intraligand
55A	3.488	0.0005	105a → 114a α	0.777	intraligand
56A	3.490	0.0013	94a → 112a β	0.837	LMCT
57A	3.511	0.0001	104a → 114a α	0.948	intraligand
58A	3.527	0.0000	103a → 114a α	0.923	intraligand
59A	3.540	0.0026	103a → 114a β	0.584	intraligand
60A	3.553	0.0015	102a → 114a β	0.666	intraligand
			103a → 114a β	0.244	intraligand
61A	3.584	0.0015	102a → 114a α	0.945	intraligand
62A	3.598	0.0073	112a → 116a α	0.897	MLCT
63A	3.610	0.0042	101a → 114a β	0.726	intraligand
			100a → 114a β	0.205	intraligand
64A	3.615	0.0138	100a → 114a β	0.231	intraligand
			101a → 114a β	0.163	intraligand
			102a → 114a β	0.108	intraligand
65A	3.635	0.0002	110a → 116a β	0.996	MLCT
66A	3.643	0.0010	111a → 115a α	0.993	MLCT
67A	3.661	0.0190	100a → 114a β	0.284	intraligand
			113a → 121a α	0.268	MLCT
			100a → 113a β	0.116	LMCT
68A	3.673	0.0013	113a → 121a α	0.701	MLCT
69A	3.716	0.0011	111a → 117a β	0.996	MLCT
70A	3.763	0.0005	111a → 118a β	0.968	MLCT

Table S14. Calculated electronic excitations for the nickel hook model *B* (Ni^{II}, S = 0).

No.	E/eV	f	orbitals	fraction	assignment
1A	1.690	0.0002	112a → 113a	0.520	LF
			111a → 113a	0.448	LF
2A	1.828	0.0002	111a → 113a	0.489	LF
			112a → 113a	0.401	LF
3A	2.016	0.0002	110a → 113a	0.882	LF
4A	2.306	0.0014	109a → 113a	1.000	LMCT
5A	2.738	0.0039	108a → 113a	0.920	LF
6A	2.793	0.0019	107a → 113a	0.600	LMCT
			106a → 113a	0.185	LMCT
			105a → 113a	0.161	LMCT
7A	2.844	0.0008	105a → 113a	0.833	LMCT
			107a → 113a	0.123	LMCT
8A	2.901	0.0017	104a → 113a	0.871	LMCT
9A	2.914	0.0073	103a → 113a	0.728	LMCT
			106a → 113a	0.171	LMCT
10A	2.938	0.0203	106a → 113a	0.541	LMCT
			103a → 113a	0.266	LMCT
			107a → 113a	0.115	LMCT
11A	3.017	0.0011	112a → 114a	0.988	MLCT
12A	3.070	0.0005	112a → 115a	0.991	MLCT
13A	3.118	0.0004	111a → 114a	0.990	MLCT
14A	3.149	0.0017	101a → 113a	0.576	LMCT
			102a → 113a	0.356	$2Sp_{\pi} \rightarrow Nid_{\sigma}*$
15A	3.178	0.0009	111a → 115a	0.951	MLCT
16A	3.247	0.0001	112a → 116a	0.890	MLCT
17A	3.260	0.0031	102a → 113a	0.508	$2Sp_{\pi} \rightarrow Nid_{\sigma}*$
			101a → 113a	0.331	LMCT
18A	3.300	0.0019	110a → 114a	0.985	MLCT

Table S14. continued.

No.	E/eV	f	orbitals	fraction	assignment
19A	3.355	0.0033	111a → 116a	0.884	MLCT
			110a → 115a	0.108	MLCT
20A	3.358	0.0007	110a → 115a	0.883	MLCT
			111a → 116a	0.105	MLCT
21A	3.423	0.0020	100a → 113a	0.495	LMCT
			112a → 117a	0.419	MLCT
22A	3.442	0.0011	112a → 117a	0.514	MLCT
			100a → 113a	0.353	LMCT
23A	3.469	0.0009	98a → 113a	0.467	$2Sp_{\sigma} \rightarrow Nid_{\sigma^*}$
			99a → 113a	0.438	$2Sp_{\sigma} \rightarrow Nid_{\sigma^*}$
24A	3.506	0.0004	112a → 118a	0.947	MLCT
25A	3.530	0.0004	110a → 116a	0.965	MLCT
26A	3.544	0.0017	111a → 117a	0.921	MLCT
27A	3.592	0.0002	112a → 119a	0.519	MLCT
			97a → 113a	0.441	LMCT
28A	3.596	0.0031	97a → 113a	0.483	LMCT
			112a → 119a	0.470	MLCT
29A	3.614	0.0010	111a → 118a	0.893	MLCT
30A	3.650	0.0004	96a → 113a	0.707	$1am\ \pi \rightarrow Nid_{\sigma^*}$
			95a → 113a	0.124	LMCT
31A	3.707	0.0009	111a → 119a	0.971	MLCT
32A	3.719	0.0007	110a → 117a	0.885	MLCT
33A	3.743	0.0001	95a → 113a	0.784	LMCT
34A	3.798	0.0008	112a → 120a	0.726	MLCT
			112a → 121a	0.118	MLCT
35A	3.803	0.0007	110a → 118a	0.823	MLCT
36A	3.813	0.0012	94a → 113a	0.893	LMCT

Table S14. continued.

No.	E/eV	f	orbitals	fraction	assignment
37A	3.875	0.0016	110a → 119a	0.392	MLCT
			112a → 121a	0.390	MLCT
			111a → 120a	0.135	MLCT
38A	3.881	0.0017	110a → 119a	0.583	MLCT
			112a → 121a	0.317	MLCT
39A	3.939	0.0031	111a → 120a	0.473	MLCT
			93a → 113a	0.334	LMCT
40A	3.943	0.0002	93a → 113a	0.581	LMCT
			111a → 120a	0.153	MLCT
			111a → 121a	0.116	MLCT
41A	3.994	0.0062	111a → 121a	0.749	MLCT
42A	4.029	0.0000	109a → 114a	1.000	intraligand
43A	4.089	0.0028	109a → 115a	0.943	intraligand
44A	4.090	0.0366	112a → 122a	0.258	MLCT
			98a → 113a	0.135	2Sp_{σ} → Nid_{σ}*
			99a → 113a	0.122	2Sp_{σ} → Nid_{σ}*
45A	4.113	0.0123	110a → 120a	0.403	MLCT
			112a → 122a	0.396	MLCT
46A	4.116	0.0112	112a → 122a	0.315	MLCT
			110a → 120a	0.306	MLCT

Table S15. Calculated electronic excitations for the nickel hook model *C* (Ni^{II}, $S = 0$).

No.	E/eV	f	orbitals	fraction	assignment
1A	1.730	0.0005	112a → 113a	0.982	LF
2A	1.867	0.0002	111a → 113a	0.962	LF
3A	2.213	0.0004	110a → 113a	0.988	LF
4A	2.347	0.0000	112a → 114a	0.999	MLCT
5A	2.503	0.0004	111a → 114a	1.000	MLCT
6A	2.645	0.0007	110a → 114a	0.999	MLCT
7A	2.736	0.0088	109a → 113a	0.907	LF
8A	2.785	0.0040	112a → 115a	0.948	MLCT
9A	2.894	0.0008	108a → 113a	0.889	$Sp_{\delta} \rightarrow Nid_{\sigma^*}$
10A	2.919	0.0013	112a → 116a	0.998	MLCT
11A	2.938	0.0001	111a → 115a	0.996	MLCT
12A	3.004	0.0032	112a → 117a	0.977	MLCT
13A	3.067	0.0012	112a → 118a	0.967	MLCT
14A	3.077	0.0009	111a → 116a	0.978	MLCT
15A	3.088	0.0026	110a → 115a	0.962	MLCT
16A	3.101	0.0022	112a → 119a	0.974	MLCT
17A	3.151	0.0022	111a → 117a	0.987	MLCT
18A	3.169	0.0174	107a → 113a	0.763	$Sp_{\pi} \rightarrow Nid_{\sigma^*}$
19A	3.219	0.0004	111a → 118a	0.588	MLCT
			110a → 116a	0.408	MLCT
20A	3.223	0.0029	110a → 116a	0.567	MLCT
			111a → 118a	0.389	MLCT
21A	3.244	0.0015	112a → 120a	0.394	MLCT
			106a → 113a	0.287	LMCT
			105a → 113a	0.194	LMCT

Table S15. continued.

No.	E/eV	f	orbitals	fraction	assignment
22A	3.252	0.0022	112a → 120a	0.546	MLCT
			105a → 113a	0.242	LMCT
			106a → 113a	0.115	LMCT
23A	3.261	0.0040	111a → 119a	0.951	MLCT
24A	3.288	0.0018	105a → 113a	0.398	LMCT
			106a → 113a	0.265	LMCT
			103a → 113a	0.122	LMCT
25A	3.294	0.0008	110a → 117a	0.940	MLCT
26A	3.333	0.0001	103a → 113a	0.720	LMCT
			102a → 113a	0.146	LMCT
27A	3.353	0.0000	102a → 113a	0.461	LMCT
			104a → 113a	0.337	LMCT
28A	3.360	0.0002	110a → 118a	0.988	MLCT
29A	3.398	0.0003	110a → 119a	0.951	MLCT
30A	3.409	0.0020	111a → 120a	0.847	MLCT
31A	3.420	0.0019	104a → 113a	0.361	LMCT
			102a → 113a	0.202	LMCT
			112a → 121a	0.161	MLCT
32A	3.435	0.0001	101a → 113a	0.707	LMCT
33A	3.471	0.0001	109a → 114a	0.995	MLCT
34A	3.534	0.0229	112a → 121a	0.450	Nid → 1-im π^*
			110a → 120a	0.307	MLCT
35A	3.553	0.0109	110a → 120a	0.681	MLCT
			112a → 121a	0.178	Nid → 1am π^*
36A	3.576	0.0019	111a → 121a	0.943	Nid → 1am π^*
37A	3.747	0.0031	110a → 121a	0.721	Nid → 1am π^*
			112a → 122a	0.226	MLCT
38A	3.768	0.0003	108a → 114a	0.983	intraligand
39A	3.779	0.0093	98a → 113a	0.836	LMCT

Table S15. continued.

No.	E/eV	f	orbitals	fraction	assignment
40A	3.798	0.0535	112a → 122a	0.616	MLCT
			110a → 121a	0.156	Nid → 1am π^*
41A	3.898	0.0101	97a → 113a	0.524	LMCT
			99a → 113a	0.252	$2Sp_\sigma$ → Ni d_σ^*
			109a → 115a	0.137	MLCT
42A	3.903	0.0025	109a → 115a	0.583	MLCT
			111a → 122a	0.344	MLCT
43A	3.908	0.0005	111a → 122a	0.620	MLCT
			109a → 115a	0.245	MLCT
			97a → 113a	0.101	LMCT
44A	3.971	0.0440	100a → 113a	0.378	$6Sp_\sigma$ → Ni d_σ^*
			110a → 122a	0.138	MLCT

Table S16. Calculated electronic excitations for the nickel hook model *D* (Ni^{III}, *S* = ½).

No.	E/eV	f	orbitals	fraction	assignment
1A	0.911	0.0027	111a → 112a β	0.978	$Sp_{\delta}*$ → Ni d_{z2}
2A	1.097	0.0032	110a → 112a β	0.980	$Sp_{\pi}*$ → Ni d_{z2}
3A	1.240	0.0003	112a → 113a α	0.698	$Sp_{\delta}*$ → Ni $d_{\sigma}*$
			111a → 113a β	0.267	$Sp_{\delta}*$ → Ni $d_{\sigma}*$
4A	1.418	0.0002	111a → 113a α	0.778	$Sp_{\pi}*$ → Ni $d_{\sigma}*$
			110a → 113a α	0.107	LF
5A	1.504	0.0003	110a → 113a α	0.553	LF
			110a → 113a β	0.161	$Sp_{\pi}*$ → Ni $d_{\sigma}*$
			111a → 113a α	0.133	$Sp_{\pi}*$ → Ni $d_{\sigma}*$
6A	1.641	0.0011	111a → 113a β	0.638	$Sp_{\delta}*$ → Ni $d_{\sigma}*$
			112a → 113a α	0.192	$Sp_{\delta}*$ → Ni $d_{\sigma}*$
7A	1.684	0.0037	109a → 112a β	0.937	LMCT
8A	1.770	0.0026	108a → 112a β	0.650	LMCT
			107a → 112a β	0.247	LMCT
9A	1.833	0.0011	107a → 112a β	0.577	LMCT
			106a → 112a β	0.186	LMCT
			108a → 112a β	0.137	LMCT
10A	1.850	0.0003	106a → 112a β	0.588	LMCT
			110a → 113a β	0.219	$Sp_{\pi}*$ → Ni $d_{\sigma}*$
11A	1.872	0.0010	110a → 113a β	0.301	$Sp_{\pi}*$ → Ni $d_{\sigma}*$
			106a → 112a β	0.212	LMCT
			110a → 113a α	0.168	LF
			108a → 112a β	0.131	LMCT
12A	1.898	0.0001	105a → 112a β	0.989	LMCT
13A	1.919	0.0000	104a → 112a β	0.996	LMCT
14A	1.951	0.0007	103a → 112a β	0.956	LMCT
15A	2.043	0.0019	109a → 113a α	0.866	LMCT
16A	2.111	0.0010	108a → 113a α	0.656	LMCT
			102a → 112a β	0.123	LF

Table S16. continued.

No.	E/eV	f	orbitals	fraction	assignment
17A	2.180	0.0071	102a → 112a β	0.686	LF
18A	2.190	0.0004	107a → 113a α	0.936	LMCT
19A	2.239	0.0001	106a → 113a α	0.845	LMCT
20A	2.256	0.0003	101a → 112a β	0.493	LF
			105a → 113a α	0.285	LMCT
			106a → 113a α	0.117	LMCT
21A	2.267	0.0003	105a → 113a α	0.671	LMCT
			101a → 112a β	0.193	LF
22A	2.277	0.0004	104a → 113a α	0.535	LMCT
			108a → 113a β	0.147	LMCT
			101a → 112a β	0.122	LF
23A	2.305	0.0008	103a → 113a α	0.877	LMCT
24A	2.374	0.0024	100a → 112a β	0.860	LF
25A	2.383	0.0024	109a → 113a β	0.496	LMCT
			108a → 113a β	0.253	LMCT
			107a → 113a β	0.124	LMCT
26A	2.410	0.0003	99a → 112a β	0.732	LMCT
			108a → 113a β	0.144	LMCT
27A	2.465	0.0051	107a → 113a β	0.493	LMCT
			101a → 113a α	0.104	LMCT
28A	2.509	0.0058	106a → 113a β	0.372	LMCT
			108a → 113a β	0.160	LMCT
			101a → 113a α	0.114	LMCT
29A	2.527	0.0034	106a → 113a β	0.580	LMCT
			101a → 113a α	0.120	LMCT
30A	2.571	0.0001	105a → 113a β	0.906	LMCT
31A	2.587	0.0003	104a → 113a β	0.324	LMCT
			102a → 113a α	0.231	LF
			98a → 112a β	0.140	1am π → Nid_{z2}
			102a → 113a β	0.123	LF

Table S16. continued.

No.	E/eV	f	orbitals	fraction	assignment
32A	2.594	0.0001	104a → 113a β	0.627	LMCT
			98a → 112a β	0.173	1am π → Nid$_{z2}$
33A	2.628	0.0051	98a → 112a β	0.339	1am π → Nid$_{z2}$
			102a → 113a α	0.131	LF
34A	2.637	0.0002	103a → 113a β	0.530	LMCT
			97a → 112a β	0.277	LMCT
35A	2.647	0.0003	97a → 112a β	0.581	LMCT
			96a → 112a β	0.120	LMCT
			103a → 113a β	0.108	LMCT
36A	2.661	0.0014	96a → 112a β	0.832	LMCT
37A	2.717	0.0011	101a → 113a α	0.290	LMCT
			100a → 113a α	0.281	2Sp$_{\sigma}$ → Ni d$_{\sigma}$*
			102a → 113a α	0.184	LF
			102a→ 113a β	0.106	LF
38A	2.723	0.0404	101a → 113a β	0.214	LF
			98a → 112a β	0.167	1am π → Nid$_{z2}$
			107a → 113a β	0.117	LMCT
39A	2.794	0.0030	99a → 113a α	0.360	LF
			100a → 113a β	0.294	LF
40A	2.831	0.0007	95a → 112a β	0.659	6Sp$_{\sigma}$ → Ni d$_{z2}$
			94a → 112a β	0.112	2Sp$_{\sigma}$ → Ni d$_{z2}$
41A	2.896	0.0001	94a → 112a β	0.591	Sσ → Ni d$_{z2}$
			95a → 112a β	0.169	S σ → Ni d$_{z2}$
			93a → 112a β	0.112	LMCT
42A	2.940	0.0002	93a → 112a β	0.400	LMCT
			102a → 113a β	0.144	LF
			100a → 113a α	0.138	6Sp$_{\sigma}$ → Ni d$_{\sigma}$*
43A	2.980	0.0053	98a → 113a α	0.474	LMCT
			93a → 112a β	0.172	LMCT
44A	2.994	0.0013	98a → 113a α	0.425	LMCT
			97a → 113a β	0.418	LMCT

Table S16. continued.

No.	E/eV	f	orbitals	fraction	assignment
45A	3.010	0.0009	97a → 113a α	0.440	LMCT
			93a → 112a β	0.103	LMCT
46A	3.035	0.0075	92a → 112a β	0.288	LMCT
			96a → 113a α	0.221	LMCT
			98a → 113a β	0.111	LMCT
47A	3.048	0.0001	99a → 113a β	0.432	LMCT
			92a → 112a β	0.343	LMCT
			100a → 113a β	0.118	LF
48A	3.088	0.0035	92a → 112a β	0.304	LMCT
			99a → 113a β	0.137	LMCT
49A	3.157	0.0183	101a → 113a β	0.281	LF
			94a → 113a β	0.126	2Sp$_\sigma$ → Ni d$_\sigma$*
			96a → 113a α	0.110	1amπ → Ni d$_\sigma$*
50A	3.197	0.0026	92a → 113a α	0.200	LF
			94a → 113a α	0.180	2Sp$_\sigma$ → Ni d$_\sigma$*
			95a → 113a α	0.148	LMCT
			98a → 113a β	0.145	1amπ → Ni d$_\sigma$*
			96a → 113a α	0.127	1amπ → Ni d$_\sigma$*
51A	3.239	0.0041	94a → 113a α	0.295	2Sp$_\sigma$ → Ni d$_\sigma$*
			95a → 113a β	0.177	6Sp$_\sigma$ → Ni d$_\sigma$*
			92a → 113a α	0.129	LF
			93a → 113a α	0.100	LMCT
52A	3.268	0.0057	95a → 113a α	0.518	LMCT
			92a → 113a α	0.106	LF
53A	3.319	0.0000	97a → 113a β	0.938	LMCT
54A	3.330	0.0003	96a → 113a β	0.851	LMCT
55A	3.357	0.0065	98a → 113a β	0.367	1amπ → Ni d$_\sigma$*
			93a → 113a α	0.199	LMCT
			96a → 113a β	0.112	LMCT
			94a → 113a α	0.109	2Sp$_\sigma$ → Ni d$_\sigma$*
56A	3.394	0.0002	93a → 113a α	0.523	LMCT
			95a → 113a β	0.109	6Sp$_\sigma$ → Ni d$_\sigma$*

Table S16. continued.

**No.**	**E/eV**	**f**	**orbitals**	**fraction**	**assignment**
57A	3.461	0.0222	100a → 113a β	0.229	LF
			99a → 113a α	0.134	LF
			95a → 113a α	0.129	LMCT
58A	3.508	0.0006	91a → 112a β	0.963	LMCT
59A	3.607	0.0033	93a → 113a β	0.634	LMCT
			94a → 113a β	0.252	2Sp_σ → Ni d_σ*
60A	3.666	0.0002	111a → 114a β	0.991	intraligand
61A	3.700	0.0064	92a → 113a β	0.795	LMCT
			95a → 113a β	0.124	6Sp_σ → Ni d_σ*
62A	3.796	0.0005	112a → 114a α	0.984	intraligand
63A	3.829	0.0006	111a → 115a β	0.992	intraligand
64A	3.834	0.0085	90a → 112a β	0.894	Nσ → Ni d_{z2}
65A	3.861	0.0015	111a → 114a α	0.980	intraligand
66A	3.900	0.0016	110a → 114a β	0.991	intraligand
67A	3.927	0.0475	91a → 113a α	0.538	LMCT
68A	3.961	0.0022	111a → 116a β	0.679	intraligand
			112a → 115a α	0.285	intraligand
69A	3.962	0.0008	112a → 115a α	0.671	intraligand
			111a → 116a β	0.298	intraligand

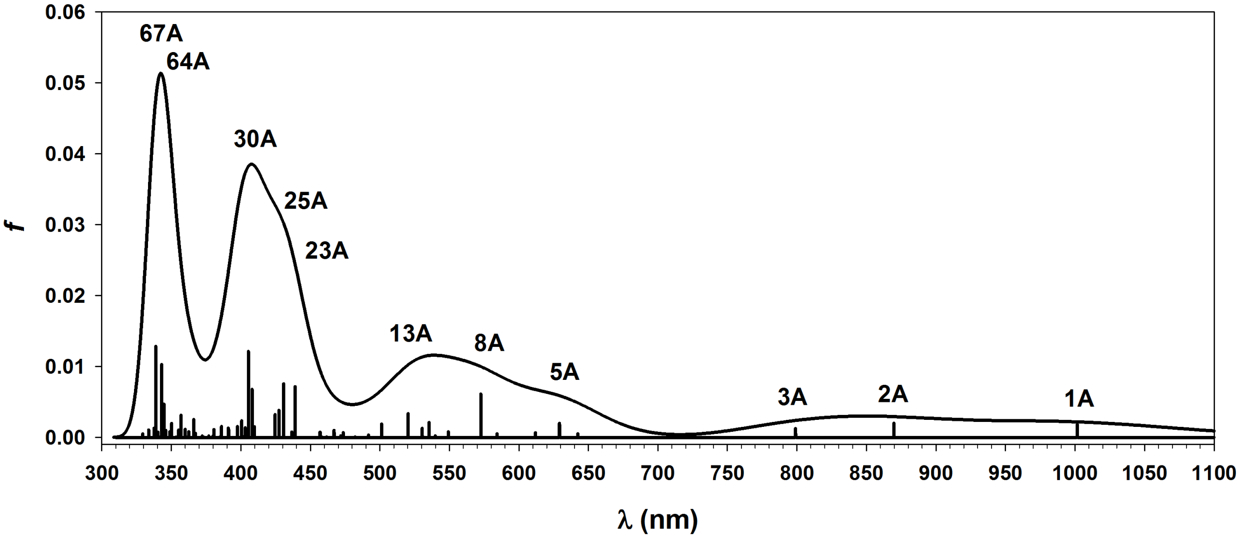

Figure S9. Calculated electronic spectrum of the nickel hook model *A* (Ni^{II}, *S* = 1), rendered with arbitrary peak widths of 1700 cm^{-1}. All calculated transitions are listed in Table S13.

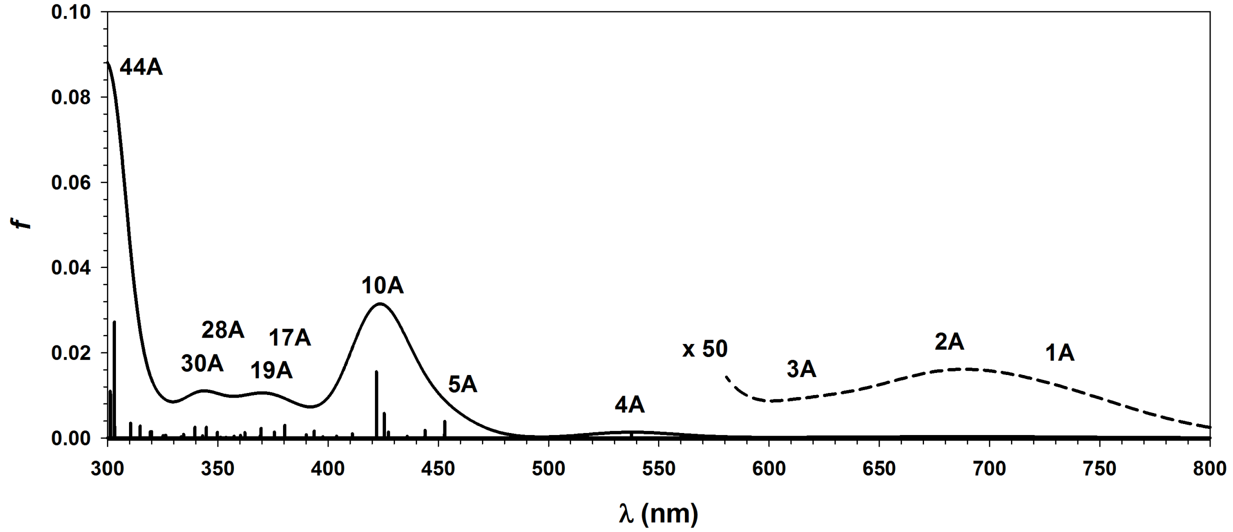

Figure S10. Calculated electronic spectrum of the nickel hook model *B* (Ni^{II}, *S* = 0), rendered with arbitrary peak widths of 1700 cm^{-1}. All calculated transitions are listed in Table S14.

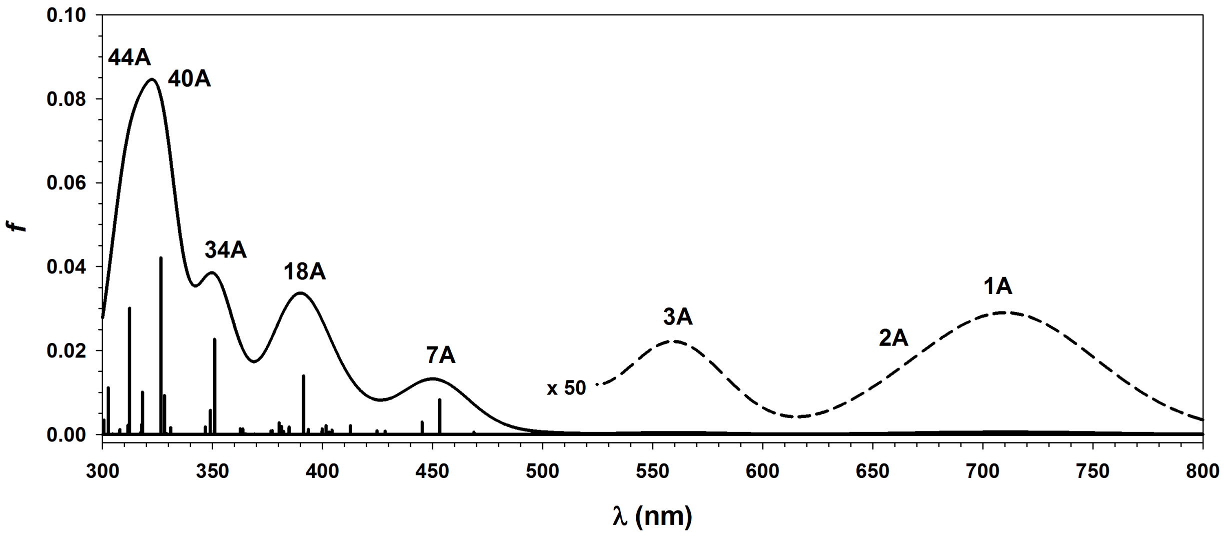

Figure S11. Calculated electronic spectrum of the nickel hook model *C* (Ni^{II}, *S* = 0), rendered with arbitrary peak widths of 1700 cm^{-1}. All calculated transitions are listed in Table S15.

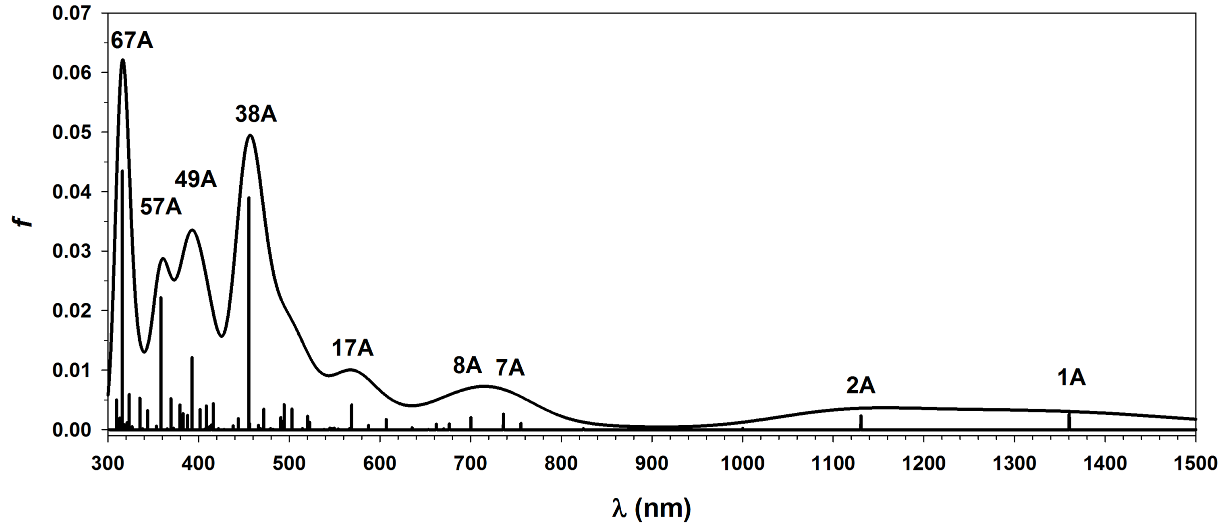

Figure S12. Calculated electronic spectrum of the nickel hook model *D* (Ni^{III}, *S* = ½), rendered with arbitrary peak widths of 1700 cm^{-1}. All calculated transitions are listed in Table S16.

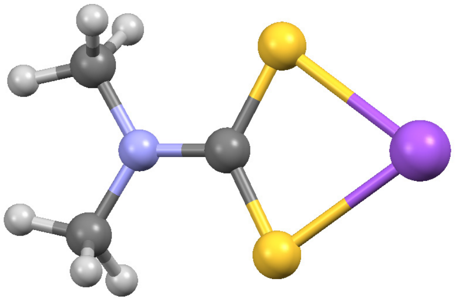

Figure S13. Optimized structure of C_{2v}-NaS_2CNMe_2 complex, model *0*.

Table S17. Optimized atomic coordinates for C_{2v}-NaS_2CNMe_2, model *0*.

	X	Y	Z
Na	0.00000000	0.00000000	2.98454607
S	0.00000000	-1.54822063	0.80606510
S	0.00000000	1.54822063	0.80606510
C	0.00000000	0.00000000	0.00000000
N	0.00000000	0.00000000	-1.36153398
C	0.00000000	1.25940910	-2.11297230
C	0.00000000	-1.25940910	-2.11297230
H	-0.88605744	-1.85993252	-1.86373775
H	0.88605744	-1.85993252	-1.86373775
H	0.00000000	-1.02999325	-3.18346915
H	0.88605744	1.85993252	-1.86373775
H	-0.88605744	1.85993252	-1.86373775
H	0.00000000	1.02999325	-3.18346915

Table S18. Frontier molecular orbitals for the C_{2v}-$[S_2CNMe_2]^-$ free ligand anion, which was derived by deletion of Na^+ from model *0*.

No.	E (eV)	S 3p (%)	C 2p (%)	N 2p (%)	symmetry	
$4b_1$	− 1.32	52	33	18	π^*	(LUMO)
$6b_2$	− 4.43	99	0	0	$\sigma_{+/-}$	(HOMO)
$2a_2$	− 5.06	99	0	0	δ	
$3b_1$	− 5.38	45	0	39	π	
$7a_1$	− 5.39	91	2	2	$\sigma_{+/+}$	

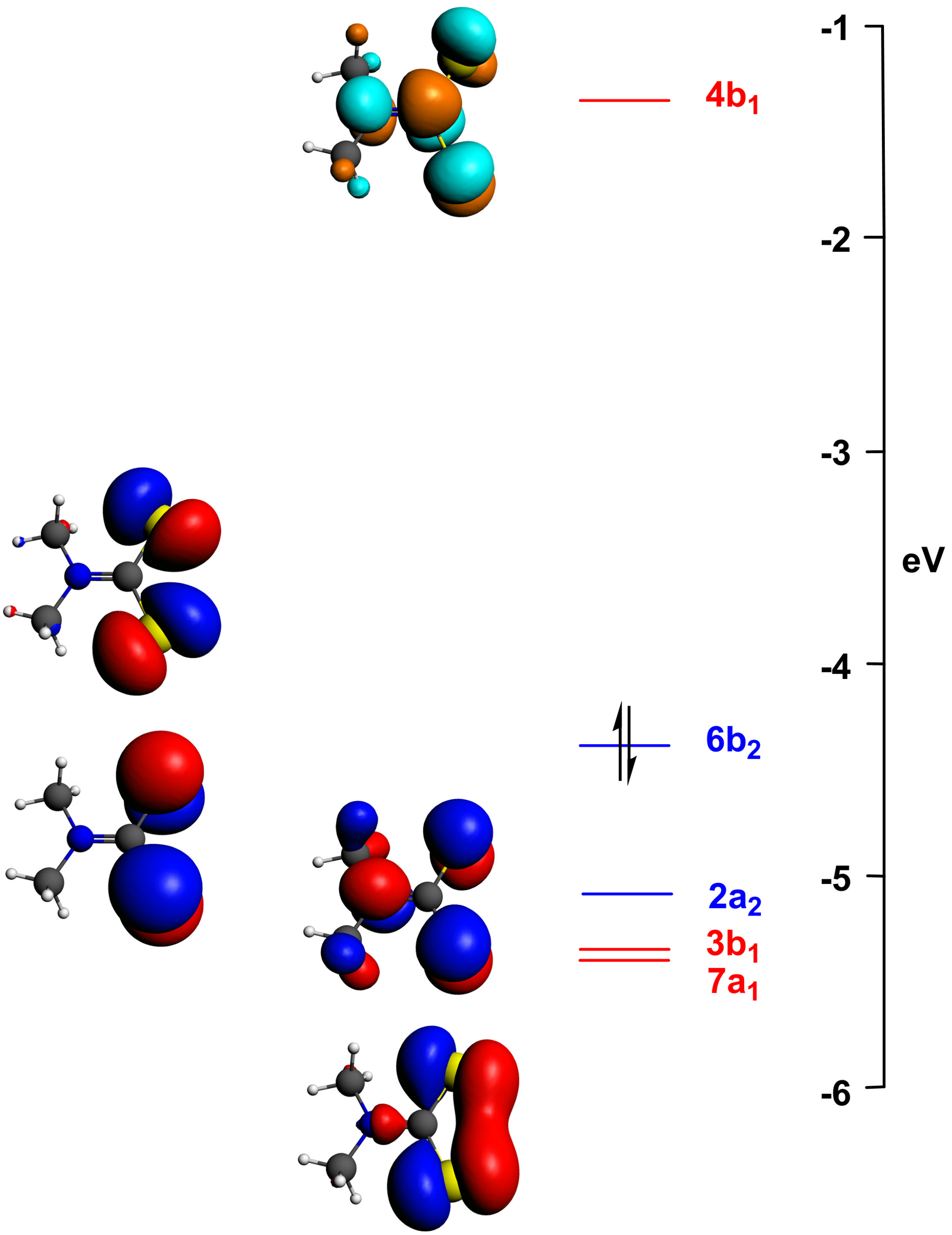

Figure S14. Relative energies of frontier orbitals (from Table S18, right) for the C_{2v}-$[S_2CNMe_2]^-$ free ligand anion, which was obtained by deletion of Na^+ from model *0*, with isocontour plots of the orbitals (left).

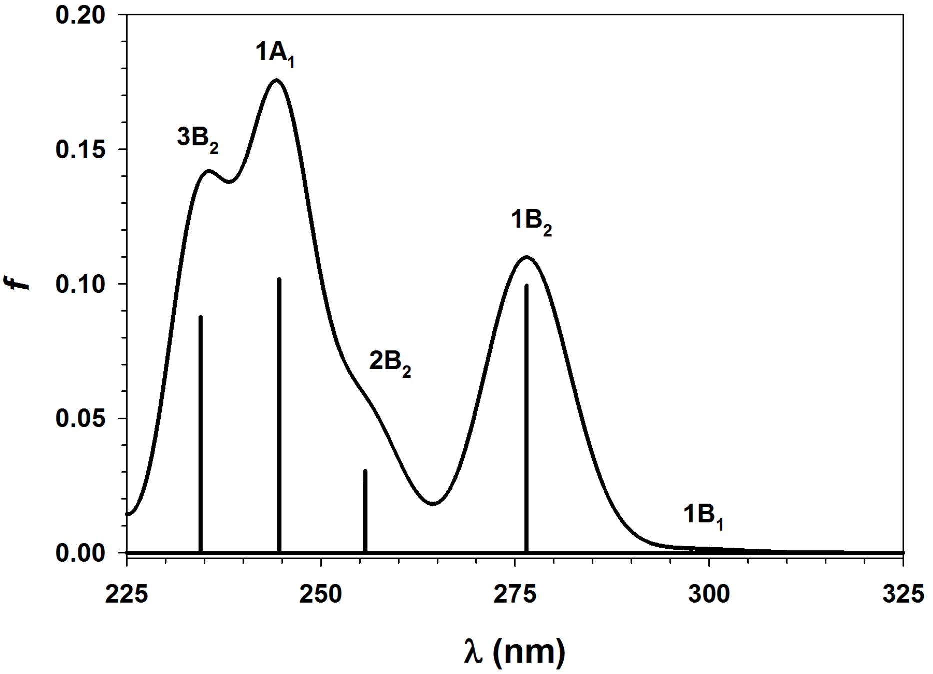

Figure S15. Calculated electronic spectrum of the C_{2v}-$[S_2CNMe_2]^-$ free ligand anion, with peak widths arbitrarily set to 1700 cm^{-1}. Transitions are given in Table S19.

Table S19. Calculated electronic excitations for the C_{2v}-$[S_2CNMe_2]^-$ free ligand anion.

No.	E/eV	*f*	orbitals	fraction
$1B_1$	4.167	0.001	$7a_1 \rightarrow 4b_1$	0.999
$1B_2$	4.484	0.110	$2a_2 \rightarrow 4b_1$	0.873
$2B_2$ [(a)]	4.849	0.050	$6b_2 \rightarrow 8a_1$	0.921
$1A_1$	5.068	0.166	$3b_1 \rightarrow 4b_1$	0.929
$3B_2$ [(b)]	5.287	0.131	$6b_2 \rightarrow 9a_1$	0.856

(a) The $8a_1$ acceptor orbital (not shown in Figure S14) is primarily C–H σ^* in character.
(b) The $9a_1$ acceptor orbital (not shown in Figure S14) is primarily C–S σ^* in character.

Table S20. Calculated normal modes of vibration for *C_{2v}-exo*-NaS_2CNMe_2, model *0*.

mode	cm^{-1}	Intensity	Assignment
1 A_1	3065	33	ν(C–H)
2	2944	30	ν(C–H)
**3**	**1473**	**110**	**δ(Me)**
4	1437	0	δ(Me)
**5**	**1317**	**179**	**ν(C=N)**
**6**	**1112**	**28**	**ρ(Me)**
7	858	0	ν_s(N–Me_2)/ν_s(C–S_2)
8	573	6	ν_s(C–S)/δ(N–Me_2)
9	428	8	δ(N–Me_2)/ν_s(C–S_2)
10	349	15	δ(CS_2)/ν_s(Na–S)
11	216	37	ν_s(Na–S_2C)
1 A_2	3009	0	ν(C–H)
2	1413	0	δ(Me)
3	1079	0	ρ(Me)
4	125	0	ρ(Me_2N–CS_2)
5	53	0	τ_{as}(N–Me_2)
1 B_1	3010	30	ν(C–H)
2	1427	24	δ(Me)
3	1099	0	ρ(Me)
4	556	3	π(S_2*C*N)
5	221	1	π(C*N*Me_2)
6	51	33	π(Na)
7	−12	−0.3	τ_s(N–Me_2)
1 B_2	3052	2	ν(C–H)
2	2937	17	ν(C–H)
3	1447	1	δ(Me)
4	1385	0	δ(Me)
**5**	**1245**	**69**	**ν_{as}(N–Me_2)**
6	1029	15	ρ(Me)
**7**	**949**	**184**	**ν_{as}(C–S_2)**
8	434	17	ρ(Me_2N–CS_2)
9	286	0	ν_{as}(Na–S_2C)
10	185	17	ρ(NaS_2CNMe_2)

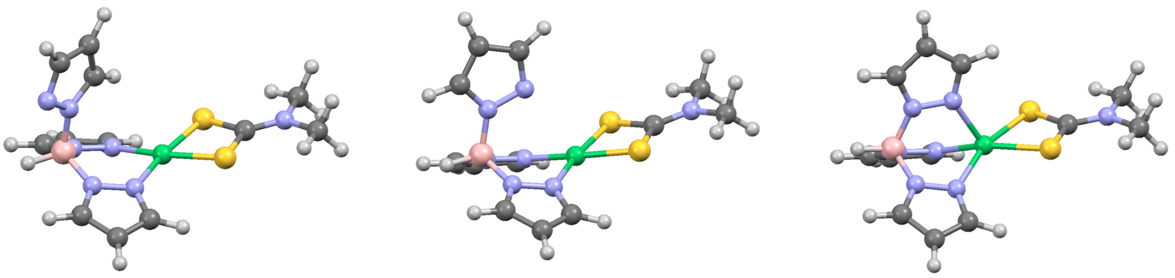

Figure S16. Optimized DFT models *1-3* of square-planar/pyramidal [(Tp)NiS_2CNMe_2]: C_1 (S = 0), left; C_s (S = 0), middle; C_s (S = 1), right. Atomic coordinates are given in Tables S30–S32, respectively.

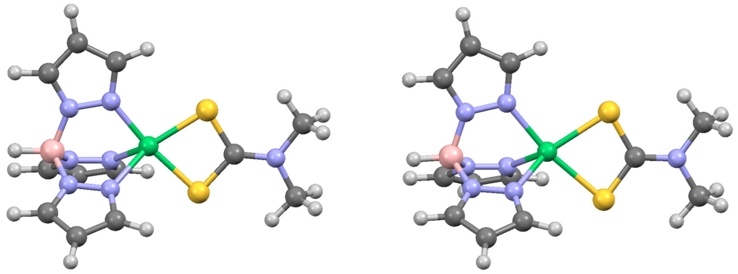

Figure S17. DFT models *4* and *5* of trigonal-bipyramidal [(Tp)NiS_2CNMe_2]: C_s (S = 0), left; C_s (S = 1), right. Atomic coordinates are given in Tables S33 and S34, respectively.

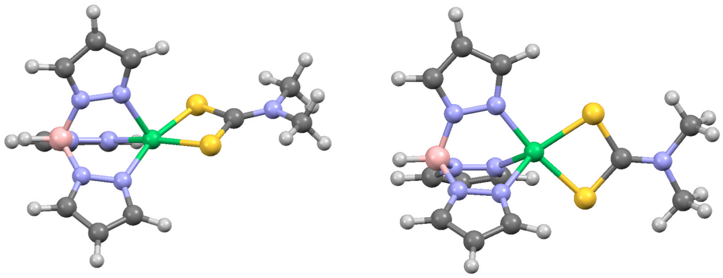

Figure S18. DFT models of square-pyramidal *6* (left) and trigonal-bipyramidal *7* (right) of C_s-[(Tp)NiS_2CNMe_2]$^+$ (S = ½). Atomic coordinates are given in Tables S35 and S36, respectively.

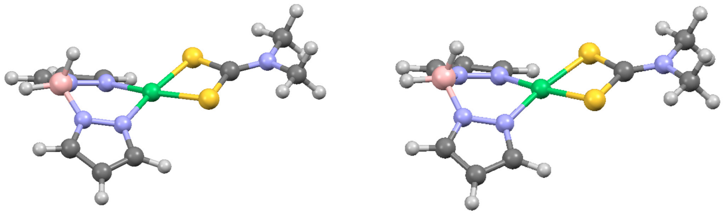

Figure S19. Optimized DFT models *8* and *9* of C_s-[(Bp)NiS_2CNMe_2]$^{n+}$: n = 0 (S = 0), left; n = 1 (S = ½), right. Atomic coordinates are given in Tables S37 and S38, respectively.

Table S21. Relative energies (eV) calculated for optimized [(Tp)NiS_2CNMe_2] models.

Model	*S*	symmetry	geometry	BP86	OPBE	B3LYP*
1	0	C_1	square-planar	0.00	0.02	0.02
2	0	C_s	square-pyramidal	0.00	0.00	0.00
3	1	C_s	square-pyramidal	0.16	0.13	−0.20
4	0	C_s	trigonal-bipyramidal	0.25	0.27	0.34
5	1	C_s	trigonal-bipyramidal	0.20	0.20	−0.15

Table S22. Relative energies (eV) calculated for optimized [(Tp)NiS_2CNMe_2]$^+$ models.

Model	*S*	symmetry	geometry	BP86
6	½	C_s	square-pyramidal	0.00
7	½	C_s	trigonal-bipyramidal	0.09
	$^3/_2$	C_s	square-pyramidal	0.93 [(a)]
	$^3/_2$	C_s	trigonal-bipyramidal	1.11 [(a)]

(a) converged to non-aufbau electronic configuration

Table S23. Relative energies (eV) calculated for optimized [(Bp)NiS_2CNMe_2] models.

Model	*S*	symmetry	geometry	BP86
8	0	C_s	square-planar	0.00
	1	C_s	distorted tetrahedral	0.70

Table S24. Relative energies (eV) calculated for optimized [(Bp)NiS_2CNMe_2]$^+$ models.

Model	*S*	symmetry	geometry	BP86
9	½	C_s	square-planar	0.00
	½	C_s	distorted tetrahedral	0.56
	$^3/_2$	C_s	distorted tetrahedral	0.84 [(a)]

(a) converged to non-aufbau electronic configuration

Table S25. Bond lengths (Å) calculated for optimized Ni(II) synthetic models.

Model	*S*	sym.	geom.	Ni–N_{ax}	Ni–N_{eq}	Ni–S_{ax}	Ni–S_{eq}	S_{ax}–C	S_{eq}–C	C=NMe_2	Ni•••N_2S_2	Ni•••N_2S
8	0	C_s	sq. pl.		1.941		2.240		1.739	1.325	0.019	
1	0	C_1	sq. pl.		1.939<br>1.942		2.241<br>2.240		1.738<br>1.738	1.325	0.023	
2	0	C_s	sq. pyr.	3.055	1.941		2.242		1.737	1.328	0.047	
3	1	C_s	sq. pyr.	2.033	2.092		2.416		1.734	1.352	0.300	
4	0	C_s	trig. bipyr.	1.943	2.149	2.246	2.243	1.726	1.720	1.349		0.087
5	1	C_s	trig. bipyr.	2.112	2.059	2.459	2.376	1.725	1.744	1.351		0.068

Table S26. Bond lengths (Å) calculated for optimized oxidized model cations.

Model	*S*	sym.	geom.	Ni–N_{ax}	Ni–N_{eq}	Ni–S_{ax}	Ni–S_{eq}	S_{ax}–C	S_{eq}–C	C=NMe_2	Ni•••N_2S_2	Ni•••N_2S
9	½	C_s	sq. pl.		1.945		2.203		1.743	1.311	0.035	
6	½	C_s	sq. pyr.	2.050	1.987		2.261		1.738	1.318	0.233	
7	½	C_s	trig. bipyr.	1.978	2.031	2.259	2.252	1.731	1.745	1.316		0.088

Table S27. Bond angles (°) calculated for optimized Ni(II) synthetic models.

Model	*S*	sym.	geom.	N_{ax}–Ni–N_{eq}	N_{eq}–Ni–N_{eq}	N_{eq}–Ni–S_{eq} *trans*	N_{eq}–Ni–S_{eq} *cis*	N_{eq}–Ni–S_{eq}	N_{eq}–Ni–S_{ax}	N_{ax}–Ni–S_{ax}	N_{ax}–Ni–S_{eq}	S–Ni–S	S–C–S
8	0	C_s	sq. pl.		93.89	171.92	94.11					77.86	108.06
1	0	C_1	sq. pl.		93.67	171.83<br>171.92	94.39<br>94.21					77.71	107.95
2	0	C_s	sq. pyr.	78.97	93.00	171.94	94.58				105.20	77.73	108.14
3	1	C_s	sq. pyr.	89.37	88.49	163.06	96.37				106.85	74.61	115.23
4	0	C_s	trig. bipyr.	89.76	86.69			136.39	95.63	172.57	95.72	76.85	108.09
5	1	C_s	trig. bipyr.	88.98	88.03			135.82	98.01	170.24	95.70	74.53	115.19

Table S28. Bond angles (°) calculated for optimized oxidized model cations.

Model	*S*	sym.	geom.	N_{ax}–Ni–N_{eq}	N_{eq}–Ni–N_{eq}	N_{eq}–Ni–S_{eq} *trans*	N_{eq}–Ni–S_{eq} *cis*	N_{eq}–Ni–S_{eq}	N_{eq}–Ni–S_{ax}	N_{ax}–Ni–S_{ax}	N_{ax}–Ni–S_{eq}	S–Ni–S	S–C–S
9	½	C_s	sq. pl.		94.42	171.70	93.61					78.29	105.86
6	½	C_s	sq. pyr.	90.05	90.94	165.44	94.69				103.34	76.85	107.91
7	½	C_s	trig. bipyr.	90.80	88.05			135.69	95.31	171.48	94.57	76.91	107.59

Table S29. Calculated Mulliken charges and spin densities.

**Model**	**Na/Ni**		**S**		**C**		**N**	
	Z	***S***	***Z***	***S***	***Z***	***S***	***Z***	***S***
0	0.74	0.00	−0.50	0.00	0.03	0.00	−0.13	0.00
1	0.05	0.00	−0.03<br>−0.03	0.00	0.07	0.00	−0.12	0.00
2	0.09	0.00	−0.05	0.00	0.06	0.00	−0.12	0.00
3	0.28	1.41	−0.20	0.16	0.04	−0.02	−0.13	0.02
4 ax<br>eq	0.02	0.00	−0.02<br>−0.04	0.00	0.05	0.00	−0.14	0.00
5 ax<br>eq	0.25	1.39	−0.19<br>−0.19	0.18<br>0.15	0.04	−0.02	−0.13	0.02
6	0.11	0.76	0.11	−0.03	0.10	0.01	−0.12	0.03
7 ax<br>eq	0.08	0.74	0.12<br>0.13	−0.06<br>0.03	0.10	0.01	−0.11	0.05
8	0.05	0.00	−0.04	0.00	0.07	0.00	−0.12	0.00
9	0.16	0.48	0.21	0.19	0.11	−0.02	−0.10	−0.01

Table S30. Optimized atomic coordinates for model *1.*

	X	Y	Z
Ni	0.00000000	0.00000000	0.00000000
B	-3.29905824	0.32071381	-0.04049677
H	-4.36884021	0.86596515	-0.06925600
N	-2.50263420	0.78592010	-1.28864909
N	-1.15360178	0.60725160	-1.43481673
N	-3.51627945	-1.21519152	-0.03854264
N	-4.56908276	-1.73986582	-0.74544396
N	-2.54443100	0.77123065	1.22895196
N	-1.19010736	0.63845082	1.39486792
C	-3.00903411	1.32882583	-2.42430605
C	-1.97082109	1.51461271	-3.33255261
H	-2.03190030	1.93223217	-4.33165466
C	-0.82683606	1.05079001	-2.66734622
H	-4.07019979	1.53833634	-2.50483709
H	0.20450260	1.02704028	-3.00342943
C	2.77953630	-2.21006480	0.53369523
C	-3.36127091	-3.42824830	0.19860130
H	-3.03317838	-4.41941191	0.49772706
C	-4.46963901	-3.07409509	-0.60038229
H	-1.90304893	-1.98823191	1.13523957
H	-5.20598462	-3.72389132	-1.06744629
C	-3.08466989	1.30190414	2.35581042
C	-2.06671579	1.52299693	3.27762247
H	-2.15561913	1.94438513	4.27300210
C	-0.89847198	1.09438335	2.63106322
H	-4.15247314	1.48236560	2.41773952
H	0.12772027	1.10608486	2.98291716
S	1.56903929	-0.81533739	-1.37475569
S	1.55958238	-0.72876075	1.43493764
C	2.50229635	-1.17795271	0.04569576
N	3.71735658	-1.70643376	0.06746162
C	4.43099323	-2.06912459	-1.16042884
H	5.37927195	-1.51783827	-1.20363806
H	3.82299575	-1.82041189	-2.03477604
H	4.63868983	-3.14724028	-1.15343502
C	4.42738239	-1.97128142	1.32223917
H	4.62246194	-3.04818684	1.40958674
H	3.82370617	-1.63884626	2.17143884
H	5.38223361	-1.42969216	1.31660149

Table S31. Optimized atomic coordinates for model *2.*

	X	Y	Z
Ni	0.00000000	0.00000000	0.00000000
B	-3.24692351	0.24506894	0.00000000
H	-4.36305486	0.69017852	0.00000000
N	-2.50567785	0.77024018	-1.25571215
N	-1.14948148	0.68105800	-1.40785025
N	-3.33953555	-1.29512195	0.00000000
N	-2.22619850	-2.09215776	0.00000000
N	-2.50567785	0.77024018	1.25571215
N	-1.14948148	0.68105800	1.40785025
C	-3.04992164	1.31234229	-2.37537005
C	-2.02733557	1.58559363	-3.27929105
H	-2.11722692	2.02661267	-4.26623824
C	-0.85384512	1.17451645	-2.62781002
H	-4.12177035	1.46361428	-2.44679530
H	0.17634835	1.21949467	-2.96540042
C	-4.47719400	-2.04885510	0.00000000
C	-4.10382868	-3.38797576	0.00000000
H	-4.75737667	-4.25546219	0.00000000
C	-2.69022841	-3.35397623	0.00000000
H	-5.45782293	-1.58290479	0.00000000
H	-1.99120952	-4.18740273	0.00000000
C	-3.04992164	1.31234229	2.37537005
C	-2.02733557	1.58559363	3.27929105
H	-2.11722692	2.02661267	4.26623824
C	-0.85384512	1.17451645	2.62781002
H	-4.12177035	1.46361428	2.44679530
H	0.17634835	1.21949467	2.96540042
S	1.55372288	-0.79522463	-1.40661406
S	1.55372288	-0.79522463	1.40661406
C	2.46347339	-1.25493295	0.00000000
N	3.64514115	-1.86073097	0.00000000
C	4.32878879	-2.22993773	-1.24242288
H	5.33872489	-1.80035449	-1.23851743
H	3.77277499	-1.84693170	-2.10293027
H	4.40201863	-3.32384042	-1.31048334
C	4.32878879	-2.22993773	1.24242288
H	4.40201863	-3.32384042	1.31048334
H	3.77277499	-1.84693170	2.10293027
H	5.33872489	-1.80035449	1.23851743

Table S32. Optimized atomic coordinates for model *3.*

	X	Y	Z
Ni	0.00000000	0.00000000	0.00000000
B	1.88032976	2.46153953	0.00000000
H	2.62892897	3.40160888	0.00000000
N	0.97768694	2.49310670	1.25788455
N	0.06890937	1.49703989	1.45968153
N	2.68465087	1.13563780	0.00000000
N	2.03248738	-0.06233354	0.00000000
N	0.97768694	2.49310670	-1.25788455
N	0.06890937	1.49703989	-1.45968153
C	0.92507644	3.37873088	2.28452527
C	-0.04609015	2.94829246	3.18354927
H	-0.34298084	3.42411468	4.11208634
C	-0.55195148	1.76302822	2.61984108
H	1.57900025	4.24433190	2.30151972
H	-1.32391281	1.09306630	2.98486587
C	4.02471654	0.92107674	0.00000000
C	4.24961909	-0.45205157	0.00000000
H	5.20583802	-0.96413934	0.00000000
C	2.96531390	-1.02549868	0.00000000
H	4.71618261	1.75698743	0.00000000
H	2.66469081	-2.06801415	0.00000000
C	0.92507644	3.37873088	-2.28452527
C	-0.04609015	2.94829246	-3.18354927
H	-0.34298084	3.42411468	-4.11208634
C	-0.55195148	1.76302822	-2.61984108
H	1.57900025	4.24433190	-2.30151972
H	-1.32391281	1.09306630	-2.98486587
S	-0.75494871	-1.76740192	1.46425035
S	-0.75494871	-1.76740192	-1.46425035
C	-1.17909347	-2.59360984	0.00000000
N	-1.79511612	-3.79731637	0.00000000
C	-2.15952138	-4.49271768	1.23163690
H	-3.24876986	-4.64910787	1.26445193
H	-1.85048773	-3.89375593	2.09368185
H	-1.66150200	-5.47402313	1.26822757
C	-2.15952138	-4.49271768	-1.23163690
H	-1.66150200	-5.47402313	-1.26822757
H	-1.85048773	-3.89375593	-2.09368185
H	-3.24876986	-4.64910787	-1.26445193

Table S33. Optimized atomic coordinates for model *4.*

	X	Y	Z
Ni	0.00000000	0.00000000	0.00000000
B	1.22455252	2.85366627	0.00000000
H	1.61843353	3.98988383	0.00000000
N	1.71663107	2.11186260	1.26211827
N	1.32856859	0.82264661	1.47479284
N	1.71663107	2.11186260	-1.26211827
N	1.32856859	0.82264661	-1.47479284
N	-0.32895610	2.83740124	0.00000000
N	-1.01321870	1.65756734	0.00000000
C	2.53420858	2.52540078	2.26526725
C	2.68955658	1.47145439	3.15940348
H	3.27600038	1.46175311	4.07222349
C	1.91399473	0.42888858	2.61613632
H	2.94367874	3.53032659	2.26763597
H	1.74514545	-0.57612745	2.98942371
C	2.53420858	2.52540078	-2.26526725
C	2.68955658	1.47145439	-3.15940348
H	3.27600038	1.46175311	-4.07222349
C	1.91399473	0.42888858	-2.61613632
H	2.94367874	3.53032659	-2.26763597
H	1.74514545	-0.57612745	-2.98942371
C	-1.20932536	3.86879179	0.00000000
C	-2.50014330	3.34904468	0.00000000
H	-3.43489927	3.89913551	0.00000000
C	-2.32423573	1.95608362	0.00000000
H	-0.85221740	4.89291809	0.00000000
H	-3.06614284	1.16511824	0.00000000
S	0.91370174	-2.05126745	0.00000000
S	-1.78767316	-1.35483318	0.00000000
C	-0.69294899	-2.68179805	0.00000000
N	-1.03043105	-3.98818180	0.00000000
C	-0.02111986	-5.03908463	0.00000000
H	-0.13009921	-5.67066962	0.89529241
H	0.97728303	-4.58823957	0.00000000
H	-0.13009921	-5.67066962	-0.89529241
C	-2.42254232	-4.41810866	0.00000000
H	-2.63341489	-5.02256303	-0.89560445
H	-3.07506510	-3.53791116	0.00000000
H	-2.63341489	-5.02256303	0.89560445

Table S34. Optimized atomic coordinates for model *5.*

	X	Y	Z
Ni	0.00000000	0.00000000	0.00000000
B	1.05816884	2.92346038	0.00000000
H	1.48479004	4.04686419	0.00000000
N	1.55041658	2.16086966	1.25553639
N	1.21060514	0.85214948	1.43034853
N	1.55041658	2.16086966	-1.25553639
N	1.21060514	0.85214948	-1.43034853
N	-0.48877799	2.93594275	0.00000000
N	-1.17271426	1.75653530	0.00000000
C	2.33164180	2.57080720	2.28639954
C	2.50611178	1.49919396	3.15689886
H	3.07497938	1.48470588	4.08028623
C	1.78513374	0.44271482	2.57157620
H	2.70429774	3.58894921	2.32458921
H	1.65267785	-0.58171001	2.90414853
C	2.33164180	2.57080720	-2.28639954
C	2.50611178	1.49919396	-3.15689886
H	3.07497938	1.48470588	-4.08028623
C	1.78513374	0.44271482	-2.57157620
H	2.70429774	3.58894921	-2.32458921
H	1.65267785	-0.58171001	-2.90414853
C	-1.36097855	3.97538406	0.00000000
C	-2.65369850	3.46039910	0.00000000
H	-3.58554971	4.01581261	0.00000000
C	-2.47883329	2.06477368	0.00000000
H	-1.00118796	4.99895687	0.00000000
H	-3.22241933	1.27432805	0.00000000
S	0.99871884	-2.24689334	0.00000000
S	-1.83515180	-1.50913908	0.00000000
C	-0.64166096	-2.78023495	0.00000000
N	-0.99073847	-4.08535767	0.00000000
C	0.00264976	-5.15680888	0.00000000
H	-0.12640682	-5.78528994	0.89467957
H	1.00670421	-4.72142844	0.00000000
H	-0.12640682	-5.78528994	-0.89467957
C	-2.38181631	-4.53289930	0.00000000
H	-2.57689299	-5.14401402	-0.89457316
H	-3.04483846	-3.66277508	0.00000000
H	-2.57689299	-5.14401402	0.89457316

Table S35. Optimized atomic coordinates for model *6.*

	X	Y	Z
Ni	0.00000000	0.00000000	0.00000000
B	1.95820217	2.35222356	0.00000000
H	2.70756695	3.28285813	0.00000000
N	1.04164270	2.37359535	1.25015248
N	0.11150854	1.38863643	1.41616385
N	2.73294845	1.01098241	0.00000000
N	2.04310023	-0.16654089	0.00000000
N	1.04164270	2.37359535	-1.25015248
N	0.11150854	1.38863643	-1.41616385
C	0.96987514	3.23391228	2.29496298
C	-0.02865913	2.79333066	3.16202069
H	-0.34492519	3.25263117	4.09224028
C	-0.53952281	1.62786159	2.57043956
H	1.63027664	4.09292710	2.34690603
H	-1.33087455	0.96599141	2.90525640
C	4.06426565	0.74907927	0.00000000
C	4.23967271	-0.63301534	0.00000000
H	5.17661555	-1.17966173	0.00000000
C	2.93967069	-1.16765610	0.00000000
H	4.78484792	1.56044627	0.00000000
H	2.61326576	-2.20219968	0.00000000
C	0.96987514	3.23391228	-2.29496298
C	-0.02865913	2.79333066	-3.16202069
H	-0.34492519	3.25263117	-4.09224028
C	-0.53952281	1.62786159	-2.57043956
H	1.63027664	4.09292710	-2.34690603
H	-1.33087455	0.96599141	-2.90525640
S	-0.65743332	-1.64500379	1.40528397
S	-0.65743332	-1.64500379	-1.40528397
C	-1.08892755	-2.57211030	0.00000000
N	-1.64301710	-3.76808009	0.00000000
C	-1.98782217	-4.46411409	1.24455377
H	-3.07455548	-4.61144132	1.28289020
H	-1.66739991	-3.87380109	2.10844020
H	-1.48517232	-5.43903780	1.25859837
C	-1.98782217	-4.46411409	-1.24455377
H	-1.48517232	-5.43903780	-1.25859837
H	-1.66739991	-3.87380109	-2.10844020
H	-3.07455548	-4.61144132	-1.28289020

Table S36. Optimized atomic coordinates for model *7.*

	X	Y	Z
Ni	0.00000000	0.00000000	0.00000000
B	1.17611985	2.83945017	0.00000000
H	1.60594993	3.95445388	0.00000000
N	1.64408268	2.05652782	1.25073217
N	1.24639904	0.76162491	1.41172515
N	1.64408268	2.05652782	-1.25073217
N	1.24639904	0.76162491	-1.41172515
N	-0.37523392	2.84671129	0.00000000
N	-1.06389206	1.66720405	0.00000000
C	2.42888479	2.41805674	2.29596368
C	2.54654775	1.32692473	3.15444932
H	3.10172876	1.27652932	4.08506067
C	1.79001327	0.30583667	2.55469789
H	2.84217407	3.41937139	2.35497331
H	1.61135703	-0.71254734	2.88312456
C	2.42888479	2.41805674	-2.29596368
C	2.54654775	1.32692473	-3.15444932
H	3.10172876	1.27652932	-4.08506067
C	1.79001327	0.30583667	-2.55469789
H	2.84217407	3.41937139	-2.35497331
H	1.61135703	-0.71254734	-2.88312456
C	-1.25677775	3.87557969	0.00000000
C	-2.54913574	3.35226514	0.00000000
H	-3.48443093	3.90135231	0.00000000
C	-2.37850929	1.96099697	0.00000000
H	-0.90440924	4.90151288	0.00000000
H	-3.12486681	1.17417481	0.00000000
S	0.91984793	-2.06329699	0.00000000
S	-1.79546965	-1.35862038	0.00000000
C	-0.68720659	-2.70697983	0.00000000
N	-1.02288301	-3.97930302	0.00000000
C	-0.00783650	-5.03864129	0.00000000
H	-0.13846427	-5.65786471	0.89620654
H	0.99488038	-4.59931529	0.00000000
H	-0.13846427	-5.65786471	-0.89620654
C	-2.42122984	-4.42181879	0.00000000
H	-2.60206865	-5.02752646	-0.89641904
H	-3.08910708	-3.55521028	0.00000000
H	-2.60206865	-5.02752646	0.89641904

Table S37. Optimized atomic coordinates for model *8.*

	X	Y	Z
Ni	0.00000000	0.00000000	0.00000000
B	2.16906736	2.39109103	0.00000000
H	2.73294154	3.45916034	0.00000000
N	1.25774033	2.30371818	1.25616717
N	0.37879859	1.26981490	1.41823647
H	2.94346573	1.44863496	0.00000000
N	1.25774033	2.30371818	-1.25616717
N	0.37879859	1.26981490	-1.41823647
C	1.23486489	3.08664511	2.36066124
C	0.31736016	2.55635328	3.26821987
H	0.05649395	2.94195927	4.24796944
C	-0.19817700	1.41956385	2.63134596
H	1.87572345	3.95950687	2.42643151
H	-0.95188499	0.71708984	2.97156833
C	1.23486489	3.08664511	-2.36066124
C	0.31736016	2.55635328	-3.26821987
H	0.05649395	2.94195927	-4.24796944
C	-0.19817700	1.41956385	-2.63134596
H	1.87572345	3.95950687	-2.42643151
H	-0.95188499	0.71708984	-2.97156833
S	-0.53902205	-1.65660428	1.40727903
S	-0.53902205	-1.65660428	-1.40727903
C	-0.89114710	-2.61527008	0.00000000
N	-1.34350697	-3.86107479	0.00000000
C	-1.61477267	-4.59108078	1.24157079
H	-2.67663859	-4.86859102	1.27398269
H	-1.37444899	-3.96416382	2.10475343
H	-1.00184875	-5.50143945	1.26726894
C	-1.61477267	-4.59108078	-1.24157079
H	-1.00184875	-5.50143945	-1.26726894
H	-1.37444899	-3.96416382	-2.10475343
H	-2.67663859	-4.86859102	-1.27398269

Table S38. Optimized atomic coordinates for model *9.*

	X	Y	Z
Ni	0.00000000	0.00000000	0.00000000
B	2.12222066	2.41080830	0.00000000
H	2.69054907	3.47270586	0.00000000
N	1.20326256	2.32739433	1.25324517
N	0.34513424	1.27527374	1.42719633
H	2.87630645	1.45724963	0.00000000
N	1.20326256	2.32739433	-1.25324517
N	0.34513424	1.27527374	-1.42719633
C	1.15652220	3.11299605	2.34674759
C	0.23945604	2.57124298	3.25848417
H	-0.03472506	2.96384562	4.23134128
C	-0.24817811	1.42244707	2.63636069
H	1.78119009	3.99735777	2.41296284
H	-0.99178250	0.71095040	2.97851844
C	1.15652220	3.11299605	-2.34674759
C	0.23945604	2.57124298	-3.25848417
H	-0.03472506	2.96384562	-4.23134128
C	-0.24817811	1.42244707	-2.63636069
H	1.78119009	3.99735777	-2.41296284
H	-0.99178250	0.71095040	-2.97851844
S	-0.52370520	-1.62632738	1.39070195
S	-0.52370520	-1.62632738	-1.39070195
C	-0.92702813	-2.59644141	0.00000000
N	-1.41689023	-3.81282760	0.00000000
C	-1.71295215	-4.52952390	1.24674340
H	-2.78609085	-4.75446959	1.28040065
H	-1.43784116	-3.91506378	2.10837670
H	-1.14106860	-5.46481699	1.26139155
C	-1.71295215	-4.52952390	-1.24674340
H	-1.14106860	-5.46481699	-1.26139155
H	-1.43784116	-3.91506378	-2.10837670
H	-2.78609085	-4.75446959	-1.28040065

Table S39. Frontier molecular orbitals for model *1* (square-planar Ni^{II}, *S* = 0).

No.	E (eV)	Ni 3d (%)	S 3p (%)	assignment	Ni/S_2 overlap
66a	− 1.51	0	0	pz π^*	
65a	− 1.73	2	0	pz π^*	
64a	− 2.21	0	22	ligand $4b_1$	
63a	− 3.41	47	36	Ni $3d_{x2-y2}$	σ^* (LUMO)
62a	− 4.96	70	19	Ni $3(d_{xz} - d_{yz})$	δ^* (HOMO)
61a	− 5.00	87	0	Ni $3d_z{}^2$	
60a	− 5.10	74	6	Ni $3(d_{xz} + d_{yz})$	π^*
59a	− 6.03	1	0	pz π (ax)	
58a	− 6.12	25	0	pz π (ax)	
57a	− 6.23	51	4	Ni $3d_{xy}$	
56a	− 6.24	6	60	ligand $2a_2$	δ
55a	− 6.59	4	5	pz π (eq)	
54a	− 6.67	5	15	pz π (eq)	
53a	− 6.77	9	20	ligand $3b_1$	π
52a	− 6.81	19	51	ligand $6b_2$	$\sigma_{+/-}$
51a	− 6.94	0	0	N_{ax} lp	
50a	− 7.02	14	16	pz π (eq)	
49a	− 7.12	18	0	pz π (eq)	
48a	− 7.89	12	59	ligand $7a_1$	$\sigma_{+/+}$
47a	− 8.47	0	0	σ(B–H)	

Table S40. Frontier molecular orbitals for model *2* (square-pyramidal Ni^{II}, *S* = 0).

No.	E (eV)	Ni 3d (%)	S 3p (%)	assignment	Ni/S2 overlap
27a″	− 1.11	1	1	pz π*	
39a′	− 1.38	1	1	pz π*	
38a′	− 2.12	0	24	ligand $4b_1$	
26a″	− 3.27	48	35	Ni $3d_{x2-y2}$	σ* (LUMO)
37a′	− 4.63	86	0	Ni $3d_{z}^{2}$	(HOMO)
25a″	− 4.77	75	17	Ni $3(d_{xz} – d_{yz})$	δ*
36a′	− 4.91	77	6	Ni $3(d_{xz} + d_{yz})$	π*
24a″	− 5.96	0	0	pz π (ax)	
35a′	− 5.98	75	4	Ni $3d_{xy}$	
23a″	− 6.13	1	12	pz π (ax)	
22a″	− 6.17	4	54	ligand $2a_2$	δ
21a″	− 6.54	5	5	pz π (eq)	
34a′	− 6.58	10	27	ligand $3b_1$	π
33a′	− 6.67	2	0	pz π (eq)	
20a″	− 6.69	19	51	ligand $6b_2$	$σ_{+/–}$
32a′	− 6.78	12	0	N_{ax} lp	
19a″	− 6.91	11	16	pz π (eq)	
31a′	− 7.14	13	0	pz π (eq)	
30a′	− 7.78	11	59	ligand $7a_1$	$σ_{+/+}$
29a′	− 8.32	0	0	σ(B–H)	

Table S41. Frontier molecular orbitals for model *3* (square-pyramidal Ni^{II}, *S* = 1).

α spin

**No.**	**E (eV)**	**Ni 3d (%)**	**S 3p (%)**	**assignment**	**Ni/S$_2$ overlap**
28a″	− 0.60	0	0	pz π*	
39a′	− 1.27	0	0	pz π*	
27a″	− 1.31	0	0	pz π*	
38a′	− 1.94	0	30	ligand $4b_1$ (α-spin LUMO)	
26a″	− 4.78	30	56	Ni $3d_{x2-y2}$ (α-spin HOMO)	σ*
25a″	− 4.77	17	77	Ni $3(d_{xz} - d_{yz})$	δ*
37a′	− 5.71	25	30	Ni $3(d_{xz} + d_{yz})$	π*
36a′	− 5.99	45	0	Ni $3d_z{}^2$	
24a″	− 6.10	27	20	ligand $2a_2$	δ
23a″	− 6.12	0	0	pz π	
35a′	− 5.98	65	18	Ni $3d_{xy}$	
22a″	− 6.45	0	0	pz π	
34a′	− 6.64	21	3	pz π	
21a″	− 6.65	4	5	pz π	
33a′	− 6.80	44	7	ligand $3b_1$	π
20a″	− 6.94	23	27	ligand $6b_2$	$σ_{+/-}$
32a′	− 7.03	38	5	pz π	
19a″	− 7.28	52	5	pz π	
31a′	− 7.78	25	40	ligand $7a_1$	$σ_{+/+}$
29a′	− 8.47	0	0	σ (B–H)	

Table S41. continued.

β spin

**No.**	**E (eV)**	**Ni 3d (%)**	**S 3p (%)**	**assignment**	**Ni/S_2 overlap**
28a″	− 0.57	0	0	pz π^*	
39a′	− 1.23	0	0	pz π^*	
27a″	− 1.26	1	0	pz π^*	
38a′	− 1.89	0	32	ligand $4b_1$	
26a″	− 3.10	59	28	Ni $3d_{x2-y2}$	σ^*
37a′	− 3.94	75	2	Ni $3d_{z^2}$ (β-spin LUMO)	
25a″	− 5.26	51	37	Ni $3(d_{xz} - d_{yz})$(β-spin HOMO)	δ^*
36a′	− 5.42	67	9	Ni $3(d_{xz} + d_{yz})$	π^*
24a″	− 5.78	11	71	ligand $2a_2$	δ
35a′	− 5.82	76	12	Ni $3d_{xy}$	
23a″	− 6.11	0	0	pz π	
34a′	− 6.27	25	28	ligand $3b_1$	π
22a″	− 6.28	31	48	ligand $6b_2$	$\sigma_{+/-}$
21a″	− 6.43	0	0	pz π	
33a′	− 6.62	8	0	pz π	
20a″	− 6.67	7	0	pz π	
32a′	− 6.82	8	5	pz π	
19a″	− 7.00	21	3	pz π	
31a′	− 7.24	12	64	ligand $7a_1$	$\sigma_{+/+}$
30a′	− 8.46	0	0	σ (B–H)	

Table S42. Frontier molecular orbitals for model *4* (trigonal-bipyramidal Ni^{II}, S = 0).

No.	E (eV)	Ni 3d (%)	S 3p (%)	assignment		Ni/S$_2$ overlap
41a′	− 1.11	1	0	pz π^*		
25a″	− 1.26	1	0	pz π^*		
24a″	− 1.98	1	28	ligand 4b$_1$		
40a′	− 3.41	48	36	Ni 3d$_{z2}$	(LUMO)	σ^*
23a″	− 3.97	74	4	Ni 3d$_{xy}$	(HOMO)	σ^*/π^* (N_{eq}/S_{eq})
39a′	− 4.44	84	4	Ni 3d$_{x2-y2}$		
22a″	− 4.90	72	12	Ni 3d$_{yz}$		π^* (S_{ax})
38a′	− 5.86	80	2	Ni d$_{xz}$		
21a″	− 6.00	4	70	ligand 2a$_2$		δ
20a″	− 6.05	0	2	pz π (eq)		
19a″	− 6.32	0	7	pz π (eq)		
18a″	− 6.48	6	28	ligand 3b$_1$		π
37a′	− 6.50	6	0	pz π (eq)		
36a′	− 6.65	15	19	pz π (eq)		
17a″	− 6.65	4	2	pz π (ax)		
35a′	− 7.92	17	37	ligand 6b$_2$		$\sigma_{+/-}$
16a″	− 8.24	13	8	pz π (ax)		
34a′	− 7.73	14	54	ligand 7a$_1$		$\sigma_{+/+}$
15a″	− 8.15	17	0	σ(N_{eq}–Ni)		
33a′	− 8.39	0	0	σ(B–H)		

Table S43. Frontier molecular orbitals for model *5* (trigonal-bipyramidal Ni^{II}, S = 1).

α spin

**No.**	**E (eV)**	**Ni 3d (%)**	**S 3p (%)**	**assignment**	**Ni/S_2 overlap**
25a″	− 1.28	0	0	pz π*	
41a′	− 1.31	0	0	pz π*	
24a″	− 1.95	0	28	ligand $4b_1$ (α-spin LUMO)	
40a′	− 4.54	29	46	Ni $3d_{z^2}$ (α-spin HOMO)	σ*
23a″	− 5.67	17	75	Ni $3d_{yz}$	δ*
22a″	− 5.73	26	28	Ni $3d_{xy}$	σ*/π* (N_{eq}/S_{eq})
39a′	− 5.94	48	7	Ni $3d_{x2-y2}$	
21a″	− 6.12	4	1	pz π	
20a″	− 6.13	24	16	ligand $2a_2$	π*/π* (N_{ax}/S_{ax})
19a″	− 6.45	0	0	pz π	
38a′	− 6.49	55	15	Ni $3d_{xz}$	
18a″	− 6.59	17	9	ligand $3b_1$	π
37a′	− 6.67	10	3	pz π	
17a″	− 6.74	28	5	pz π	
36a′	− 6.98	28	32	ligand $6b_2$	$σ_{+/-}$
35a′	− 7.10	43	2	pz π	
16a″	− 7.18	38	4	pz π	
34a′	− 7.68	40	39	ligand $7a_1$	$σ_{+/+}$
33a′	− 8.48	0	0	σ (B–H)	

Table S43. continued.

β spin

**No.**	**E (eV)**	**Ni 3d (%)**	**S 3p (%)**	**assignment**	**Ni/S$_2$ overlap**
25a″	− 1.23	1	0	pz π*	
41a′	− 1.27	0	0	pz π*	
24a″	− 1.89	0	29	ligand 4b$_1$	
40a′	− 3.17	59	28	Ni 3d$_{z2}$	σ*
23a″	− 4.03	75	4	Ni 3d$_{xy}$ (β-spin LUMO)	σ*/π* (N$_{eq}$/S$_{eq}$)
22a″	− 5.29	59	26	Ni 3d$_{yz}$ (β-spin HOMO)	π*/π*(N$_{ax}$/S$_{ax}$)
39a′	− 5.31	61	20	Ni 3d$_{x2-y2}$	
21a″	− 5.84	13	71	ligand 2a$_2$	δ
38a′	− 5.86	83	6	Ni 3d$_{xz}$	
20a″	− 6.11	0	0	pz π	
19a″	− 6.22	14	34	ligand 3b$_1$	π
37a′	− 6.30	30	51	ligand 6b$_2$	σ$_{+/-}$
18a″	− 6.44	0	0	pz π	
17a″	− 6.65	7	0	pz π	
36a′	− 6.66	8	0	pz π	
35a′	− 6.88	11	7	pz π	
16a″	− 6.91	12	3	pz π	
34a′	− 7.37	26	48	ligand 7a$_1$	σ$_{+/+}$
33a′	− 8.47	0	0	σ (B–H)	

Table S44. Frontier molecular orbitals for model *6* (square-pyramidal Ni^{III}, *S* = ½).

α spin

**No.**	**E (eV)**	**Ni 3d (%)**	**S 3p (%)**	**assignment**	**Ni/S_2 overlap**
39 a′	− 1.73	1	0	pz π*	
38 a′	− 2.60	0	25	ligand $4b_1$	
26 a″	− 4.87	33	51	Ni $3d_{x2-y2}$ (α-spin LUMO)	σ*
25 a″	− 6.42	17	65	ligand $2a_2$ (α-spin HOMO)	δ*
24 a″	− 6.44	0	0	pz π	
37 a′	− 6.45	28	22	Ni $3d_z^2$	
36 a′	− 6.94	18	7	pz π	
23 a″	− 6.67	4	21	pz π	
22 a″	− 6.86	0	5	pz π	
35 a′	− 7.03	6	5	pz π	
21 a″	− 7.04	2	1	pz π	
34 a′	− 7.37	27	15	ligand $3b_1$	π*
20 a″	− 7.74	20	35	ligand $6b_2$	$σ_{+/-}$
33 a′	− 7.93	53	15	Ni $3(d_{xz} + d_{yz})$	π
32 a′	− 8.32	73	2	Ni $3d_{xy}$	
19 a″	− 8.34	71	8	Ni $3(d_{xz} - d_{yz})$	δ
31 a′	− 8.83	31	31	ligand $7a_1$	$σ_{+/+}$
30 a′	− 8.89	0	0	σ (B–H)	

Table S44. continued.

β spin

**No.**	**E (eV)**	**Ni 3d (%)**	**S 3p (%)**	**assignment**		**Ni/S_2 overlap**
39 a′	− 1.70	1	0	pz π*		
38 a′	− 2.56	0	25	ligand $4b_1$		
26 a″	− 4.51	42	41	Ni $3d_{x2-y2}$		σ*
37 a′	− 5.28	63	5	Ni $3d_z{}^2$	(β-spin LUMO)	
25 a″	− 6.31	27	50	ligand $2a_2$	(β-spin HOMO)	δ*
24 a″	− 6.43	0	0	pz π		
36 a′	− 6.47	28	15	ligand $3b_1$		π*
23 a″	− 6.62	3	30	pz π		
22 a″	− 6.84	0	1	pz π		
35 a′	− 6.94	14	8	pz π		
21 a″	− 7.02	2	1	pz π		
34 a′	− 7.17	12	7	pz π		
20 a″	− 7.63	19	42	ligand $6b_2$		$\sigma_{+/-}$
33 a′	− 7.66	61	14	Ni $3(d_{xz} + d_{yz})$		π
32 a′	− 7.96	66	0	Ni $3d_{xy}$		
19 a″	− 8.09	64	15	Ni $3(d_{xz} - d_{yz})$		δ
31 a′	− 8.73	25	41	ligand $7a_1$		$\sigma_{+/+}$
30 a′	− 8.88	0	0	σ (B–H)		

Table S45. Frontier molecular orbitals for model *7* (trigonal-bipyramidal Ni^{III}, S = ½).

α spin

**No.**	**E (eV)**	**Ni 3d (%)**	**S 3p (%)**	**assignment**	**Ni/S_2 overlap**
25 a″	− 1.73	0	0	pz π*	
24 a″	− 2.62	0	25	ligand $4b_1$	
40 a′	− 4.92	30	50	Ni $3d_{z^2}$ (α-spin LUMO)	σ*
23 a″	− 6.41	28	25	Ni $3d_{xy}$ (α-spin HOMO)	σ*/π*(N_{eq}/S_{eq})
22 a″	− 6.43	0	0	pz π	
21 a″	− 6.49	18	49	ligand $3b_1$	π* (N_{ax}/S_{ax})
39 a′	− 6.53	25	4	pz π	
20 a″	− 6.73	3	30	ligand $2a_2$	δ
19 a″	− 6.85	0	10	pz π	
38 a′	− 6.98	6	3	pz π	
18 a″	− 7.06	2	3	pz π	
37 a′	− 7.42	33	17	Ni $3d_{x2-y2}$	
17 a″	− 7.64	25	15	pz π	
36 a′	− 7.92	30	38	ligand $6b_2$	$\sigma_{+/-}$
16 a″	− 8.24	67	6	Ni $3d_{yz}$	π (N_{ax}/S_{ax})
35 a′	− 8.32	78	4	Ni $3d_{xz}$	
34 a′	− 8.88	3	0	σ (B–H)	
33 a′	− 8.96	36	33	ligand $7a_1$	$\sigma_{+/+}$

Table S45. continued.

β spin

**No.**	**E (eV)**	**Ni 3d (%)**	**S 3p (%)**	**assignment**	**Ni/S_2 overlap**
25 a″	− 1.70	0	0	pz π*	
24 a″	− 2.56	0	25	ligand $4b_1$	
40 a′	− 4.58	42	40	Ni $3d_{z2}$	σ*
23 a″	− 5.31	58	8	Ni $3d_{xy}$ (β-spin LUMO)	σ*/π* (N_{eq}/S_{eq})
22 a″	− 6.35	31	36	ligand $3b_1$ (β-spin HOMO)	π*
39 a′	− 6.37	36	7	pz π	
21 a″	− 6.42	0	0	pz π	
20 a″	− 6.66	2	45	ligand $2a_2$	δ
19 a″	− 6.82	0	11	pz π	
38 a′	− 6.95	8	4	pz π	
18 a″	− 7.02	2	7	pz π	
37 a′	− 7.24	32	11	Ni $3d_{x2-y2}$	
17 a″	− 7.35	11	21	pz π	
36 a′	− 7.78	39	31	ligand $6b_2$	$σ_{+/-}$
16 a″	− 7.94	56	8	Ni $3d_{yz}$	π (N_{ax}/S_{ax})
35 a′	− 8.00	64	13	Ni $3d_{xz}$	
34 a′	− 8.84	14	20	ligand $7a_1$	$σ_{+/+}$
33 a′	− 8.90	7	19	σ (B–H)	

Table S46. Frontier molecular orbitals for model *8* (square-planar Ni^{II}, *S* = 0).

No.	E (eV)	Ni 3d (%)	S 3p (%)	assignment	Ni/S_2 overlap
24a″	− 0.94	0	0	pz π^*	
30a′	− 1.33	1	3	pz π^*	
29a′	− 2.18	2	22	ligand $4b_1$	
23a″	− 3.33	45	36	Ni $3d_{x2-y2}$	σ^* (LUMO)
22a″	− 4.89	73	18	Ni $3(d_{xz} - d_{yz})$	δ^* (HOMO)
28a′	− 4.93	89	0	Ni $3d_{z}^{2}$	
27a′	− 5.03	75	7	Ni $3(d_{xz} + d_{yz})$	π^*
26a′	− 6.05	64	2	Ni $3d_{xy}$	
21a″	− 6.18	4	56	ligand $2a_2$	δ
25a′	− 6.21	11	0	pz π	
20a″	− 6.41	4	7	pz π	
24a′	− 6.69	11	35	ligand $3b_1$	π
19a″	− 6.73	19	46	ligand $6b_2$	$\sigma_{+/-}$
18a″	− 6.92	16	27	pz π	
23a′	− 6.99	22	0	pz π	
22a′	− 7.80	7	46	ligand $7a_1$	$\sigma_{+/+}$
21a′	− 7.84	2	8	$\sigma(BH_2)$	
20a′	− 8.83	0	0	$\sigma(BH_2)$	

Table S47. Frontier molecular orbitals for model *9* (square-planar "Ni^{III}", $S = ½$).

α spin

No.	E (eV)	Ni 3d (%)	S 3p (%)	assignment	Ni/S_2 overlap
30a′	− 1.80	3	3	pz π*	
29a′	− 2.81	2	19	ligand $4b_1$	
23a″	− 4.63	38	41	Ni $3d_{x2-y2}$ (α-spin LUMO)	σ*
28a′	− 6.46	33	12	ligand $3b_1$ (α-spin HOMO)	π*
27a′	− 6.59	4	2	pz π	
22a″	− 6.65	10	11	pz π	
21a″	− 6.87	9	51	ligand $2a_2$	δ*
26a′	− 7.01	84	4	Ni $3d_z{}^2$	
20a″	− 7.02	0	23	pz π	
25a′	− 7.25	16	14	pz π	
24a′	− 7.84	58	9	Ni $3(d_{xz} + d_{yz})$	π
19a″	− 7.85	33	12	ligand $6b_2$	$σ_{+/-}$
23a′	− 8.09	27	3	σ (BH_2)	
22a′	− 8.21	34	4	Ni $3d_{xy}$	
18a″	− 8.58	77	12	Ni $3(d_{xz} + d_{yz})$	δ
21a′	− 8.86	22	41	ligand $7a_1$	$σ_{+/+}$
20a′	− 9.18	4	0	σ (BH_2)	

Table S47. continued.

β spin

**No.**	**E (eV)**	**Ni 3d (%)**	**S 3p (%)**	**assignment**	**Ni/S$_2$ overlap**
30a′	− 1.75	3	3	pz π*	
29a′	− 2.76	0	18	ligand 4b$_1$	
23a″	− 4.39	39	38	Ni 3d$_{x2-y2}$	σ*
22a″	− 6.10	44	37	ligand 2a$_2$ (β-spin LUMO)	δ*
28a′	− 6.24	43	14	ligand 3b$_1$ (β-spin HOMO)	π*
27a′	− 6.57	0	0	pz π	
21a″	− 6.66	0	24	pz π	
26a′	− 6.75	85	4	Ni 3d$_{z}{}^{2}$	
20a″	− 6.92	2	12	pz π	
25a′	− 7.13	15	13	pz π	
24a′	− 7.64	53	13	Ni 3(d$_{xz}$ + d$_{yz}$)	π
19a″	− 7.70	42	34	Ni 3(d$_{xz}$ − d$_{yz}$)	δ
18a″	− 7.77	26	42	ligand 6b$_2$	σ$_{+/-}$
23a′	− 7.93	58	4	Ni 3d$_{xy}$	
22a′	− 8.15	6	0	σ (BH$_2$)	
21a′	− 8.80	10	45	ligand 7a$_1$	σ$_{+/+}$
20a′	− 9.16	2	0	σ (BH$_2$)	

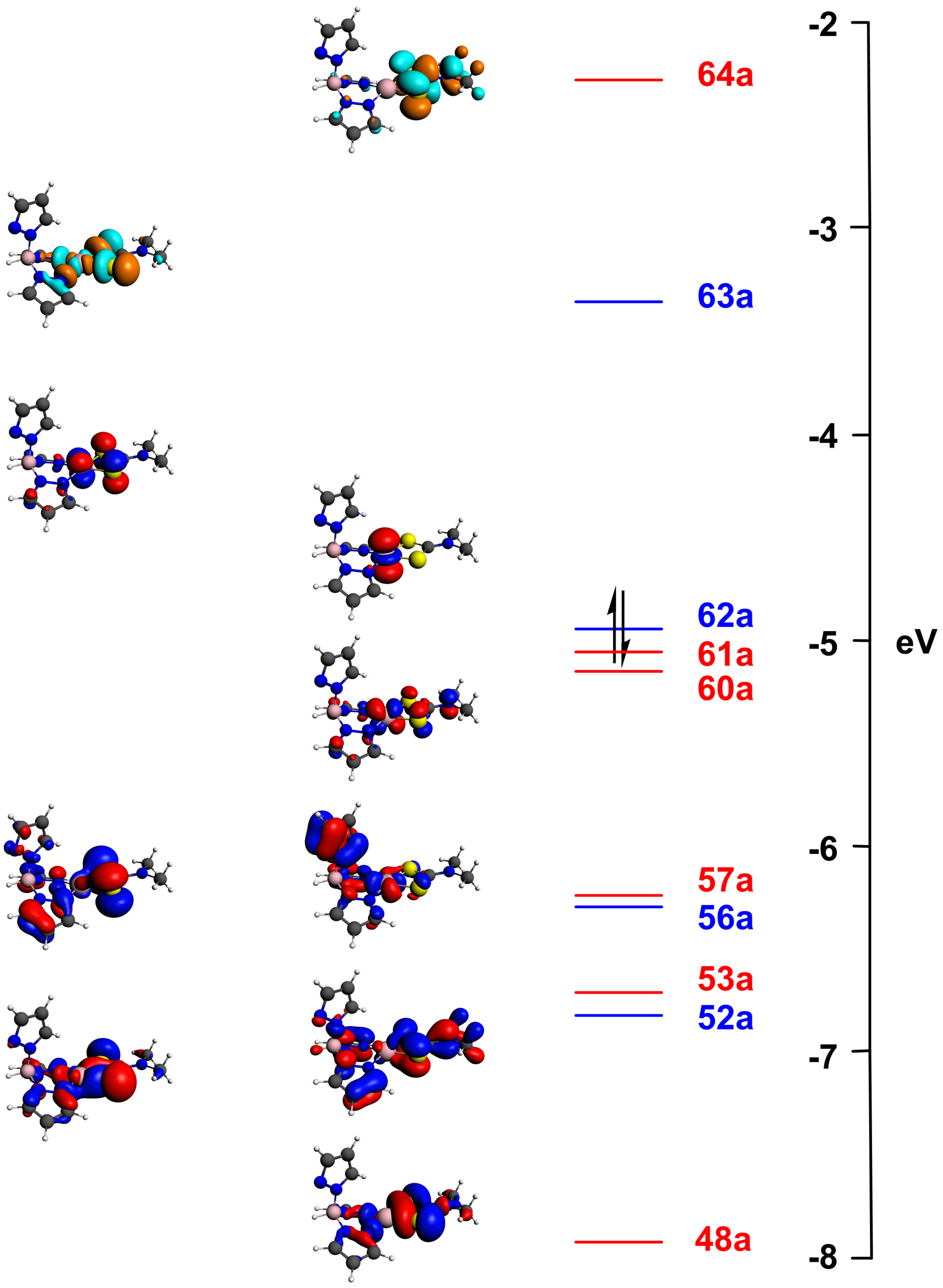

Figure S20. Relative energies of select Ni 3d- and S 3p-based frontier orbitals (Table S39) for model *1*, color-coded by implicit mirror symmetry (right), and corresponding isocontour plots (left).

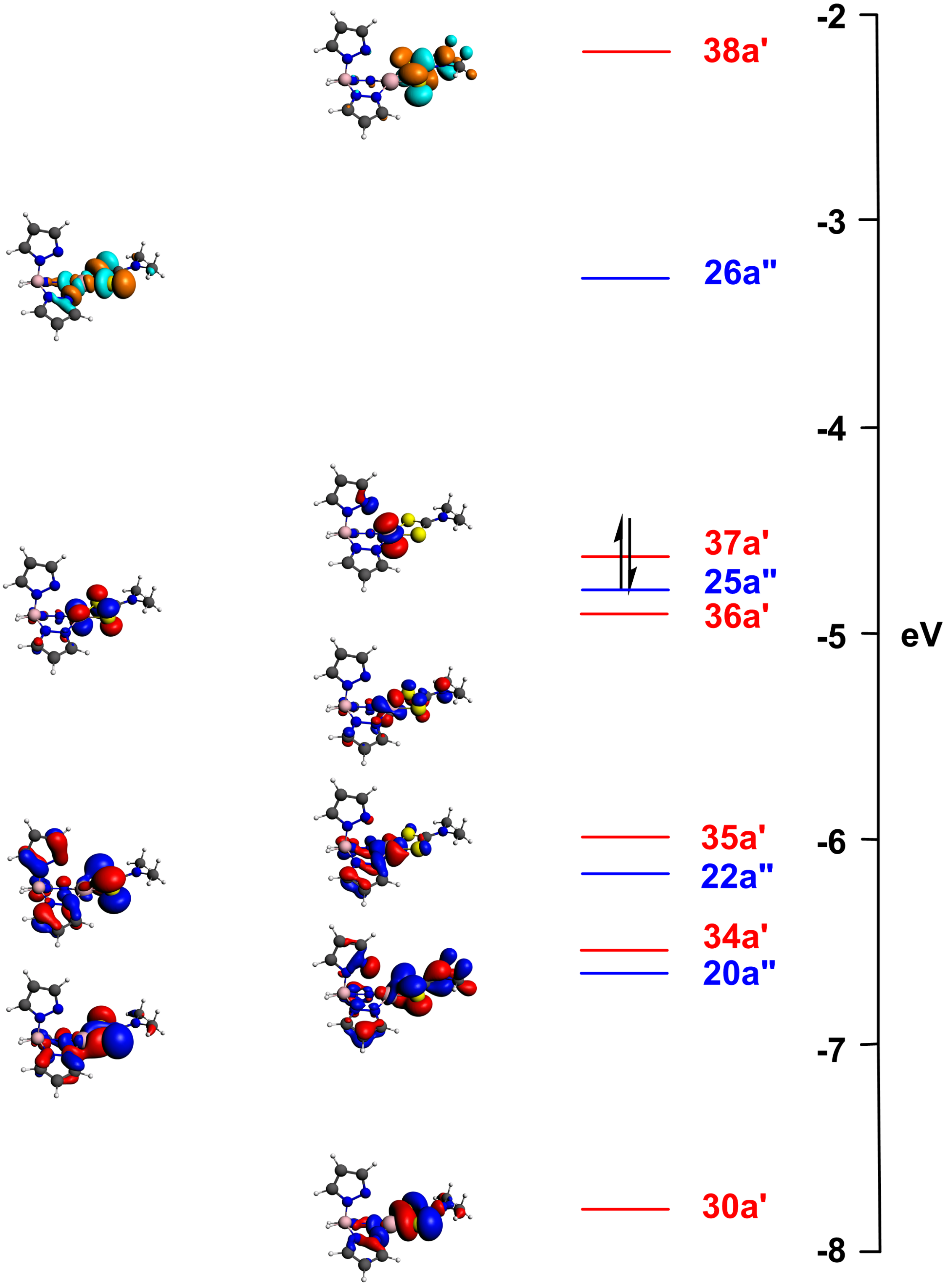

Figure S21. Relative energies of select Ni 3d- and S 3p-based frontier orbitals (Table S40) for model *2*, color-coded by mirror symmetry (right), and corresponding isocontour plots (left).

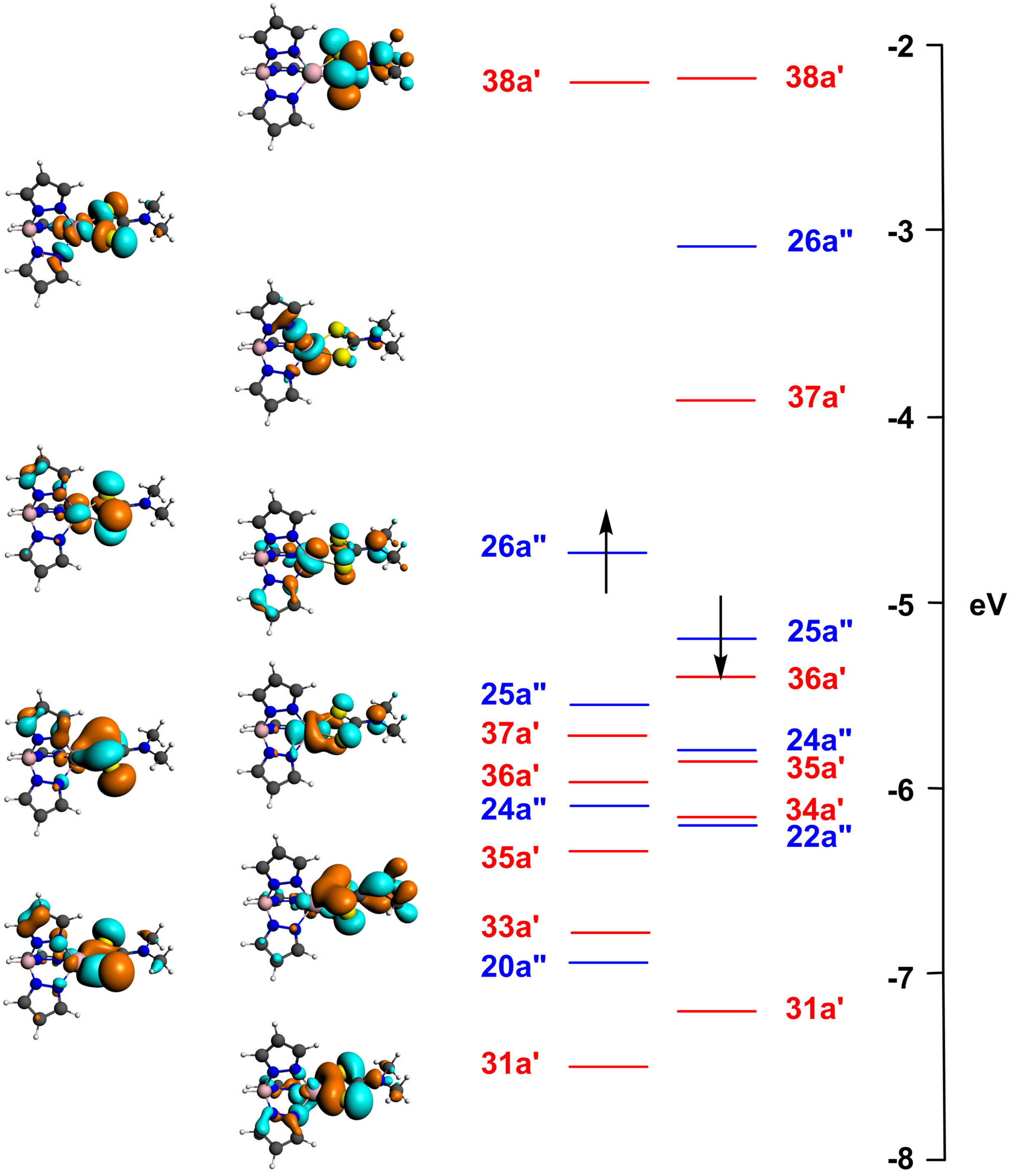

Figure S22. Relative energies of select Ni 3d- and S 3p-based frontier orbitals (Table S41) for model *3*, color-coded by mirror symmetry (right), and corresponding isocontour plots (left).

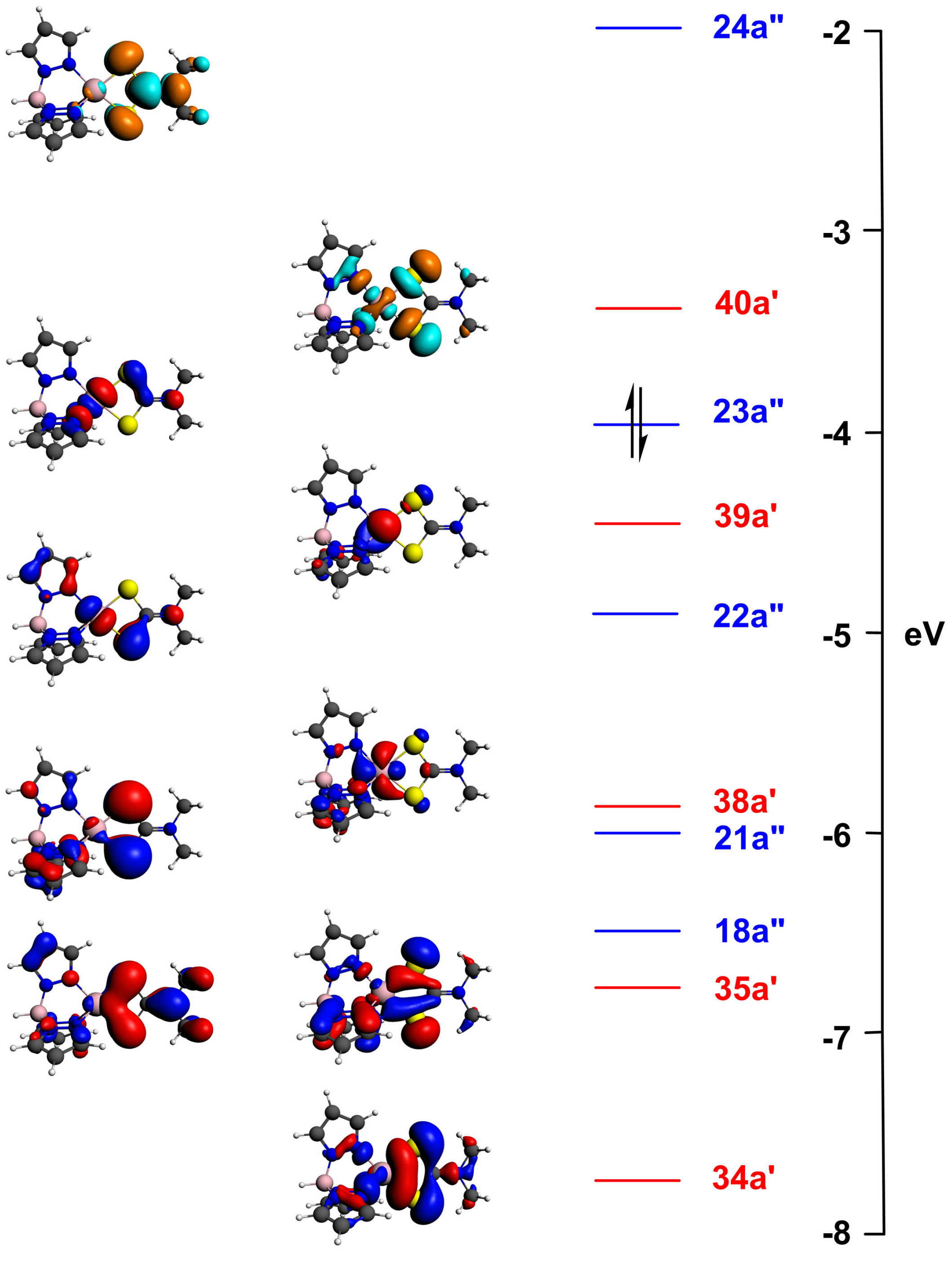

Figure S23. Relative energies of select Ni 3d- and S 3p-based frontier orbitals (Table S42) for model *4*, color-coded by mirror symmetry (right), and corresponding isocontour plots (left).

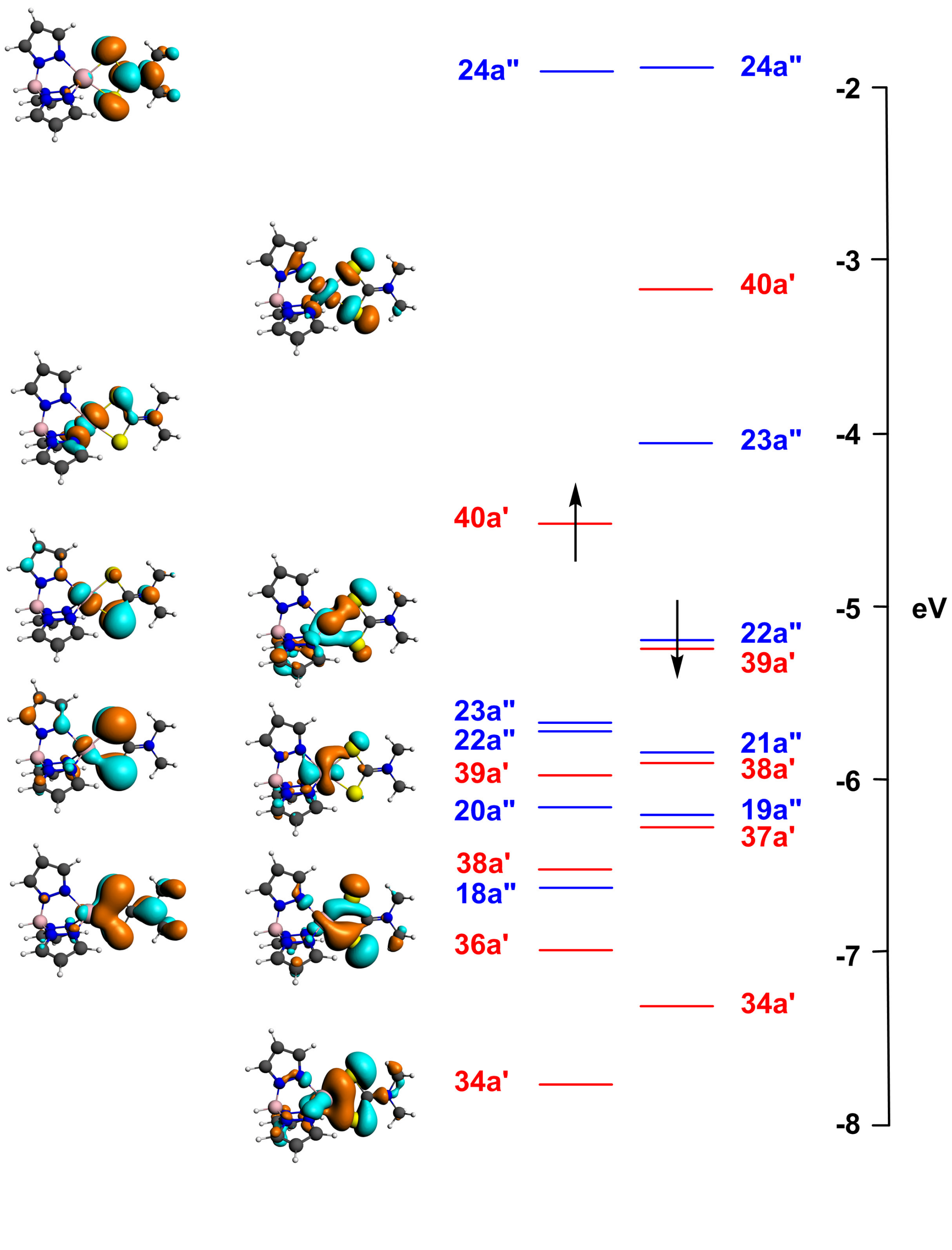

Figure S24. Relative energies of select Ni 3d- and S 3p-based frontier orbitals (Table S43) for model *5*, color-coded by mirror symmetry (right), and corresponding isocontour plots (left).

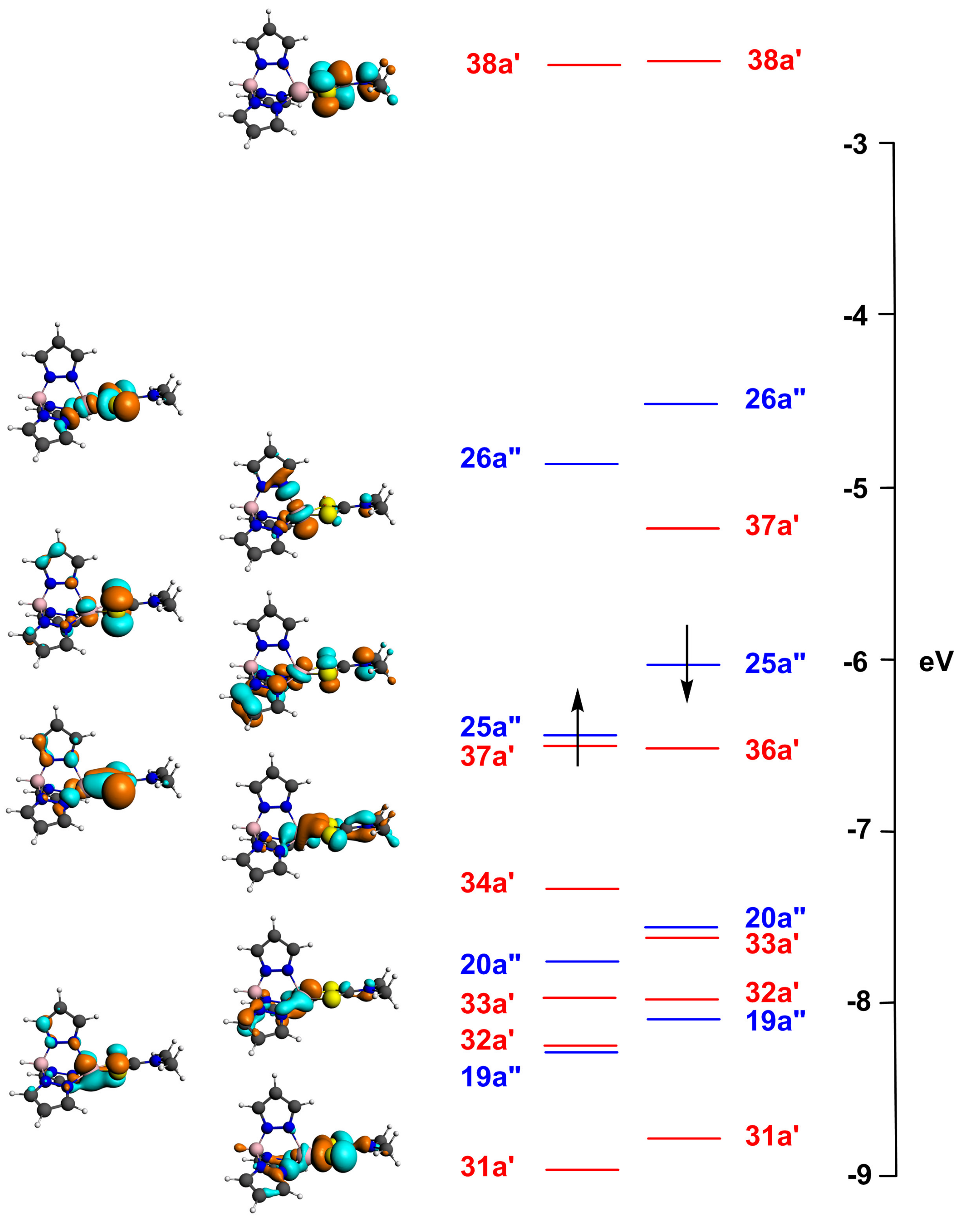

Figure S25. Relative energies of select Ni 3d- and S 3p-based frontier orbitals (Table S44) for model *6*, color-coded by mirror symmetry (right), and corresponding isocontour plots (left).

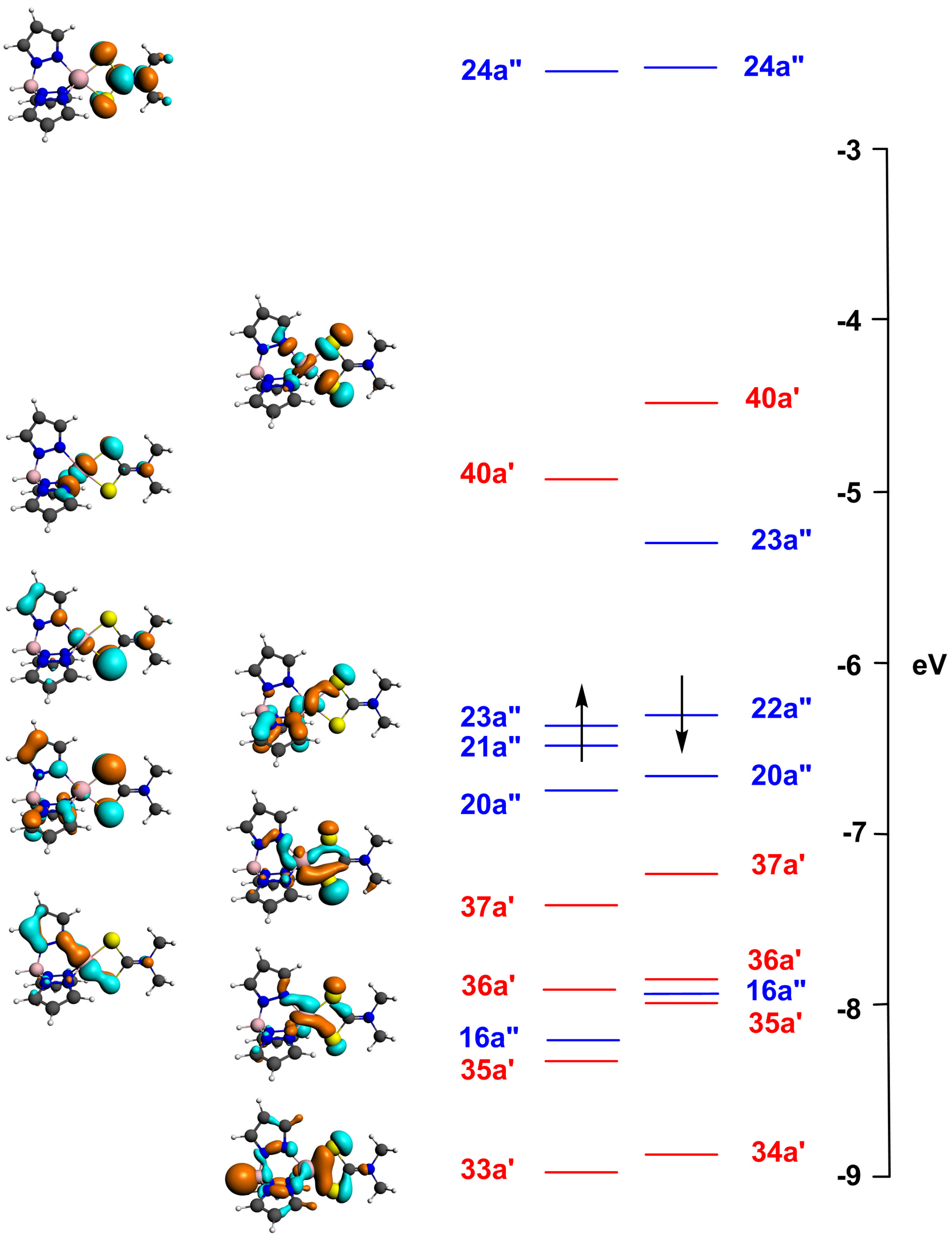

Figure S26. Relative energies of select Ni 3d- and S 3p-based frontier orbitals (Table S45) for model *7*, color-coded by mirror symmetry (right), and corresponding isocontour plots (left).

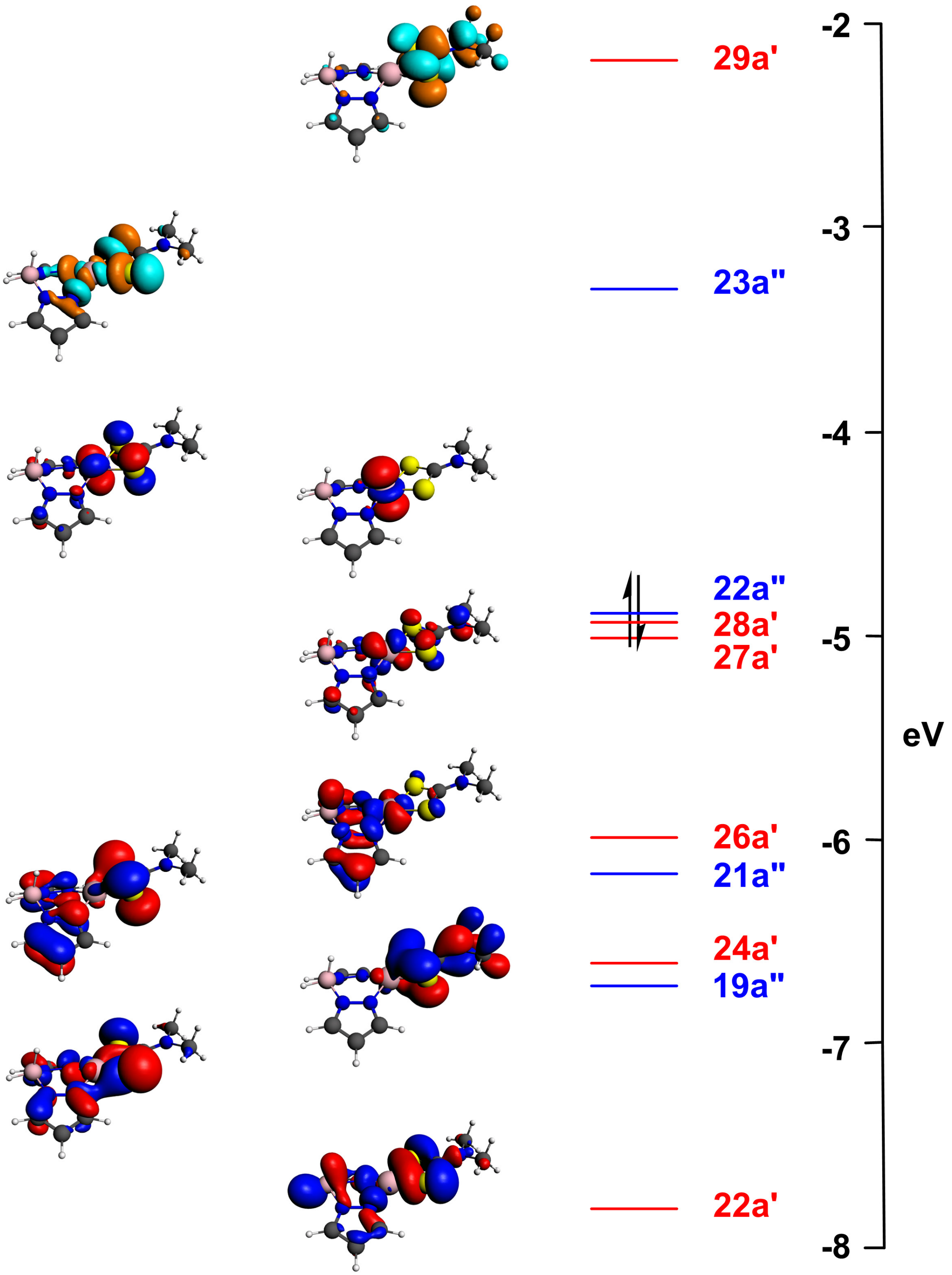

Figure S27. Relative energies of select Ni 3d- and S 3p-based frontier orbitals (Table S46) for model *8*, color-coded by mirror symmetry (right), and corresponding isocontour plots (left).

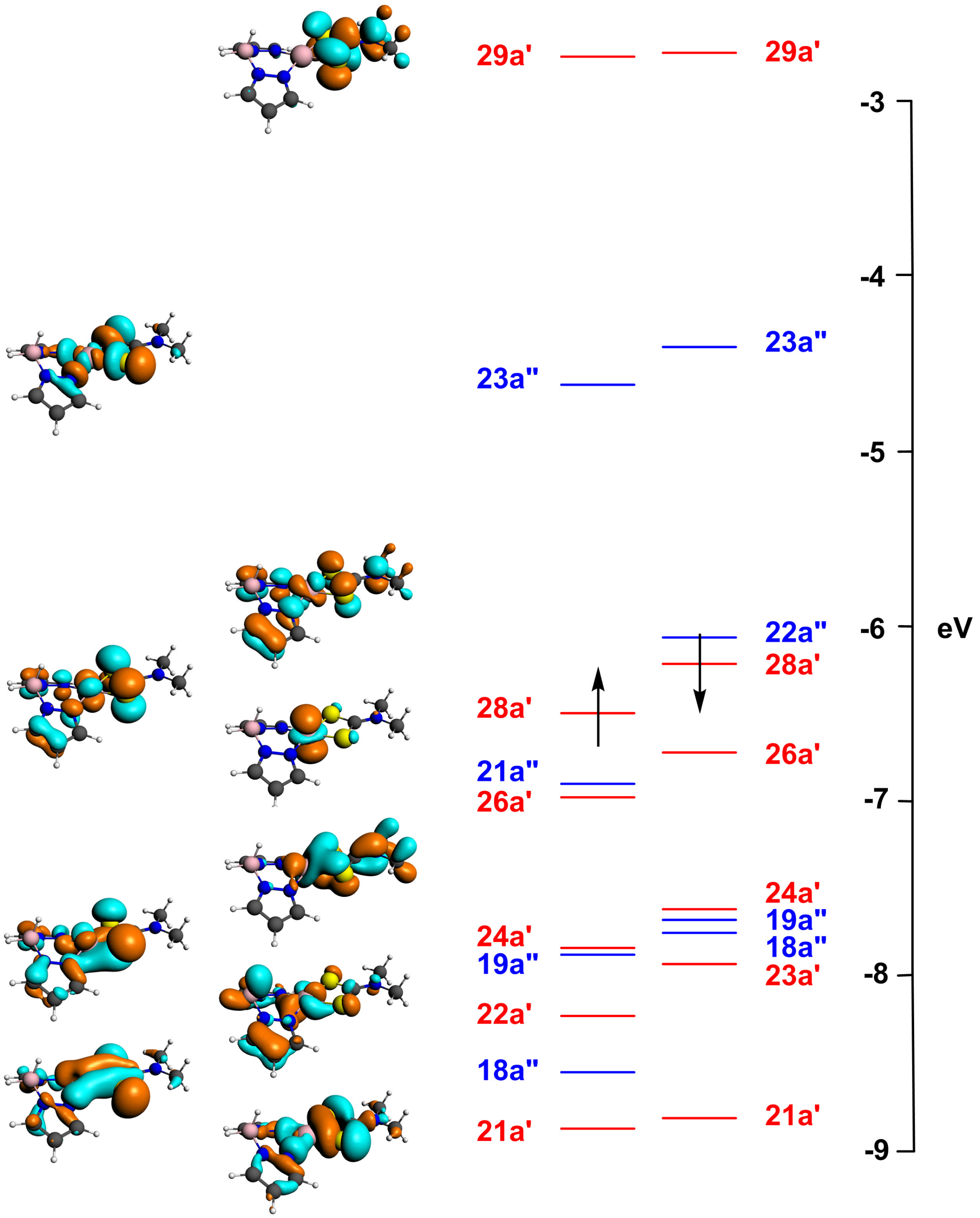

Figure S28. Relative energies of select Ni 3d- and S 3p-based frontier orbitals (Table S47) for model *9*, color-coded by mirror symmetry (right), and corresponding isocontour plots (left).

Table S48. Calculated electronic excitations for the synthetic model *1.*

No.	E/eV	f	orbitals	fraction	assignment
1A	1.983	0.0003	62a → 63a	0.975	LF
2A	2.107	0.0001	60a → 63a	0.870	LF
			61a → 63a	0.109	LF
3A	2.202	0.0004	61a → 63a	0.862	LF
			60a → 63a	0.111	LF
4A	2.632	0.0005	59a → 63a	0.992	LMCT (pz)
5A	2.746	0.0003	58a → 63a	0.837	LMCT (pz)
			57a → 63a	0.127	LF
6A	2.821	0.0021	61a → 64a	0.732	Ni d → $4b_1$ π^*
			62a → 64a	0.232	Ni d → $4b_1$ π^*
7A	2.832	0.0004	62a → 64a	0.718	Ni d → $4b_1$ π^*
			61a → 64a	0.231	Ni d → $4b_1$ π^*
8A	2.963	0.0005	56a → 63a	0.885	$2a_2$ δ → Ni d_σ^*
9A	2.988	0.0010	57a → 63a	0.775	LF
			58a → 63a	0.141	LMCT (pz)
10A	3.081	0.0531	60a → 64a	0.932	Ni d → $4b_1$ π^*
11A	3.227	0.0005	55a → 63a	0.948	LMCT (pz)
12A	3.295	0.0001	54a → 63a	0.871	LMCT (pz)
			53a → 63a	0.112	$3b_1$ π → Ni d_σ^*
13A	3.454	0.0003	53a → 63a	0.836	$3b_1$ π → Ni d_σ^*
14A	3.528	0.0036	61a → 65a	0.443	MLCT (pz)
			62a → 65a	0.284	MLCT (pz)
			51a → 63a	0.245	LMCT (pz)
15A	3.531	0.0013	51a → 63a	0.740	LMCT (pz)
			61a → 65a	0.134	MLCT (pz)
			62a → 65a	0.103	MLCT (pz)
16A	3.538	0.0087	62a → 65a	0.547	MLCT (pz)
			61a → 65a	0.397	MLCT (pz)
17A	3.703	0.0006	60a → 65a	0.416	MLCT (pz)
			50a → 63a	0.397	LMCT (pz)

Table S48. continued.

No.	E/eV	f	orbitals	fraction	assignment
18A	3.727	0.0114	60a → 65a	0.379	MLCT (pz)
			50a → 63a	0.339	LMCT (pz)
			62a → 66a	0.136	MLCT (pz)
			52a → 63a	0.109	$6b_2$ σ → Ni d_σ*
19A	3.811	0.0002	49a → 63a	0.887	LMCT (pz)
20A	3.830	0.0008	59a → 64a	0.995	Interligand
21A	3.870	0.0005	61a → 66a	0.913	MLCT (pz)
22A	3.916	0.0107	58a → 64a	0.840	Interligand
			62a → 66a	0.130	MLCT (pz)
23A	3.948	0.0426	62a → 66a	0.594	MLCT (pz)
			58a → 64a	0.141	Interligand
			60a → 65a	0.127	MLCT (pz)
24A	4.005	0.0118	60a → 66a	0.930	MLCT (pz)
25A	4.043	0.0046	57a → 64a	0.921	Ni d → $4b_1$ π*
26A	4.239	0.0151	56a → 64a	0.735	Interligand
			55a → 64a	0.102	Interligand
27A	4.344	0.0105	62a → 67a	0.843	MLCT (pz)
			61a → 67a	0.130	MLCT (pz)
28A	4.380	0.0443	61a → 67a	0.788	MLCT (pz)
29A	4.424	0.0024	55a → 64a	0.844	Interligand
30A	4.434	0.0044	62a → 68a	0.971	MLCT (pz)
31A	4.465	0.1462	61a → 68a	0.530	MLCT (pz)
			52a → 63a	0.245	$6b_2$ σ → Ni d_σ*
32A	4.477	0.0044	60a → 67a	0.874	MLCT (pz)
33A	4.506	0.0111	54a → 64a	0.458	Interligand
			53a → 64a	0.279	Intraligand
			61a → 68a	0.141	MLCT (pz)
34A	4.535	0.1638	52a → 63a	0.259	$6b_2$ σ → Ni d_σ*
			61a → 68a	0.176	MLCT (pz)
			54a → 64a	0.134	Interligand

Table S49. Calculated electronic excitations for the synthetic model *2*.

No.	E/eV	f	orbitals	fraction	assignment
1A"	1.945	0.0004	37a' → 26a"	0.979	LF
1A'	1.948	0.0005	25a" → 26a"	0.986	LF
2A"	2.080	0.0001	36a' → 26a"	0.973	LF
2A'	2.540	0.0031	37a' → 38a'	0.988	Ni d → $4b_1$ π^*
3A'	2.702	0.0001	24a" → 26a"	0.999	LMCT (pz)
3A"	2.725	0.0000	25a" → 38a'	0.965	Ni d → $4b_1$ π^*
4A'	2.870	0.0008	23a" → 26a"<br>22a" → 26a"	0.871<br>0.126	LMCT (pz)<br>$2a_2$ δ → Ni d_σ^*
4A"	2.908	0.0014	35a' → 26a"	0.956	LF
5A'	2.974	0.0416	36a' → 38a'	0.923	Ni d → $4b_1$ π^*
6A'	3.022	0.0045	22a" → 26a"<br>23a" → 26a"	0.816<br>0.119	$2a_2$ δ → Ni d_σ^*<br>LMCT (pz)
7A'	3.281	0.0012	37a' → 39a'	0.954	MLCT (pz)
8A'	3.319	0.0007	21a" → 26a"	0.903	LMCT (pz)
5A"	3.377	0.0000	34a' → 26a"<br>33a' → 26a"	0.705<br>0.274	$3b_1$ π → Ni d_σ^*<br>LMCT (pz)
6A"	3.449	0.0001	33a' → 26a"<br>34a' → 26a"	0.697<br>0.252	LMCT (pz)<br>$3b_1$ π → Ni d_σ^*
7A"	3.459	0.0115	25a" → 39a'	0.934	MLCT (pz)
8A"	3.526	0.0007	37a' → 27a"	0.950	MLCT (pz)
9A"	3.589	0.0001	32a' → 26a"	0.875	LMCT (pz)
9A'	3.622	0.0015	36a' → 39a'<br>25a" → 27a"	0.649<br>0.314	MLCT (pz)<br>MLCT (pz)
10A'	3.715	0.0014	19a" → 26a"<br>20a" → 26a"	0.692<br>0.192	LMCT (pz)<br>$6b_2$ σ → Ni d_σ^*
11A'	3.812	0.0541	25a" → 27a"<br>36a' → 39a'	0.546<br>0.284	MLCT (pz)<br>MLCT (pz)

Table S49. continued.

No.	E/eV	f	orbitals	fraction	assignment
10A"	3.845	0.0110	36a' → 27a"	0.958	MLCT (pz)
11A"	3.850	0.0000	24a" → 38a'	0.994	interligand
12A'	3.892	0.0061	35a' → 38a'	0.927	Ni d → $4b_1$ π^*
12A"	3.941	0.0000	31a' → 26a"	0.972	LMCT (pz)
13A"	4.018	0.0001	23a" → 38a'	0.858	interligand
			22a" → 38a'	0.140	intraligand
14A"	4.17379	0.0001	37a' → 28a"	0.997	MLCT (pz)
13A'	4.18979	0.0029	37a' → 40a'	0.967	MLCT (pz)
15A"	4.26975	0.0116	22a" → 38a'	0.638	intraligand
			21a" → 38a'	0.120	interligand
16A"	4.32370	0.0000	25a" → 40a'	0.987	MLCT (pz)
14A'	4.33293	0.0211	25a" → 28a"	0.947	MLCT (pz)
17A"	4.45330	0.0031	36a' → 28a"	0.921	MLCT (pz)
15A'	4.46034	0.0162	36a' → 40a'	0.911	MLCT (pz)
18A"	4.46698	0.0019	21a" → 38a'	0.770	interligand
16A'	4.50833	0.3354	20a" → 26a"	0.526	$6b_2$ σ → Ni d_σ^*
			19a" → 26a"	0.158	LMCT (pz)

Table S50. Calculated electronic excitations for the synthetic model *3*.

No.	E/eV	f	orbitals	fraction	assignment
1A″	1.288	0.0001	25a" → 37a' β	0.992	LF
1A′	1.546	0.0064	36a' → 37a' β	0.908	LF
2A″	1.856	0.0005	24a" → 37a' β	0.992	$2a_2$ δ → Ni d_{z2}
2A′	1.986	0.0011	35a' → 37a' β	0.854	LF
3A"	2.171	0.0002	23a" → 37a' β	1.000	LMCT (pz)
3A′	2.280	0.0060	25a" → 26a" β	0.838	LF
4A "	2.303	0.0005	36a' → 26a" β<br>22a" → 37a' β	0.738<br>0.217	LF<br>$6b_2$ σ → Ni d_{z2}
5A"	2.360	0.0004	22a" → 37a' β<br>36a' → 26a" β	0.759<br>0.185	$6b_2$ σ → Ni d_{z2}<br>LF
4A′	2.384	0.0035	34a' → 37a' β	0.905	$3b_1$ π → Ni d_{z2}
6A"	2.493	0.0025	21a" → 37a' β	0.998	LMCT (pz)
7A"	2.543	0.0000	26a" → 38a' α	0.970	Ni d → $4b_1$ π*
5A′	2.695	0.0010	33a' → 37a' β	0.983	LMCT (pz)
8A"	2.708	0.0003	20a" → 37a' β<br>35a' → 26a" β	0.584<br>0.380	LMCT (pz)<br>LF
9A"	2.759	0.0009	35a' → 26a" β<br>20a" → 37a' β	0.508<br>0.412	LF<br>LMCT (pz)
6A′	2.800	0.0161	24a" → 26a" β	0.863	$2a_2$ δ → Ni $d_σ$*
7A′	2.919	0.0109	32a' → 37a' β	0.974	LMCT (pz)
8A′	3.017	0.0001	23a" → 26a" β	0.998	LMCT (pz)
10A"	3.050	0.0001	19a" → 37a' β	0.945	LMCT (pz)
9A′	3.180	0.0023	26a" → 27a" α	0.966	MLCT (pz)
11A"	3.201	0.0001	26a" → 39a' α<br>34a' → 26a" β	0.783<br>0.211	MLCT (pz)<br>$3b_1$ π → Ni $d_σ$*

Table S50. continued.

No.	E/eV	f	orbitals	fraction	assignment
12A"	3.237	0.0002	34a' → 26a" β	0.725	$3b_1$ π → Ni d_σ*
			26a" → 39a' α	0.210	MLCT (pz)
10A′	3.306	0.0004	31a' → 37a' β	0.845	$7a_1$ σ → Ni d_{z2}
			22a" → 26a" β	0.133	$6b_2$ σ → Ni d_σ*
11A′	3.334	0.0000	21a" → 26a" β	0.994	LMCT (pz)
13A"	3.343	0.0007	25a" → 38a' β	0.822	Ni d → $4b_1$ π*
			25a" → 38a' α	0.159	Ni d → $4b_1$ π*
12A′	3.476	0.0003	36a' → 38a' β	0.749	Ni d → $4b_1$ π*
			37a' → 38a' α	0.199	Ni d → $4b_1$ π*
14A"	3.532	0.0005	33a' → 26a" β	0.981	LMCT (pz)
13A′	3.552	0.0327	20a" → 26a" β	0.686	LMCT (pz)
			22a" → 26a" β	0.189	$6b_2$ σ → Ni d_σ*
15A"	3.668	0.0002	25a" → 38a' α	0.434	Ni d → $4b_1$ π*
			24a" → 38a' β	0.299	intraligand
			32a' → 26a" β	0.144	LMCT (pz)
14A′	3.699	0.0839	22a" → 26a" β	0.406	$6b_2$ σ → Ni d_σ*
			20a" → 26a" β	0.278	LMCT (pz)
			37a' → 38a' α	0.104	Ni d → $4b_1$ π*
16A"	3.733	0.0020	32a' → 26a" β	0.833	LMCT (pz)
			24a" → 38a' β	0.107	intraligand
15A′	3.820	0.0784	35a' → 38a' β	0.406	Ni d → $4b_1$ π*
			37a' → 38a' α	0.237	Ni d → $4b_1$ π*
			36a' → 38a' β	0.147	Ni d → $4b_1$ π*
16A′	3.881	0.0010	26a" → 28a" α	0.971	MLCT (pz)
17A′	3.935	0.0024	19a" → 26a" β	0.935	LMCT (pz)
18A′	3.999	0.0211	25a" → 27a" β	0.519	MLCT (pz)
			35a' → 38a' β	0.185	Ni d → $4b_1$ π*
			36a' → 38a' α	0.126	Ni d → $4b_1$ π*
19A′	4.018	0.0179	25a" → 27a" β	0.386	MLCT (pz)
			36a' → 38a' α	0.222	Ni d → $4b_1$ π*
			35a' → 38a' β	0.165	Ni d → $4b_1$ π*

Table S50. continued.

No.	E/eV	f	orbitals	fraction	assignment
17A''	4.022	0.0002	25a'' → 39a' β	0.420	MLCT (pz)
			24a'' → 38a' β	0.227	intraligand
			24a'' → 38a' α	0.144	intraligand
			25a'' → 38a' α	0.108	Ni d → $4b_1$ π*
18A''	4.037	0.0042	25a'' → 39a' β	0.560	MLCT (pz)
			24a'' → 38a' α	0.156	intraligand
			24a'' → 38a' β	0.113	intraligand
19A''	4.059	0.0002	26a'' → 40a' α	0.912	MLCT (pz)
20A′	4.067	0.0276	36a' → 38a' α	0.601	Ni d → $4b_1$ π*
			35a' → 38a' β	0.180	Ni d → $4b_1$ π*
			34a' → 38a' β	0.150	intraligand

Table S51. Calculated electronic excitations for the synthetic model *5.*

No.	E/eV	f	orbitals	fraction	assignment
1A"	1.237	0.0001	39a' → 23a" β	0.993	LF
1A'	1.349	0.0018	22a" → 23a" β	0.987	LF
2A"	1.859	0.0000	38a' → 23a" β	0.768	LF
			22a" → 40a' β	0.222	LF
2A'	1.872	0.0080	21a" → 23a" β	0.962	$2a_2$ δ → Ni d_{xy}
3A'	2.084	0.0002	20a" → 23a" β	1.000	LMCT (pz)
3A"	2.198	0.0000	22a" → 40a' β	0.717	LF
			38a' → 23a" β	0.190	LF
4A"	2.259	0.0001	37a' → 23a" β	0.925	$6b_2$ σ → Ni d_{xy}
4A'	2.271	0.0025	19a" → 23a" β	0.713	$3b_1$ π → Ni d_{xy}
			39a' → 40a' β	0.265	LF
5A'	2.392	0.0276	39a' → 40a' β	0.539	LF
			18a" → 23a" β	0.191	LMCT (pz)
			19a" → 23a" β	0.149	$3b_1$ π → Ni d_{xy}
6A'	2.426	0.0131	18a" → 23a" β	0.797	LMCT (pz)
5A"	2.587	0.0001	40a' → 24a" α	0.953	Ni d → $4b_1$ π*
7A'	2.604	0.0002	17a" → 23a" β	0.880	LMCT (pz)
6A"	2.626	0.0000	36a' → 23a" β	0.898	LMCT (pz)
8A'	2.679	0.0051	38a' → 40a' β	0.793	LF
			17a" → 23a" β	0.111	LMCT (pz)
7A"	2.683	0.0000	21a" → 40a' β	0.908	$2a_2$ δ → Ni d_{z2}
8A"	2.867	0.0006	35a' → 23a" β	0.963	LMCT (pz)
9A'	2.929	0.0038	16a" → 23a" β	0.963	LMCT (pz)
9A"	2.941	0.0000	20a" → 40a' β	0.999	LMCT (pz)
10A"	3.062	0.0000	19a" → 40a' β	0.987	$3b_1$ π → Ni d_{z2}
10A'	3.239	0.0012	40a' → 41a' α	0.985	MLCT (pz)

Table S51. continued.

No.	E/eV	f	orbitals	fraction	assignment
11A''	3.255	0.0000	40a' → 25a'' α	0.618	MLCT (pz)
			18a'' → 40a' β	0.376	LMCT (pz)
12A''	3.273	0.0000	18a'' → 40a' β	0.620	LMCT (pz)
			40a' → 25a'' α	0.364	MLCT (pz)
13A''	3.322	0.0006	34a' → 23a'' β	0.970	$7a_1$ σ → Ni d_{xy}
11A'	3.351	0.0007	22a'' → 24a'' β	0.776	Ni d → $4b_1$ π^*
			23a'' → 24a'' α	0.153	Ni d → $4b_1$ π^*
14A''	3.419	0.0004	39a' → 24a'' β	0.984	Ni d → $4b_1$ π^*
12A'	3.462	0.0163	36a' → 40a' β	0.823	LMCT (pz)
			37a' → 40a' β	0.148	$6b_2$ σ → Ni d_{z2}
15A''	3.463	0.0000	17a'' → 40a' β	0.991	LMCT (pz)
13A'	3.614	0.0766	37a' → 40a' β	0.403	$6b_2$ σ → Ni d_{z2}
			22a'' → 24a'' α	0.150	Ni d → $4b_1$ π^*
			23a'' → 24a'' α	0.126	Ni d → $4b_1$ π^*
			36a' → 40a' β	0.119	LMCT (pz)
14A'	3.701	0.0445	21a'' → 24a'' β	0.319	intraligand
			23a'' → 24a'' α	0.317	Ni d → $4b_1$ π^*
			37a' → 40a' β	0.201	$6b_2$ σ → Ni d_{z2}
15A'	3.724	0.0036	35a' → 40a' β	0.871	LMCT (pz)
16A''	3.742	0.0000	16a'' → 40a' β	0.992	LMCT (pz)
16A'	3.841	0.1083	22a'' → 24a'' α	0.441	Ni d → $4b_1$ π^*
			19a'' → 24a'' β	0.226	intraligand
			22a'' → 24a'' β	0.108	Ni d → $4b_1$ π^*
17A''	3.942	0.0000	40a' → 26a'' α	0.992	MLCT (pz)
18A''	3.969	0.0000	38a' → 24a'' β	0.971	Ni d → $4b_1$ π^*
19A''	3.986	0.0004	39a' → 24a'' α	0.978	Ni d → $4b_1$ π^*
20A''	4.023	0.0010	22a'' → 41a' β	0.982	MLCT (pz)
17A'	4.038	0.0028	39a' → 41a' β	0.766	MLCT (pz)
			22a'' → 25a'' β	0.158	MLCT (pz)

Table S51. continued.

No.	E/eV	f	orbitals	fraction	assignment
18A'	4.070	0.0010	22a'' → 25a'' β	0.759	MLCT (pz)
			39a' → 41a' β	0.112	MLCT (pz)
21A''	4.074	0.0029	39a' → 25a'' β	0.975	MLCT (pz)
19A'	4.078	0.0028	20a'' → 24a'' α	0.391	intraligand
			21a'' → 24a'' β	0.227	intraligand
			23a'' → 24a'' α	0.137	Ni d → $4b_1$ π*

Table S52. Calculated electronic excitations for the synthetic model *6.*

**No.**	**E/eV**	**f**	**orbitals**	**fraction**	**assignment**
1A"	1.023	0.0006	25a" → 37a' β	0.994	$2a_2$ δ → Ni d_{z2}
2A"	1.154	0.0003	24a" → 37a' β	0.999	LMCT (pz)
1A'	1.243	0.0118	36a' → 37a' β	0.986	$3b_1$ π → Ni d_{z2}
3A"	1.352	0.0001	23a" → 37a' β	0.995	LMCT (pz)
2A'	1.469	0.0001	25a" → 26a" α	0.759	$2a_2$ δ → Ni d_{σ}*
			25a" → 26a" β	0.234	$2a_2$ δ → Ni d_{σ}*
4A"	1.572	0.0017	22a" → 37a' β	0.894	LMCT (pz)
			37a' → 26a" α	0.100	LF
3A'	1.575	0.0001	24a" → 26a" α	0.996	LMCT (pz)
5A"	1.626	0.0007	37a' → 26a" α	0.785	LF
6A"	1.702	0.0004	36a' → 26a" α	0.705	LMCT (pz)
			36a' → 26a" β	0.145	$3b_1$ π → Ni d_{σ}*
4A'	1.704	0.0011	35a' → 37a' β	0.927	LMCT (pz)
7A"	1.743	0.0000	21a" → 37a' β	0.942	LMCT (pz)
5A'	1.793	0.0005	23a" → 26a" α	0.851	LMCT (pz)
6A'	1.896	0.0019	25a" → 26a" β	0.400	$2a_2$ δ → Ni d_{σ}*
			24a" → 26a" β	0.351	LMCT (pz)
			25a" → 26a" α	0.118	$2a_2$ δ → Ni d_{σ}*
7A'	1.913	0.0008	24a" → 26a" β	0.637	LMCT (pz)
			25a" → 26a" β	0.210	$2a_2$ δ → Ni d_{σ}*
8A'	1.970	0.0002	34a' → 37a' β	0.803	LMCT (pz)
8A"	1.991	0.0002	36a' → 26a" β	0.745	$3b_1$ π → Ni d_{σ}*
			36a' → 26a" α	0.181	LMCT (pz)
9A'	1.993	0.0003	22a" → 26a" α	0.844	LMCT (pz)
10A'	2.106	0.0002	23a" → 26a" β	0.675	LMCT (pz)
			21a" → 26a" α	0.195	LMCT (pz)
9A"	2.168	0.0000	35a' → 26a" α	0.883	LMCT (pz)

Table S52. continued.

**No.**	**E/eV**	**f**	**orbitals**	**fraction**	**assignment**
11A'	2.197	0.0039	21a" → 26a" α	0.729	LMCT (pz)
			23a" → 26a" β	0.189	LMCT (pz)
12A'	2.327	0.0001	22a" → 26a" β	0.968	LMCT (pz)
10A"	2.367	0.0009	34a' → 26a" α	0.339	$3b_1$ π → Ni $d_σ$*
			35a' → 26a" β	0.317	LMCT (pz)
			20a" → 37a' β	0.249	$6b_2$ σ → Ni d_{z2}
11A"	2.395	0.0023	20a" → 37a' β	0.595	$6b_2$ σ → Ni d_{z2}
			35a' → 26a" β	0.354	LMCT (pz)
13A'	2.404	0.0049	33a' → 37a' β	0.866	LF
14A'	2.492	0.0001	21a" → 26a" β	0.835	LMCT (pz)
12A"	2.537	0.0001	34a' → 26a" β	0.352	LMCT (pz)
			34a' → 26a" α	0.303	$3b_1$ π → Ni $d_σ$*
			35a' → 26a" β	0.251	LMCT (pz)
15A'	2.679	0.0065	20a" → 26a" α	0.435	$6b_2$ σ → Ni $d_σ$*
			20a" → 26a" β	0.403	$6b_2$ σ → Ni $d_σ$*
13A"	2.713	0.0001	34a' → 26a" β	0.583	LMCT (pz)
			34a' → 26a" α	0.316	$3b_1$ π → Ni $d_σ$*
14A"	2.777	0.0001	19a" → 37a' β	0.967	LF
16A'	2.827	0.0029	32a' → 37a' β	0.951	LF
15A"	2.891	0.0006	33a' → 26a" α	0.484	LF
			33a' → 26a" β	0.464	LF
16A"	3.228	0.0000	32a' → 26a" β	0.555	LF
			32a' → 26a" α	0.394	LF
17A'	3.297	0.0000	19a" → 26a" α	0.562	LF
			19a" → 26a" β	0.418	LF
18A'	3.436	0.0043	31a' → 37a' β	0.842	$7a_1$ σ → Ni d_{z2}
17A"	3.471	0.0094	33a' → 26a" β	0.400	LF
			33a' → 26a" α	0.322	LF
			32a' → 26a" α	0.166	LF

Table S52. continued.

No.	E/eV	f	orbitals	fraction	assignment
19A'	3.602	0.0003	30a' → 37a' β	0.991	LMCT
18A"	3.639	0.0004	32a' → 26a" α	0.316	LF
			32a' → 26a" β	0.308	LF
			25a" → 38a' β	0.191	intraligand
19A"	3.644	0.0004	25a" → 38a' β	0.333	intraligand
			25a" → 38a' α	0.326	intraligand
			32a' → 26a" β	0.113	LF
			32a' → 26a" α	0.102	LF
20A'	3.722	0.0010	37a' → 38a' α	0.463	Ni d → $4b_1$ π*
			36a' → 38a' β	0.436	intraligand
21A'	3.811	0.0517	19a" → 26a" β	0.345	LF
			19a" → 26a" α	0.263	LF
			20a" → 26a" β	0.136	$6b_2$ σ → Ni d_{σ}*
			31a' → 37a' β	0.105	$7a_1$ σ → Ni d_{z2}
20A"	3.841	0.0000	24a" → 38a' α	0.955	interligand
21A"	3.843	0.0007	31a' → 26a" α	0.610	$7a_1$ σ → Ni d_{σ}*
			31a' → 26a" β	0.285	$7a_1$ σ → Ni d_{σ}*
22A"	3.872	0.0000	24a" → 38a' β	0.978	interligand
23A"	3.928	0.0007	25a" → 38a' α	0.309	intraligand
			25a" → 38a' β	0.302	intraligand
			23a" → 38a' β	0.285	interligand
22A'	3.969	0.0009	36a' → 38a' α	0.628	interligand
			37a' → 38a' α	0.257	Ni d → $4b_1$ π*
24A"	4.019	0.0000	30a' → 26a" α	0.957	LMCT
25A"	4.038	0.0000	23a" → 38a' α	0.651	interligand
			23a" → 38a' β	0.258	interligand
23A'	4.092	0.0493	36a' → 38a' β	0.217	intraligand
			36a' → 38a' α	0.183	interligand
			35a' → 38a' β	0.103	interligand
			20a" → 26a" β	0.102	$6b_2$ σ → Ni d_{σ}*

Table S52. continued.

No.	E/eV	f	orbitals	fraction	assignment
26A"	4.187	0.0000	23a" → 38a' β	0.285	interligand
			23a" → 38a' α	0.215	interligand
			22a" → 38a' α	0.154	interligand
			31a' → 26a" β	0.101	$7a_1$ σ → Ni d_σ*
24A′	4.187	0.3318	36a' → 38a' β	0.158	intraligand
			35a' → 38a' β	0.143	interligand
			20a" → 26a" β	0.135	$6b_2$ σ → Ni d_σ*
			20a" → 26a" α	0.111	$6b_2$ σ → Ni d_σ*

Table S53. Calculated electronic excitations for the synthetic model *7.*

No.	E/eV	f	orbitals	fraction	assignment
1A"	1.032	0.0002	39a' → 23a" β	0.990	LMCT (pz)
1A'	1.095	0.0027	22a" → 23a" β	0.829	$3b_1$ π → Ni d_{xy}
			21a" → 23a" β	0.164	LMCT (pz)
2A'	1.114	0.0015	21a" → 23a" β	0.835	LMCT (pz)
			22a" → 23a" β	0.163	$3b_1$ π → Ni d_{xy}
3A'	1.412	0.0087	20a" → 23a" β	0.946	$2a_2$ δ → Ni d_{xy}
2A"	1.489	0.0001	21a" → 40a' α	0.667	$3b_1$ π → Ni d_{z2}
			22a" → 40a' β	0.241	$3b_1$ π → Ni d_{z2}
4A'	1.497	0.0004	39a' → 40a' α	0.530	LMCT (pz)
			19a" → 23a" β	0.260	LMCT (pz)
			39a' → 40a' β	0.188	LMCT (pz)
3A"	1.517	0.0000	22a" → 40a' α	0.942	LMCT (pz)
4A"	1.539	0.0015	23a" → 40a' α	0.799	LF
5A'	1.542	0.0034	19a" → 23a" β	0.698	LMCT (pz)
			39a' → 40a' α	0.141	LMCT (pz)
			39a' → 40a' β	0.114	LMCT (pz)
5A"	1.645	0.0004	38a' → 23a" β	0.899	LMCT (pz)
6A'	1.735	0.0041	18a" → 23a" β	0.947	LMCT (pz)
6A"	1.795	0.0000	20a" → 40a' α	0.771	$2a_2$ δ → Ni d_{z2}
7A"	1.828	0.0002	22a" → 40a' β	0.410	$3b_1$ π → Ni d_{z2}
			21a" → 40a' β	0.261	LMCT (pz)
			20a" → 40a' α	0.127	$2a_2$ δ → Ni d_{z2}
			21a" → 40a' α	0.108	$3b_1$ π → Ni d_{z2}
8A"	1.846	0.0000	21a" → 40a' β	0.718	LMCT (pz)
			22a" → 40a' β	0.141	$3b_1$ π → Ni d_{z2}
7A'	1.901	0.0034	39a' → 40a' β	0.464	LMCT (pz)
			39a' → 40a' α	0.261	LMCT (pz)
			38a' → 40a' α	0.200	LMCT (pz)
9A"	1.931	0.0000	19a" → 40a' α	0.903	LMCT (pz)

Table S53. continued.

No.	E/eV	f	orbitals	fraction	assignment
10A''	1.976	0.0012	37a' → 23a'' β	0.814	LF
8A'	2.101	0.0017	38a' → 40a' α	0.682	LMCT (pz)
			39a' → 40a' β	0.128	LMCT (pz)
11A''	2.111	0.0000	20a'' → 40a' β	0.753	$2a_2$ δ → Ni d_{z2}
			18a'' → 40a' α	0.130	LMCT (pz)
12A''	2.154	0.0001	18a'' → 40a' α	0.834	LMCT (pz)
			20a'' → 40a' β	0.116	$2a_2$ δ → Ni d_{z2}
9A'	2.193	0.0184	17a'' → 23a'' β	0.818	LMCT (pz)
13A''	2.251	0.0002	19a'' → 40a' β	0.941	LMCT (pz)
10A'	2.317	0.0001	38a' → 40a' β	0.552	LMCT (pz)
			37a' → 40a' α	0.337	LF
14A''	2.437	0.0000	18a'' → 40a' β	0.976	LMCT (pz)
11A'	2.488	0.0044	37a' → 40a' β	0.479	LF
			38a' → 40a' β	0.305	LMCT (pz)
			37a' → 40a' α	0.166	LF
15A''	2.496	0.0008	36a' → 23a'' β	0.807	$6b_2$ σ → Ni d_{xy}
16A''	2.673	0.0010	17a'' → 40a' α	0.430	LMCT (pz)
			17a'' → 40a' β	0.274	LMCT (pz)
			35a' → 23a'' β	0.146	LF
			36a' → 23a'' β	0.123	$6b_2$ σ → Ni d_{xy}
12A'	2.717	0.0050	16a'' → 23a'' β	0.935	LF
17A''	2.725	0.0000	35a' → 23a'' β	0.640	LF
			17a'' → 40a' β	0.314	LMCT (pz)
13A'	2.757	0.0032	36a' → 40a' α	0.492	$6b_2$ σ → Ni d_{z2}
			36a' → 40a' β	0.398	$6b_2$ σ → Ni d_{z2}
18A''	2.891	0.0030	17a'' → 40a' α	0.447	LMCT (pz)
			17a'' → 40a' β	0.397	LMCT (pz)
			35a' → 23a'' β	0.120	LF
14A'	3.015	0.0241	37a' → 40a' β	0.358	LF
			37a' → 40a' α	0.353	LF

Table S53. continued.

No.	E/eV	f	orbitals	fraction	assignment
19A"	3.162	0.0001	16a" → 40a' α	0.505	LF
			16a" → 40a' β	0.482	LF
15A'	3.170	0.0040	35a' → 40a' β	0.432	LF
			35a' → 40a' α	0.430	LF
20A"	3.515	0.0027	34a' → 23a" β	0.728	$7a_1$ σ → Ni d_{xy}
			16a" → 40a' β	0.144	LF
			16a" → 40a' α	0.115	LF
16A'	3.556	0.0127	35a' → 40a' α	0.432	LF
			35a' → 40a' β	0.234	LF
			36a' → 40a' β	0.215	$6b_2$ σ → Ni d_{z2}
21A"	3.576	0.0003	33a' → 23a" β	0.759	LMCT
17A'	3.651	0.0002	22a" → 24a" β	0.560	intraligand
			21a" → 24a" α	0.353	intraligand
22A"	3.655	0.0023	16a" → 40a' α	0.279	LF
			16a" → 40a' β	0.272	LF
			33a' → 23a" β	0.231	LMCT
			34a' → 23a" β	0.176	$7a_1$ σ → Ni d_{xy}
18A'	3.781	0.0005	23a" → 24a" α	0.746	Ni d → $4b_1$ π*
			20a" → 24a" β	0.102	intraligand
19A'	3.809	0.0000	22a" → 24a" α	0.990	interligand
23A"	3.815	0.0003	39a' → 24a" β	0.978	interligand
20A'	3.864	0.0000	21a" → 24a" β	0.985	interligand
24A"	3.911	0.0004	39a' → 24a" α	0.985	interligand
21A'	3.914	0.0035	33a' → 40a' α	0.499	$7a_1$ σ → Ni d_{z2}
			34a' → 40a' β	0.173	$7a_1$ σ → Ni d_{z2}
			34a' → 40a' α	0.144	LMCT
22A'	3.967	0.0003	34a' → 40a' α	0.844	LMCT
			33a' → 40a' α	0.120	$7a_1$ σ → Ni d_{z2}
23A'	4.044	0.0000	20a" → 24a" α	0.552	intraligand
			20a" → 24a" β	0.315	intraligand

Table S53. continued.

No.	E/eV	f	orbitals	fraction	assignment
24A'	4.069	0.0081	21a" → 24a" α	0.224	intraligand
			22a" → 24a" β	0.188	intraligand
			20a" → 24a" β	0.102	intraligand
			20a" → 24a" α	0.101	intraligand

Table S54. Calculated electronic excitations for the synthetic model *8*.

No.	E/eV	f	orbitals	fraction	assignment
1A'	1.990	0.0004	22a" → 23a"	0.986	LF
1A"	2.114	0.0001	27a' → 23a"	0.856	LF
			28a' → 23a"	0.124	LF
2A"	2.219	0.0004	28a' → 23a"	0.865	LF
			27a' → 23a"	0.124	LF
3A"	2.780	0.0000	22a" → 29a'	0.941	Ni d → $4b_1$ π*
2A'	2.783	0.0019	28a' → 29a'	0.970	Ni d → $4b_1$ π*
4A"	2.838	0.0003	26a' → 23a"	0.561	LF
			25a' → 23a"	0.403	LMCT (pz)
3A'	2.955	0.0003	21a" → 23a"	0.904	$2a_2$ δ → Ni d_σ*
5A"	2.974	0.0009	25a' → 23a"	0.592	LMCT (pz)
			26a' → 23a"	0.380	LF
4A'	3.037	0.0513	27a' → 29a'	0.920	Ni d → $4b_1$ π*
5A'	3.129	0.0011	20a" → 23a"	0.919	LMCT (pz)
6A"	3.468	0.0000	24a' → 23a"	0.952	$3b_1$ π → Ni d_σ*
6A'	3.626	0.0009	28a' → 30a'	0.681	MLCT (pz)
			18a" → 23a"	0.170	LMCT (pz)
			19a" → 23a"	0.138	$6b_1$ σ → Ni d_σ*
7A"	3.633	0.0124	22a" → 30a'	0.943	MLCT (pz)
7A'	3.681	0.0045	18a" → 23a"	0.364	LMCT (pz)
			28a' → 30a'	0.314	MLCT (pz)
			19a" → 23a"	0.286	$6b_1$ σ → Ni d_σ*
8A"	3.770	0.0000	23a' → 23a"	0.962	LMCT (pz)
8A'	3.824	0.0127	27a' → 30a'	0.786	MLCT (pz)
			22a" → 24a"	0.144	MLCT (pz)
9A'	3.899	0.0001	26a' → 29a'	0.929	Ni d → $4b_1$ π*
9A"	4.010	0.0001	28a' → 24a"	0.991	MLCT (pz)
10A'	4.047	0.0018	25a' → 29a'	0.940	interligand

Table S54. (continued).

No.	E/eV	f	orbitals	fraction	assignment
11A'	4.110	0.0797	22a" → 24a"	0.729	MLCT (pz)
			27a' → 30a'	0.133	MLCT (pz)
10A"	4.134	0.0002 A"	27a' → 24a"	0.476	MLCT (pz)
			21a" → 29a'	0.398	Ni d → $4b_1$ π*
11A"	4.184	0.0192 A"	27a' → 24a"	0.467	MLCT (pz)
			21a" → 29a'	0.246	Ni d → $4b_1$ π*
			20a" → 29a'	0.236	interligand
12A"	4.316	0.0132 A"	20a" → 29a'	0.636	interligand
			21a" → 29a'	0.197	intraligand
13A"	4.398	0.0000 A"	22a" → 31a'	0.989	MLCT (pz)
12A'	4.445	0.0767 A'	28a' → 31a'	0.820	MLCT (pz)
14A"	4.515	0.0002 A"	21a' → 23a"	0.795	$2a_2$ δ → Ni $d_σ$*
			22a' → 23a"	0.204	$7a_1$ σ → Ni $d_σ$*
13A'	4.525	0.2709	19a" → 23a"	0.315	$6b_2$ σ→ Ni $d_σ$*
			18a" → 23a"	0.279	LMCT (pz)
			28a' → 31a'	0.145	MLCT (pz)

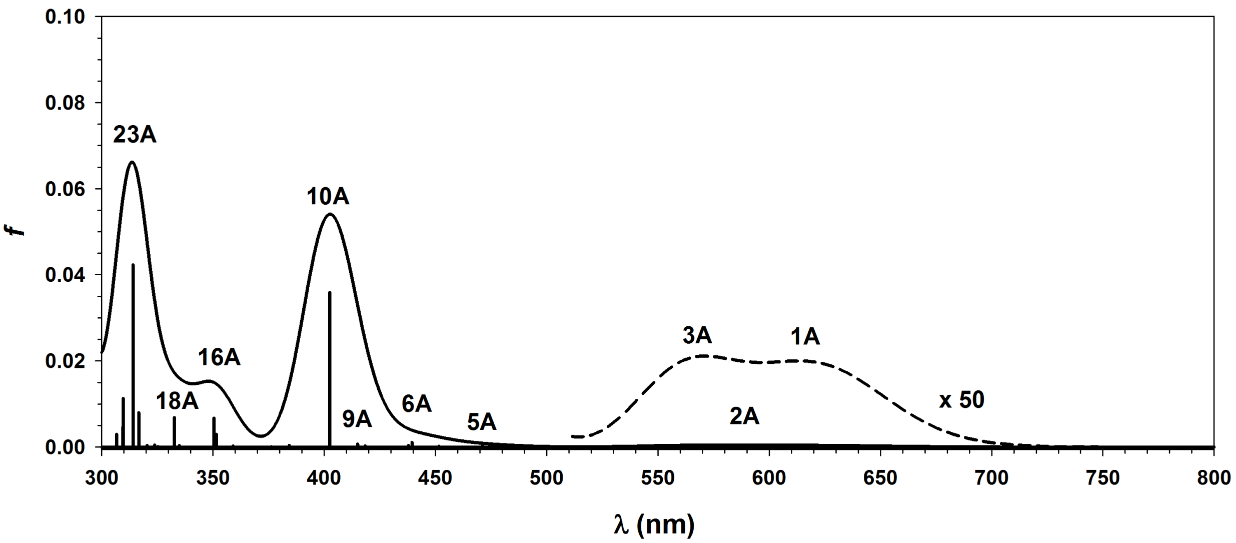

Figure S29. Calculated electronic spectrum of the low-spin (*S* = 0), square-planar Ni(II) synthetic model *1*, rendered with arbitrary peak widths of 1700 cm^{-1}. Calculated transitions are listed in Table S48.

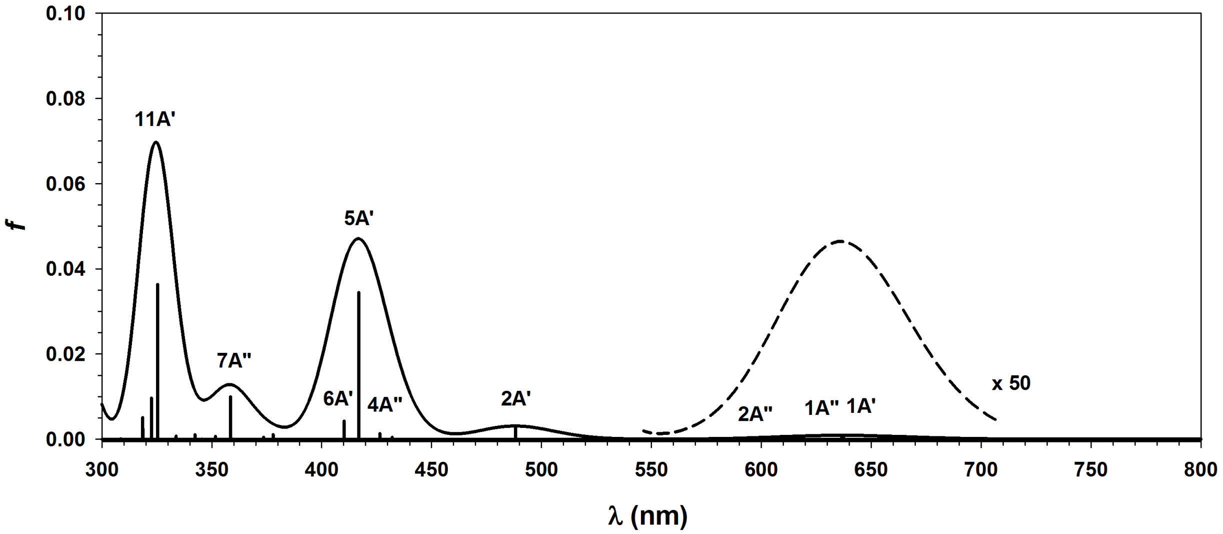

Figure S30. Calculated electronic spectrum of the low-spin (*S* = 0), square-pyramidal Ni(II) synthetic model *2*, rendered with arbitrary peak widths of 1700 cm^{-1}. All calculated transitions are listed in Table S49.

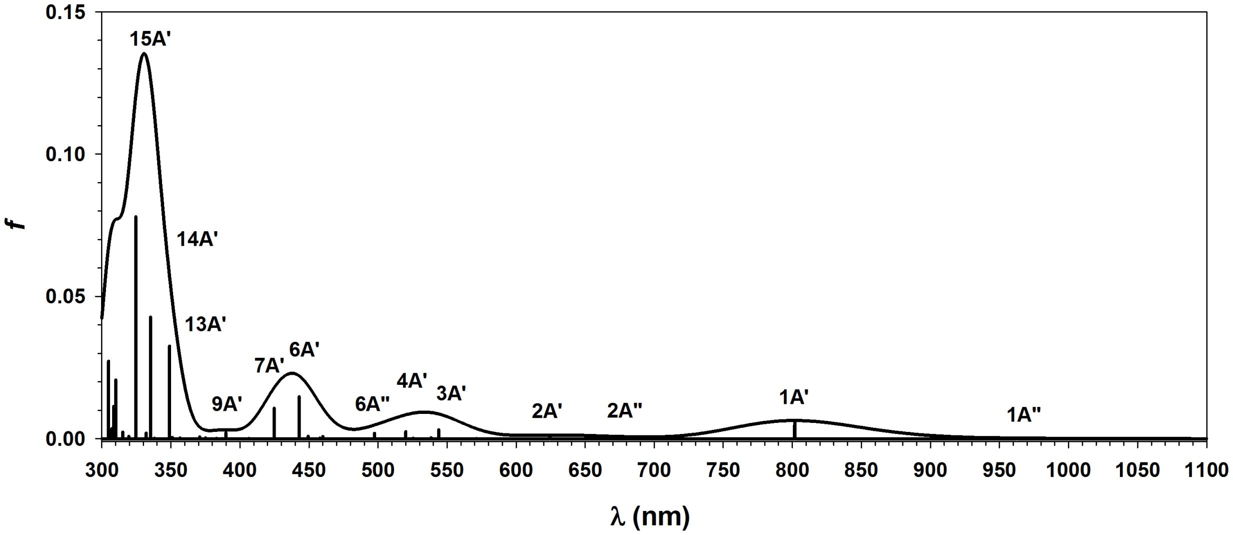

Figure S31. Calculated electronic spectrum of the high-spin (S = 1), square-pyramidal Ni(II) synthetic model *3*, rendered with arbitrary peak widths of 1700 cm^{-1}. All calculated transitions are listed in Table S50.

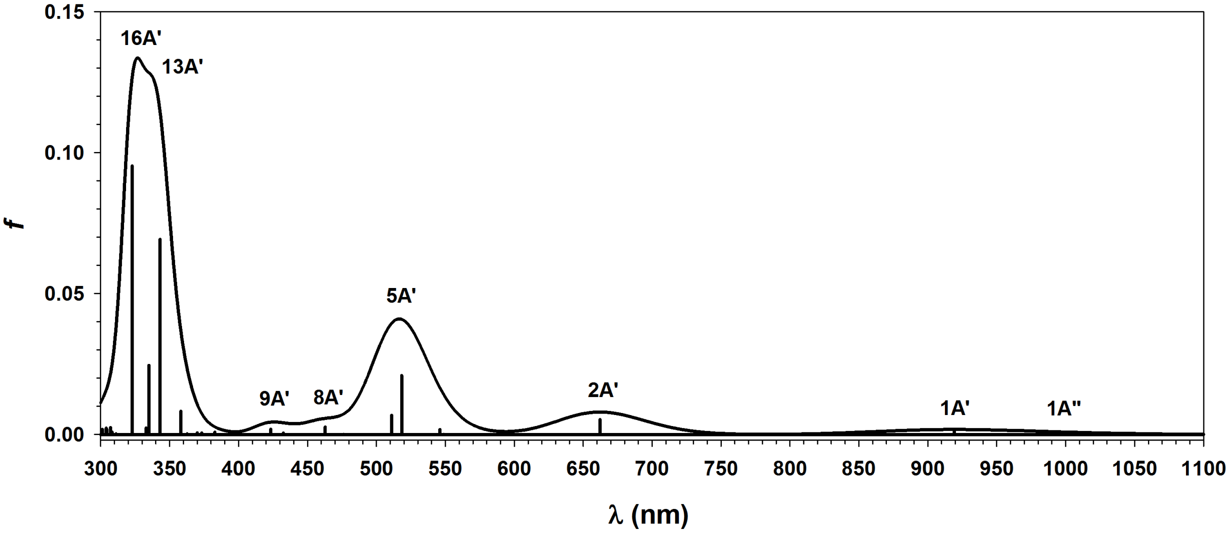

Figure S32. Calculated electronic spectrum of the high-spin (S = 1), trigonal-bipyramidal Ni(II) synthetic model *5*, rendered with arbitrary peak widths of 1700 cm^{-1}. All calculated transitions are listed in Table S51.

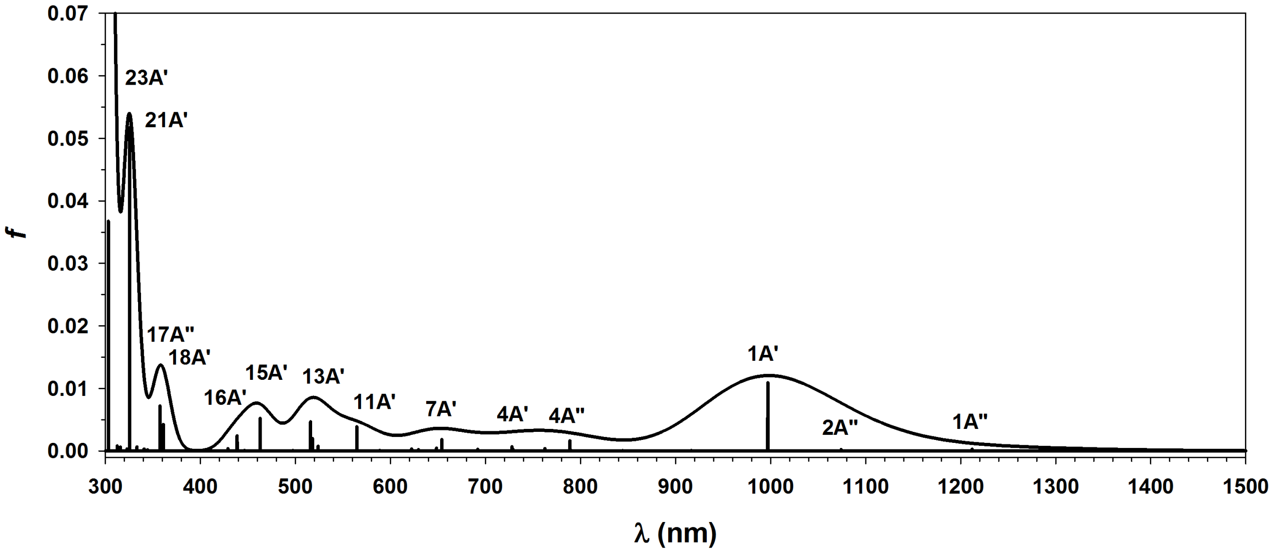

Figure S33. Calculated electronic spectrum of the square-pyramidal Ni(III) synthetic model *6*, rendered with arbitrary peak widths of 1700 cm^{-1}. All calculated transitions are listed in Table S52.

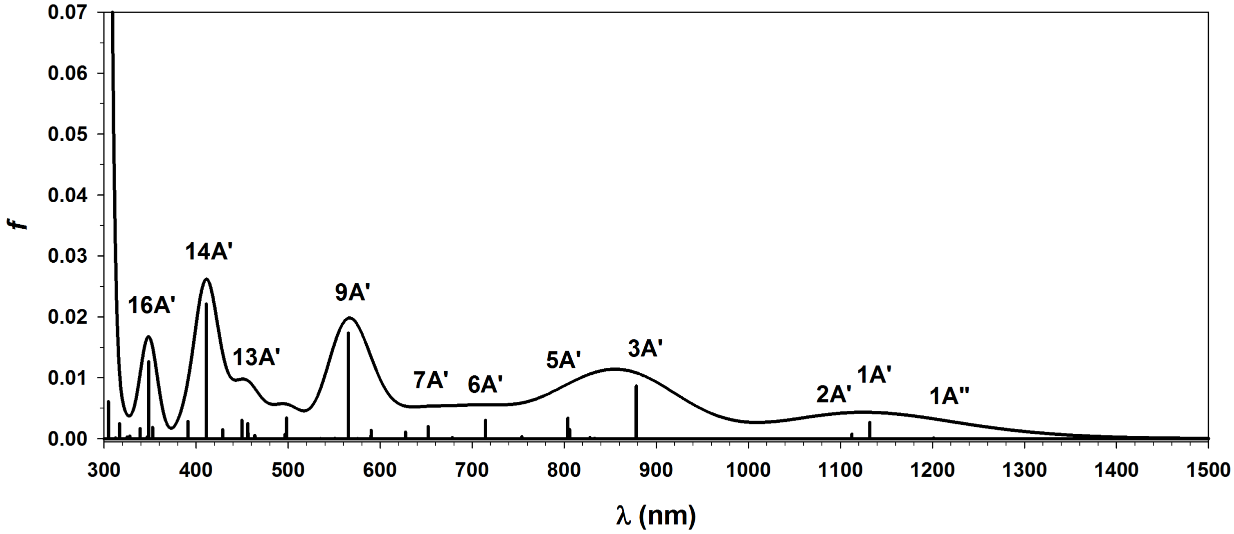

Figure S34. Calculated electronic spectrum of the trigonal-bipyramidal Ni(III) synthetic model *7*, rendered with arbitrary peak widths of 1700 cm^{-1}. All calculated transitions are listed in Table S53.

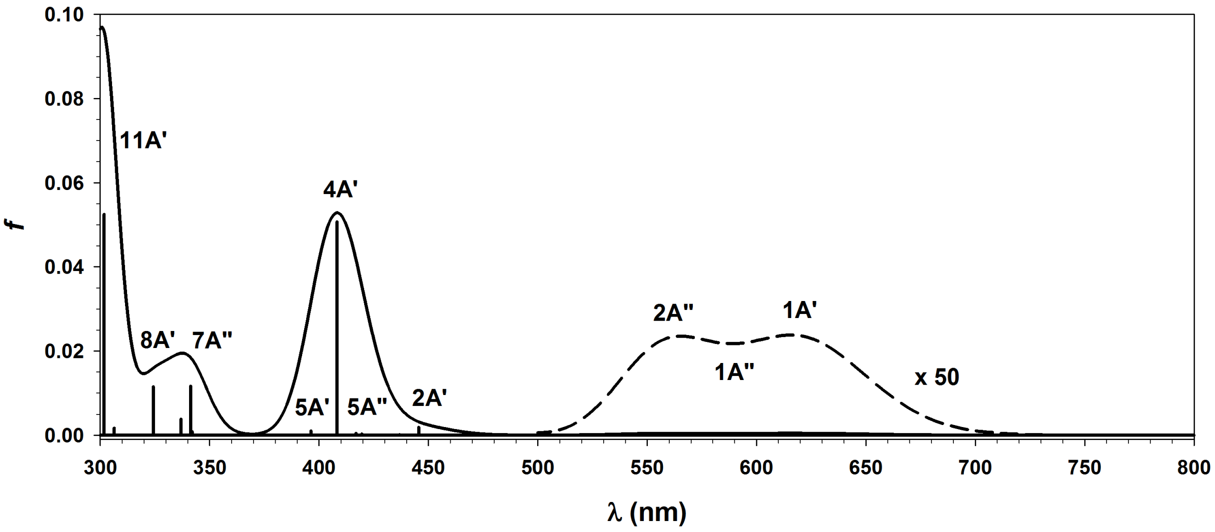

Figure S35. Calculated electronic spectrum of the square-planar Ni(II) synthetic model *8*, rendered with arbitrary peak widths of 1700 cm^{-1}. All calculated transitions are listed in Table S54.

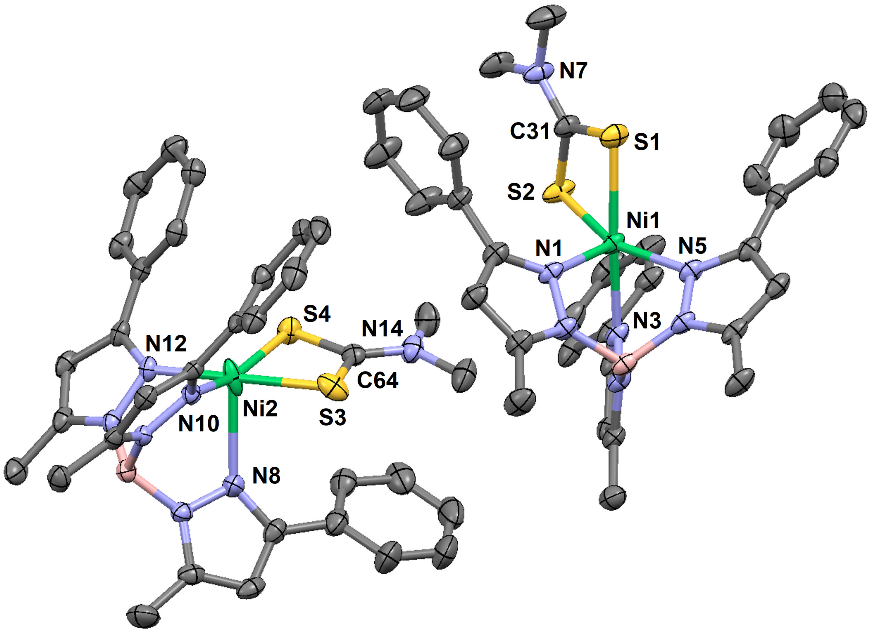

Figure S36. Thermal ellipsoid plot for the two independent molecules of [($Tp^{Ph,Me}$)NiS_2CNMe_2] (**1**) at 123 K (50% probability). Hydrogen atoms are omitted for clarity. Select bond lengths (Å) for **1**: Ni1–N1, 2.038(2); Ni1–N3, 2.111(2); Ni1–N5, 2.048(2); Ni1–S1, 2.4006(6); Ni1–S2, 2.3420(6); C31–S1, 1.712(2); C31–S2, 1.723(2); C31–N7, 1.327(3); Ni2–N8, 2.401(2); Ni2–N10, 1.972(2); Ni2–N12, 2.003(2); Ni2–S3, 2.2721(7); Ni2–S4, 2.2567(6); C64–S3, 1.705(2); C64–S4, 1.720(2); C64–N14, 1.320(3). Select bond angles (°) for **1**: N1–Ni1–N3, 90.77(7); N1–Ni1–N5, 95.18(7); N3–Ni1–N5, 84.13(7); N1–Ni1–S1, 96.05(5); N1–Ni1–S2, 108.94(5); N3–Ni1–S1, 172.88(5); N3–Ni1–S2, 100.43(5); N5–Ni1–S1, 97.25(5); N5–Ni1–S2, 155.29(5); S1–Ni1–S2,

75.43(2); S1–C31–S2, 115.3(1); N7–C31–S1, 122.5(2); N7–C31–S2, 122.2(2); N8–Ni2–N10, 93.18(7); N8–Ni2–N12, 88.77(6); N10–Ni2–N12, 88.45(6); N8–Ni2–S3, 89.71(5); N8–Ni2–S4, 103.82(5); N10–Ni2–S3, 96.54(5); N10–Ni2–S4, 161.87(5); N12–Ni2–S3, 174.86(5); N12–Ni2–S4, 98.05(5); S3–Ni2–S4, 77.54(2); S3–C64–S4, 111.8(1); N14–C64–S3, 124.2(2); N14–C64–S4, 124.1(2). Select torsions (°) for **1**: H–B1–N2–N1, 179.9; H–B2–N9–N8, 163.5.

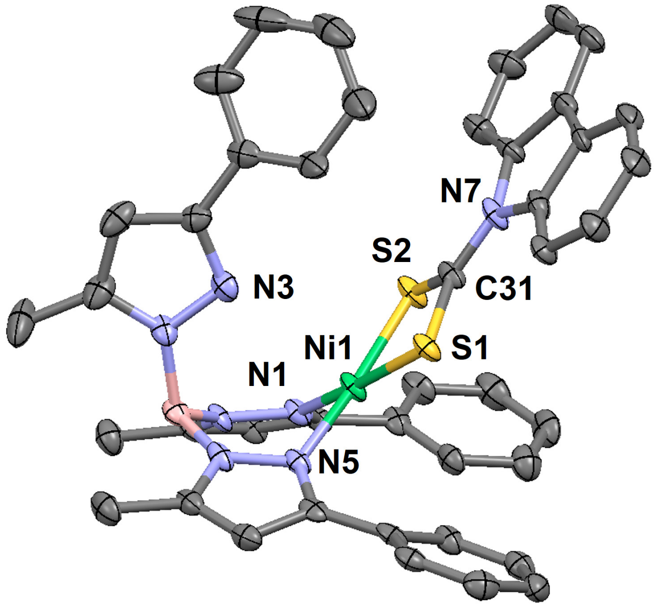

Figure S37. Thermal ellipsoid plots (30% probability) for red crystals of [(Tp^{Ph,Me})NiS$_2$CNC$_{12}$H$_8$] (**4**) at 173 K. Hydrogen atoms are omitted for clarity. Select bond lengths (Å) for **4**: Ni1–N1, 1.913(5); Ni1•••N3, 2.942(5); Ni1–N5, 1.922(5); Ni1–S1, 2.192(2); Ni1–S2, 2.176(2); C31–N7, 1.359(7); C31–S1, 1.696(6); C31–S2, 1.702(6). Select bond angles (°) for **4**: N1–Ni1–N5, 90.2(2); N1–Ni1–S1, 171.8(2); N1–Ni1–S2, 94.3(2); N5–Ni1–S1, 98.0(2); N5–Ni1–S2, 173.0(2); N3•••Ni1–N1, 85.1(2); N3•••Ni1–N5, 85.4(2); N3•••Ni1–S1, 96.6(1); N3•••Ni1–S2, 89.5(1); S1–Ni1–S2, 77.68(6); S1–C31–S2, 107.5(3); S1–C31–N7, 127.2(5); S2–C31–N7, 125.3(5). Select torsion (°) for **4**: H–B1–N4–N3, 174.4.

Summary of the structure and refinement: $C_{43}H_{36}BN_7NiS_2 \cdot CH_2Cl_2$; 869.35 g/mol; red block; 173(2) K; monoclinic, P2$_1$/n (No. 14); a, 13.222(2) Å; b, 14.652(3) Å; c, 24.713(4) Å; α, 90°; β, 102.266(3)°; γ, 90°; V, 4678(1) Å^3; Z = 4; D_{calcd}, 1.23 g/cm^3; absorption coefficient, 0.655 mm^{-1}; crystal size 0.25 × 0.25 × 0.10 mm; 38260 reflections collected; 8461 independent reflections (R_{int}, 0.0929); 5062 reflections observed; 8461/0/517 data/restraints/parameters; GoF, 1.051; $R1$, 0.0879, $I > 2\sigma(I)$; $wR2$, 0.2049, $I > 2\sigma(I)$; $R1$, 0.1458, all data; $wR2$, 0.2281, all data; difference peak/hole, 1.266/−0.510 e Å^{-3}.

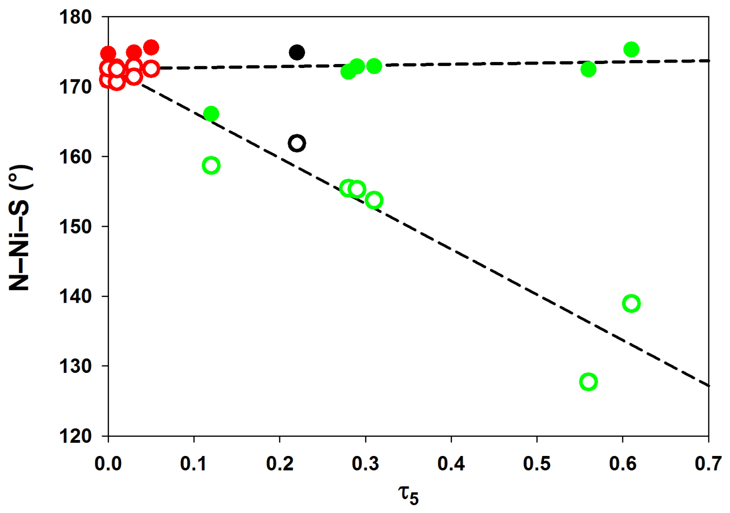

Figure S38. Plot of N−Ni−S *trans* angles vs. the derived τ^5 parameter for complexes **1**−**5** and **7** (Table 3). Data for diamagnetic complexes are shown in red, and paramagnetic complexes in green; those rendered in black are for the Ni2 site of complex **1** at 123 K, which exhibits partial spin crossover. Dashed lines are linear least-squares regressions: $y = 172.5(9) + 2(3)x$, $r^2 = 0.02$ (●); $y = 173(1) - 65(5)x$, $r^2 = 0.93$ (○).

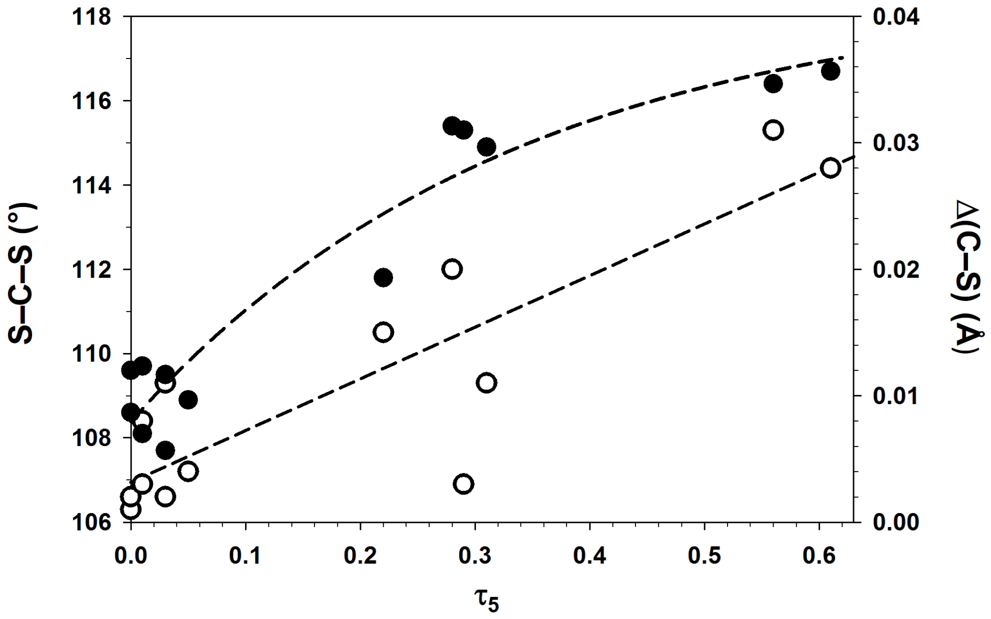

Figure S39. Plots of τ^5 values vs. S–C–S bond angle (●) and the difference in C–S bond lengths (○) within the dithiocarbamate ligands.

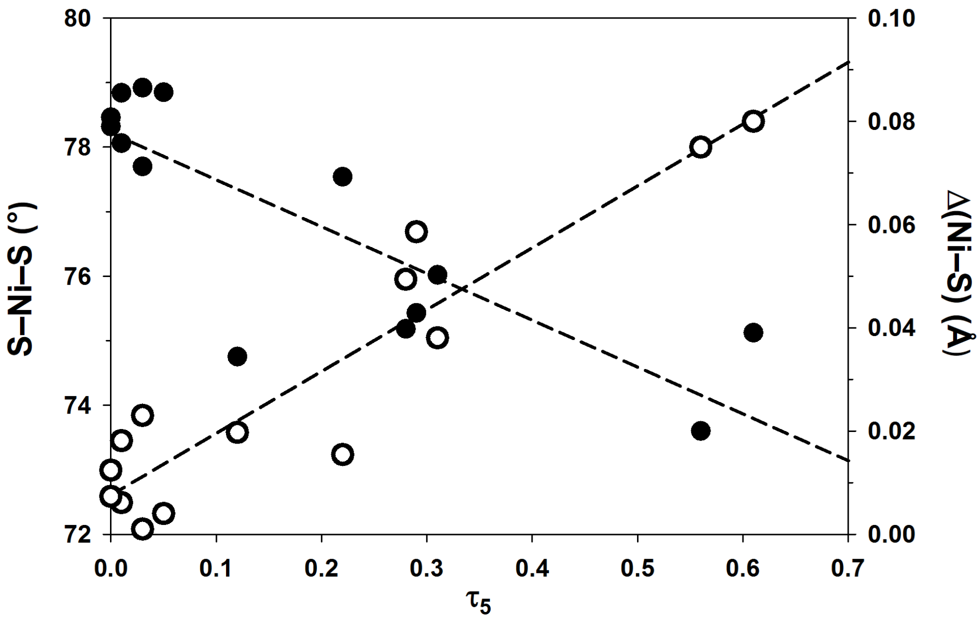

Figure S40. Plots of τ^5 values vs. S–Ni–S bond angle (●) and the difference in Ni–S bond lengths (○).

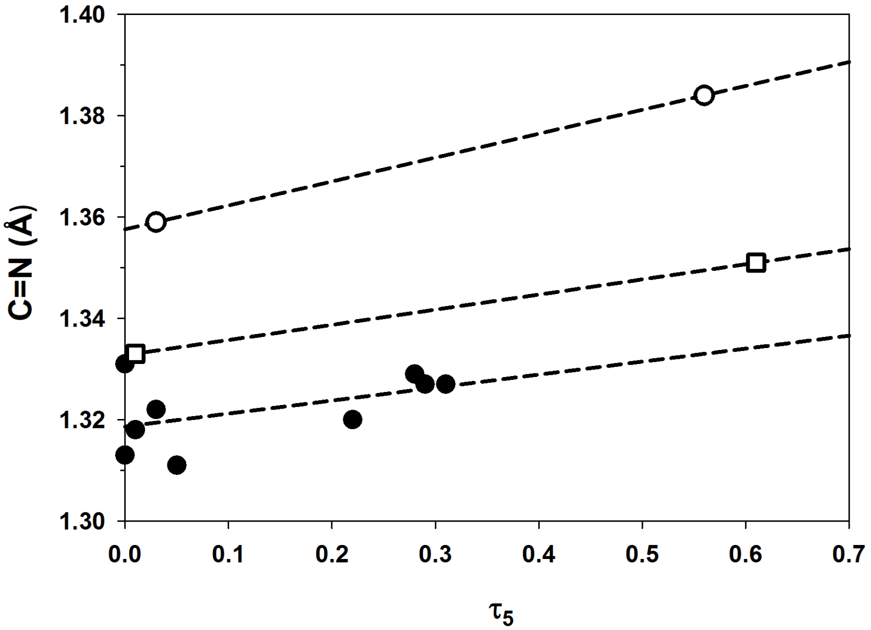

Figure S41. Plots of τ^5 values vs. $S_2C=NR_2$ bond length for R = alkyl (●) and phenyl (□), and for the carbazole-derived ligand (○).

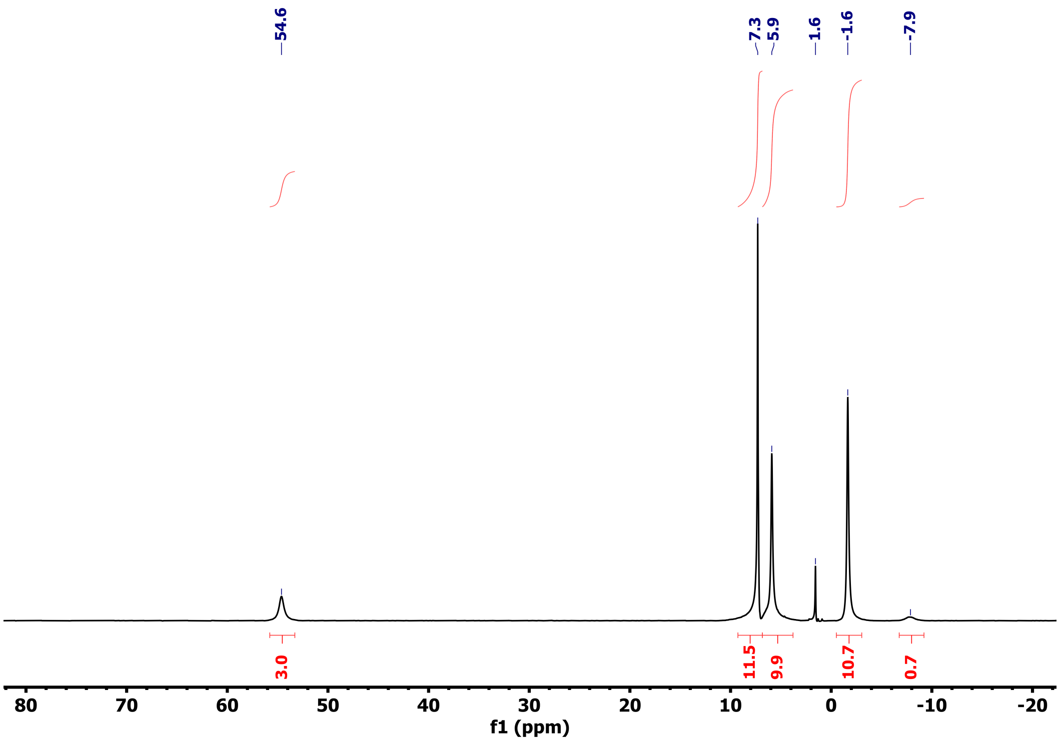

Figure S42. ^{1}H NMR spectrum ($CDCl_3$, 295 K) of [($Tp^{Ph,Me}$)$_2$Ni].

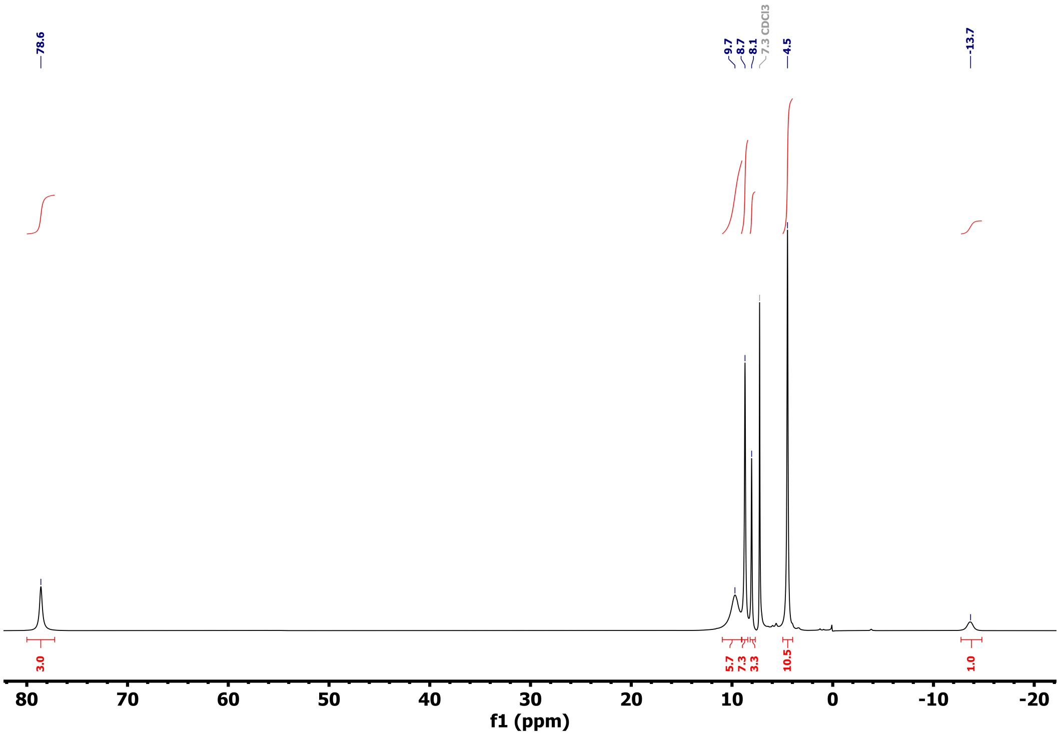

Figure S43. ^{1}H NMR spectrum ($CDCl_3$, 295 K) of [($Tp^{Ph,Me}$)Ni–Cl].

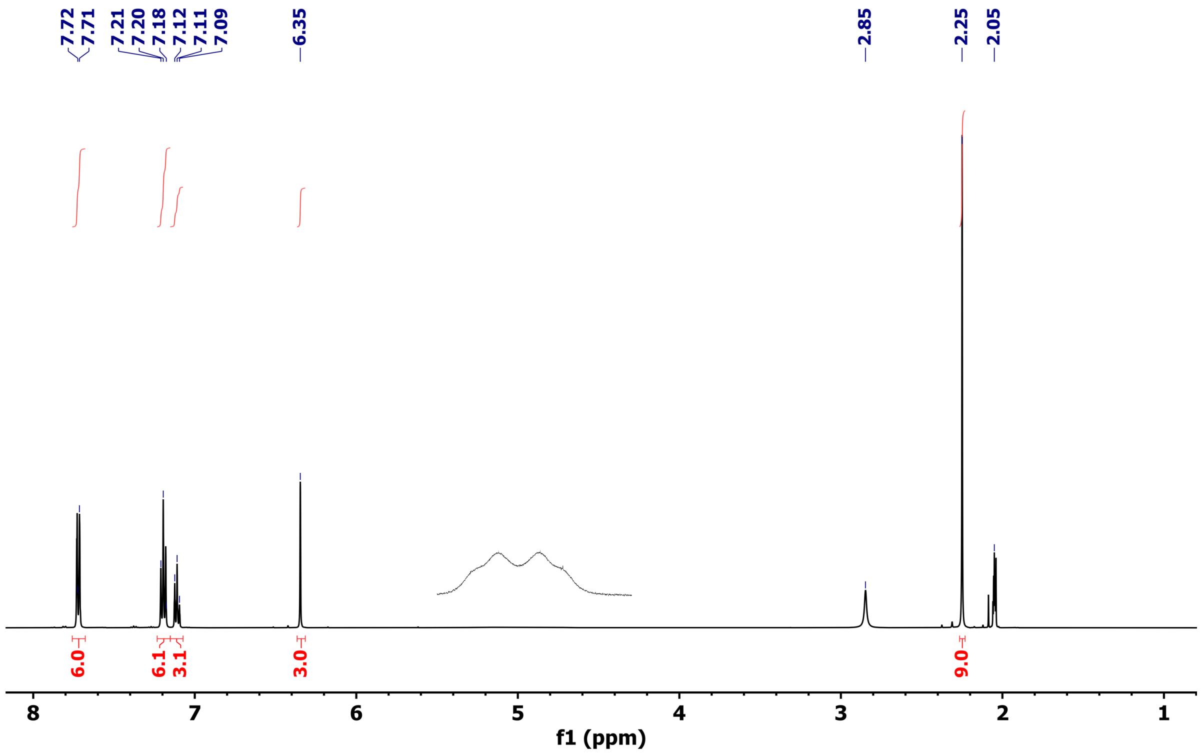

Figure S44. ^{1}H NMR spectrum (d_6-acetone, 295 K) of K(Tp^{Ph,Me}). Vertically expanded inset shows the borohydride resonance.

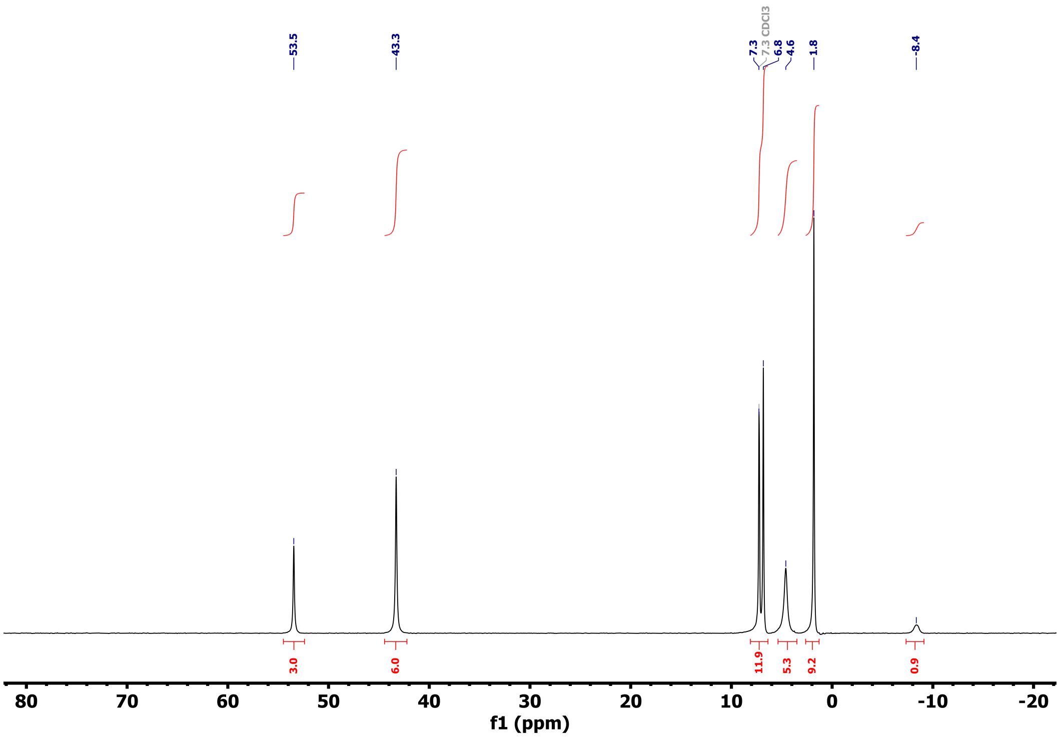

Figure S45. ^{1}H NMR spectrum ($CDCl_3$, 295 K) of [(Tp^{Ph,Me})NiS_2CNMe_2] (**1**).

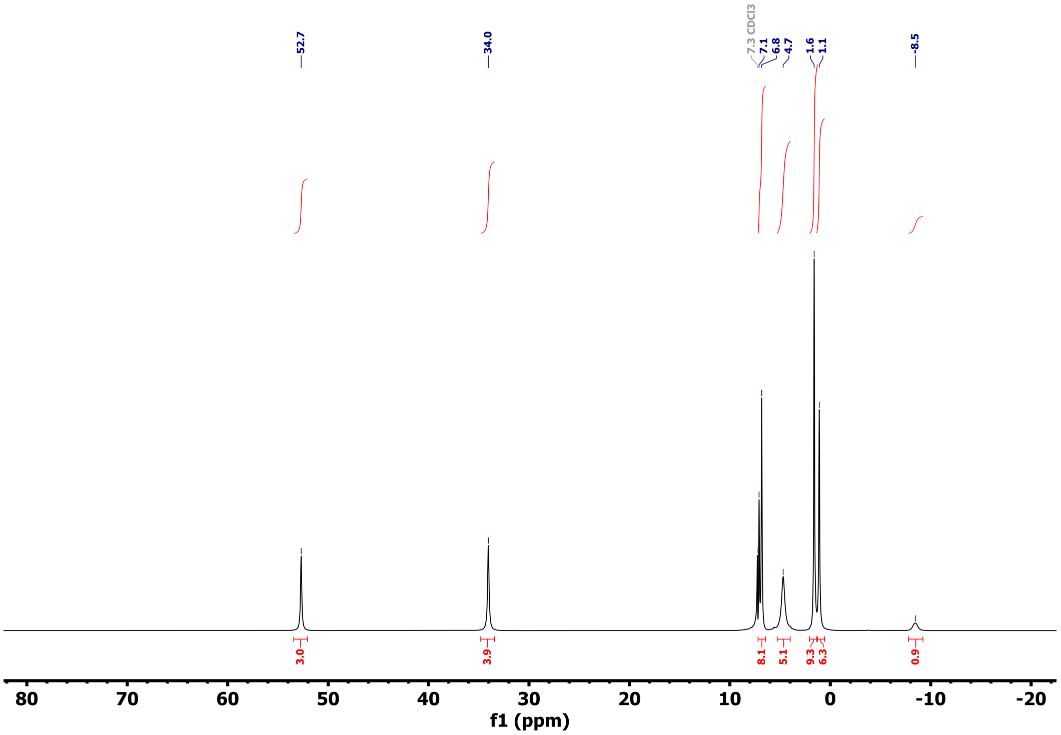

Figure S46. ^{1}H NMR spectrum ($CDCl_3$, 295 K) of [(Tp^{Ph,Me})NiS_2CNEt_2] (**2**).

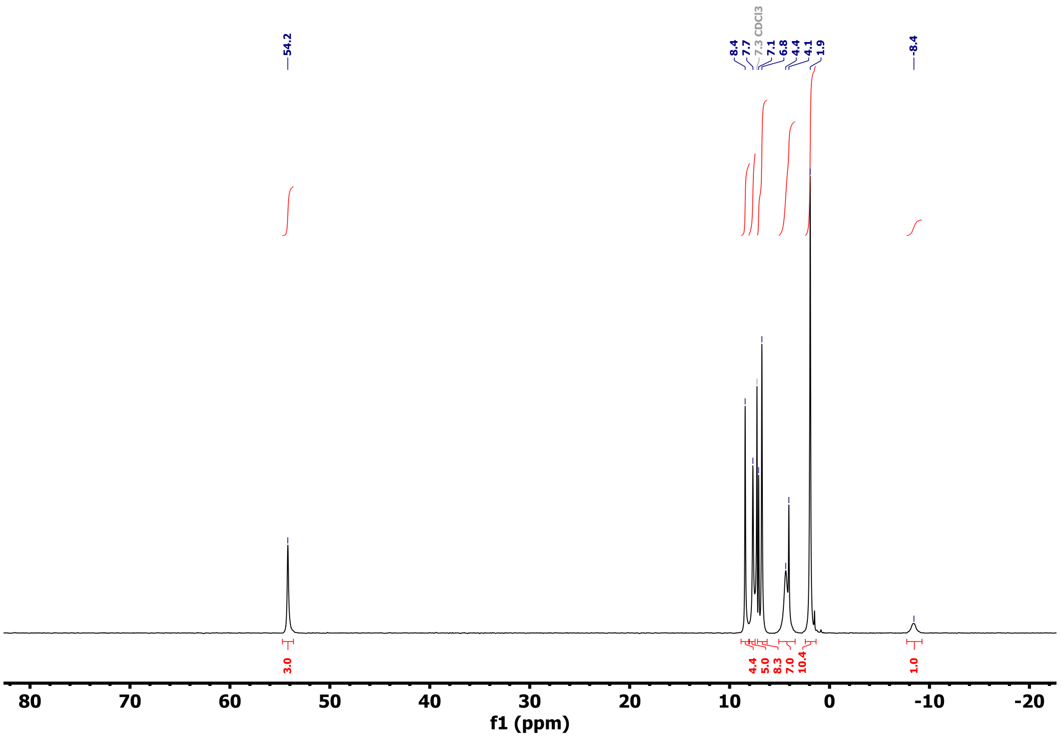

Figure S47. ^{1}H NMR spectrum ($CDCl_3$, 295 K) of [($Tp^{Ph,Me}$)NiS_2CNPh_2] (**3**).

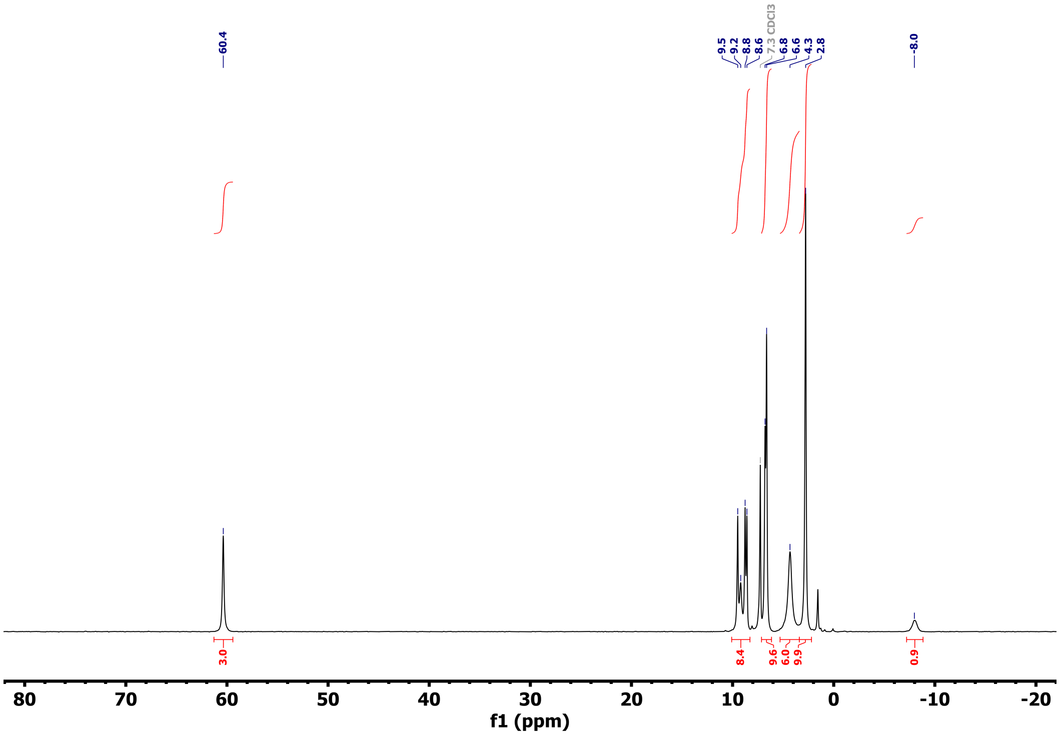

Figure S48. ^{1}H NMR spectrum ($CDCl_3$, 295 K) of [($Tp^{Ph,Me}$)$NiS_2CNC_{12}H_8$] (**4**).

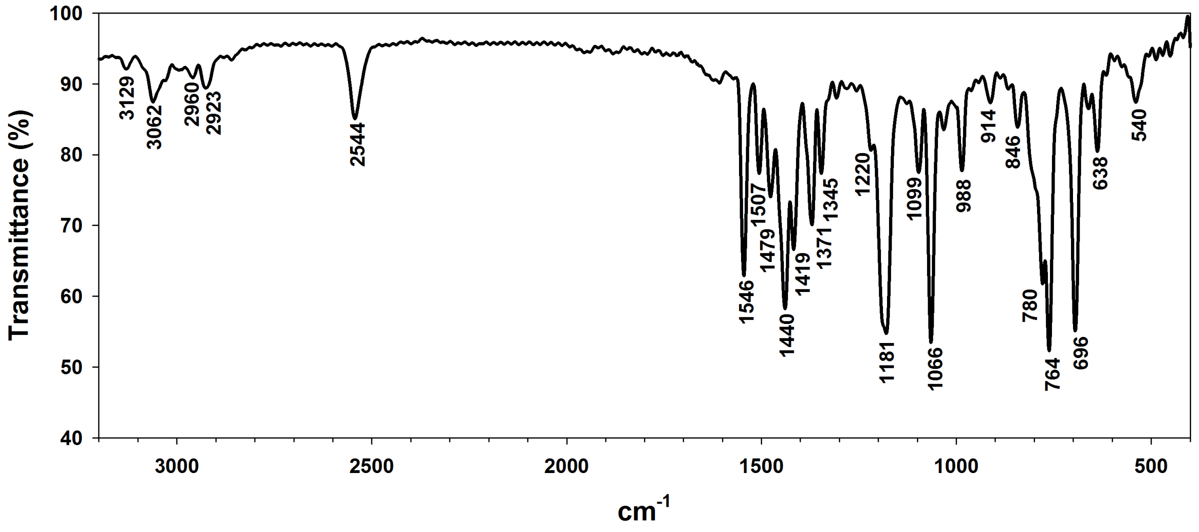

Figure S49. FTIR spectrum (KBr pellet) of [(Tp^{Ph,Me})Ni–Cl].

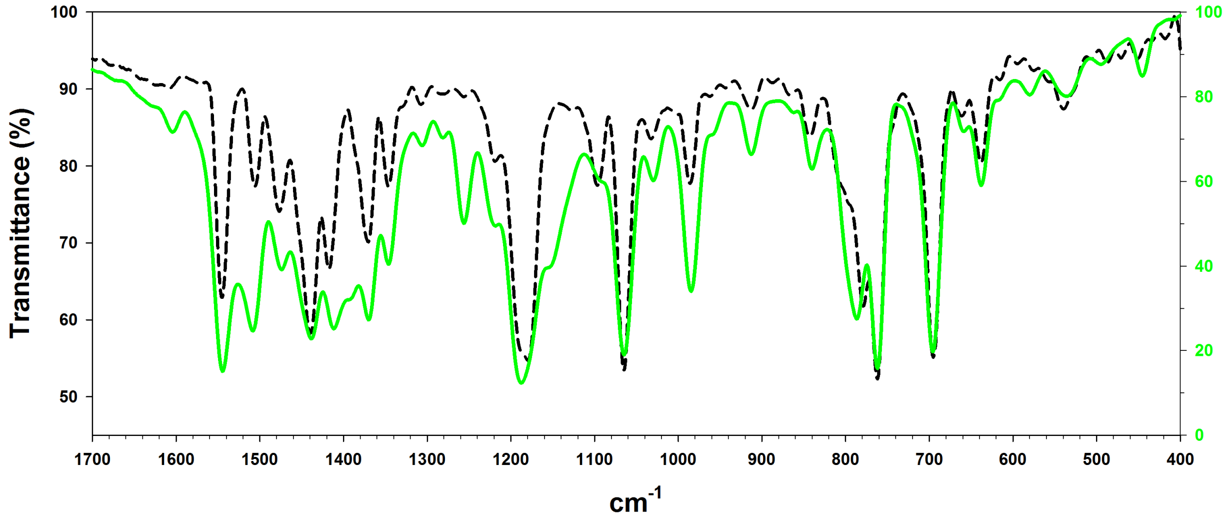

Figure S50. Comparison of FTIR spectra (KBr pellets) in the fingerprint region of complex **1** (solid green) and [(Tp^{Ph,Me})Ni–Cl] (dashed black).

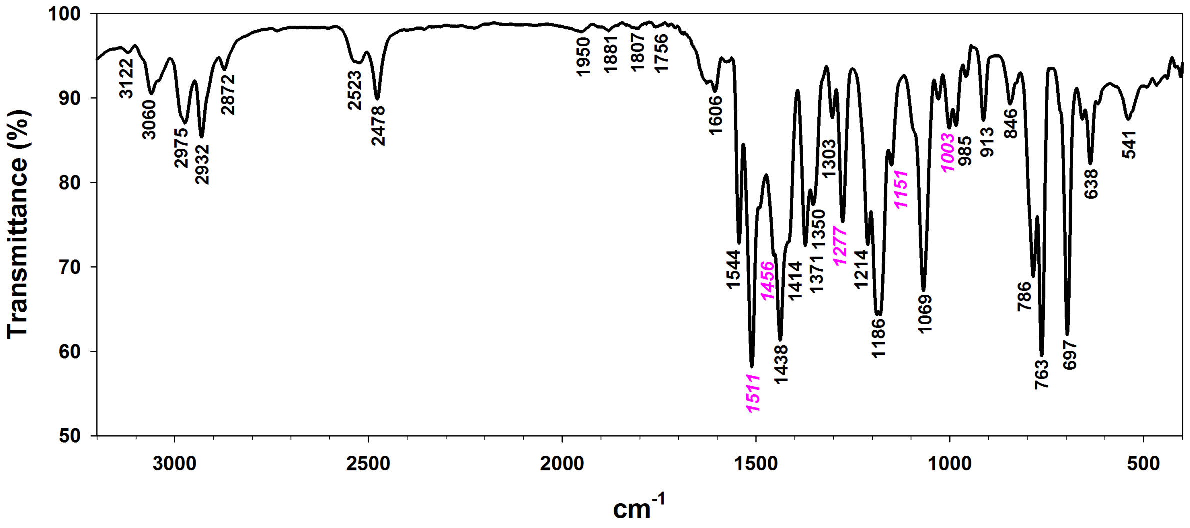

Figure S51. FTIR spectrum (KBr pellet) of complex **2**. Peaks labeled in italic pink do not coincide with any band in the spectrum of [(Tp^{Ph,Me})Ni–Cl] (Figure S49), and are assigned accordingly to modes within the diethyldithiocarbamate ligand.

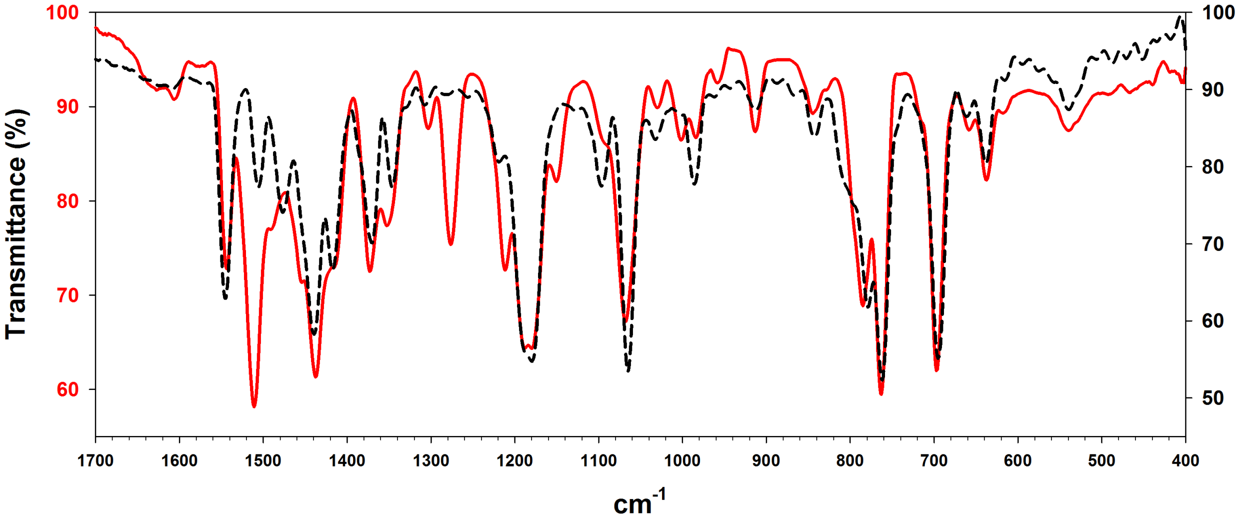

Figure S52. Comparison of FTIR spectra (KBr pellets) in the fingerprint region of complex **2** (solid black) and [(Tp^{Ph,Me})Ni–Cl] (dashed red).

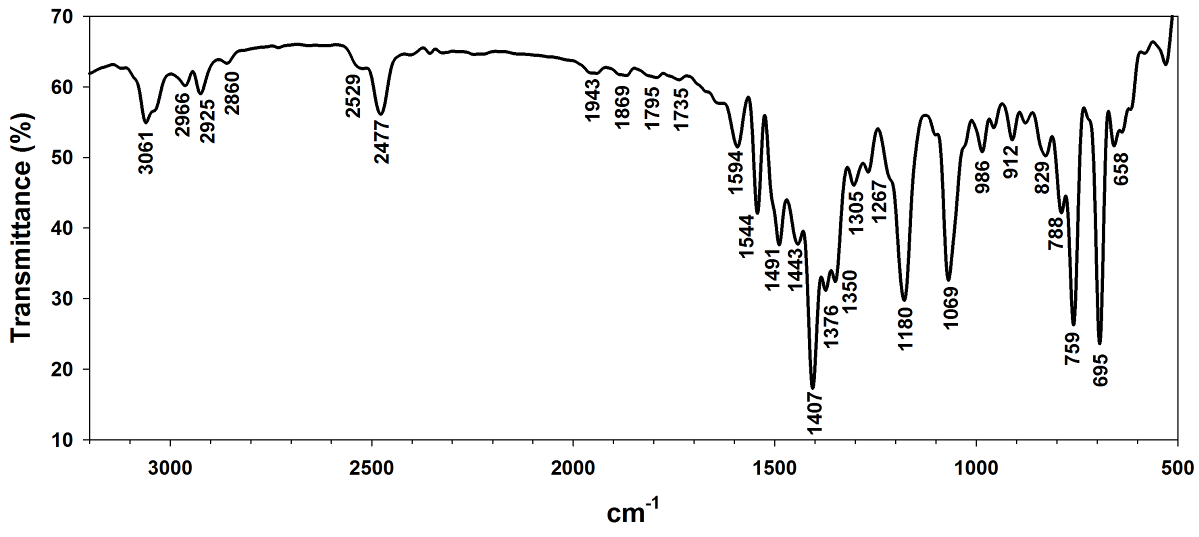

Figure S53. FTIR spectrum (KBr pellet) of red crystals of complex **3**.

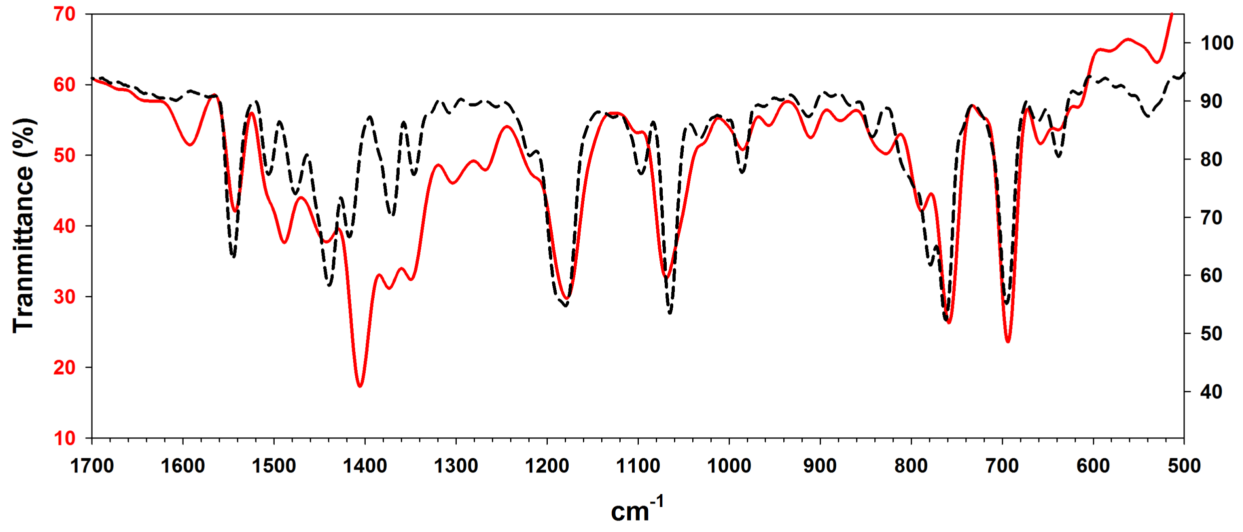

Figure S54. Comparison of FTIR spectra (KBr pellets) in the fingerprint region for red crystals of complex **3** (solid red) and [(Tp^{Ph,Me})Ni–Cl] (dashed black).

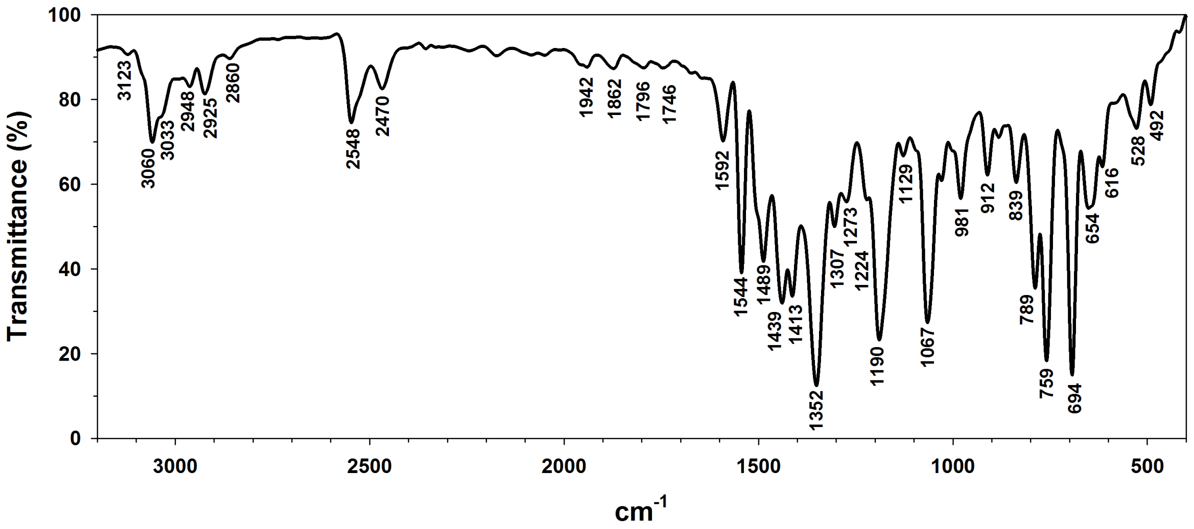

Figure S55. FTIR spectrum (KBr pellet) of green crystals of complex **3**.

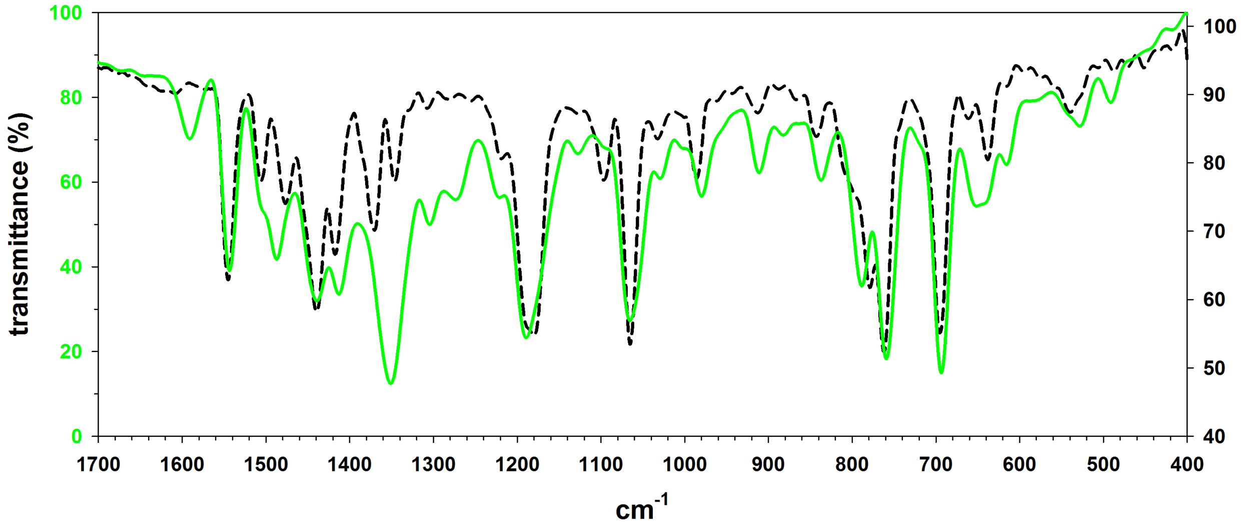

Figure S56. Comparison of FTIR spectra (KBr pellets) in the fingerprint region for green crystals of complex **3** (solid green) and [(Tp^{Ph,Me})Ni–Cl] (dashed black).

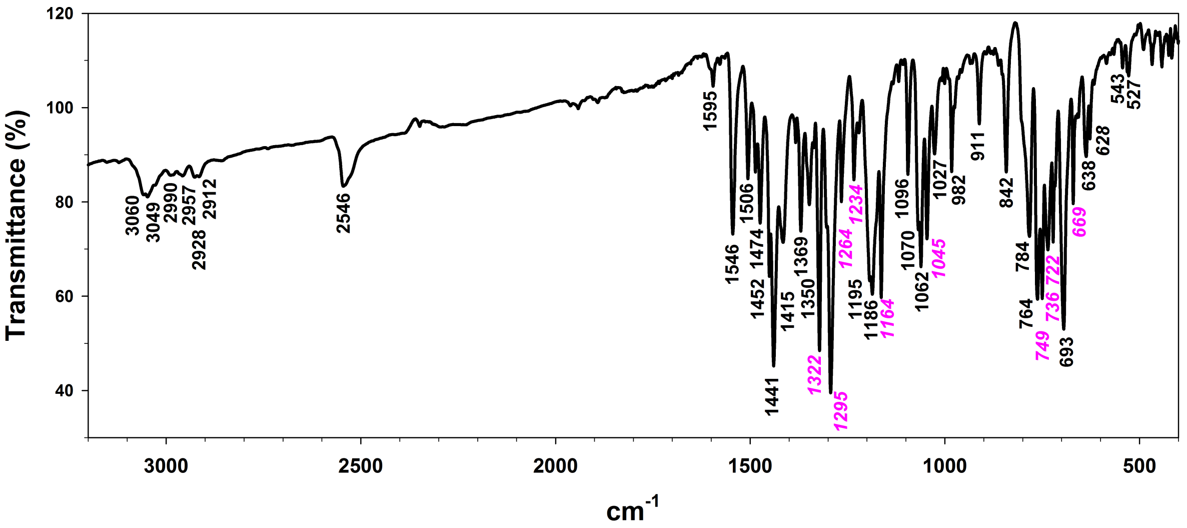

Figure S57. FTIR spectrum (KBr pellet) of green crystals of complex **4**. Peaks labeled in italic pink do not coincide with any band in the spectrum of [(Tp^{Ph,Me})Ni–Cl] (Figure S49), and are assigned accordingly to modes within the dithiocarbamate ligand.

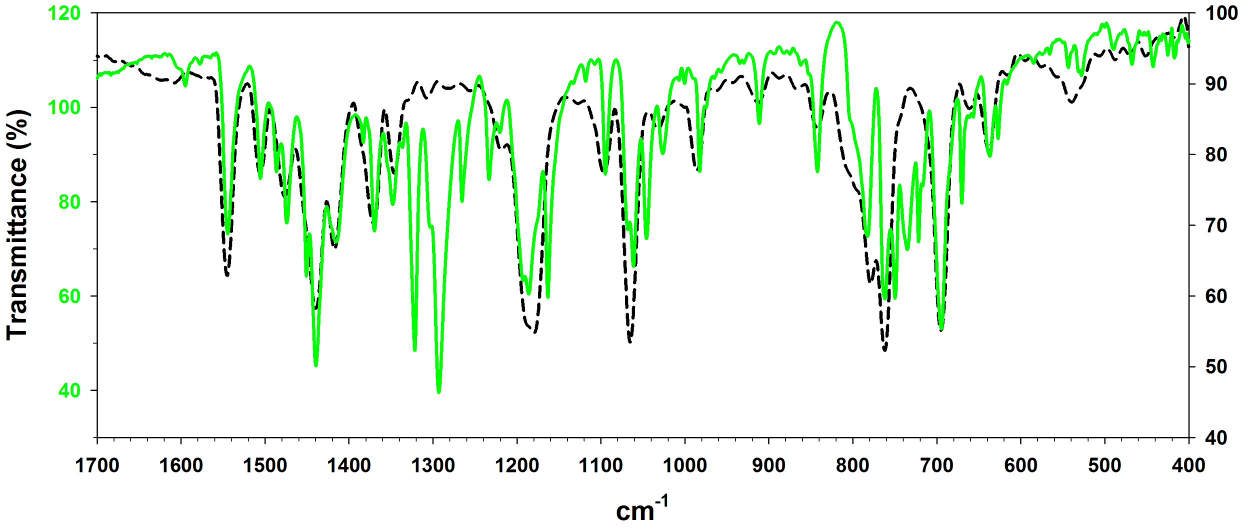

Figure S58. Comparison of FTIR spectra (KBr pellets) in the fingerprint region for green crystals of complex **4** (solid green) and [(Tp^{Ph,Me})Ni–Cl] (dashed black).

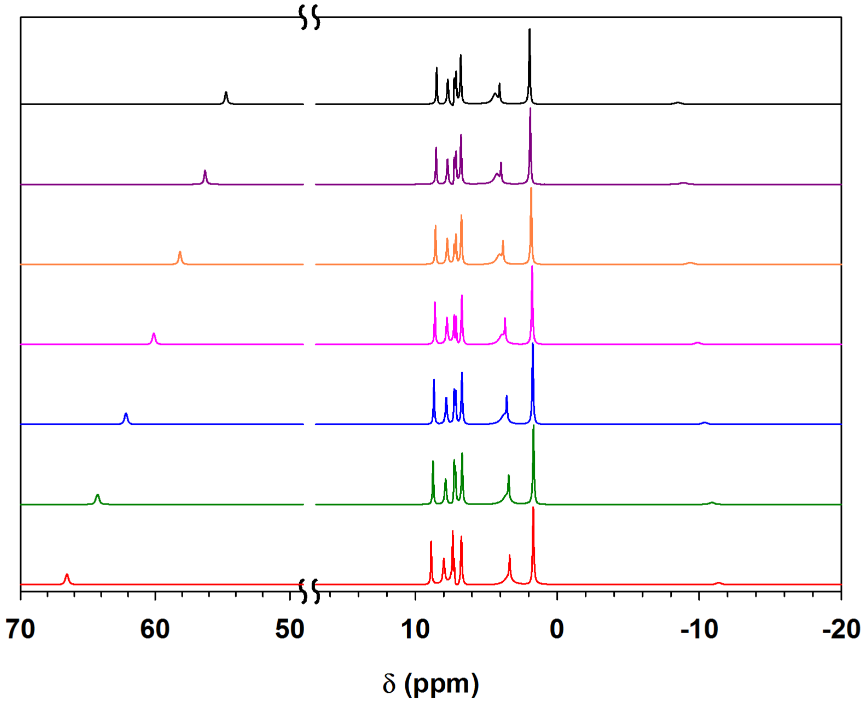

Figure S59. Temperature-dependent ^{1}H NMR spectra of **3**, recorded in 10° intervals from −35 (bottom, red) to 25 °C (top, black) in $CDCl_3$ solution.

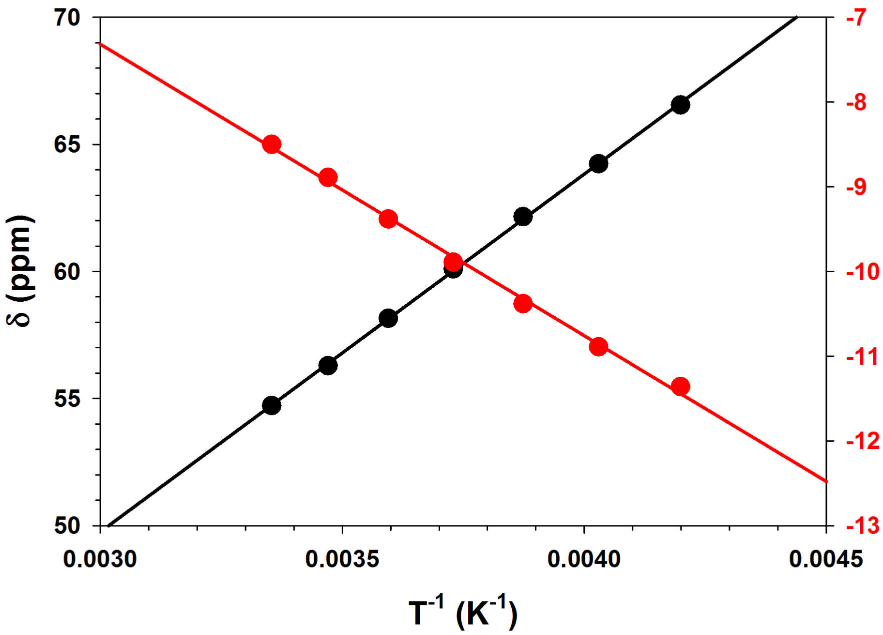

Figure S60. ^{1}H NMR chemical shifts of 4-pyrazolyl (black) and borohydride (red) resonances of **3** from Figure S59 as a function of inverse temperature. Solid lines are linear least-squares fits: 4–H, δ = 7.5(4) + [1.41(1) × 10^4]/T, r^2 = 0.9997; B–H, δ = 3.0(3) − [3.44(8) × 10^3]/T, r^2 = 0.9972.

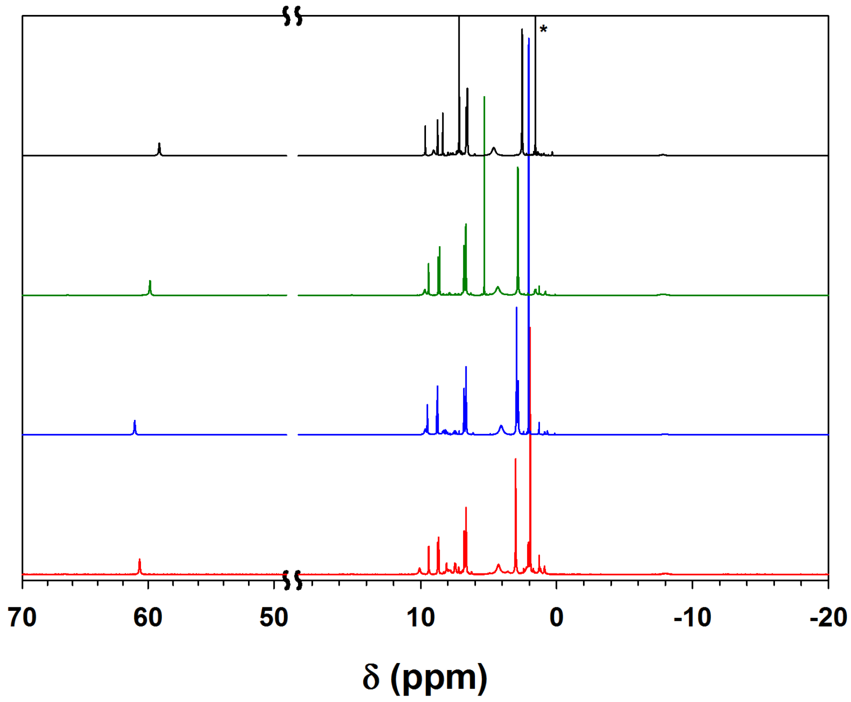

Figure S61. Solvent-dependent ^{1}H NMR spectra of **4**, recorded at 295 K in C_6D_6 (black, top), CD_2Cl_2 (green), d_6-acetone (blue) and CD_3CN (red, bottom). An acetone impurity peak in the top spectrum is denoted (*).

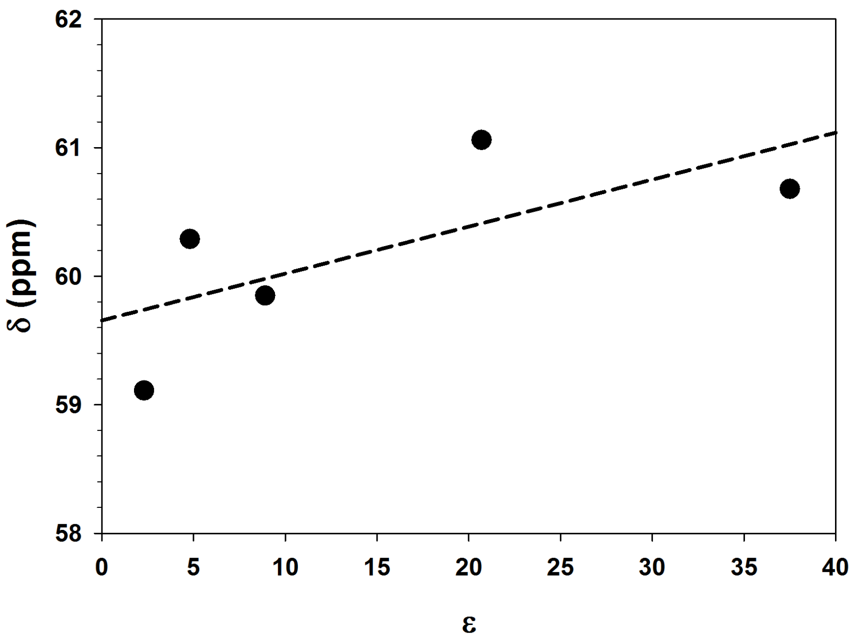

Figure S62. ^{1}H NMR chemical shift of the 4-pyrazolyl signal vs. solvent dielectric constant from the spectra of **4** at 295 K in Figure S61. The dashed line is a least-squares regression: δ = 59.7(4) + 0.04(2)ε, r^2 = 0.492.

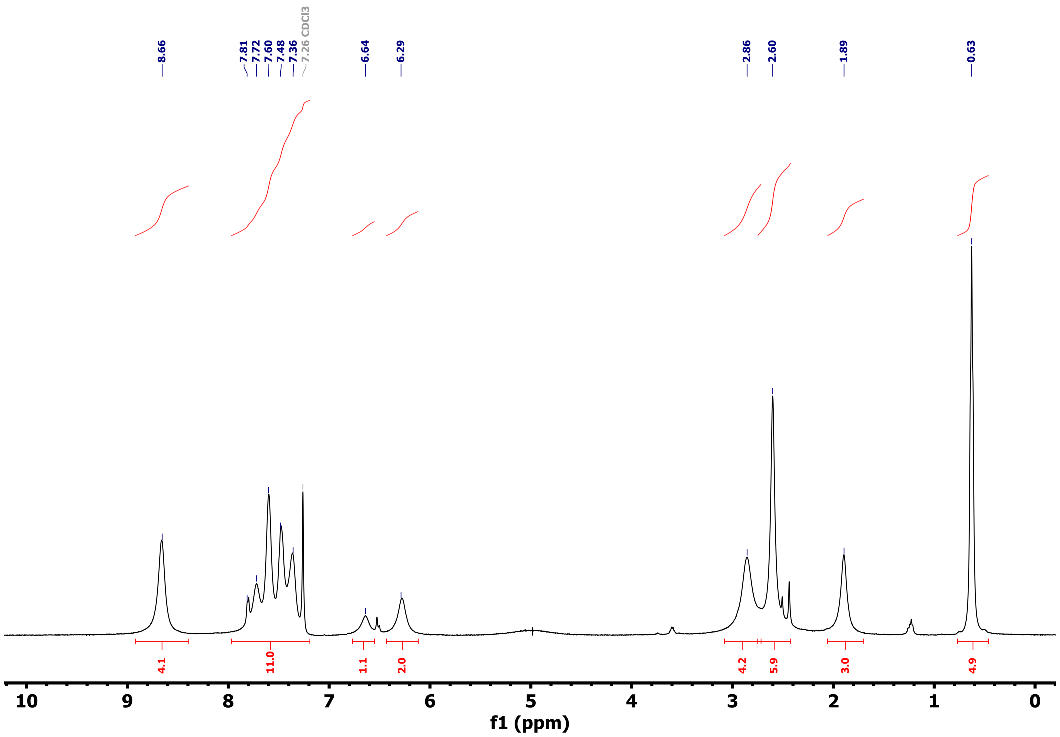

Figure S63. ^{1}H NMR spectrum ($CDCl_3$ + CF_3CO_2H, 295 K) of **2•H⁺**.

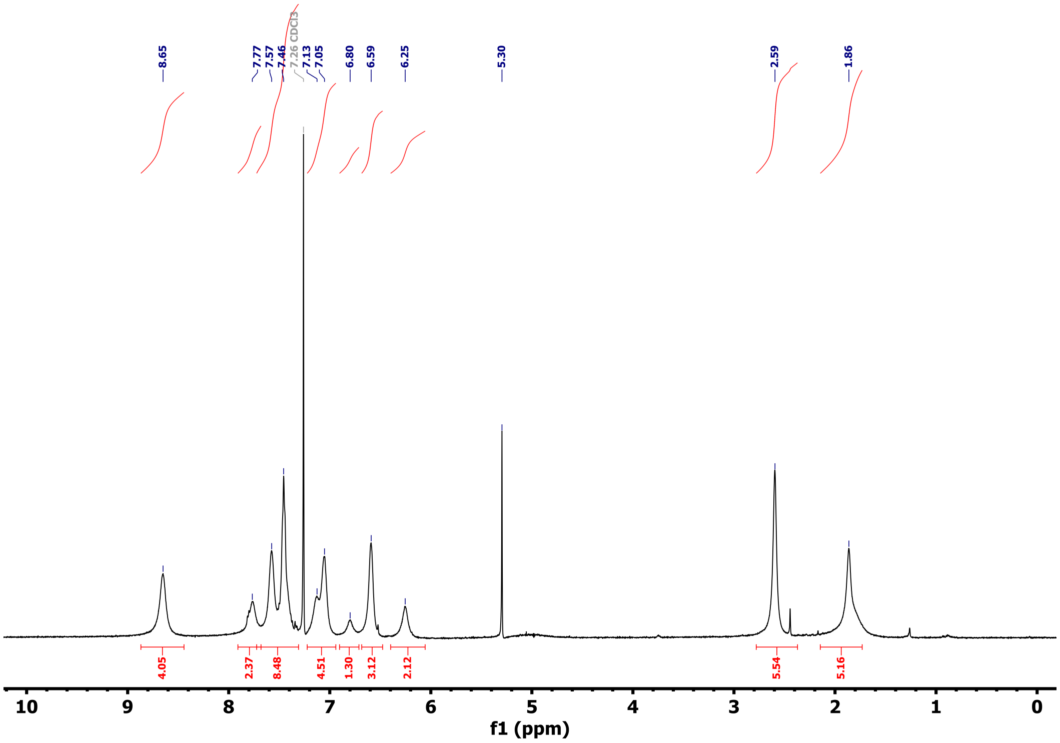

Figure S64. ^{1}H NMR spectrum ($CDCl_3$ + CF_3CO_2H, 295 K) of **3•H⁺**.

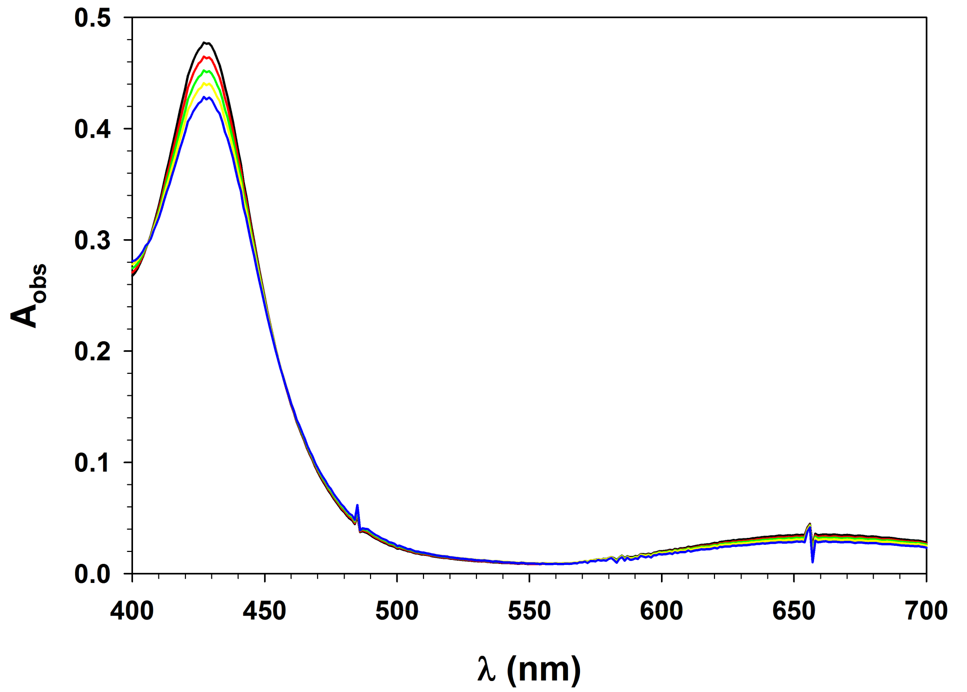

Figure S65. Observed temperature-dependent UV–vis spectra of complex **3** in MeCN, not corrected for change in solvent density (ΔT = 10 °C: 20 °C, blue; 60 °C).

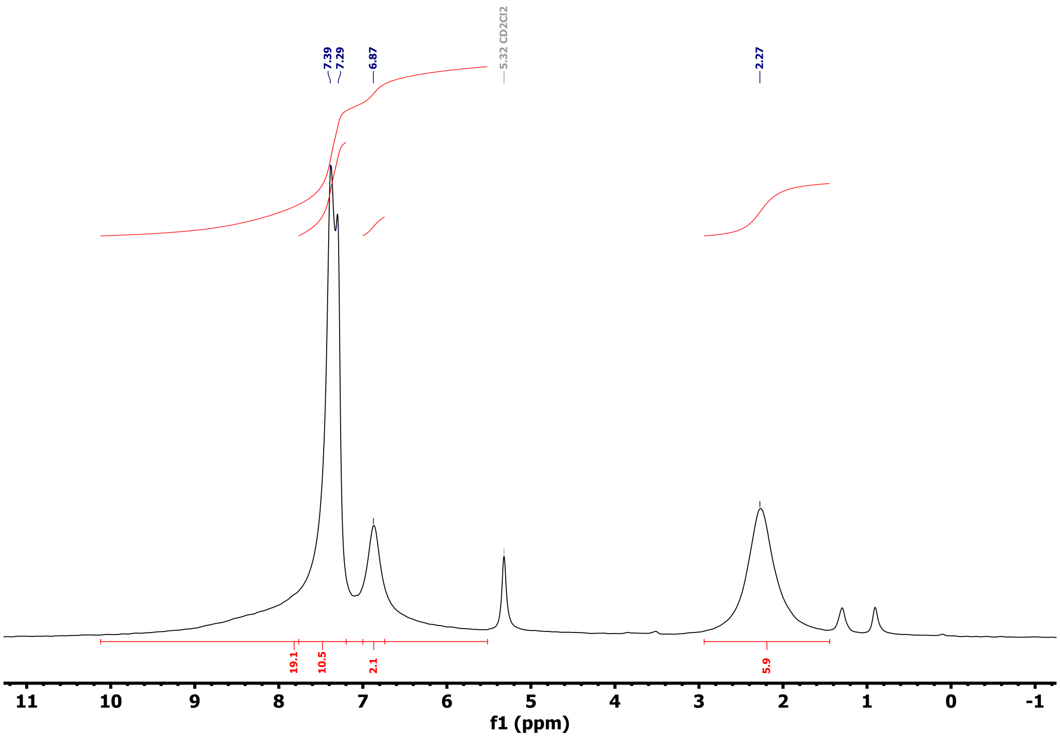

Figure S66. ^{1}H NMR spectrum (CD_2Cl_2, 295 K) of [(HB{pz^{Ph,Me}}$_2${OC(O)CF_3})NiS_2CNPh_2] (**5**).

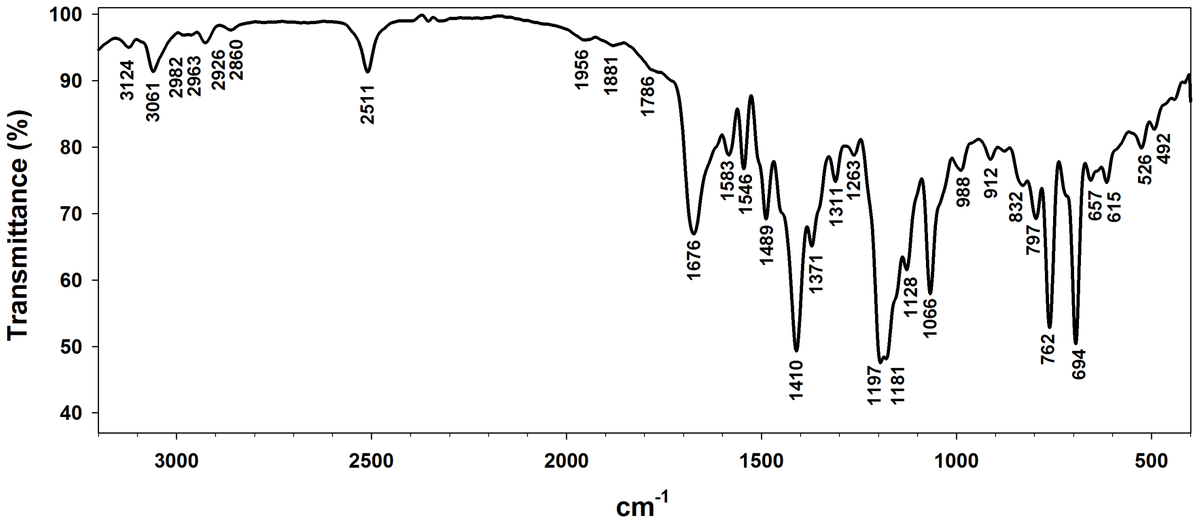

Figure S67. FTIR spectrum (KBr pellet) of complex **5**.

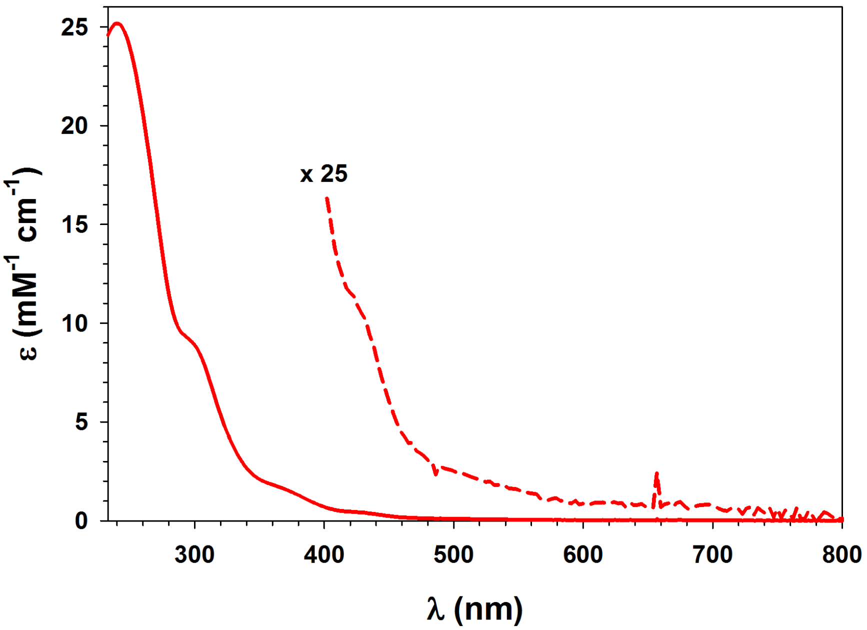

Figure S68. UV–vis–NIR spectrum (CH_2Cl_2, 295 K) of complex **5**.

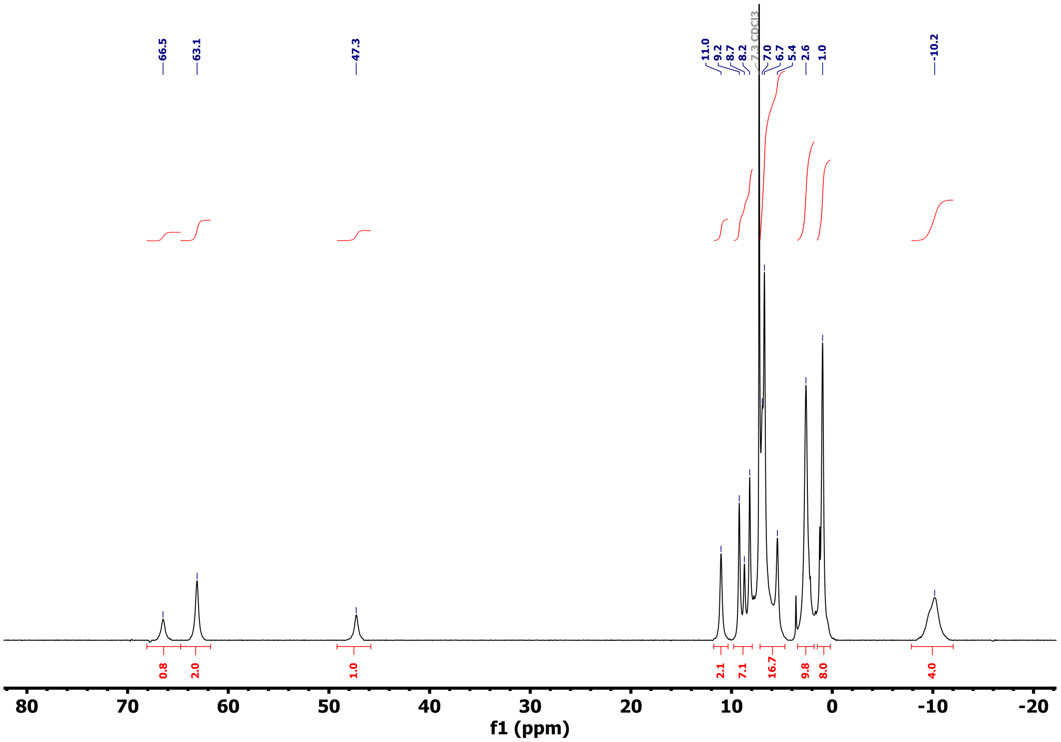

Figure S69. ^{1}H NMR spectrum ($CDCl_3$, 295 K) of [($Tp^{Ph,Me}$)Ni($Hpz^{Ph,Me}$)(OC{O}CF_3)] (**6**).

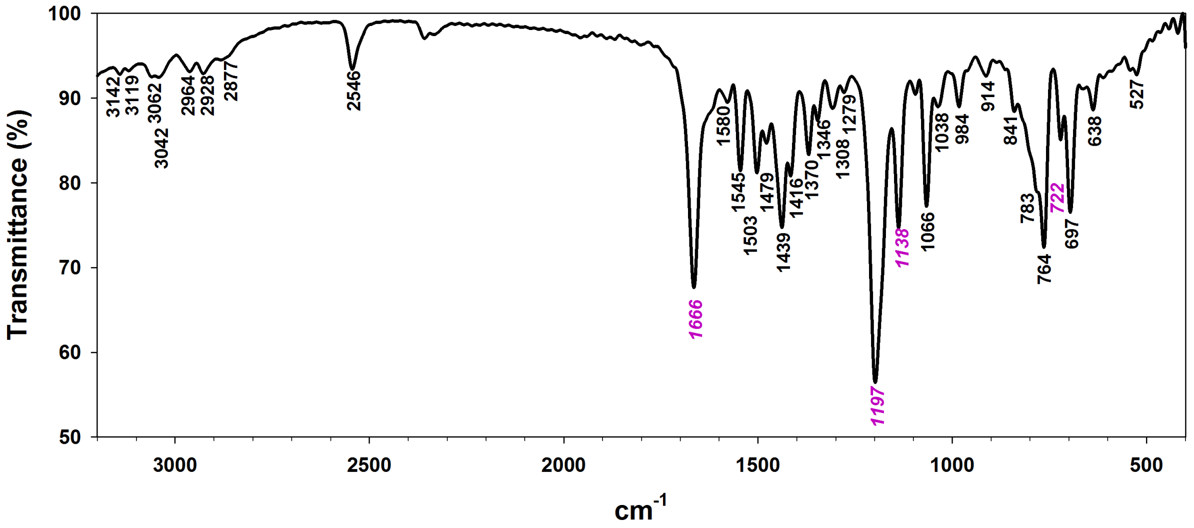

Figure S70. FTIR spectrum (KBr pellet) of complex **6**. Peaks labeled in italic purple do not coincide with any band in the spectrum of [(Tp^{Ph,Me})Ni–Cl] (Figure S49).

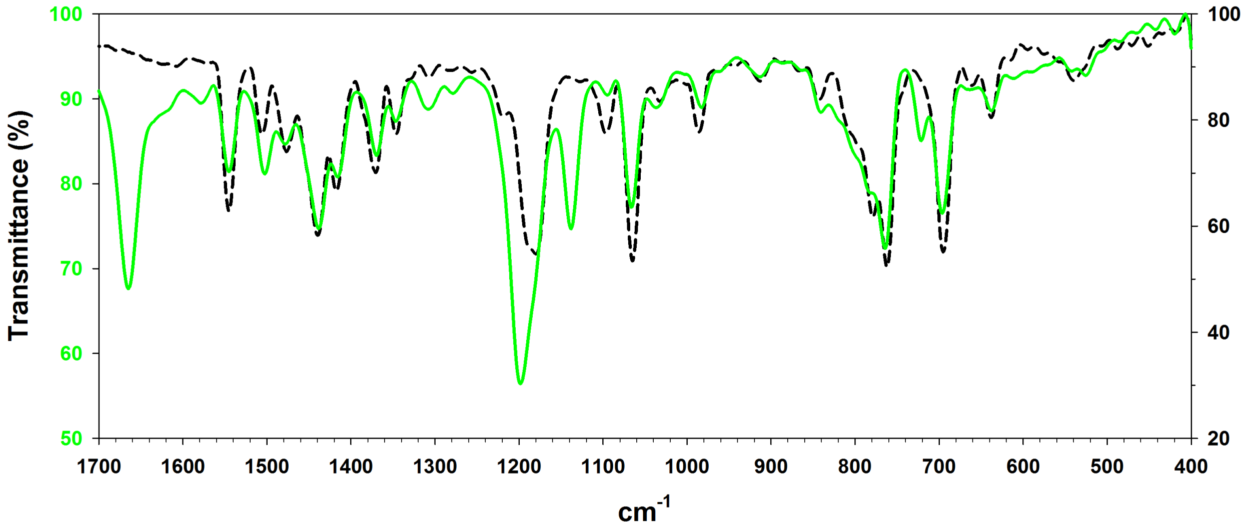

Figure S71. Comparison of FTIR spectra (KBr pellets) in the fingerprint region for complex **6** (solid green) and [(Tp^{Ph,Me})Ni–Cl] (dashed black).

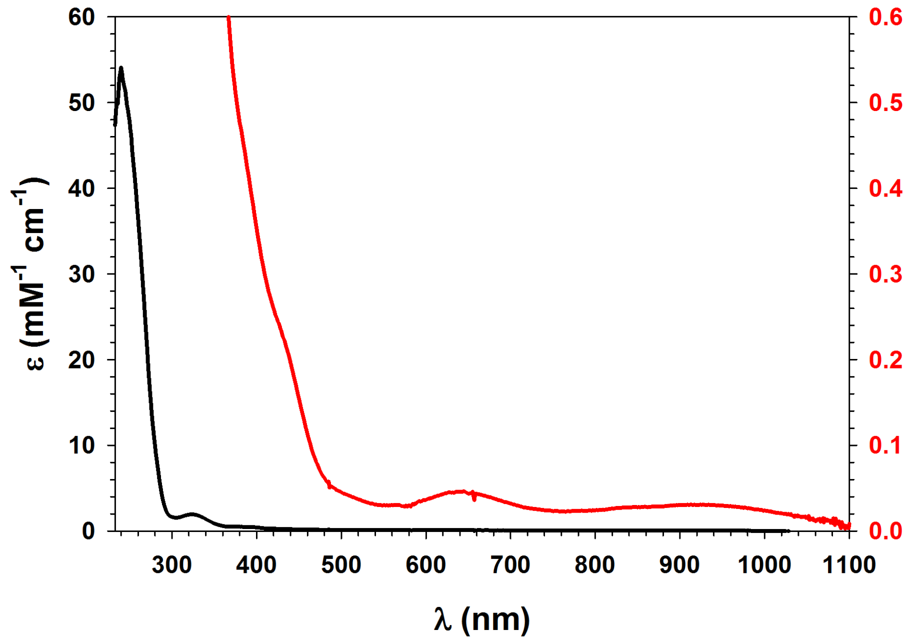

Figure S72. UV–vis–NIR spectra (CH_2Cl_2, 295 K) of complex **6**, at concentrations of 0.06 (black) and 0.40 (red) mM.

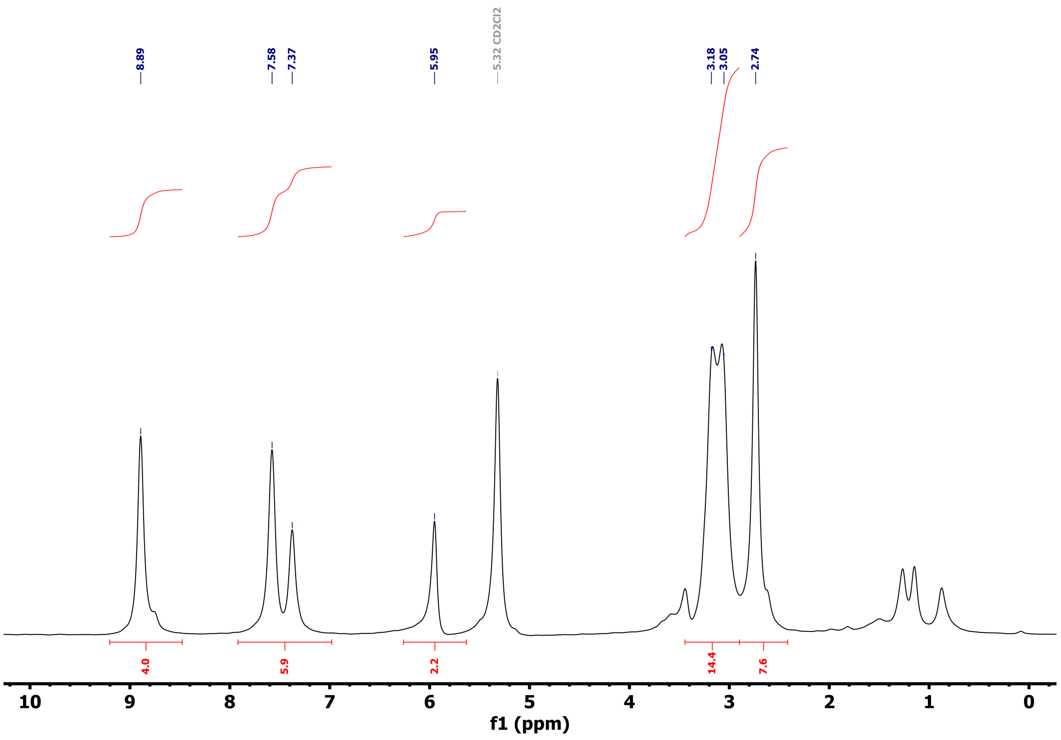

Figure S73. ^{1}H NMR spectrum (CD_2Cl_2, 295 K) of [(μ-pz^{Ph,Me})NiS_2CNMe_2]$_2$ (**7**).

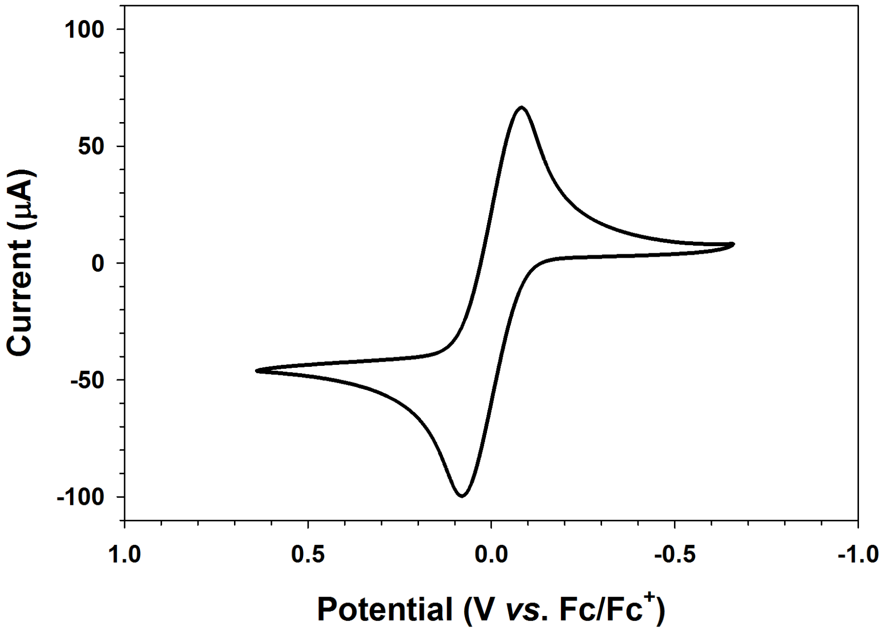

Figure S74. Cyclic voltammogram of ferrocene (CH_2Cl_2, 0.1 M $^{n}Bu_4NPF_6$, 100 mV/s, 295 K).

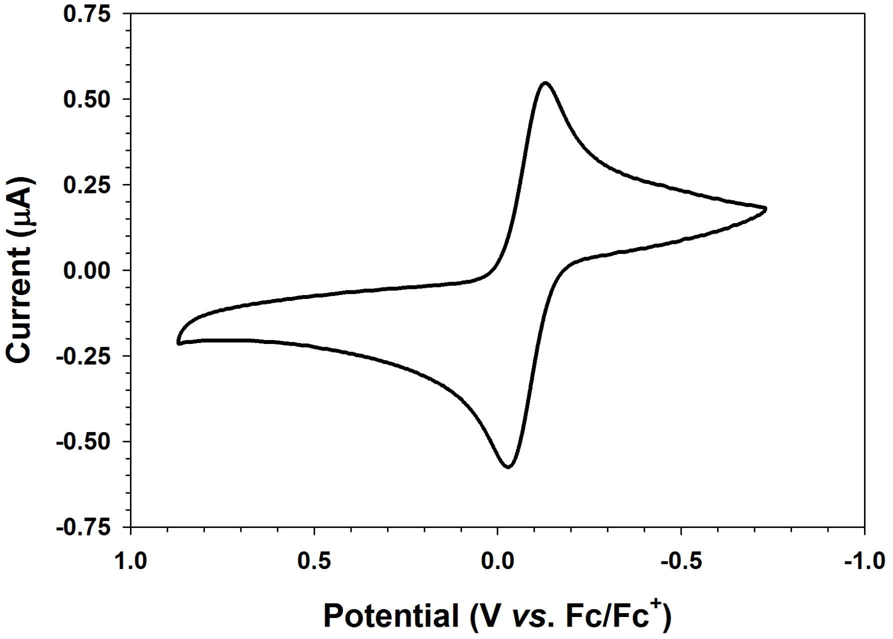

Figure S75. Cyclic voltammogram of **2** (CH_2Cl_2, 0.1 M $^{n}Bu_4NPF_6$, 50 mV/s, 295 K).

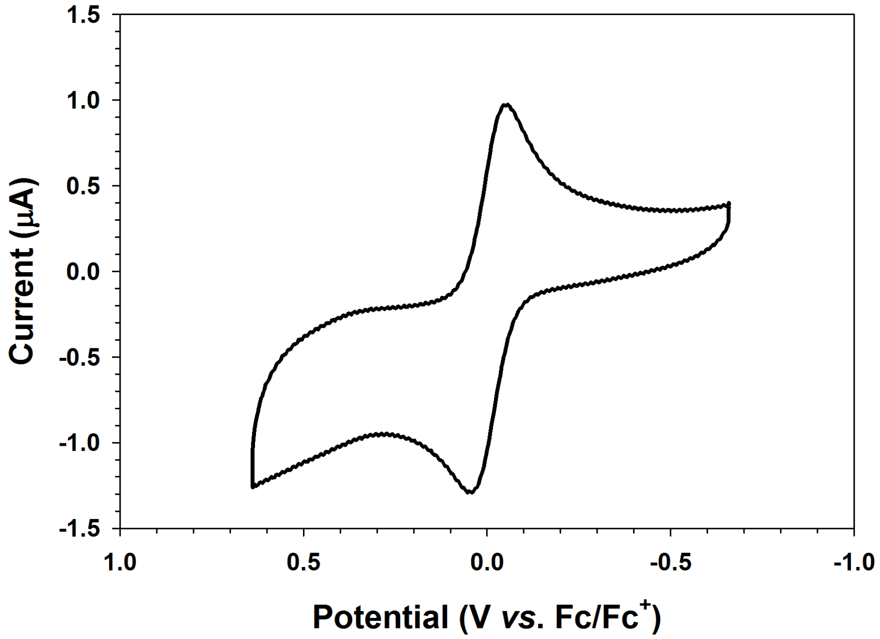

Figure S76. Cyclic voltammogram of **3** (CH_2Cl_2, 0.1 M $^{n}Bu_4NPF_6$, 100 mV/s, 295 K).

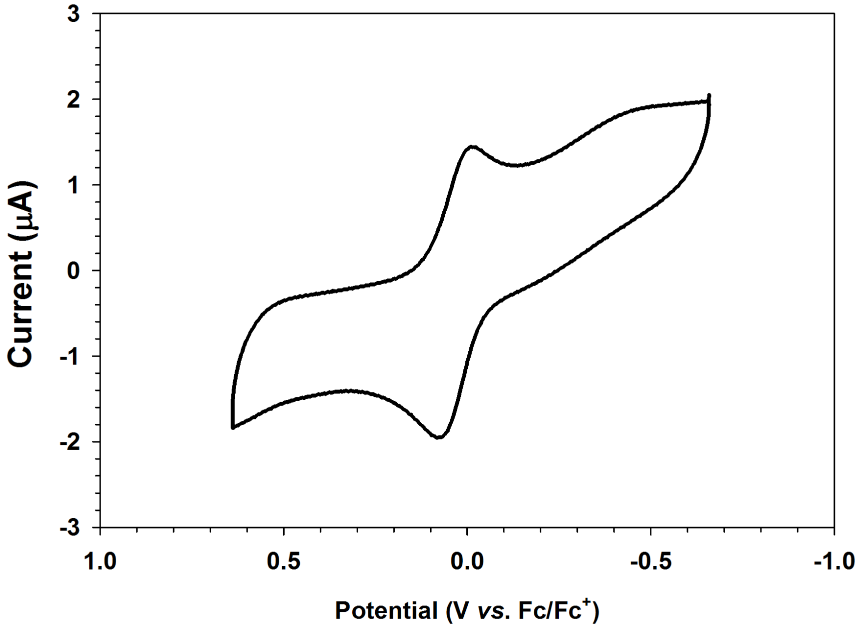

Figure S77. Cyclic voltammogram of **5** (CH_2Cl_2, 0.1 M $^{n}Bu_4NPF_6$, 100 mV/s, 295 K).

Table S55. Coordinate bond angles (°) of base-off NiSOD vs. analogous models.

dataset/model	N1–Ni–N2	S2–Ni–S6	N2–Ni–S2	N1–Ni–S6	N1–Ni–S2	N2–Ni–S6	ref.
base-off NiSOD (X-ray)							
1T6U.pdb	83.6(1.6)	95.0(6)	88.2(7)	93.3(1.3)	170.5	176.3	17,18
1Q0M.pdb	82.1	96.5	88.6	93.1	170.4	169.7	19,21
base-off Ni(II) hook (DFT)							
Ni^{II}-off-ls	82.9	98.9	89.0	88.4	168.1	170.3	37
red	83.8	99.6	89.2	87.5	173.0	170.0	24
red-S6H	84.7	90.6	88.3	96.0	172.8	167.7	24
4′	82.5	100.7	89.0	87.1	171.0	164.4	30
C	81.2	94.7	87.8	96.4	168.9	175.7	(a)
base-off Ni(II) synthetic complexes (X-ray)							
**2** (293 K)	90.3	78.1	96.2/95.7		172.5/172.9		(a)
**3** • ½ MeCN	90.3	78.8	94.4/95.6		170.7/171.3		(a)
**5**	90.5	78.7	93.4/96.7		171.0/171.0		(a)
**7** (Ni1)	90.5	79.0	94.2/96.2		172.9/174.9		(a)
**7** (Ni 2)	90.1	78.9	94.0/96.9		172.5/175.5		(a)
base-off Ni(II) synthetic models (DFT)							
1	93.7	77.7	94.2/94.4		171.8/171.9		(a)
8	93.9	77.9	94.1		171.9		(a)

(a) this work.

Table S56. Coordinate bond angles (°) of base-on NiSOD vs. analogous models.

dataset/model	N1$_{eq}$–Ni–N2	S2–Ni–S6	N2–Ni–S2	N1$_{eq}$–Ni–S6	N1$_{eq}$–Ni–S2	N2–Ni–S6	N1$_{ax}$–Ni–N1$_{eq}$	N1$_{ax}$–Ni–N2	N1$_{ax}$–Ni–S2	N1$_{ax}$–Ni–S6	ref.
base-on NiSOD (X-ray)											
1T6U.pdb	81.5(1.4)	95.0(6)	88.2(7)	95.8(1.1)	168.2	176.3	82(2)	82(2)	107(2)	94(2)	17,18
1Q0D.pdb	81.5	92.4	88.2	98.9	167.1	171.5	77	76	108	96	19,20
Ni(III) hook (DFT)											
ox[1]	81.0	96.5	88.0	90.7	162.6	164.2	86.2	94.0	108.2	99.0	24
Ni^{III}-on	81.6	95.8	88.3	91.9	166.0	165.5	86.8	93.0	103.4	99.6	37
1	82.2	97.1	87.6	90.5	166.6	164.0	86.9	94.7	102.6	99.1	30
D	81.6	95.2	86.7	92.3	165.3	157.4	88.8	89.0	99.9	112.8	(a)
Ni(III) synthetic model											
6	90.9	76.9	94.7		165.4		90.1		103.3		(a)
base-on, low-spin Ni(II) hook (DFT)											
B	83.7	94.4	88.1	93.6	171.8	170.3	73.9	85.1	104.9	85.2	(a)
base-on, low-spin Ni(II) synthetic complexes (X-ray)											
**2** • CH_2Cl_2	90.8	78.5	94.5/96.3		172.6/174.7		79.5/88.5		93.0/98.6		(a)
**4** (red)	90.2	77.7	94.3/98.0		171.8/173.0		85.1/85.4		89.5/96.6		(b)
base-on, low-spin Ni(II) synthetic model (DFT)											
2	93.0	77.7	94.6		171.9		79.0		105.2		(a)
base-on, high-spin Ni(II) hook (DFT)											
Ni^{II}-on-hs	76.6	102.7	87.4	90.9	163.2	158.7	86.5	92.0	99.4	104.5	37
4	80.8	101.8	81.9	91.4	160.4	159.5	86.5	92.9	103.5	105.7	30
A	78.1	94.8	85.3	100.2	157.7	174.1	89.1	89.5	105.7	96.1	(a)
base-on, high-spin Ni(II) synthetic complexes (X-ray)											
**1** (Ni 1, 293 K)	84.3	75.2	97.3/100.2		155.5/172.1		90.7/95.5		96.8/108.5		(a)
base-on, high-spin Ni(II) synthetic model (DFT)											
3	88.5	74.6	96.4		163.1		89.4		106.9		(a)

(a) this work. (b) Figure S37.

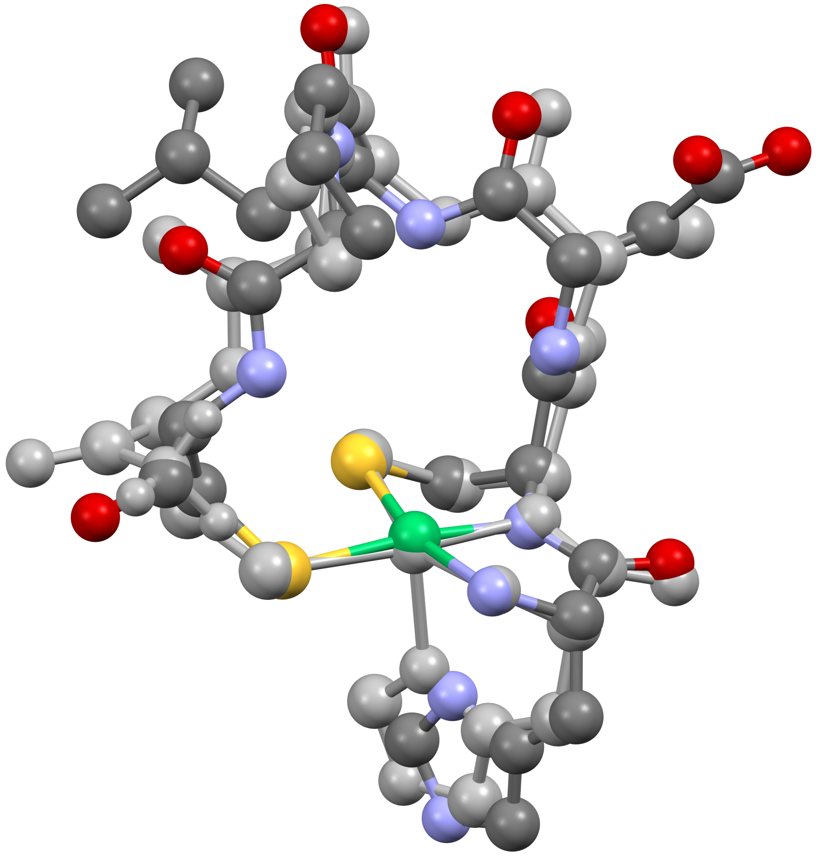

Figure S78. Least-squares overlay of the equatorial donor atoms in the experimental NiSOD base-on conformation (1Q0D.pdb, in color)[19,20] with the base-on, high-spin Ni(II) model *A* (rendered in gray). Hydrogen atoms are omitted for clarity, except for those on the C-terminal amide, one of which is hydrogen bonded to the Cys-6 thiolate sulfur atom.

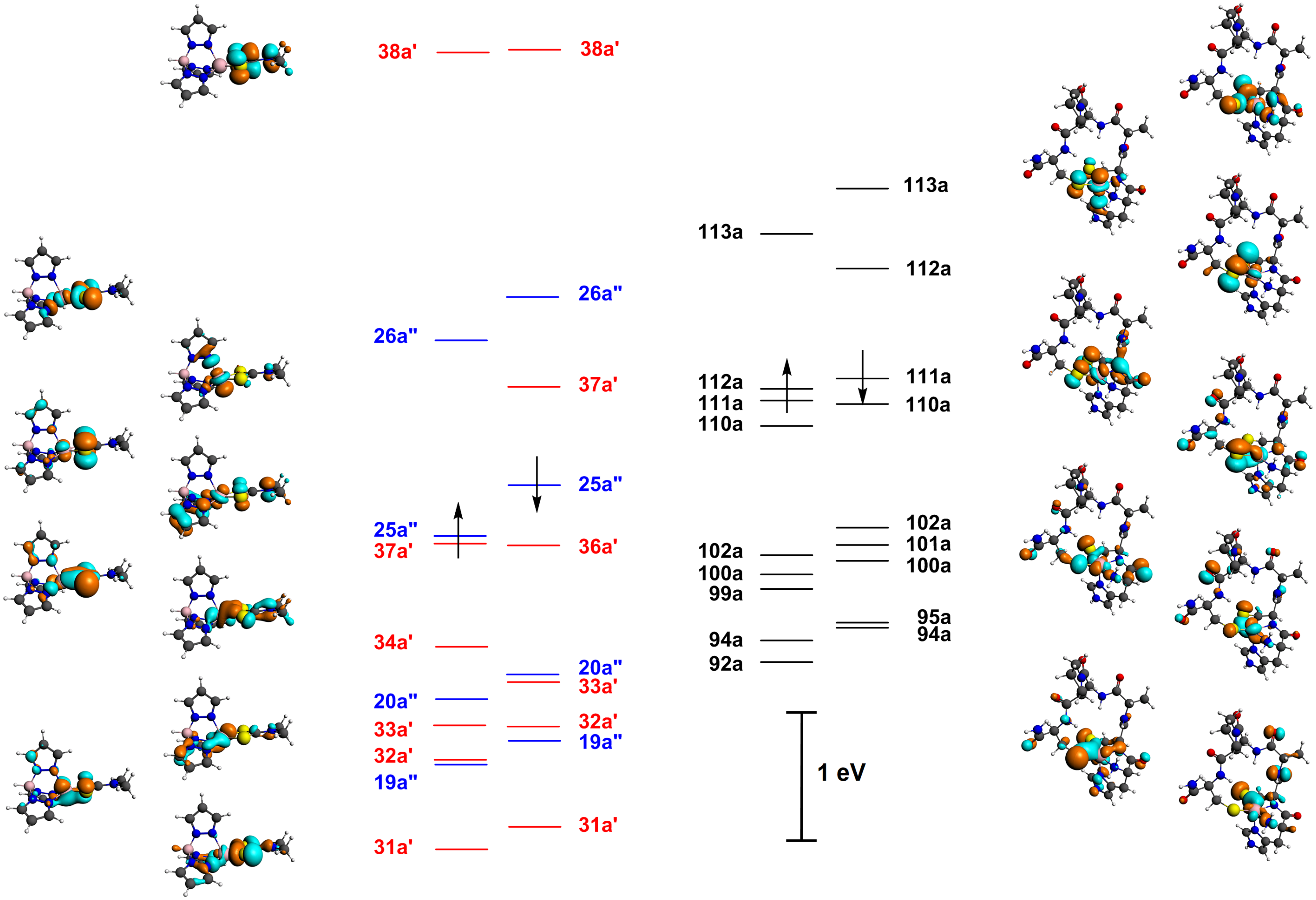

Figure S79. Comparison of select Ni- and S-centered frontier orbitals for the oxidized nickel hook model *D* (right) and the synthetic analogue model *6* (left). Relative energies are indicated at center (those of *6* are color-coded by mirror symmetry), and isocontour plots of β-spin orbitals are shown on the periphery (those of *6* are separated by mirror symmetry).

Table S57. Observed bands for complex **5** vs. calculated transitions for model *8*.[a]

Band	λ_{max} (nm/cm^{-1}, obs)	ε (M^{-1} cm^{-1})	Transition[b]	λ_{max} (cm^{-1}/nm, calc)	ε, calc [c]	orbitals	assignment[d]
A	510 / 19600	90	1A'	16050 / 620	40	22a″ → 23a″	LF: Ni 3d$_{\delta}$* → 3d$_{x2-y2}$
			1A″	17050 / 590	10	27a′ → 23a″	LF: Ni 3d$_{\pi}$* → 3d$_{x2-y2}$
						28a′ → 23a″	LF: Ni 3d$_{z2}$ → 3d$_{x2-y2}$
B	420 / 23600	460	4A'	24500 / 410	5100	27a′ → 29a′	MLCT: Ni 3d$_{\pi}$* → 4b$_1$ π*
C	360 / 27600	1800	7A″	29300 / 340	1200	22a″ → 30a′	MLCT: Ni 3d$_{\delta}$* → pyrazole π*
			8A'	30840 / 324	1300	27a′ → 30a′	MLCT: Ni 3d$_{\pi}$* → pyrazole π*
						22a″ → 24a″	MLCT: Ni 3d$_{\delta}$* → pyrazole π*
D	300 / 33360	8800	11A'	33150 / 300	8000	22a″ → 24a″	MLCT: Ni 3d$_{\delta}$* → pyrazole π*
						27a′ → 30a′	MLCT: Ni 3d$_{\pi}$* → pyrazole π*
E	270 / 37420	16000	13A'	36500 / 270	27000	19a″ → 23a″	LMCT: 6b$_2$ σ → 3d$_{x2-y2}$
						18a″ → 23a″	LMCT: pyrazole π → 3d$_{x2-y2}$
						28a′ → 31a′	MLCT: Ni 3d$_{z2}$ → pyrazole π*

(a) Figure S80. (b) Table S54. (c) $f \times 10^5$. (d) Table S46.

Table S58. Observed bands for complex **1** vs. calculated transitions for model *3*.[a]

Band	λ_{max} (nm/cm^{-1}, obs)	ε (M^{-1} cm^{-1})	Transition[b]	λ_{max} (cm^{-1}/nm, calc)	ε, calc [c]	orbitals	assignment[d]
A	650 / 15360	100	1A'	12470 / 800	640	36a′ → 37a′ β	LF: Ni 3d$_{\pi}$* → 3d$_{z2}$
B	430 / 23490	800	3A'	18390 / 540	600	25a″ → 26a″ β	LF: Ni 3d$_{\delta}$* → 3d$_{x2-y2}$
			4A'	19230 / 520	350	34a′ → 37a′ β	LMCT: 3b$_1$ π → 3d$_{z2}$
C[e]	360 / 27450	1300	6A'	22580 / 430	1610	24a″ → 26a″ β	LMCT: 2a$_2$ δ → 3d$_{x2-y2}$
			7A'	23540 / 420	1090	32a′ → 37a′ β	LMCT: pyrazole π → 3d$_{z2}$
D[e]	300 / 33290	8500	13A'	28650 / 350	3270	20a″ → 26a″ β	LMCT: pyrazole π → 3d$_{x2-y2}$
						22a″ → 26a″ β	LMCT: 6b$_2$ σ → 3d$_{x2-y2}$
			14A'	29830 / 340	8390	22a″ → 26a″ β	LMCT: 6b$_2$ σ → 3d$_{x2-y2}$
						20a″ → 26a″ β	LMCT: pyrazole π → 3d$_{x2-y2}$
						37a′ → 38a′ α	MLCT: Ni 3d$_{\pi}$* → 4b$_1$ π*
			15A'	30810 / 320	7840	35a′ → 38a′ β	MLCT: Ni 3d$_{xy}$ → 4b$_1$ π*
						37a′ → 38a′ α	MLCT: Ni 3d$_{\pi}$* → 4b$_1$ π*
						36a′ → 38a′ β	MLCT: Ni 3d$_{\pi *}$ → 4b$_1$ π*

(a) Figure S81. (b) Table S50. (c) $f \times 10^5$. (d) Table S41. (e) shoulder.

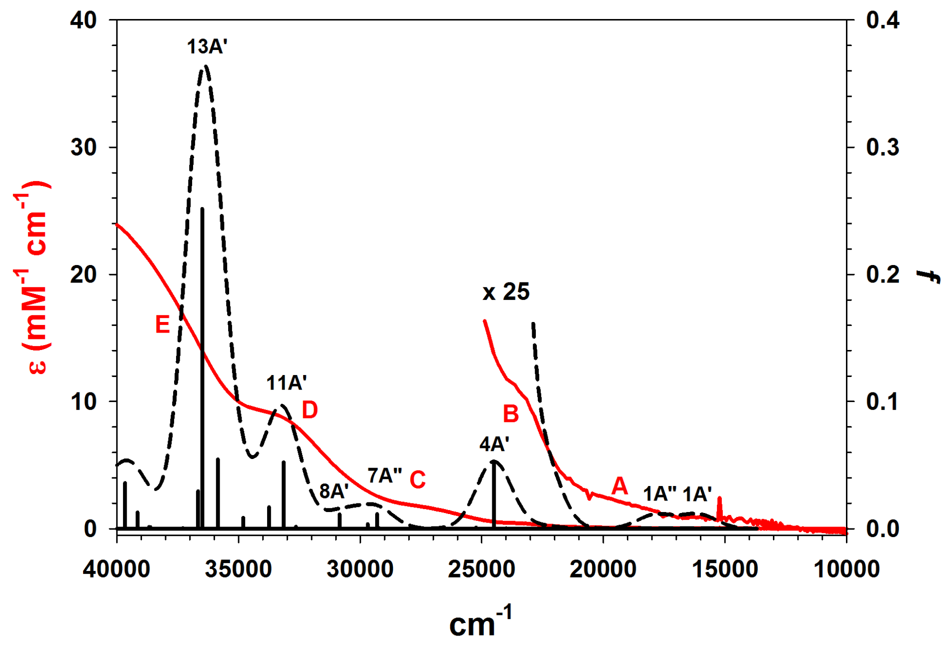

Figure S80. Comparison of experimental UV–vis–NIR spectrum of complex **5** (red), with five resolved features labeled A–E, and the calculated transitions for the corresponding DFT model *8* (black), rendered with an arbitrary linewidth of 1700 cm^{-1} (dashed), and with prominent transitions labeled (Table S54).

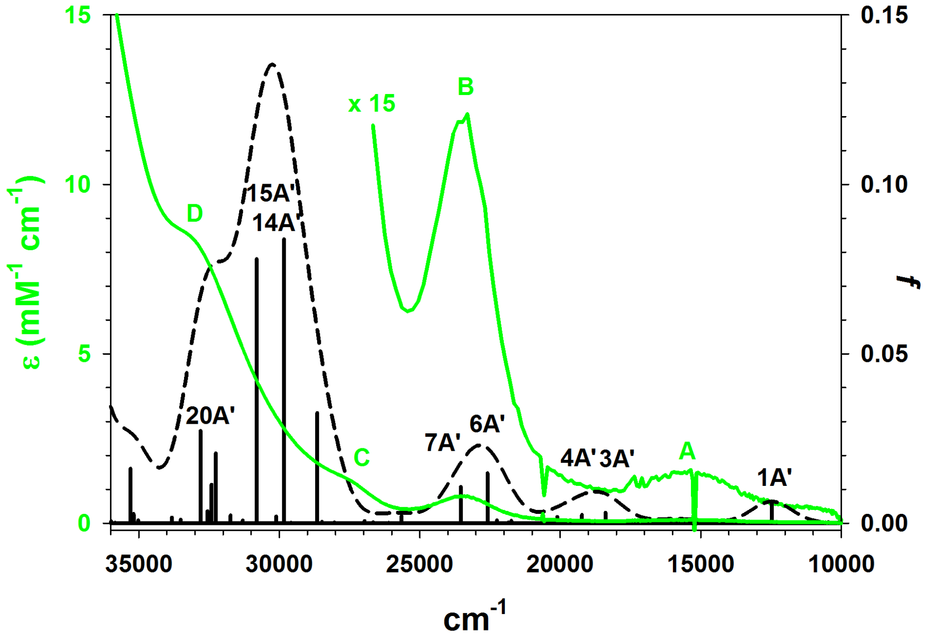

Figure S81. Comparison of experimental UV–vis–NIR spectrum of complex **1** (green), with four resolved features labeled A–D, and the calculated transitions for the corresponding DFT model *3* (black), rendered with an arbitrary linewidth of 1700 cm^{-1} (dashed), and with prominent transitions labeled (Table S50).

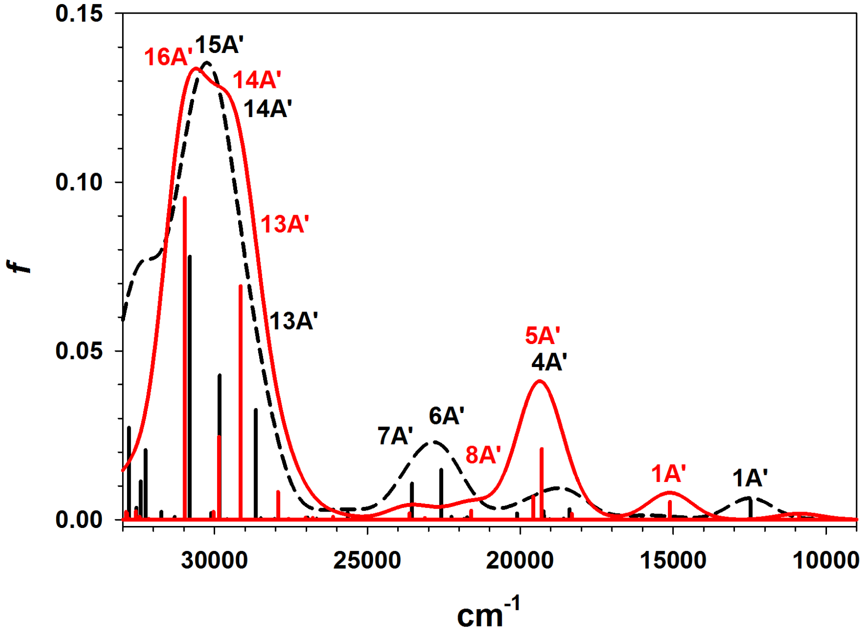

Figure S82. Calculated electronic spectra of the Ni(II) synthetic models *3* (dashed black line) and *5* (solid red line), rendered with arbitrary linewidths of 1700 cm^{-1}. All calculated transitions are listed in Tables S50 and S51, respectively.
